# Potent Anthranilic Anilide-Based TRPM4 Channel Inhibitors Identified by a Structure–Activity Relationship Study

**DOI:** 10.1021/acs.jmedchem.5c02015

**Published:** 2026-04-13

**Authors:** Christian E. Gerber, Bartlomiej S. Augustynek, Philipp Grossenbacher, Barbara Hauert, Simon A. Singer, Christine Peinelt, Martin Lochner

**Affiliations:** Institute of Biochemistry and Molecular Medicine, 27210 University of Bern, Bühlstrasse 28, 3012 Bern, Switzerland

## Abstract

We report the discovery of novel anthranilic anilide-based compound PBA (**118**), an inhibitor of Ca^2+^-activated monovalent cation channel TRPM4. PBA exhibits increased potency, ligand efficiency, aqueous solubility, and lipophilic ligand efficiency, as well as lower cytotoxicity compared to reference inhibitor NBA. The phenyl ring of the 4-chloro-2-(2-phenoxyacetamido)­benzoic acid scaffold was found to be essential for activity, and PBA resulted from a focused SAR study conducted on this ring. The *meta*-position proved to be favorable for substitutions, where lipophilic groups generally led to more potent inhibitors compared to polar substituents. X-ray structures and NMR studies of anthranilic anilides revealed bifurcated intramolecular hydrogen bonds stabilizing a “bent” conformation that might be important for their mode of action. The discovery of TRPM4 inhibitor PBA, together with other findings from our SAR study, will benefit future TRPM4 drug development efforts and research.

## Graphical abstract

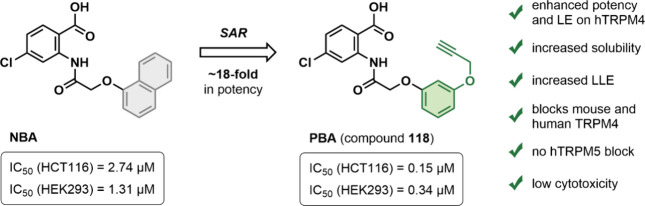

## Introduction

TRPM4 (transient receptor potential melastatin 4) is a nonselective, voltage-dependent cation channel, activated by an intracellular increase in Ca^2+^ concentrations.^1,2^ The channel conducts monovalent cations such as Na^+^ or K^+^ but is impermeable to bivalent cations such as Ca^2+^.^1^ The TRPM4 channel is a tetramer, each subunit consisting of a small extracellular domain, a transmembrane domain formed by six helices, and a larger intracellular domain.^3–6^ TRPM4 is involved in crucial physiological processes and has been associated with cardiovascular diseases,^7–9^ neuronal diseases,^10^ and cancer,^11,12^ which makes it an interesting research and drug target.^13,14^ In the past, several anthranilic acids^15–18^ and compounds such as glibenclamide^19^ or 9-phenanthrol^20^ (Chart 1) have been identified to inhibit TRPM4. Although the FDA-approved drugs glibenclamide^21^ and meclofenamate^18^ (Chart 1) were able to modulate TRPM4-related pathologies *in vivo*, these compounds are poor TRPM4 inhibitors and lack selectivity and specificity.^13,22^ Striving to discover inhibitors with higher potencies and selectivity, a previous study^23^ discovered anthranilic anilide-based TRPM4 inhibitors LBA and CBA (Chart 1) in a ligand-based virtual screening campaign. Subsequent synthetic alterations of the 4-chloro-2-(2-phenoxyacetamido)­benzoic acid scaffold were only tolerable to a limited extent without significantly losing activity. Substitutions or positional shift of the carboxylic acid or the chlorine erased activity, and modifications to the acetoxy linker resulted in weaker inhibitors. The phenoxy ring of the scaffold was more accepting for modifications, which yielded NBA as the most active compound (Chart 1).^23^

**Chart 1:**
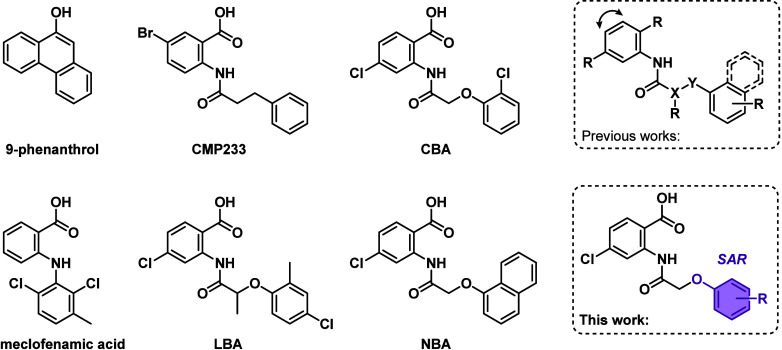
Structures of the Previously Discovered Potent Anthranilic Anilide-based TRPM4 Inhibitors LBA, CBA, NBA, and CMP233^a^

a 4-Chloro-2-((phenoxy)­acetamido)­benzoic acid scaffold with indicated sites of modifications in the previous and present SAR study.

Since their discovery, LBA, CBA, and NBA have been validated to specifically inhibit TRPM4 in patch clamp electrophysiology measurements using various cell types, either overexpressing or endogenously expressing hTRPM4.^23–26^ Interestingly, one study found that NBA blocked both human and mouse TRPM4, whereas CBA only blocked human TRPM4.^26^ In a follow-up study, the application of NBA showed a potent effect on disrupting atrioventricular conduction and induced third-degree atrioventricular block on 40% of wild-type mouse hearts.^27^ Clinically identified TRPM4 gain-of-function mutations lead to cardiac conduction disease,^28^ which might be addressed by selective inhibitors. TRPM4 was found to be highly expressed in colorectal cancer tissues and contributing to several cancer hallmark functions.^29^ In this context, NBA was able to significantly reduce cell proliferation and altered cell cycle of human colorectal cancer cells (HCT116) in a TRPM4-specific manner.^25^ Furthermore, another study demonstrated that CBA and NBA protect neuronal cells (HT22) from glutamate-induced cell death.^30^ Excessive glutamate release, as a consequence of ischemic stroke for instance, leads to glutamate excitotoxicity, which is also linked to the pathogenesis of neurodegenerative diseases. Studies have shown that TRPM4 forms a complex with glutamate NMDA receptors, which increases vulnerability of neurons to excitotoxicity.^31^ Collectively, these findings indicate that anthranilic anilide-based compounds have translational potential to modulate TRPM4 function in pathophysiological situations raising the demand for new analogues with improved potencies and ADMET properties. A recent study has presented a methyl ester analogue of NBA (**130** in this study) as a novel potent TRPM4 inhibitor with improved antiproliferative activity in prostate cancer cells.^32^ This finding is in contradiction to a previous and another recent SAR study, which demonstrated that the free carboxylic acid is essential for TRPM4 activity.^23,33^ This latter work has identified anthranilic anilide-based TRPM4 inhibitor CMP233 (Chart 1), showing a ∼6-fold higher potency compared to CBA and a similar potency to NBA.^33^ *In silico* docking identified a TRPM4 binding site for CMP233, presumably different from that of CBA, as mutations in this proposed binding pocket affected CMP233 activity, but had no effects on CBA inhibition.

The purpose of the present study was to further exploit the potential of the 4-chloro-2-(2-phenoxyacetamido)­benzoic acid scaffold. Our strategy was to revisit the phenoxy ring and conduct a focused, systematic SAR study by substituting different positions with groups of varying polarity, electronic property, and size (Chart 1), to fine-tune biological activity on TRPM4 and optimize general compound properties. During the experimental work, our collaborators identified the TRPM4 binding site for anthranilic anilides NBA (PDB ID: 8RD9) and IBA (PDB ID: 8RCU) (compound **103** from this work) by cryo-EM.^34^ This binding site was found to be in the transmembrane domain between the S3 and S4 helices, the S4–S5 linker, and the TRP helix, located in the inner membrane leaflet on the cytosolic side, which is very similar to the location identified by docking studies with CMP233.^33^ The derived binding orientations of NBA and IBA in these cryo-EM structures suggested that the 4-chloro-2-amidobenzoic acid moiety was embedded into a well-defined polar binding pocket, forming close polar contacts with surrounding amino acid side chains, whereas the iodophenyl (IBA) or naphthyl moieties (NBA) were tilted away from the protein, pointing toward a more spacious lipophilic pocket. This further supported our strategy to focus our structural changes on the phenoxy ring. We have also experimentally determined the solubility, lipophilicity, and solid-state and solution conformational preference of our most active anthranilic anilides. Collectively, these data suggest a pharmacological mode of action of these compounds and provide relevant properties for further drug development and their utility as molecular tools in biomedical research.

## Results and Discussion

### Design and Synthesis

For the planned conformational and solubility studies, we synthesized truncated CBA derivatives in which the carboxylic acid and/or phenoxy moiety were either missing or modified (Scheme 1). Acetylation of the commercially available aniline **1** with acetyl chloride gave anilide **2**, which both lacks the carboxylic acid and the phenoxy moiety. Reaction of **1** with chloroacetyl chloride delivered anilide **3**, which was subsequently *O*-alkylated with 2-chlorophenol to give carboxyl-truncated CBA derivative **4**. We synthesized 2-acetamido-4-chlorobenzoate-based compounds **6** and **7** by reacting commercially available methyl anthranilate **5** with acetyl chloride or chloroacetyl chloride. Compound **8** was synthesized by reacting carboxylic acid **9** with thionyl chloride to form acyl chloride **10**, which by reacting with methyl anthranilate **5** yielded compound **8**. Basic hydrolysis of compounds **6**–**8** yielded anthranilic anilides **11**–**14**. Compound **13** was isolated as a side product during basic hydrolysis of methyl anthranilate **7** in MeOH. Starting from commercially available 4-aminobenzoate **15** and conducting the same set of synthetic transformations delivered CBA congeners **17** and **20**, having the carboxylic acid group shifted to the *para*-position with respect to the amide.

**Scheme 1:**
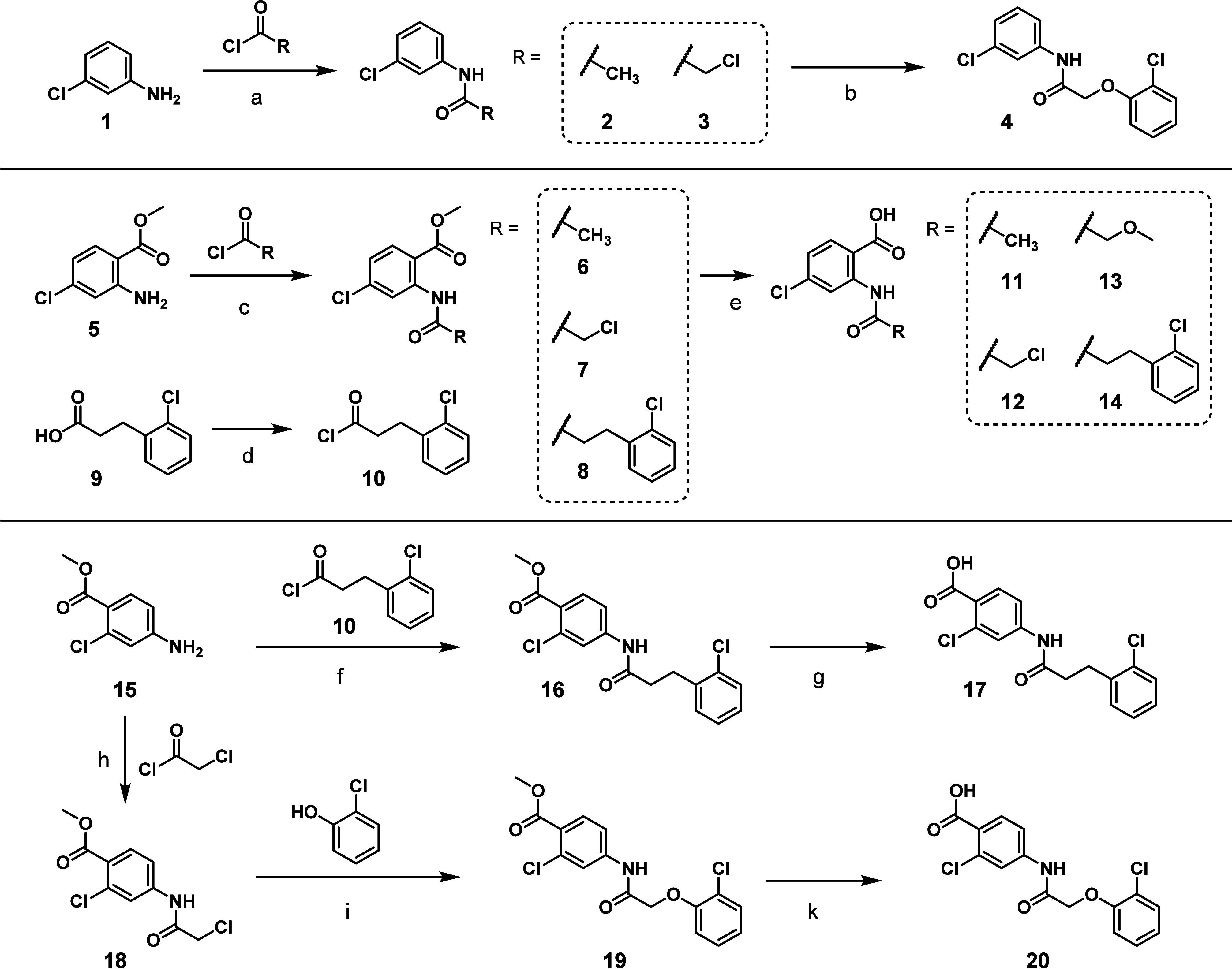
Reaction Pathways for the Synthesis of Truncated and Stripped Compounds **2**–**4**, **6**–**8**, **10**–**14**, and **16**–**20** ^a^

a Reagents and conditions: (a) for **2**: acetyl chloride, K_2_CO_3_, THF, rt, 60 min, quant.; for **3**: chloroacetyl chloride, THF, K_2_CO_3_, rt, 16 h, quant.; (b) 2-chlorophenol, K_2_CO_3_, DMF, 80 °C, 21 h, 34%; (c) for **6**: acetyl chloride, K_2_CO_3_, THF, rt, 16 h, 87%; for **7**: chloroacetyl chloride, THF, K_2_CO_3_, rt, 16 h, 81%; for **8**: **10**, THF, K_2_CO_3_, rt, 2 h, 70%; (d) thionyl chloride, DCM, DMF (cat.), reflux, 17 h; (e) for **11**: KOH, MeOH/H_2_O (5:1), 65 °C, 10 min, 93%; for **12** and **13**: KOH, MeOH/H_2_O (5:1), 65 °C, 3 h, **12** (35%) and **13** (17%); for **14**: KOH, MeOH/H_2_O (5:1), 65 °C, 10 min, 97%; (f) **10**, K_2_CO_3_, THF, rt, 26 h, 93%; (g) KOH, MeOH/H_2_O (5:1), 65 °C, 2 h, 96%; for (h – k) see ref.^23^

For the synthesis of the extended library of 4-chloro-2-(2-phenoxyacetamido)­benzoic acids with various substituents on the phenoxy ring, we adapted and optimized the previously published procedure^23^ that was used for the synthesis of CBA and NBA (Scheme 3). First, we synthesized a series of substituted phenols or naphthol nucleophiles (Scheme 2) by reacting commercially available hydroxyphenols **21**–**23**, **28**, or hydroxynaphthols **30** and **31** with propargyl bromide or 1-bromobut-2-yne under basic conditions to give the corresponding substituted phenols **24** – **27**, **29**, and naphthols **32** and **33**. Phenols **36** and **39** were synthesized according to Scheme 2. Methyl anthranilate **7** was reacted with substituted phenols under basic *O*-alkylation conditions to yield the corresponding methyl 4-chloro-2-(2-phenoxyacetamido)­benzoate intermediates **41**–**85** (Scheme 3). The final anthranilic anilide-based compounds **86**–**128** were obtained via basic hydrolysis of the corresponding methyl esters **41**–**85** and isolated in high purity (>95% by HPLC) after direct precipitation upon addition of aqueous hydrochloric acid. Some precipitates needed further purification by flash column chromatography or recrystallization to achieve the required purity.

**Scheme 2:**
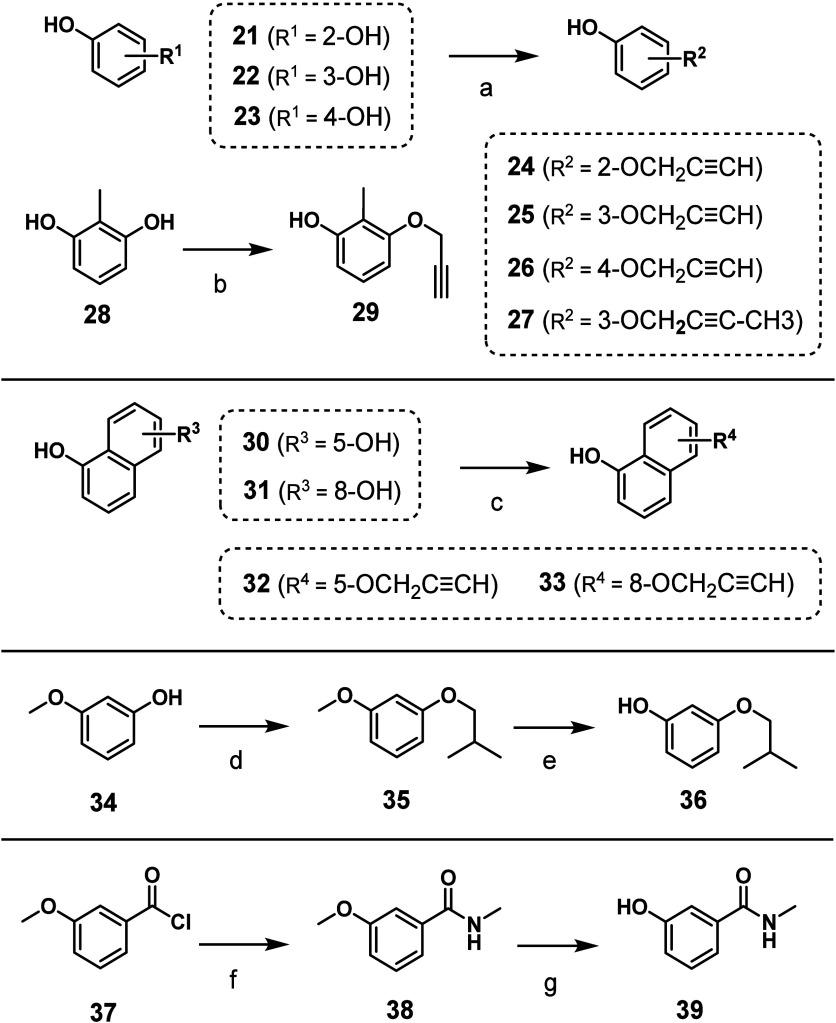
Reaction Pathways for the Synthesis of Substituted Phenols **24**–**27**, **29**, **32**, **33**, **36**, and **39** Used in the Synthesis of Final Compounds **111** and **117**–**122** ^a^

a Reagents and conditions: (a) for **24**-**26**: propargyl bromide, K_2_CO_3_, acetone, 65 °C, 16 h, 26–32%; for **27**: **22**, 1-bromobut-2-yne, K_2_CO_3_, acetone, 65 °C, 6 h, 40%; (b) propargyl bromide, K_2_CO_3_, acetone, 65 °C, 16.5 h, 29%; (c) propargyl bromide, K_2_CO_3_, acetone, 65 °C, 19–22.5 h, 25–65%; (d) 1-bromo-2-methylpropane, K_2_CO_3_, DMF, 80 °C, 22 h, 65%; (e) BBr_3_, DCM, 0 °C, 4 h, 11%; (f) K_2_CO_3_, methylamine hydrochloride salt, 18-crown-6, Et_2_O, rt, 96 h, 86%; (g) BBr_3_, DCM, 0 °C, 4.5 h, 96%.

**Scheme 3:**
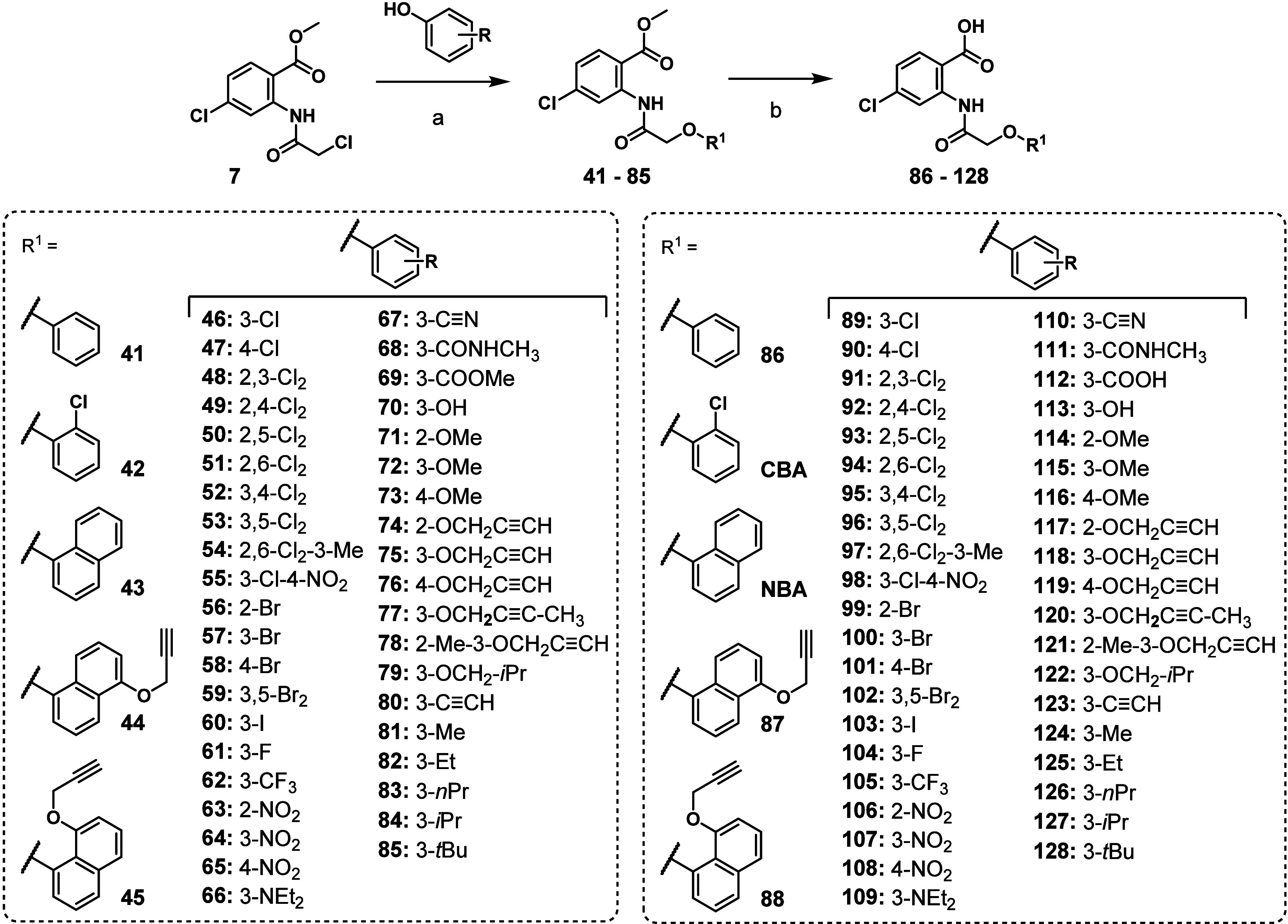
Synthetic Routes for Methyl 2-(2-Aryloxyacetamido)-4-chlorobenzoates **41**–**85**, 2-(2-Aryloxyacetamido)-4-chlorobenzoic Acids CBA, NBA, and Anthranilic Anilides **86**–**128** ^a^

a Reagents and conditions: (a) K_2_CO_3_, substituted phenol, DMF, 80 °C, 20 min–16 h, 20–98%; (b) KOH, MeOH/H_2_O (5:1), 65 °C, 20 min – 5 h, 24% – quant.

### HEK293 Cell-Based TRPM4 Activity Screening

We assessed our synthesized compounds for their ability to inhibit the TRPM4 ion channel *in vitro* using a fluorescence-based sodium influx assay on HEK293 cells, overexpressing hTRPM4. The methods for this assay were taken from previous work^23^ and adapted according to our needs (see the Experimental Section). We observed a rapid fluorescence response upon Na^+^-addition in Ca^2+^-stimulated controls (full TRPM4 activation) and a much less profound and slower response in the nonstimulated controls (no TRPM4 activation) (Figures S1 and S2). Preincubation of cells with TRPM4 inhibitor NBA at 10 μM concentration reduced the fluorescence signal of Ca^2+^-stimulated cells almost to the same level as observed for the nonstimulated controls (Figures S1 and S2) and was therefore used as a third control representing full inhibitor-mediated TRPM4 inactivation. To verify target engagement, we repeated the identical assay with HEK293 WT cells. The fluorescence signal increase upon Na^+^ addition in nonstimulated WT cells was comparable to the signal increase in nonstimulated hTRPM4-overexpressing cells (Figure S3A), whereas signal increase in Ca^2+^-stimulated WT cells was significantly lower and slower compared to Ca^2+^-stimulated hTRPM4-overexpressing cells (Figure S3B). WT cells responded to Ca^2+^ activation and this additional influx signal was slightly reduced in NBA-treated WT cells, indicating endogenous expression of TRPM4 (Figure S3B,C). This observation is in line with results from previous studies, reporting low levels of endogenous TRPM4 expression in HEK293 WT cells.^23^ Na^+^-influx reduction in NBA-treated and Ca^2+^-stimulated hTRPM4-overexpressing cells was significantly higher compared to Ca^2+^-stimulated WT cells (Figure S3C). However, the addition of NBA, even at concentrations up to 20 μM, did not completely reduce the signal back to the level of nonstimulated cells (Figures S3C and S4) and this residual signal increase did not differ between the WT and hTRPM4-overexpressing cell lines (Figure S3D). This indicates that some Na^+^ enters HEK293 cells via unknown Ca^2+^-activated but TRPM4-independent mechanisms. Since the aim of our assay was to quantify the inhibition of TRPM4 function, we decided to normalize the responses obtained for the test compounds to the response of the NBA-mediated full-block controls (10 μM NBA added to ionomycin-stimulated hTRPM4 cells). To exclude interference of potentially fluorescent compounds during measurements, all synthesized compounds were characterized by UV–vis measurements, which showed that the compounds absorbed light neither in the region of the excitation wavelength (482.5 nm ± 12.5) nor in the region of the emission wavelength (545 nm ± 30 nm) of the Na^+^-sensitive fluorescent dye (Figure S19).

### The Phenoxy Ring of the 4-Chloro-2-(2-phenoxyacetamido)­benzoic Acid Scaffold Is Essential for TRPM4 Inhibition

We first validated the 4-chloro-2-(2-phenoxyacetamido)­benzoic acid scaffold as a core platform with minimal TRPM4 inhibition activity for further SAR modifications. To address the contradictory findings between previous SAR studies,^23,33^ where substitutions of the carboxylic acid resulted in dramatically reduced activities and a more recent study,^32^ in which a methyl ester analogue of NBA (**130**, Figure S13A) was reported as a potent TRPM4 inhibitor, we synthesized methyl ester analogues of CBA and NBA (**42** and **43**, respectively) and tested their TRPM4 activity. Both methyl ester compounds **42** and **43** were not active at 5 and 10 μM concentrations (Figure S5). We also resynthesized the reported methyl ester compound **130**,^32^ but it was completely inactive in our TRPM4-specific assays (Figure S13B–D). Only after hydrolysis of the methyl ester to the corresponding carboxylic acid **131** was activity restored (Figure S13A,D), albeit to a lesser extent than that of NBA. Complete removal of the carboxylic acid results in complete loss of activity, as shown with CBA analogue **4** (Figure S5). This re-emphasizes the essentiality of the free carboxylic acid for inhibitory activity and is in line with the proposed binding contacts of this functional group with Arg-1064 in the cryo-EM structures of TRPM4 bound with NBA or IBA.^34^ Removing the phenoxy moiety (**11** and **12**) or the phenyl ring (**13**) resulted in completely inactive compounds (Table 1, Figure S5). Putting back an unsubstituted phenyl ring (**86**) was able to rescue TRPM4 inhibition activity (Table 1, Figure S5). We thus concluded that the 4-chloro-2-(2-phenoxyacetamido)­benzoic acid structure is the minimal pharmacophore for TRPM4 inhibition and figured that the phenoxy ring offers a straightforward moiety for compound optimization (Table 2, Figures S6 and S7). This localized extension SAR approach was later supported by the solved cryo-EM structures, which indicated that the aryloxy rings of NBA and IBA point into a rather spacious pocket.^34^

**Table 1:**
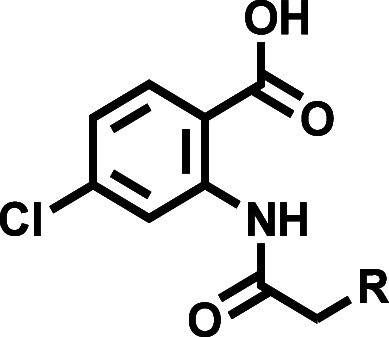

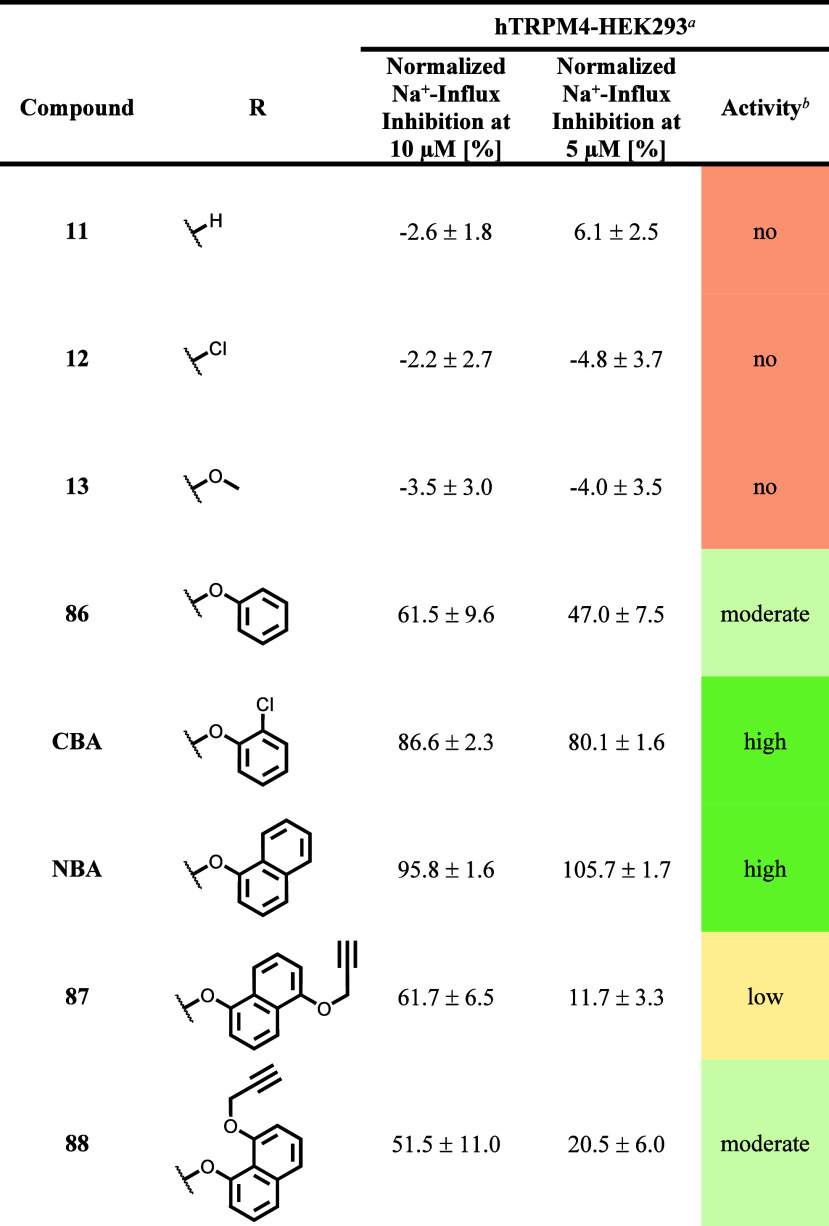
TRPM4 Inhibitory Activities of Reference Compounds CBA, NBA, and Compounds **11**–**13**, **86**–**88** Sharing a 4-Chloro-2-acetamidobenzoic Acid Scaffold

a TRPM4 inhibition of compounds was tested *in vitro* at single concentrations of 10 and 5 μM in the fluorescence-based Na^+^-influx assay using HEK293 cells stably overexpressing hTRPM4. All measured values were normalized to 25 μM ionomycin + 1% DMSO as the fully activated TRPM4 control and 25 μM ionomycin + 10 μM NBA as the full-block control to calculate TPRM4 % inhibition where 100% ≙ full inhibition and 0% ≙ no inhibition. All values are mean ± SEM of at least 9 technical replicates from 3 independent biological replicates. An outlier test with the ROUT method (*Q* = 1), an ordinary one-way ANOVA test, and Dunnett’s multiple comparison test were conducted for statistical significance using GraphPad Prism.

b Compounds were classified as having “no activity” (mean Na^+^-influx inhibition did not differ significantly compared to DMSO controls), “low activity” (<20% inhibition), moderate activity (20–80% inhibition), or high activity (>80% inhibition), at 5 μM concentrations.

**Table 2:**
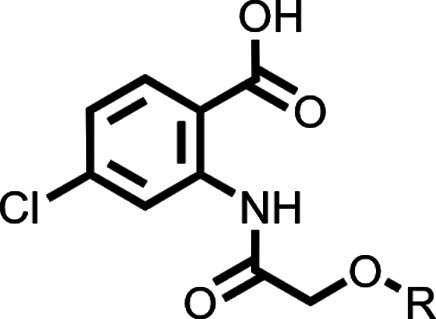

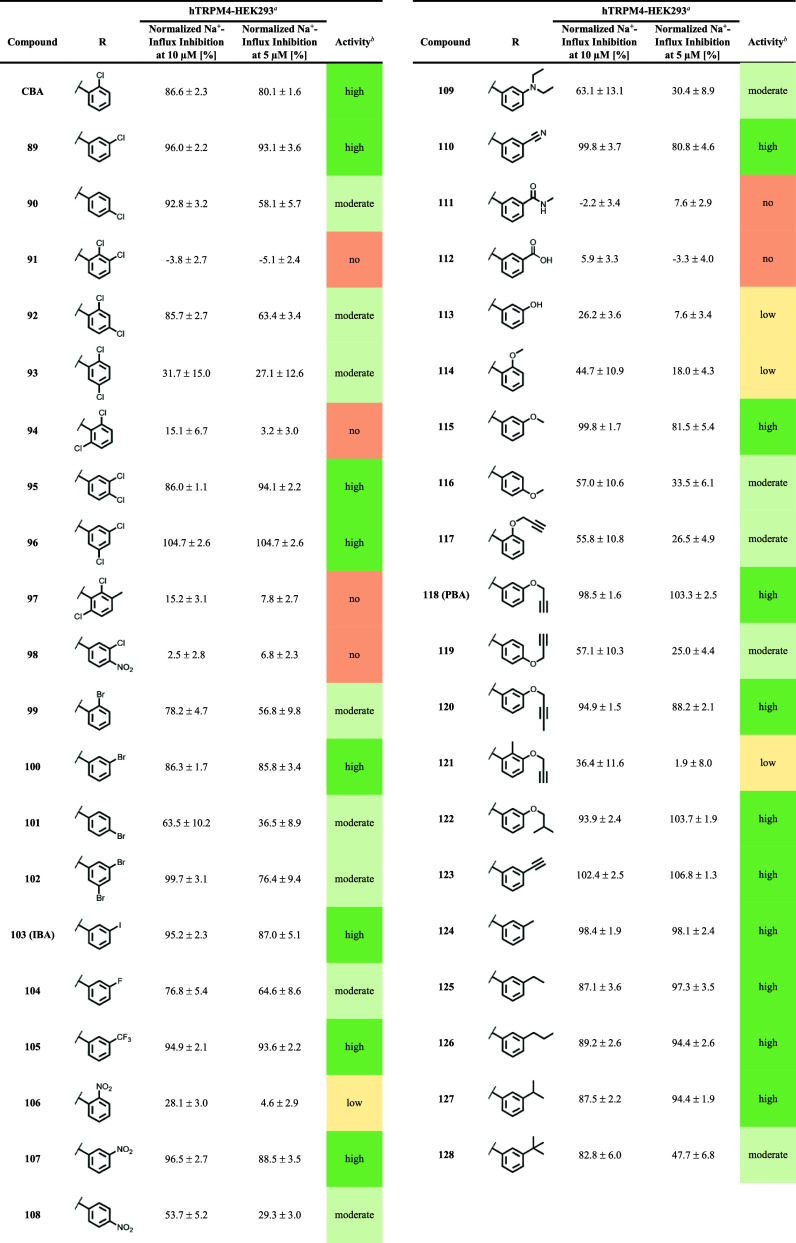
TRPM4 Inhibitory Activities of CBA and Compounds **89**–**128** Sharing a 4-Chloro-2-(2-phenoxyacetamido)­benzoic Acid Scaffold

a TRPM4 inhibition of compounds was tested *in vitro* at single concentrations of 10 and 5 μM in the fluorescence-based Na^+^-influx assay using HEK293 cells stably overexpressing hTRPM4. All measured values were normalized to 25 μM ionomycin + 1% DMSO as the fully activated TRPM4 control and 25 μM ionomycin + 10 μM NBA as the full-block control to calculate TPRM4 % inhibition where 100% ≙ full inhibition and 0% ≙ no inhibition. All values are mean ± SEM of at least 9 technical replicates from 3 independent biological replicates. An outlier test with the ROUT method (*Q* = 1), an ordinary one-way ANOVA test, and Dunnett’s multiple comparison test were conducted for statistical significance using GraphPad Prism.

b Compounds were classified as having “no activity” (Na^+^-influx inhibition did not differ significantly compared to DMSO controls), “low activity” (<20% inhibition), moderate activity (20–80% inhibition), or high activity (>80% inhibition), at 5 μM concentrations.

### SAR Study on the Phenoxy Ring of the 4-Chloro-2-(2-phenoxyacetamido)­benzoic Acid Scaffold

To identify the ideal position for substituents on the phenoxy ring, we introduced single chloro-, bromo-, methoxy-, propargyloxy-, and nitro-substituents in either the *ortho*-, *meta*-, or *para*-position. For all these substituents, the *meta*-position clearly proved to be advantageous, compared to *ortho* or *para* (Table 2 and Figure S6). For the most active compounds, we ran full dose–response curves to determine IC_50_ values (Table 3 and Figure S9) and the positional trend observed at single compound concentrations was also reflected in the IC_50_ values. For instance, *meta*-chloro compound **89** showed a ∼2-fold greater potency than *ortho*-chloro compound CBA and a ∼3.4-fold greater potency than *para*-chloro compound **90** (Table 3). Consequently, we introduced other substituents in the *meta*-position of the phenoxy ring. We completed the halogen series by synthesizing compounds **100** (*m*-Br), **103** (*m*-I), and **104** (*m*-F). While all these *meta*-halogenated compounds showed high activities at single concentrations (Table 2, Figure S7), the determination of their IC_50_ values revealed a decrease in potency in the order *m*-Cl > *m*-Br > *m*-I > *m*-F (Table 3 and Figure S9). Further, we investigated some polysubstituted derivatives. Only the 3,4-dichloro **95** and 3,5-dichloro **96** congeners showed high activity at single concentrations, whereas other substitution patterns resulted in compounds with only moderate (**92**, **93**, and **102**) or low to no (**91**, **94**, **97**, and **98**) activity (Table 2). To probe the influence of size and polarity of substituents, we mainly compared *meta*-substituted compounds. For instance, the nitrogen-containing compounds **107** (*m*-NO_2_) and **110** (*m*-CN) both exhibited high activities at single concentrations. However, both compounds were not able to exceed the potency of the most potent halogenated compound **89** (*m*-Cl). Interestingly, the bulkier amine substituent in compound **109** (*m*-NEt_2_) resulted in markedly reduced activity. This poor acceptance of bulky substituents was also reflected by the significantly reduced activities of the two extended NBA derivatives **87** (5-propargyloxy) and **88** (8-propargyloxy) (Table 1). The *meta*-*O*-derivatized compounds **115** (*m*-OMe), **118** (*m*-propargyloxy), **120** (*m*-2-butynyloxy), and **122** (*m*-O*i*Bu) all exhibited high activities at single concentrations, except for the more polar compound **113** (*m*–OH) showing only low activity (Table 2). In this series, propargyloxy-substituted **118** stood out as being the most potent compound with ∼9.5-fold higher activity compared to shorter methoxy-substituted **115**. Further modifications of **118**, like an additional methyl group in the *ortho*-position (**121**) or elongation of the propargyl needle (**120**), markedly reduced the TRPM4 inhibitory activity compared to **118**. A similar reduction in potency was observed by replacing the linear alkyne moiety of **118** by a branched isobutyl group (**122**). Increasing the polarity of the *meta*-substituent further with compounds **111** (*m*-CONHMe) and **112** (*m*-COOH) completely abolished TRPM4 inhibitory activity (Table 2). On the other hand, compounds with apolar substituents, such as **105** (*m*-CF_3_), **123** (*m*-ethynyl), **124** (*m*-Me), **125** (*m*-Et), **126** (*m*-*n*Pr), and **127** (*m*-*i*Pr), all exhibited high activity at a single concentration. Measurements of the IC_50_ values revealed submicromolar potencies for compounds **123** and **127**, as well as high potency for compounds **105**, **124**, **125**, and **126**. In terms of activity with respect to the length of the alkyl restat the *meta*-position, we observed the order Et > *n*Pr > Me (Table 3). The branching of the *meta*-alkyl group also plays an important role. Particularly striking is the comparison between isomers **126** (*m*-*n*Pr) and ∼2.2-fold more potent **127** (*m*-*i*Pr). Further branching as in **128** (*m*-*t*Bu) in turn diminished TRPM4 inhibitory activity (Table 2). The most promising compound resulting from this localized SAR study was compound **118**, which we named PBA, with a significantly increased potency at TRPM4 in comparison to benchmark inhibitor NBA. As the structure of **118** did not increase the heavy atom count and therefore retained the size of the scaffold, its ligand efficiency (LE = 0.36) was improved compared to NBA (LE = 0.32).

**Table 3:**
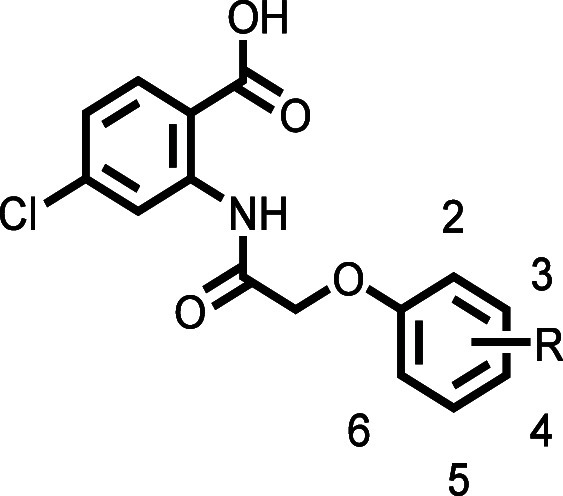

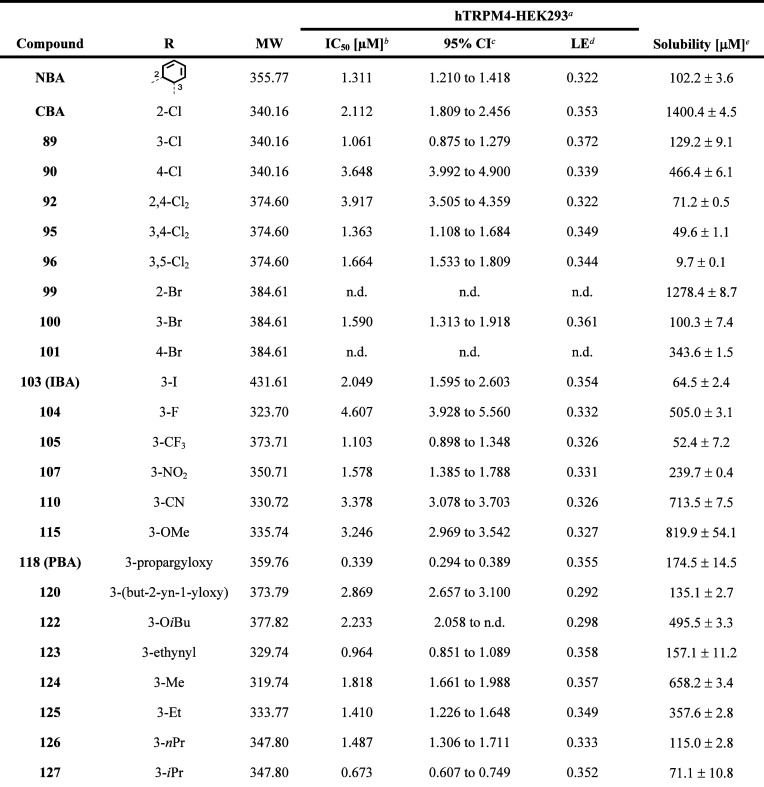
TRPM4 IC_50_ Values, Ligand Efficiencies, and Solubilities of the Most Active Hit Compounds

a Compound activities were measured *in vitro* in 2-fold dilution series from 10 to 0.078125 μM using the fluorescence-based Na^+^-influx assay on HEK293 cells stably overexpressing hTRPM4. 25 μM ionomycin + 1% DMSO was used as the fully activated TRPM4 control, and all measured activities were normalized to the activity of 25 μM ionomycin + 10 μM NBA as the full-block control.

b IC_50_ values were calculated with the Hill equation using the four-parameter logistic model (4PL) with a variable slope.

c 95% confidence intervals of IC_50_ values were calculated using an asymmetrical method.

d Ligand efficiencies (LE) were calculated using the measured IC_50_ values and the total number of non-hydrogen atoms per molecule.

e Solubilities (mean ± SD) of compounds were determined in PBS buffer (pH = 7.4), and calibration lines and sample concentrations were measured by HPLC (*n* = 3 × 3 = 9). All solvents and buffers used contained 1% DMSO.

We used the cryo-EM structure of human TRPM4 bound to NBA (PDB ID: 8RD9) as the docking template to predict binding poses for PBA (**118**). In the proposed *in silico* binding model, which is in agreement with our experimental SAR findings, the carboxylate of PBA is surrounded by polar and basic functional groups, the phenoxy ring by hydrophobic residues, and the *meta*-terminal alkyne points into a “hole” lined with hydrophobic side chains (Figure S25B,C).

### **118** (PBA) Potently Blocks Endogenous TRPM4 Currents in Human Colorectal Cancer Cells

We evaluated four of the most potent compounds identified in our fluorescence-based screening assay using whole-cell patch-clamp electrophysiology in human colorectal cancer cells (HCT116), which endogenously express TRPM4. The measured currents under these conditions are TRPM4-specific, as CRISPR/Cas9-mediated knockout of the TRPM4 gene abolishes them completely (Figure 1A). This is in line with our previous findings.^29^ Furthermore, re-expression of TRPM4 in the knockout HCT116 cell line restores the current, demonstrating that it is directly dependent on TRPM4 channel activity (Figure S11A). Importantly, the rescued current remains fully sensitive to inhibition by compound **118** (PBA), confirming both the identity of the current and the continued pharmacological responsiveness of the channel (Figure S11B). Compounds **118** (PBA), **123**, **127**, and reference compound NBA all reduced endogenous TRPM4 currents at 1 μM concentrations (Figure 1 and Figure S12). NBA reduced current density by 33% at 1 μM concentration, whereas both **118** (95% reduction) and **127** (49% reduction) demonstrated greater activity than NBA at this concentration. Compound **123** reduced current density by only 15%, while compound **105** did not significantly reduce currents, despite both compounds showing slightly higher potencies than NBA in the fluorescence-based assay (Table 3). These discrepancies could be attributed to the two different assay formats, specifically preincubation of compounds prior to measurements in the FLIPR assay versus direct compound addition during measurements in patch-clamp electrophysiology. The apparent incomplete block observed likely reflects currents from intravesicular channels that are inserted into the plasma membrane via exocytosis and are not fully blocked due to the shorter compound incubation time.^35,36^ Notably, compound **118** (PBA) demonstrated high potency both in the fluorescence-based Na^+^-influx assay and in whole-cell patch-clamp electrophysiology. Dose–response measurements for NBA and compound **118** (PBA) in whole-cell patch-clamp recordings from HCT116 cells revealed dose-dependent reductions in TRPM4-mediated currents, with IC_50_ values comparable to those obtained in the fluorescence-based screening assay (**118**: IC_50_ (HCT116) = 0.15 μM; IC_50_ (HEK293) = 0.34 μM versus NBA: IC_50_ (HCT116) = 2.74 μM; IC_50_ (HEK293) = 1.31 μM). This translates into an approximately 18-fold increase in inhibitory potency of compound **118** (PBA) compared to NBA in patch-clamp measurements on HCT116 cells (Figure 1). The determined TRPM4 inhibition activity of **118** (PBA) is on par with the reported activity of anthranilic anilide CMP233 (IC_50_ = 0.15 μM; TRPM4-HEK293, automated QPatch assay) and slightly lower than benzoxazole CMP353 (IC_50_ = 0.08 μM), presented in the same study.^33^

**Figure 1:**
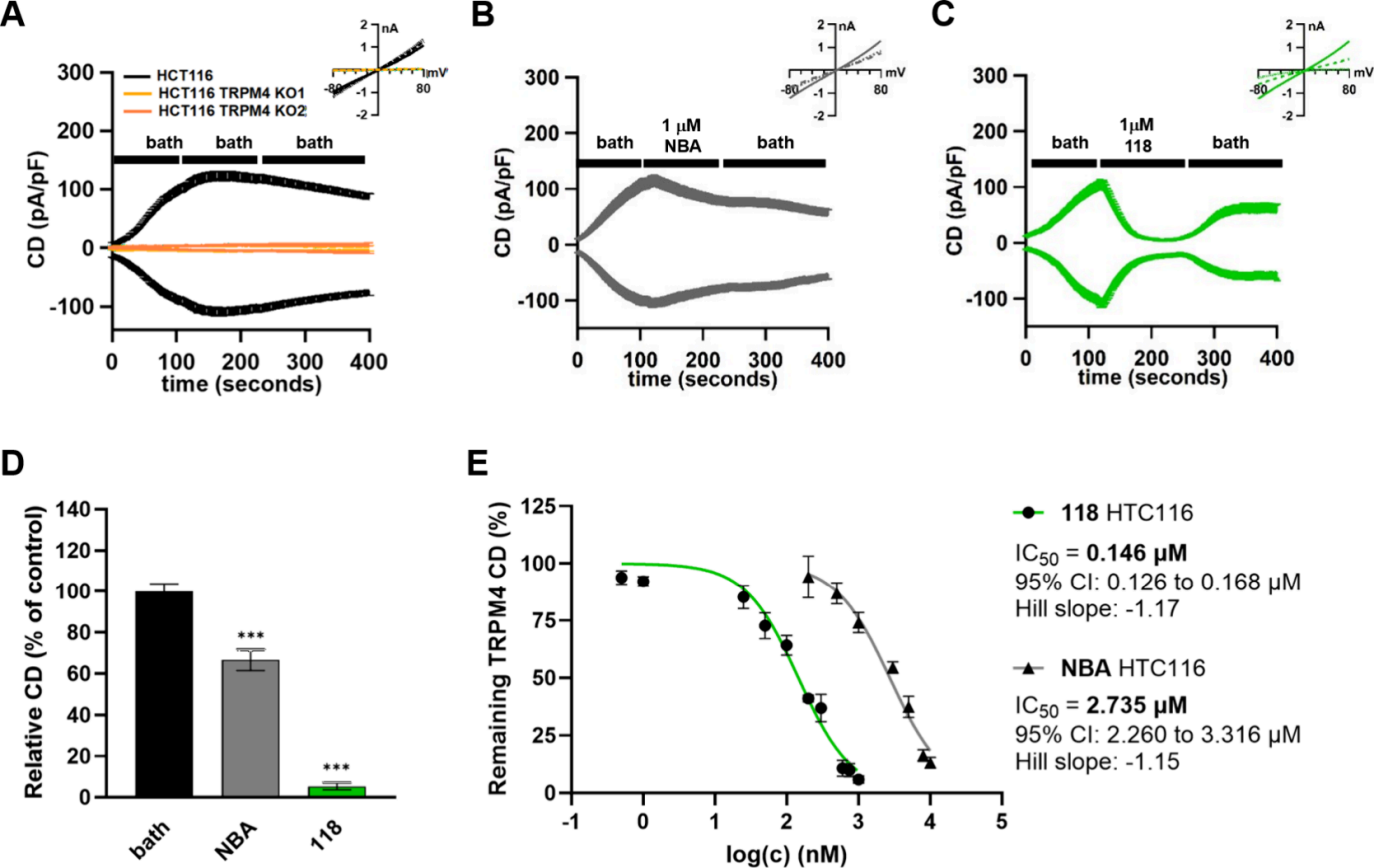
Novel inhibitor compound **118** (PBA) blocks endogenous TRPM4 currents in HCT116 cells. (A–C) Whole-cell patch-clamp recordings from HCT116 cells with 10 μM free Ca^2+^ in the pipet. Currents were normalized to cell size and plotted as current densities (CD; mean ± SEM) over time. Insets show current–voltage (*I*/*V*) relationships recorded before drug application (solid line, *t* = 100 s), during drug application (dotted line, *t* = 220 s), and after washout with bath solution (dashed line, *t* = 300 s). (A) Control (bath solution applied from 120–240 s; HCT116, black traces, *n* = 13; HCT116 TRPM4 KO1, golden traces, *n* = 11; HCT116 TRPM4 KO2, orange traces, *n* = 11). (B) 1 μM NBA applied from 120–240 s (*n* = 22). (C) 1 μM compound **118** (PBA) applied from 120–240 s (*n* = 15). (D) Corresponding TRPM4 current inhibition by 1 μM NBA and 1 μM compound **118** (PBA), expressed as a percentage of control. Student’s *t*-tests were conducted for statistical significance (_***_: *p* < 0.001). (E) Dose–response analysis showing average TRPM4 CD (mean ± SEM, % of control) plotted against compound **118** (PBA) and NBA concentrations (μM). Compound **118** (PBA) concentrations: 1 (*n* = 15), 0.75 (*n* = 6), 0.6 (*n* = 5), 0.3 (*n* = 7), 0.2 (*n* = 7), 0.1 (*n* = 11), 0.05 (*n* = 7), 0.025 (*n* = 9), 0.001 (*n* = 6), 0.0005 (*n* = 6). NBA concentrations: 10 (*n* = 5), 8 (*n* = 7), 5 (*n* = 7), 3 (*n* = 7), 1 (*n* = 18), 0.5 (*n* = 7), 0.2 (*n* = 6). Dose–response curves were fitted using the Hill equation, and IC_50_ values were determined.

### **118** (PBA) Inhibits Both Human and Mouse TPRM4 but not Human TRPM5

To assess the selectivity of **118** (PBA), we measured its activity on TRPM5, the closest relative of TRPM4, having high sequence homology and structural and functional similarities. TRPM4 and TRPM5 are the only TRP channels selective for monovalent cations, and both channels are activated by intracellular Ca^2+^. We measured the activity of **118** (PBA) and NBA in our fluorescence-based Na^+^-influx assay on HEK293 cells, stably overexpressing hTRPM5. Triphenylphosphine oxide, a selective TRPM5 inhibitor,^37^ and CBA, previously reported to be inactive on TRPM5,^23^ were included as assay control compounds. Both **118** (PBA) and NBA did not inhibit TRPM5-mediated Na^+^-influx at 5 and 10 μM concentrations (Figure S10A). While NBA blocked human and mouse TRPM4 overexpressed in tsA201 cells, CBA was inactive at mouse TRPM4 in a previously reported patch-clamp electrophysiology assay.^26^ To address potential species-dependent differences, we determined the activity of **118** (PBA) on mouse TRPM4, overexpressed in tsA201 cells, in the fluorescence-based Na^+^-influx assay. CBA and NBA were included as assay control compounds at 5 and 10 μM concentrations. As reported in the electrophysiology study, CBA was inactive at mTRPM4 in the FLIPR assay but NBA and **118** (PBA) both efficiently inhibited mTRPM4, with **118** (PBA) exhibiting higher activity than NBA (Figure S10B).

### **118** (PBA) Demonstrates Higher Aqueous Solubility and Lower Lipophilicity than NBA

We determined the solubility of our most active anthranilic anilides in PBS + 1% DMSO (pH = 7.4) at 20 °C and obtained aqueous solubilities ranging from 10 to 1400 μM (Table 3). These rather low solubilities are in line with a high melting point observed for CBA (210.1–211.7 °C) and high decomposition temperatures observed for **118** (>221 °C), NBA (>241 °C), and **89** (>250 °C). High melting points have been reported for this compound class previously,^38^ suggesting high lattice energies. The position of the phenoxy substituent has a strong influence on solubility. For instance, we observed a ∼10.9-fold decrease in solubility when changing the position of the chlorine atom from *ortho* in CBA to *meta* in **89** and then a 3.6-fold increase when changing the chlorine atom position from *meta* to *para* in **90**. The same U-shaped trend was observed for the slightly less soluble brominated analogues **99** (*o*-Br), **100** (*m*-Br), and **101** (*p*-Br) (Figure S16B). When comparing the phenoxy ring *meta*-substituted compounds, the solubility for halogenated compounds decreased in the order F > Cl > Br > I > CF_3_ (Figure S16C). Similarly, compounds with aliphatic substituents displayed a decrease in solubility in the order Me > Et > ethynyl > *n*Pr > *i*Pr (Figure S16F). The solubility of **115** (*m*-OMe), **107** (*m*-NO_2_), and **118** (*m*-propargyloxy) with polar *meta*-substituents on the phenoxy ring was higher compared to *meta*-halogenated compounds (Figure S16E) but not significantly higher compared to compounds with aliphatic *meta*-substituents. Notably, measured solubilities did not correlate with inhibitory potency (*R* ^2^ = 0.1302) determined by the Na^+^-influx assay, in which cells are preincubated with the compounds, meaning that assay results were not governed by compound solubility (Figure S16G). In this regard, compound **118** (PBA) did not only show improved potency but also increased aqueous solubility by a factor of ∼1.7, compared to reference compound NBA. To further compare compound **118** (PBA) to NBA, we measured their distribution coefficients at pH = 7.4 (log *D*_7.4_) with the shake flask method using *n*-octanol/PBS buffer. Both NBA (log *D*_7.4_ = 2.02) and **118** (log *D*_7.4_ = 1.53) can be considered lipophilic compounds, with **118** (PBA) being less lipophilic than NBA, and consequently exhibiting higher lipophilic ligand efficiency (LLE) than NBA. Experimentally determined lipophilicity was compared to calculated partition coefficients (cLog *P*, using the SwissADME tool (https://www.swissadme.ch)), which gave a similar lipophilicity difference between both compounds (cLog *P*(NBA) = 3.59 and cLog *P*(**118**) = 2.89). This increase in solubility and decrease in lipophilicity can partly be explained in terms of compound structure since **118** (PBA) possesses less aromatic rings (*N*_**118**_ = 2 vs *N*_NBA_ = 3), more rotatable bonds (*N*_**118**_ = 8 vs *N*_NBA_ = 6), a higher fraction of sp^3^-hybridized carbon atoms (Csp^3^_**118**_ = 0.11 vs Csp^3^_NBA_ = 0.05), and a higher calculated topical polar surface area^39^ (TPSA_**118**_ = 87.69 Å^2^ vs TPSA_NBA_ = 78.46 Å^2^),^40^ as well as one additional hydrogen bond acceptor (propargyl oxygen). To complement these findings, additional ADME parameters for **118** (PBA) were determined in human plasma and human liver microsomes. Human plasma protein binding was found to be 99.7% (Table S5), and **118** (PBA) showed high stability in human plasma, with no significant degradation observed over a period of 120 min (Table S6). **118** (PBA) displayed a half-life of *t*_1/2_ = 20.6 min in human liver microsomes and an intrinsic clearance of CL_*int*_ = 335.8 μL/min/mg (Table S7).

### *In Vitro* Toxicity Evaluation of **118** (PBA)

Cytotoxicity in HeLa cells of both reference compounds CBA and NBA has previously been determined, and no significant cytotoxicity was observed at lower concentrations (≤10 μM); however at higher concentrations (up to 100 μM), NBA started to exhibit significantly higher cytotoxicity compared to CBA.^23^ To probe possible cytotoxicity of **118** (PBA), we conducted an *in vitro* resazurin-based cell viability assay with HEK293 WT cells where **118** (PBA) exhibited no cytotoxic effects up to 50 μM concentrations. Only at high concentrations of 100 μM (>600-fold concentration of its IC_50_ at TRPM4), reduced cell viability of 67% was observed (Figure S14). Remarkably, in the same assay, NBA reduced cell viability already at 12.5 μM concentrations (77% viability), which decreased further at higher concentrations and resulted in 0% viability at 100 μM concentrations (Figure S14). These measurements therefore demonstrated a significantly lower cytotoxicity of **118** (PBA) in HEK293 cells compared to NBA and no cytotoxicity of **118** (PBA) at assay concentrations. To explore further safety parameters of compound **118** (PBA) with respect of its potential use *in vivo*, we investigated possible off-target effects on three cardiac ion channels hERG (K_V_11.1), hNa_V_1.5, and hCa_V_1.2 and observed no significant inhibition up to 10 μM compound concentration compared to DMSO vehicle controls (Tables S1–S3). As another safety parameter, inhibition of a panel of cytochrome P450 enzymes (CYP 1A2, 2B6, 2C8, 2C9, 2C19, 2D6, and 3A4) by **118** (PBA) was determined. At 10 μM concentration, **118** (PBA) only inhibited CYP2C8 and CYP2C9 to a significant extend (>50% of control values, Table S4).

### Conformational Stability through Intramolecular Bifurcated Hydrogen Bonding

To the best of our knowledge, crystal structures of anthranilic anilide-based TRPM4 inhibitors, such as NBA or CBA, have not been published so far. We obtained single crystals for NBA and CBA, as well as 11 anthranilic anilide congeners, and resolved their structures by X-ray crystallography. All crystal structures indicated strong intramolecular bifurcated hydrogen bonds between the amide hydrogen and the carboxyl oxygen, as well as between the amide hydrogen and the phenoxy oxygen, with hydrogen bond lengths of 1.775–2.012 and 2.088–2.245 Å, respectively (Figure 2 and Table S8). Thus, the hydrogen bond between amide hydrogen and carboxyl oxygen seems stronger compared to the one between amide hydrogen and phenoxy oxygen. These intramolecular noncovalent interactions form an interesting 6–6–5 pseudoring system and lock the anthranilic anilides into a “bent” conformation (Figure 2A, top view), which appears completely flat when viewed edge-on (Figure 2A, side view). The tendency to form these bifurcated hydrogen bonds is very strong. They remained intact even in **88** with a bulky 8-propargyloxynaphtol group, although this moiety twists out of the molecular plane (Figure 2B). In addition, when cocrystallized with hydrogen bond disruptors such as H_2_O (**70**) or DMSO (**95** and **102**), the intramolecular hydrogen bonds were still preferentially formed (Figure 2B and Table S8).

**Figure 2:**
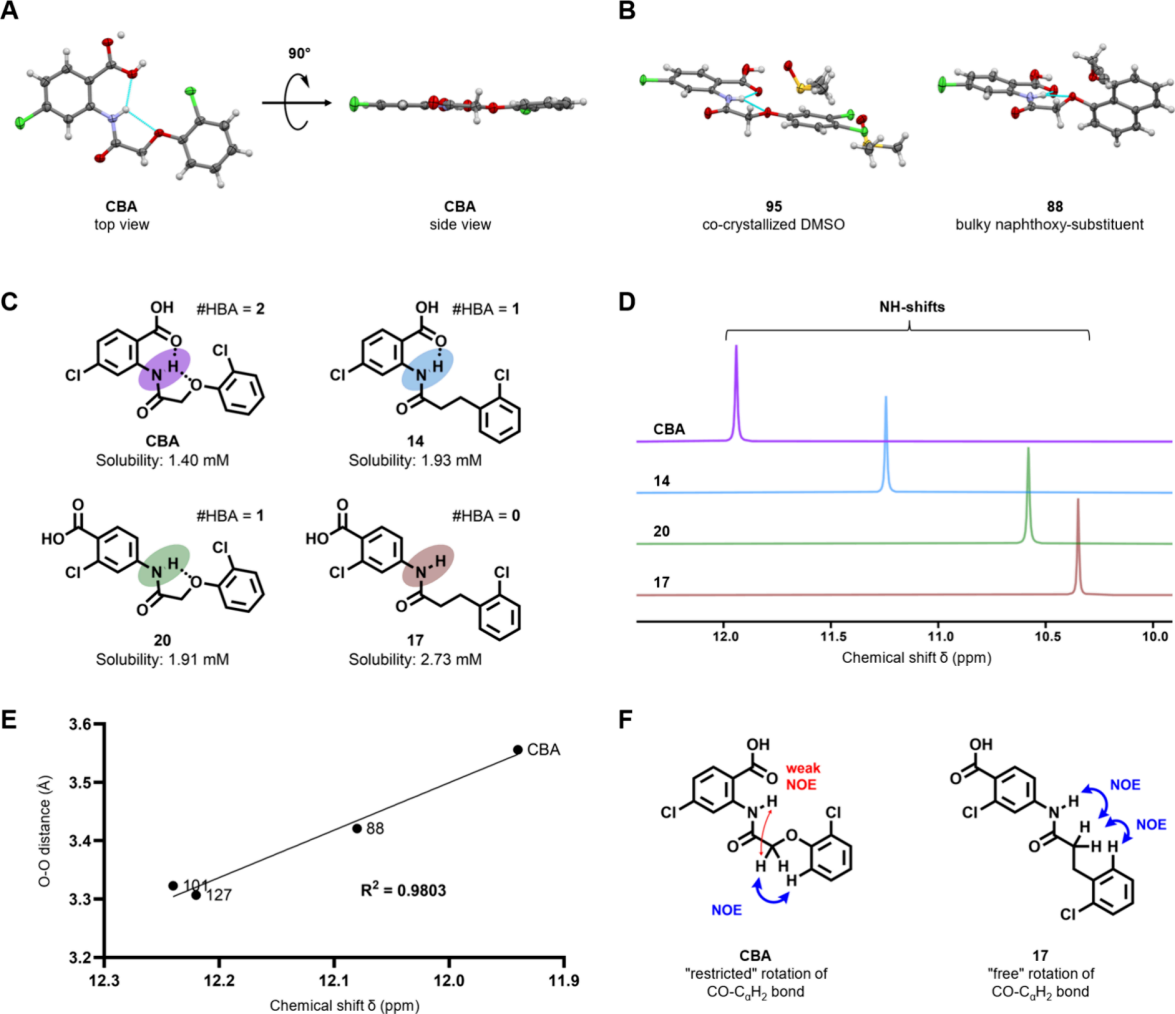
(A) Crystal structure of CBA as top and side view, indicating the completely flat structure as a result of bifurcated intramolecular hydrogen bonds (cyan) between carbonyl oxygen, amide hydrogen, and phenoxy oxygen (O-(N)­H–O). (B) Crystal structure of **88**, indicating that bifurcated intramolecular hydrogen bonding is retained even with bulky substituents (8-propargylnaphthoxy) on the scaffold. Crystal structures of **95**, showing that the flat structure is retained when cocrystallized with known hydrogen bond disrupter solvent DMSO. (C) Compounds with various numbers of hydrogen bond acceptors in the vicinity of the amide hydrogen. CBA with two H-bond acceptors, **14** with a carboxyl-oxygen as the only H-bond acceptor, **20** with phenoxy oxygen as the only H-bond acceptor, and **17** with no H-bond acceptors. (D) ^1^H NMR shifts (ppm) of the amide hydrogen in DMSO-*d*_6_ observed for the compounds CBA, **14**, **20**, and **17** with various numbers of hydrogen bond acceptors. (E) Linear correlation (*R* ^2^ = 0.9803) between O–O distances from single crystal X-ray structures of anthranilic anilides CBA, **101**, **88**, and **127** (without cocrystallized solvent molecules or conformational polymorphism) and their corresponding amide-hydrogen ^1^H NMR shifts in DMSO-*d*_6_. (F) Schematically illustrated ^1^H–^1^H couplings between relevant protons in CBA and **17** from ^1^H/^1^H-NOESY measured in DMSO-*d*_6_.

These structural studies, together with the rather low aqueous solubilities of the anthranilic anilides, led us to believe that these intramolecular hydrogen bonds are also present in solution. We found experimental evidence for our hypothesis by recording unusually large downfield shifts of amide hydrogen ^1^H NMR signals in DMSO-*d*_6_ for our anthranilic anilides (12.59–11.88 ppm), indicating strong deshielding of this proton by proximal H-bond acceptors (Figure S20A). To exclude the possibility of solvent effects, we measured ^1^H NMR spectra of CBA in protic MeOH-*d*_4_, polar aprotic DMSO-*d*_6_, THF-*d*_8_, CDCl_3_, acetone-*d*_6_ and ACN-*d*_3_, and apolar benzene-*d*_6_ and toluene-*d*_8_. Measurements in D_2_O could not be evaluated due to the low solubility of CBA in H_2_O and rapid amide hydrogen exchange rates. All amide hydrogen ^1^H signals in these solvents were located at large downfield chemical shifts between 11.77 and 12.19 ppm (Figure S20B), which supports our view of these unusual chemical shifts being a result of intramolecular hydrogen bonding. To find further evidence for the presence of the two intramolecular hydrogen bonds in solution, we synthesized CBA derivatives **14**, **17**, and **20** (Figure 2C) with varying numbers of intramolecular hydrogen bond acceptors adjacent to the amide hydrogen and measured their ^1^H NMR spectra in DMSO-*d*_6_. The ^1^H signal at 11.94 ppm for the amide hydrogen in CBA shifted upfield to 11.24 ppm (Δ­(ppm) = 0.7) for **14** and to 10.58 ppm (Δ­(ppm) = 1.36 ppm) for **20**, both offering only one adjacent hydrogen bond acceptor (Figure 2D). For **17**, lacking intramolecular hydrogen bond acceptors in close vicinity, this signal shifted further upfield to 10.35 ppm (Δ­(ppm) = 1.59 ppm). A similar trend in amide NH chemical shifts was observed when comparing truncated CBA analogues **2**, **4**, **11**, and **13** (Figure S21). It is suffice to say that all seven compounds **2**, **4**, **11**, **13**, **14**, **17**, and **20** showed no TRPM4 inhibitory activity (Figures S5 and S8). In addition to the hydrogen bond lengths from X-ray, the chemical shift differences of the amide-H signals of the model compounds also suggest that the hydrogen bond between amide-H and carboxyl oxygen is stronger than between amide-H and phenoxy oxygen (Figure 2D). ^1^H/^1^H-NOESY spectra of **17** revealed strong ^1^H–^1^H coupling between the C_α_H_2_-protons and the aromatic *ortho*-hydrogen on the phenyl ring as well as with the amide hydrogen (Figure 2F and Figure S23). For CBA, strong coupling between the C_α_H_2_-protons and the aromatic *ortho*-hydrogen on the phenyl ring was also detected, but only a weak coupling with the amide hydrogen was observed (Figure 2F and Figure S24). This further supports a bent conformation (CBA), rather than an extended conformation (**17**) in solution, due to intramolecular hydrogen bonding. We also plotted the distance between the phenoxy and carboxyl oxygens from our crystal structures of anthranilic anilides against the corresponding chemical shifts of the amide hydrogen signals (Figure 2E and Figure S22), which revealed a linear correlation (*R* ^2^ = 0.9803). Interestingly, CBA having the longest observed O–O distance in the solid state and the largest amide-H upfield shift in ^1^H NMR was significantly more soluble compared to NBA, **101**, and **127**, where O–O distances were much smaller, and amide-H shifted downfield compared to CBA. Additional temperature-dependent ^1^H NMR measurements of CBA and **17** at 20, 40, 60, and 80 °C resulted in gradual upfield shifts after each temperature increase for both compounds due to weakening of intramolecular or intermolecular hydrogen bonding (Figure S20C). However, in contrast to **17** where the amide-H peak remained sharp at higher temperatures, a gradual broadening of the amide-H peak occurred after each temperature increase in CBA, suggesting a dynamic conformational exchange between different intramolecular H-bonded states. It is also worth mentioning that measured aqueous solubilities for **14**, **20**, and **17** gradually increased with decreasing number of hydrogen bond acceptors adjacent to the amide-H (Figure 2C and Figure S17). The solubilities of the two compounds **14** (1.93 mM) and **20** (1.91 mM) having only one hydrogen bond acceptor were ∼1.4-fold higher compared to CBA (1.40 mM) and the solubility for compound **17** (2.73 mM) having no hydrogen bond acceptor was even higher with a ∼2-fold increase compared to CBA. Intriguingly, even though both compounds **14** (TPSA = 66.40 Å^2^) and **17** (TPSA = 66.40 Å^2^) lack a phenoxy oxygen and therefore have a lower calculated TPSA compared to CBA (TPSA = 75.63 Å^2^), their measured aqueous solubilities were higher (Figure S17). This indicates that these bifurcated intramolecular hydrogen bonds mask the polarity of these heteroatoms in solution. Taken together, these structural studies strongly point to a bent and flat conformation of the anthranilic anilides in the solid state and in solution, which partly explains their poor aqueous solubility. It is also interesting to note that the pseudocyclopenta­[*a*]­naphthalene structure, resulting from these intramolecular interactions, bears strong resemblance to polycyclic compounds 9-phenanthrol^20^ and CFTR channel activator MBP-104, which are known weak TRPM4 inhibitors.

## Conclusions

In the course of this focused SAR study, several anthranilic anilide-based TRPM4 inhibitors, exceeding the potency of NBA and CBA, have been developed and valuable SAR trends of the 4-chloro-2-(2-phenoxyacetamido)­benzoic acid scaffold were identified. From our observations, we can summarize that (1) the phenoxy ring is an essential moiety to maintain TRPM4 inhibitory activity, (2) substitutions at this phenoxy ring led to further enhancement of inhibitor activity, and (3) the *meta*-position of the phenyl ring was the ideal site for substitutions. Furthermore, (4) polysubstitutions of the phenoxy ring were not favorable but rather decreased the activity and solubility, (5) compounds with weakly polar or nonpolar substituents generally showed higher activities, and (6) the length, branching, and degree of freedom of apolar substituents seemed to be important factors for activity. Our SAR findings indicate that the phenoxy ring is situated in a well-defined hydrophobic pocket in the TRPM4 binding site. The cryo-EM structures of TRPM4 complexed with NBA or compound **103** (IBA) indeed show that the aryloxy rings of both inhibitors point into a hydrophobic pocket, which is also the case in our docking model for **118** (PBA).^34^ However, to exercise caution, the low resolution of these cryo-EM structures allows only limited direct comparison with SAR outcomes. Measurements of aqueous solubilities, log *D*_7.4_ values, and HPLC retention times demonstrated a lipophilic character for our anthranilic anilides. X-ray crystal structures and NMR studies of anthranilic anilides revealed bifurcated intramolecular hydrogen bonds, leading to pseudopolycyclic conformations masking their polar functional groups. We speculate that this allows the compounds to diffuse through the plasma membrane to reach the TRPM4 binding site. Our study has shown that the 4-chloro-2-(2-phenoxyacetamido)­benzoic acid scaffold is amenable to fine-tuning and our discovered inhibitor **118** (PBA) seems to best balance potent inhibitory activity, decent aqueous solubility, and also sufficient lipophilicity for efficient target site reach and engagement. By breaking up the rigid and aromatic naphthalene structure in NBA and the introduction of a more flexible propargyloxy group in *meta*-position, the aqueous solubility of **118** (PBA) was increased ∼1.7-fold compared to NBA and lipophilicity slightly reduced. **118** (PBA) showed a significant increase in inhibitory potency compared to NBA both in HEK293 cells overexpressing hTRPM4 and in human colorectal cancer (HCT116) cells endogenously expressing hTRPM4, without increasing the size (higher LE) or lipophilicity of the scaffold (higher LLE). **118** (PBA) was not active on hTRPM5 and in contrast to reference compound CBA, **118** (PBA) was also able to block mouse TRPM4, even more potently than NBA. In addition, **118** (PBA) displayed very low cytotoxicity in HEK293 cells and no inhibition of heart ion channels hERG, hNa_V_1.5, and hCa_V_1.2 at relevant concentrations. Taking together, we believe that **118** (PBA) will serve as a valuable chemical probe in TRPM4 research and exploration of potential therapeutic avenues in TRPM4-related pathologies.

## Experimental Section

### General Remarks

Reagents and organic solvents were purchased from commercial suppliers and used without further purification unless otherwise stated. Deionized H_2_O produced in-house or Milli-Q H_2_O (18.2 MΩ*cm at 32 °C) was used depending on the application. Aqueous solutions of NaOH, HCl, saturated NH_4_Cl, and saturated NaCl (brine) were prepared with deionized H_2_O. Thin-layer chromatography (TLC) was performed using Macherey-Nagel ALUGRAM Xtra SIL G/UV_254_ plates coated with 0.20 mm silica gel 60 containing fluorescent indicator. High-performance liquid chromatography (HPLC) was performed using a Thermo Fisher Scientific UltiMate 3000 RSLCnano System composed of a DIONEX UltiMate 3000 Pump, a DIONEX UltiMate 3000 Sampler, a DIONEX UltiMate 3000 Column Compartment, and a DIONEX UltiMate 3000 Diode Array Detector. HPLC measurements were conducted using Milli-Q H_2_O (+0.1% TFA) and ACN (+0.1% TFA) or Milli-Q H_2_O (+0.1% TFA) and THF (+0.1% TFA) as eluents and an Acclaim 120 C18 column (Thermo Scientific). Flash column chromatography was performed using the Teledyne Isco Combi*Flash* *Rf*+ system. Teledyne Isco Redi*Sep* *Rf* dry load cartridges were used for the preparation of dry loads. If not stated otherwise, dry loads were prepared on silica gel. Teledyne Isco Silica Redi*Sep* *Rf* prepacked silica flash columns of various sizes were used for chromatographic purification. Melting points were determined using a Büchi Melting Point M-560. Ultraviolet–visible (UV–vis) spectroscopy was performed using either a DIONEX UltiMate 3000 Diode Array Detector (HPLC system) or an Agilent Technologies Cary 60 UV–vis spectrophotometer. Nuclear magnetic resonance spectroscopy (NMR) was performed at the Department of Chemistry, Biochemistry and Pharmaceutical Sciences (DCBP), Universität Bern, Switzerland (group of Prof. Dr. J. Furrer) using a Bruker AVANCE III HD 300 GA spectrometer with a magnetic field of 7.05 T and operating frequencies of 300.13 MHz for ^1^H measurements and 75.48 MHz for ^13^C measurements. Spectra were normalized to residual internal solvents. High-resolution mass spectrometry (HRMS) was performed by the mass spectrometry service (group of Prof. Dr. S. Schürch) at the DCBP. The measurements were performed using electrospray ionization (ESI) and a ThermoScientific LTQ Orbitrap XL mass spectrometer with high mass resolution (m/Δ*m* > 100,000) and accuracy (Δ*m* < 3 ppm). X-ray crystal structures were determined at the X-ray service unit of the DCBP (group of PD Dr. S. Grabowsky).^41–45^ The fluorescence intensity measurements during the cell-based *in vitro* sodium influx assay were performed using a FLIPR Tetra High-Throughput Cellular Screening System from Molecular Devices.

The compounds NBA, CBA, **14**, **20**, **93**, and **94** portrayed in this study have previously been investigated as TRPM4 inhibitors^23^ and CBA as an inhibitor of TMEM206 at low pH^46,47^ and were freshly resynthesized and tested for this study. The previously published synthetic procedures^23^ to generate anthranilic anilide compounds were slightly adapted. Compound **103** from this SAR study was synthesized for a recently published cryo-EM study of human TRPM4.^34^ The two methyl anthranilate compounds **43** and **130** were reported in a previous study on TRPM4 and resynthesized for this study according to the literature.^32^ The compounds **86**, **90**, **95**, and **116** were reported in a non-TRPM4 related synthetic paper.^38^ Compound **92** was reported in non-TRPM4 related paper.^48^ All final and tested compounds synthesized in this study were >95% pure by HPLC analysis (Table S9).

### General Procedure A for Acetylation Reactions

The respective amine was dissolved in dry THF under an argon atmosphere and stirred for 10 min at rt. K_2_CO_3_ (2 equiv) was then added, and the mixture was cooled down to 0 °C in an ice bath and further stirred for 10 min. The respective acyl chloride (1 equiv) was then added dropwise via syringe, and the mixture was stirred at 0 °C and later slowly warmed up to rt. The reaction was followed by TLC (cyclohexane/EtOAc, 4:1). The volatiles were then removed under reduced pressure upon satisfactory conversion, and H_2_O was added under stirring. The crude product was then extracted with EtOAc, the combined organic phases were washed with brine, dried over MgSO_4_, and filtered through cotton, and the volatiles were evaporated under reduced pressure. The crude product was purified by flash column chromatography (cyclohexane/EtOAc, gradient) and the purified product was dried *in vacuo* and analyzed.

### General Procedure B for the Synthesis of Phenolic/Naphtolic Intermediates

The respective hydroxyphenol or hydroxynaphthol (1 equiv) was dissolved in dry acetone, K_2_CO_3_ (0.55 equiv) was added, and the mixture was stirred at rt. Propargyl bromide (1 equiv, 80 wt % in toluene) or 1-bromobut-2-yne (1.2 equiv) was then slowly added via syringe, and the reaction mixture was heated to 65 °C and monitored by TLC (cyclohexane/EtOAc, 4:1). After satisfactory conversion of the starting material into the product and side products, the reaction mixture was cooled down to rt and the volatiles were evaporated *in vacuo*. H_2_O and aqueous HCl (2 M) were added, and the mixture was stirred at rt for 10 min. The product was then extracted with DCM, the combined organic phases were washed with brine containing HCl (aq), dried over MgSO_4_, filtered through cotton, and the volatiles were evaporated under reduced pressure. The crude product was purified by flash column chromatography (cyclohexane/EtOAc, gradient) and the purified product was dried *in vacuo* and analyzed.

### General Procedure C for the Synthesis of Methyl Ester Intermediates

Methyl 4-chloro-2-(2-chloroacetamido)­benzoate (**7**) was dissolved in dry DMF to give a clear solution and K_2_CO_3_ (2 equiv) was added, resulting in a cloudy suspension. This mixture was stirred for 10 min at rt, and the corresponding substituted phenol (1.1 equiv) was added. The reaction mixture was then heated to 80 °C and monitored by TLC (cyclohexane/EtOAc, 4:1). Upon satisfactory conversion of the starting material, the reaction mixture was cooled to rt and H_2_O was added to the mixture, which was stirred for 10 min. The product was then extracted with EtOAc, the combined organic phases were washed with brine, dried over MgSO_4_, filtered through cotton, and the volatiles were evaporated under reduced pressure. The crude product was purified by flash column chromatography (cyclohexane/EtOAc, gradient), and the purified product was dried *in vacuo* and analyzed.

### General Procedure D for the Synthesis of Anthranilic Anilide Final Products

The corresponding methyl ester compound (see *General Procedure C*) was dissolved in MeOH, and KOH (3 equiv) dissolved in the appropriate amount of H_2_O, to result in a final MeOH/H_2_O ratio of 5:1, was added. This reaction mixture was stirred at 65 °C and monitored by TLC (DCM/MeOH, 9:1). Upon satisfactory conversion of the starting material, the reaction mixture was cooled down to rt and aqueous HCl (1 M) was then added, resulting in an immediate precipitation of the product, which was filtered off using a glass filter frit (Por. 4), subsequently washed with ice cold Milli-Q H_2_O, and dried *in vacuo*. When purities of final products were not satisfactory after precipitation, they were further purified by flash column chromatography (DCM/MeOH, gradient) and/or recrystallization, dried *in vacuo*, and analyzed.

### General Procedure E for the Synthesis of Anthranilic Anilide Final Products (One-Pot Approach)

Methyl 4-chloro-2-(2-chloroacetamido)­benzoate (**7**) was dissolved in dry DMF to give a clear solution and K_2_CO_3_ (2 equiv) was added, resulting in a cloudy suspension. This mixture was stirred for 10 min at rt, and the corresponding substituted phenol (1.1 equiv) was added. The reaction mixture was then heated to 80 °C and monitored by TLC (cyclohexane/EtOAc, 4:1). Upon satisfactory conversion of the starting material, the reaction mixture was cooled to rt, and aqueous HCl (2 M) was added until a clear solution was obtained. The volatiles were then evaporated under reduced pressure, and the crude product was directly used for the next reaction. It was subsequently dissolved in MeOH, and KOH (3 equiv), dissolved in the appropriate amount of H_2_O to give a final MeOH/H_2_O ratio of ca. 5:1, was added. This reaction mixture was stirred at 65 °C and monitored by TLC (DCM/MeOH, 9:1). Upon satisfactory conversion of the starting material, the reaction mixture was cooled down to rt and aqueous HCl (1 M) was then added, resulting in an immediate precipitation of the product, which was filtered off using a glass filter frit (Por. 4), subsequently washed with ice cold Milli-Q H_2_O, and dried *in vacuo*. The precipitate was then dissolved from the filter frit using THF, and a dry load on silica gel was prepared. The crude product was then purified by flash column chromatography (DCM/MeOH, gradient), and the purified product was dried *in vacuo.* When purities of final products were not satisfactory after flash column chromatography, they were further purified by additional flash column chromatography and/or recrystallization, dried *in vacuo*, and analyzed.

#### *N*-(3-Chlorophenyl)­acetamide (**2**)

The reaction was carried out according to general procedure A using 3-chloroaniline (7.84 mmol) dissolved in THF (50 mL), K_2_CO_3_ (15.68 mmol), and acetyl chloride (7.84 mmol). Reaction time: 60 min. Purification: Flash column chromatography (cyclohexane/EtOAc, gradient). Yield: quant. ^1^H NMR (300 MHz, DMSO-*d*_6_): δ 10.11 (s, 1H), 7.80 (t, *J* = 2.0 Hz, 1H), 7.41 (ddd, *J* = 8.2, 1.9, 1.0 Hz, 1H), 7.31 (t, *J* = 8.0 Hz, 1H), 7.07 (ddd, *J* = 7.9, 2.1, 1.0 Hz, 1H), 2.05 (s, 3H). ^13^C NMR (75 MHz, DMSO-*d*_6_): δ 168.7, 140.7, 133.0, 130.4, 122.7, 118.4, 117.3, 24.0. HRMS (ESI) *m*/*z*: [M + H]^+^ calculated for C_8_H_9_ONCl 170.0367, found 170.0371. Purity by HPLC: peak area >99% (detection at 254 nm).

#### 2-Chloro-*N*-(3-chlorophenyl)­acetamide (**3**)

The reaction was carried out according to general procedure A using 3-chloroaniline (7.84 mmol) dissolved in THF (50 mL), K_2_CO_3_ (15.68 mmol), and chloroacetyl chloride (7.84 mmol). Reaction time: 20 min at 0 °C and 19 h at rt. Purification: Flash column chromatography (cyclohexane/EtOAc, gradient). Yield: quant. ^1^H NMR (300 MHz, DMSO-*d*_6_): δ 10.47 (s, 1H), 7.79 (t, *J* = 2.0 Hz, 1H), 7.45 (ddd, *J* = 8.2, 1.9, 1.1 Hz, 1H), 7.36 (t, *J* = 8.0 Hz, 1H), 7.15 (ddd, *J* = 7.9, 2.1, 1.1 Hz, 1H), 4.27 (s, 2H). ^13^C NMR (75 MHz, DMSO-*d*_6_): δ 165.0, 139.9, 133.1, 130.6, 123.6, 118.8, 117.8, 43.5. HRMS (ESI) *m*/*z*: [M + H]^+^ calculated for C_8_H_8_ONCl_2_ 203.9977, found 203.9976. Purity by HPLC: peak area >99% (detection at 254 nm).

#### 2-(2-Chlorophenoxy)-*N*-(3-chlorophenyl)­acetamide (**4**)

The reaction was carried out according to general procedure C using **3** (1.58 mmol) dissolved in DMF (10 mL), K_2_CO_3_ (3.16 mmol), and 2-chlorophenol (1.74 mmol). Reaction time: 21 h. Purification: Flash column chromatography (cyclohexane/EtOAc, gradient). Yield: 34%. ^1^H NMR (300 MHz, DMSO-*d*_6_): δ 10.35 (s, 1H), 7.82 (t, *J* = 2.0 Hz, 1H), 7.47 (td, *J* = 7.8, 1.3 Hz, 2H), 7.36 (t, *J* = 8.1 Hz, 1H), 7.29 (ddd, *J* = 8.4, 7.5, 1.6 Hz, 1H), 7.14 (ddd, *J* = 7.9, 2.1, 1.0 Hz, 1H), 7.08 (dd, *J* = 8.3, 1.3 Hz, 1H), 6.99 (td, *J* = 7.7, 1.4 Hz, 1H), 4.85 (s, 2H). ^13^C NMR (75 MHz, DMSO-*d*_6_): δ 166.4, 153.4, 139.8, 133.1, 130.5, 130.1, 128.3, 123.4, 122.2, 121.4, 118.9, 117.8, 114.1, 67.6. HRMS (ESI) *m*/*z*: [M + H]^+^ calculated for C_14_H_12_O_2_NCl_2_ 296.0240, found 296.0243. Purity by HPLC: peak area >99% (detection at 254 nm).

#### Methyl 2-Acetamido-4-chlorobenzoate (**6**)

The reaction was carried out according to general procedure A using methyl 2-amino-4-chlorobenzoate **5** (5.39 mmol) dissolved in THF (50 mL), K_2_CO_3_ (10.79 mmol), and acetyl chloride (5.39 mmol). Reaction time: 16.5 h. Purification: Flash column chromatography (cyclohexane/EtOAc, gradient). Yield: 87%. X-ray: Single crystals were obtained from cyclohexane/EtOAc. ^1^H NMR (300 MHz, DMSO-*d*_6_): δ 10.65 (s, 1H), 8.40 (d, *J* = 2.2 Hz, 1H), 7.91 (d, *J* = 8.6 Hz, 1H), 7.24 (dd, *J* = 8.6, 2.2 Hz, 1H), 3.86 (s, 3H), 2.15 (s, 3H). ^13^C NMR (75 MHz, DMSO-*d*_6_): δ 168.9, 166.9, 140.9, 138.4, 132.2, 122.9, 120.0, 115.7, 52.6, 24.7. HRMS (ESI) *m*/*z*: [M + H]^+^ calculated for C_10_H_11_O_3_NCl 228.0422, found 228.0418.

#### Methyl 4-Chloro-2-(2-chloroacetamido)­benzoate (**7**)

The reaction was carried out according to general procedure A using methyl 2-amino-4-chlorobenzoate **5** (39.22 mmol) dissolved in THF (150 mL), K_2_CO_3_ (78.73 mmol), and chloroacetyl chloride (39.30 mmol). Reaction time: 16 h. Purification: Flash column chromatography (eluent: cyclohexane/EtOAc, gradient). The purified product (white powder) was dried *in vacuo* and recrystallized from *n*-heptane. Yield: 94%. X-ray: Single crystals were obtained from *n*-heptane. ^1^H NMR (300 MHz, DMSO-*d*_6_): δ 11.43 (s, 1H), 8.52 (d, *J* = 2.1 Hz, 1H), 7.99 (d, *J* = 8.6 Hz, 1H), 7.32 (dd, *J* = 8.6, 2.2 Hz, 1H), 4.48 (s, 2H), 3.89 (s, 3H). ^13^C NMR (75 MHz, DMSO-*d*_6_): δ 166.7, 165.7, 140.3, 138.7, 132.5, 123.8, 119.8, 115.6, 52.8, 43.3. HRMS (ESI) *m*/*z*: [M + H]^+^ calculated for C_10_H_9_Cl_2_NO_3_ 262.0032, found 262.0037. Purity by HPLC: peak area >99% (detection at 254 nm).

#### Methyl 4-Chloro-2-(3-(2-chlorophenyl)­propanamido)­benzoate (**8**)

The reaction was carried out according to general procedure A using methyl 2-amino-4-chlorobenzoate **5** (1.42 mmol) dissolved in THF (10 mL), K_2_CO_3_ (2.84 mmol), and 3-(2-chlorophenyl)­propanoyl chloride **10** (2.13 mmol). Reaction time: 120 min. Purification: Flash column chromatography (eluent: cyclohexane/EtOAc, gradient). Yield: 70%. ^1^H NMR (300 MHz, DMSO-*d*_6_): δ 10.68 (s, 1H), 8.39 (d, *J* = 2.2 Hz, 1H), 7.91 (d, *J* = 8.6 Hz, 1H), 7.41 (ddd, *J* = 11.7, 6.9, 1.8 Hz, 2H), 7.32–7.19 (m, 3H), 3.z84 (s, 3H), 3.04 (t, 2H), 2.74 (t, 2H). ^13^C NMR (75 MHz, DMSO-*d*_6_): δ 170.5, 166.8, 140.7, 138.4, 137.8, 132.9, 132.3, 130.6, 129.2, 128.2, 127.3, 123.0, 120.2, 116.0, 52.6, 36.7, 28.3. HRMS (ESI) *m*/*z*: [M + H]^+^ calculated for C_17_H_16_O_3_NCl_2_ 352.0502, found 352.0502.

#### 3-(2-Chlorophenyl)­propanoyl Chloride (**10**)

Commercially available 3-(2-chlorophenyl)­propanoic acid **9** (2.27 mmol) was dissolved in dry DCM (20 mL) under argon. Ten drops of dry DMF were then added via a syringe, and the mixture was cooled down to 0 °C in an ice bath. Thionyl chloride (22.71 mmol) was then slowly added via a syringe under stirring. The mixture was subsequently warmed up to rt, then heated up to 40 °C (55 °C on heat plate), and refluxed over the night. The reaction was followed by TLC (DCM/MeOH, 9:1), and after 17 h, the mixture was cooled down to rt and the volatiles were removed under reduced pressure under air- and moisture-free conditions. The colorless liquid product was not analyzed and directly used for further reactions.

#### 2-Acetamido-4-chlorobenzoic Acid (**11**)

The reaction was carried out according to general procedure D using **6** (4.62 mmol) dissolved in MeOH (50 mL) and KOH (13.85 mmol) dissolved in H_2_O (10 mL). Reaction time: 10 min. Yield: 93%. ^1^H NMR (300 MHz, DMSO-*d*_6_): δ 13.84 (s, 1H), 11.15 (s, 1H), 8.58 (d, *J* = 2.1 Hz, 1H), 7.97 (d, *J* = 8.6 Hz, 1H), 7.20 (dd, *J* = 8.6, 2.2 Hz, 1H), 2.15 (s, 3H). ^13^C NMR (75 MHz, DMSO-*d*_6_): δ168.9, 168.8, 141.9, 138.4, 132.8, 122.4, 119.1, 114.9, 25.0. HRMS (ESI) *m*/*z*: [M – H]^−^ calculated for C_9_H_7_O_3_NCl 212.0120, found 212.0120. Purity by HPLC: peak area >99% (detection at 254 nm).

#### 4-Chloro-2-(2-chloroacetamido)­benzoic Acid (**12**)

The reaction was carried out according to general procedure D using **7** (1.16 mmol) dissolved in MeOH (50 mL) and KOH (3.47 mmol) dissolved in H_2_O (15 mL). Reaction time: 30 min. Flash column chromatography (cyclohexane/EtOAc (85%), MeOH (10%), AcOH (5%)). Yield: 35%. ^1^H NMR (300 MHz, DMSO-*d*_6_): δ 14.00 (s, 1H), 11.92 (s, 1H), 8.62 (d, *J* = 2.1 Hz, 1H), 8.02 (d, *J* = 8.6 Hz, 1H), 7.29 (dd, *J* = 8.6, 2.2 Hz, 1H), 4.48 (s, 2H). ^13^C NMR (75 MHz, DMSO-*d*_6_): δ 168.6, 165.7, 141.0, 138.5, 132.9, 123.4, 119.1, 115.5, 43.4. HRMS (ESI) *m*/*z*: [M – H]^−^ calculated for C_9_H_6_O_3_NCl_2_ 245.9730, found 245.9730. Purity by HPLC: peak area >99% (detection at 254 nm).

#### 4-Chloro-2-(2-methoxyacetamido)­benzoic Acid (**13**)

The reaction was carried out according to general procedure D using **7** (1.43 mmol) dissolved in MeOH (30 mL) and KOH (5.70 mmol) dissolved in H_2_O (6 mL). Reaction time: 90 min. Flash column chromatography (cyclohexane/EtOAc (85%), MeOH (10%), AcOH (5%)). Yield: 17%. ^1^H NMR (300 MHz, DMSO-*d*_6_): δ 13.87 (s, 1H), 11.93 (s, 1H), 8.75 (d, *J* = 2.1 Hz, 1H), 8.01 (d, *J* = 8.6 Hz, 1H), 7.25 (dd, *J* = 8.6, 2.2 Hz, 1H), 4.05 (s, 2H), 3.43 (s, 3H). ^13^C NMR (75 MHz, DMSO-*d*_6_): δ 169.3, 168.5, 141.2, 138.5, 132.9, 122.8, 118.9, 115.0, 71.7, 59.1. HRMS (ESI) *m*/*z*: [M – H]^−^ calculated for C_10_H_9_O_4_NCl 242.0226, found 242.0224. Purity by HPLC: peak area >98% (detection at 254 nm).

#### 4-Chloro-2-(3-(2-chlorophenyl)­propanamido)­benzoic Acid (**14**)

The reaction was carried out according to general procedure D using **8** (0.95 mmol) dissolved in MeOH (50 mL) and KOH (2.85 mmol) dissolved in H_2_O (10 mL). Reaction time: 10 min. Yield: 97%. ^1^H NMR (300 MHz, DMSO-*d*_6_): δ 13.87 (s, 1H), 11.24 (s, 1H), 8.59 (d, *J* = 2.2 Hz, 1H), 7.97 (d, *J* = 8.6 Hz, 1H), 7.46–7.35 (m, 2H), 7.31–7.17 (m, 3H), 3.05 (t, *J* = 7.7 Hz, 2H), 2.74 (t, 2H). ^13^C NMR (75 MHz, DMSO-*d*_6_): δ 170.5, 168.8, 141.7, 138.5, 137.8, 132.9, 132.8, 130.6, 129.3, 128.2, 127.4, 122.5, 119.2, 115.0, 37.1, 28.4. HRMS: (ESI) *m*/*z*: [M – H]^−^ calculated for C_16_H_12_O_3_NCl_2_ 336.0200, found 336.0190. Purity by HPLC: peak area >99% (detection at 254 nm).

#### Methyl 2-Chloro-4-(3-(2-chlorophenyl)­propanamido)­benzoate (**16**)

The reaction was carried out according to general procedure A using commercially available 4-amino-2-chlorobenzoic acid **15** (1.53 mmol) dissolved in THF (20 mL), K_2_CO_3_ (4.13 mmol) and **10** (2.30 mmol). Purification: Flash column chromatography (cyclohexane/EtOAc, gradient). Yield: 93%. ^1^H NMR (300 MHz, DMSO-*d*_6_): δ 10.39 (s, 1H), 7.91 (d, *J* = 2.0 Hz, 1H), 7.84 (d, *J* = 8.6 Hz, 1H), 7.55 (dd, *J* = 8.6, 2.1 Hz, 1H), 7.46–7.40 (m, 1H), 7.40–7.33 (m, 1H), 7.32–7.19 (m, 2H), 3.82 (s, 3H), 3.02 (t, *J* = 7.6 Hz, 2H), 2.69 (t, *J* = 7.6 Hz, 2H). ^13^C NMR (75 MHz, DMSO-*d*_6_): δ 170.9, 164.7, 143.2, 138.1, 133.1, 132.9, 132.5, 130.6, 129.3, 128.1, 127.3, 123.1, 120.1, 117.0, 52.2, 36.0, 28.2. HRMS (ESI) *m*/*z*: [M + H]^+^ calculated for C_17_H_16_O_3_NCl_2_ 352.0502, found 352.0501.

#### 2-Chloro-4-(3-(2-chlorophenyl)­propanamido)­benzoic acid (**17**)

The reaction was carried out according to general procedure D using **16** (1.04 mmol) dissolved in MeOH (30 mL) and KOH (3.13 mmol) dissolved in H_2_O (6 mL). Reaction time: 120 min. Yield: 96%. ^1^H NMR (300 MHz, DMSO-*d*_6_): δ 13.08 (s, 1H), 10.35 (s, 1H), 7.88 (d, *J* = 2.0 Hz, 1H), 7.82 (d, *J* = 8.6 Hz, 1H), 7.52 (dd, *J* = 8.6, 2.1 Hz, 1H), 7.43 (dd, *J* = 7.4, 1.9 Hz, 1H), 7.40–7.33 (m, 1H), 7.32–7.19 (m, 2H), 3.02 (t, *J* = 7.6 Hz, 2H), 2.69 (t, *J* = 7.7 Hz, 2H). ^13^C NMR (75 MHz, DMSO-*d*_6_): δ 170.8, 165.9, 142.7, 138.2, 133.1, 132.9, 132.4, 130.6, 129.3, 128.1, 127.3, 124.3, 120.1, 116.9, 36.0, 28.3. HRMS (ESI) *m*/*z*: [M – H]^−^ calculated for C_16_H_12_O_3_NCl_2_ 336.0200, found 336.0194. Purity by HPLC: peak area >99% (detection at 254 nm).

#### Methyl 2-Chloro-4-(2-chloroacetamido)­benzoate (**18**)

The compound was synthesized in a previous study in our lab. The procedure was performed according to the literature.^23^

#### Methyl 2-Chloro-4-(2-(2-chlorophenoxy)­acetamido)­benzoate (**19**)

The compound was synthesized in a previous study in our lab. The procedure was performed according to the literature.^23^

#### 2-Chloro-4-(2-(2-chlorophenoxy)­acetamido)­benzoic Acid (**20**)

The compound was synthesized in a previous study in our lab. The procedure was performed according to the literature.^23^ Chemical analysis of the compound was repeated to ensure identity, which corresponded to those in the literature.^23^ Purity by HPLC: peak area >99% (detection at 254 nm).

#### 2-(Prop-2-yn-1-yloxy)­phenol (**24**)

The reaction was carried out according to general procedure B using commercially available pyrocatechol **21** (30.35 mmol), acetone (150 mL), propargyl bromide (30.35 mmol, 80 wt % in toluene), and K_2_CO_3_ (16.55 mmol). Reaction time: 16 h. Purification: Flash column chromatography (cyclohexane/EtOAc, gradient). Yield: 26%. ^1^H NMR (300 MHz, DMSO-*d*_6_): δ 9.07 (s, 1H), 6.98 (dt, *J* = 7.7, 1.1 Hz, 1H), 6.84–6.78 (m, 2H), 6.78–6.68 (m, 1H), 4.75 (d, *J* = 2.4 Hz, 2H), 3.53 (t, *J* = 2.4 Hz, 1H). ^13^C NMR (75 MHz, DMSO-*d*_6_): δ 147.2, 145.4, 122.1, 119.0, 116.1, 115.1, 79.6, 78.1, 56.1. HRMS (ESI) *m*/*z*: [M – H]^−^ calculated for C_9_H_7_O_2_ 147.0452, found 147.0455.

#### 3-(Prop-2-yn-1-yloxy)­phenol (**25**)

The reaction was carried out according to general procedure B using resorcinol **22** (30.36 mmol), acetone (150 mL), K_2_CO_3_ (16.52 mmol), and propargyl bromide (30.36 mmol, 80 wt % in toluene). Reaction time: 16 h. Purification: Flash column chromatography (cyclohexane/EtOAc, gradient). Yield: 32%. ^1^H NMR (300 MHz, DMSO-*d*_6_) δ 9.45 (s, 1H), 7.11–7.01 (m, 1H), 6.44–6.34 (m, 3H), 4.70 (d, *J* = 2.4 Hz, 2H), 3.54 (t, *J* = 2.4 Hz, 1H). ^13^C NMR (75 MHz, DMSO-*d*_6_) δ 158.5, 158.5, 129.9, 108.5, 105.4, 102.1, 79.5, 78.1, 55.2.

#### 4-(Prop-2-yn-1-yloxy)­phenol (**26**)

The reaction was carried out according to general procedure B using hydroquinone **23** (30.23 mmol), acetone (150 mL), K_2_CO_3_ (16.57 mmol), and propargyl bromide (30.23 mmol, 80 wt % in toluene). Reaction time: 16 h. Purification: Flash column chromatography (cyclohexane/EtOAc, gradient). Yield: 32%. ^1^H NMR (300 MHz, DMSO-*d*_6_) δ 8.98 (s, 1H), 6.85–6.75 (m, 2H), 6.73–6.63 (m, 2H), 4.65 (d, *J* = 2.4 Hz, 2H), 3.50 (t, *J* = 2.4 Hz, 1H). ^13^C NMR (75 MHz, DMSO-*d*_6_) δ 151.8, 150.0, 116.0, 115.6, 79.7, 77.8, 56.0. HRMS (ESI) *m*/*z*: [M – H]^−^ calculated for C_9_H_7_O_2_ 147.0452, found 147.0450.

#### 3-(But-2-yn-1-yloxy)­phenol (**27**)

The reaction was carried out according to general procedure B using resorcinol **22** (8.37 mmol), acetone (10 mL), K_2_CO_3_ (4.60 mmol), and 1-bromobut-2-yne (10.04 mmol, 80 wt % in toluene). Reaction time: 6 h. Purification: Flash column chromatography (cyclohexane/EtOAc, gradient). Yield: 40%. ^1^H NMR (300 MHz, DMSO-*d*_6_): δ 9.41 (s, 1H), 7.08–7.01 (m, 1H), 6.43–6.30 (m, 3H), 4.64 (q, *J* = 2.3 Hz, 2H), 1.83 (t, *J* = 2.3 Hz, 3H). HRMS (ESI) *m*/*z*: [M – H]^−^ calculated for C_10_H_9_O_2_ 161.0608, found 161.0605.

#### 2-Methyl-3-(prop-2-yn-1-yloxy)­phenol (**29**)

The reaction was carried out according to general procedure B using 2-methylbenzene-1,3-diol (24.38 mmol), acetone (150 mL), K_2_CO_3_ (13.41 mmol), and propargyl bromide (24.38 mmol, 80 wt % in toluene). Reaction time: 16.5 h. Purification: Flash column chromatography (cyclohexane/EtOAc, gradient). Yield: 29%. ^1^H NMR (300 MHz, DMSO-*d*_6_): δ 9.27 (s, 1H), 6.92 (t, *J* = 8.2 Hz, 1H), 6.47 (dd, *J* = 8.2, 3.2 Hz, 2H), 4.73 (d, *J* = 2.4 Hz, 2H), 3.52 (t, *J* = 2.4 Hz, 1H). ^13^C NMR (75 MHz, DMSO-*d*_6_): δ 156.3, 156.0, 125.9, 112.1, 108.5, 103.3, 79.7, 77.8, 55.8, 8.4. HRMS (ESI) *m*/*z*: [M – H]^−^ calculated for C_10_H_9_O_2_ 161.0608, found 161.0611.

#### 5-(Prop-2-yn-1-yloxy)­naphthalen-1-ol (**32**)

The reaction was carried out according to general procedure B using 1,5-dihydroxynaphtalene **30** (19.36 mmol), acetone (125 mL), K_2_CO_3_ (10.66 mmol), and propargyl bromide (19.36 mmol, 80 wt % in toluene). Reaction time: 22.5 h. Purification: Flash column chromatography (cyclohexane/EtOAc, gradient). Yield: 25%. ^1^H NMR (300 MHz, DMSO-*d*_6_): δ 10.09 (s, 1H), 7.75 (dt, *J* = 8.5, 0.9 Hz, 1H), 7.57 (dt, *J* = 8.5, 1.0 Hz, 1H), 7.32 (ddd, *J* = 18.3, 8.5, 7.6 Hz, 2H), 7.02 (dd, *J* = 7.7, 0.9 Hz, 1H), 6.90 (dd, *J* = 7.5, 1.1 Hz, 1H), 4.98 (d, *J* = 2.4 Hz, 2H), 3.61 (t, *J* = 2.3 Hz, 1H). ^13^C NMR (75 MHz, DMSO-*d*_6_): δ 153.1, 152.5, 126.3, 125.9, 125.6, 124.3, 114.9, 111.9, 108.8, 106.2, 79.3, 78.3, 55.8. HRMS (ESI) *m*/*z*: [M + H]^+^ calculated for C_13_H_11_O_2_ 199.0754, found 199.0754.

#### 8-(Prop-2-yn-1-yloxy)­naphthalen-1-ol (**33**)

The reaction was carried out according to general procedure B using 1,8-dihydroxynaphtalene **31** (6.24 mmol), acetone (100 mL), K_2_CO_3_ (3.61 mmol), and propargyl bromide (12.49 mmol). Reaction time: 19 h. Purification: Flash column chromatography (cyclohexane/EtOAc, gradient). Yield: 65%. ^1^H NMR (300 MHz, DMSO-*d*_6_): δ 9.29 (s, 1H), 7.48 (dd, *J* = 8.3, 1.0 Hz, 1H), 7.42–7.29 (m, 3H), 7.04 (dd, *J* = 7.6, 0.8 Hz, 1H), 6.81 (dd, *J* = 5.4, 3.4 Hz, 1H), 5.11 (d, *J* = 2.4 Hz, 2H), 3.70 (t, *J* = 2.4 Hz, 1H). ^13^C NMR (75 MHz, DMSO-*d*_6_): δ 153.7, 153.6, 136.5, 127.5, 125.9, 121.7, 118.7, 114.9, 110.3, 106.6, 79.2, 78.6, 56.7. HRMS (ESI) *m*/*z*: [M – H]^−^ calculated for C_13_H_9_O_2_ 197.0608, found 197.0606.

#### 1-Isobutoxy-3-methoxybenzene (**35**)

3-Methoxyphenol **34** (17.38 mmol) was dissolved in anhydrous DMF (30 mL), K_2_CO_3_ (34.77 mmol) was added, and the suspension was stirred at rt for 10 min. 1-Bromo-2-methylpropane (26.08 mmol) was then added, and the mixture was heated to 80 °C and stirred. The reaction was monitored with TLC (DCM/MeOH, 9:1 and cyclohexane/EtOAc, 4:1) and upon satisfactory conversion of the starting material after 22 h, H_2_O was added to the mixture, which was further stirred for a few minutes. The product was then extracted from the aqueous phase with EtOAc, the combined organic phases were washed with brine, dried over MgSO4, and filtered through cotton, and the volatiles were evaporated under reduced pressure. The crude product was then purified using flash column chromatography (cyclohexane/EtOAc, gradient), and the purified liquid product was dried *in vacuo* in thaw and freeze cycles using liquid nitrogen. Yield: 65%. ^1^H NMR (300 MHz, DMSO-*d*_6_): δ 7.15 (t, *J* = 8.1 Hz, 1H), 6.58–6.38 (m, 3H), 3.75–3.67 (m, 5H), 1.99 (hept, *J* = 6.7 Hz, 1H), 0.97 (d, *J* = 6.7 Hz, 6H). ^13^C NMR (75 MHz, DMSO-*d*_6_): δ 160.5, 160.0, 129.9, 106.6, 106.2, 100.6, 73.7, 55.0, 27.7, 19.1. GCMS (EI) *m*/*z*: [M] calculated for C_11_H_16_O_2_ 180.1145, found 180.1147.

#### 3-Isobutoxyphenol (**36**)

1-Isobutoxy-3-methoxybenzene **35** (11.23 mmol) was dissolved in anhydrous DCM (100 mL) under argon, and the solution was cooled down to 0 °C using an ice bath. Boron tribromide (22.46 mmol, 1 M in DCM) was then slowly added dropwise via a syringe to the solution at 0 °C under stirring. The reaction was then monitored with TLC (cyclohexane/EtOAc, 4:1), and upon satisfactory conversion of the starting material after 4 h, ice cold H_2_O was added to the mixture, which was further stirred for a few minutes. The product was then extracted from the aqueous phase with EtOAc, the combined organic phases were washed with brine, dried over MgSO_4_, filtered through cotton, and the volatiles were evaporated under reduced pressure. The crude product was then purified using flash column chromatography (cyclohexane/EtOAc, gradient), and the purified liquid product was dried *in vacuo* in thaw and freeze cycles using liquid nitrogen. Yield: 11%. ^1^H NMR (300 MHz, DMSO-*d*_6_): δ 9.36 (s, 1H), 7.04 (t, *J* = 8.2 Hz, 1H), 6.42–6.25 (m, 3H), 3.68 (s, 3H).

#### 3-Methoxy-*N*-methylbenzamide (**38**)

3-Methoxybenzoyl chloride **37** (2.93 mmol) was dissolved in diethyl ether (15 mL), and K_2_CO_3_ (5.93 mmol) was added. Methylamine hydrochloride salt (6.01 mmol) was then added as a solid, and the reaction flask was put under argon gas. The reaction mixture was monitored by TLC (DCM/MeOH, 9:1 and cyclohexane/EtOAc, 4:1) and stirred at 21 °C for 22 h. As no full conversion of the starting material was observed, a second equivalent of methylamine hydrochloride salt and 18-crown-6 (2.93 mmol) was added and the reaction mixture was further stirred at 21 °C for 96 h, after which full conversion of the starting material was observed. The volatiles were evaporated *in vacuo* leaving a yellow crude. H_2_O was added, and the suspension was sonicated for 5 min. The product was then extracted from the aqueous phase with DCM, the combined organic phases were washed with brine, dried over MgSO_4_, filtered through cotton, and the volatiles were evaporated under reduced pressure. The crude product was purified with flash column chromatography (DCM/MeOH, gradient), and the purified yellow solid was dried *in vacuo*. Yield: 86%. ^1^H NMR (300 MHz, DMSO-*d*_6_): δ 8.40 (q, *J* = 4.0 Hz, 1H), 7.43–7.32 (m, 3H), 7.10–7.04 (m, 1H), 3.79 (s, 3H), 2.77 (d, *J* = 4.6 Hz, 3H). ^13^C NMR (75 MHz, DMSO-*d*_6_): δ 166.3, 159.1, 136.0, 129.4, 119.2, 116.8, 112.2, 55.2, 26.2. HRMS (ESI) *m*/*z*: [M + H]^+^ calculated for C_9_H_12_O_2_N 166.0863, found 166.0867.

#### 3-Hydroxy-*N*-methylbenzamide (**39**)

3-Methoxy-*N*-methylbenzamide **38** (0.95 mmol) was dried *in vacuo*, put under an argon atmosphere, dissolved in anhydrous DCM (6 mL), and stirred for 10 min at 21 °C. The solution was then cooled down to 0 °C in an ice bath, and boron tribromide (3.80 mmol, 1 M in DCM) was then added dropwise via a syringe for 10 min under stirring. The reaction was followed by TLC (DCM/EtOAc, 9:1) and stirred at 0 °C for 4.5 h until full conversion of the starting material was observed. The reaction mixture was then quenched with cold H_2_O and stirred for a few minutes. The aqueous phase was then extracted first with DCM and later with EtOAc (better for extraction), the combined organic phases were washed with brine, dried over MgSO_4_, and filtered through cotton, and the volatiles were evaporated under reduced pressure. The crude product was purified by flash column chromatography (DCM/MeOH, gradient), and the purified product was dried *in vacuo*. Yield: 96%. ^1^H NMR (300 MHz, DMSO-*d*_6_): δ 9.60 (s, 1H), 8.30 (q, *J* = 4.6 Hz, 1H), 7.27–7.17 (m, 3H), 6.95–6.83 (m, 1H), 2.75 (d, *J* = 4.6 Hz, 3H). ^13^C NMR (75 MHz, DMSO-*d*_6_): δ 166.7, 157.3, 136.0, 129.2, 117.9, 117.5, 114.1, 26.2. HRMS (ESI) *m*/*z*: [M + H]^+^ calculated for C_8_H_10_O_2_N 152.0706, found 152.0706.

#### Methyl 4-Chloro-2-(2-phenoxyacetamido)­benzoate (**41**)

The reaction was carried out according to general procedure C using **7** (0.403 mmol) dissolved in DMF (1 mL), phenol (0.45 mmol), and K_2_CO_3_ (0.85 mmol). Reaction time: 120 min. Yield (crude): 53%. HRMS (ESI) *m*/*z*: [M + H]^+^ calculated for C_16_H_14_ClNO_4_ 320.0684, found 320.0685.

#### 4-Chloro-2-(2-phenoxyacetamido)­benzoic Acid (**86**)

The reaction was carried out according to general procedure D using **41** (0.16 mmol) dissolved in MeOH (9 mL) and KOH (0.52 mmol) dissolved in H_2_O (2 mL). Reaction time: 60 min. Yield: 79%. ^1^H NMR (300 MHz, DMSO-*d*_6_): δ 14.03 (s, 1H), 12.26 (s, 1H), 8.79 (d, *J* = 2.2 Hz, 1H), 8.02 (d, *J* = 8.5 Hz, 1H), 7.35 (t, *J* = 7.9 Hz, 2H), 7.26 (dd, *J* = 8.6, 2.3 Hz, 1H), 7.09 (d, *J* = 8.0 Hz, 2H), 7.01 (t, *J* = 7.3 Hz, 1H), 4.75 (s, 2H). ^13^C NMR (75 MHz, DMSO-*d*_6_): δ 168.7, 167.7, 157.0, 141.2, 138.7, 133.0, 129.7, 123.0, 121.7, 118.8, 114.9, 114.8, 67.1. HRMS (ESI) *m*/*z*: [M + H]^+^ calculated for C_15_H_12_ClNO_4_ 306.0528, found 306.0501. Purity by HPLC: peak area >96% (detection at 254 nm).

#### Methyl 4-Chloro-2-(2-(2-chlorophenoxy)­acetamido)­benzoate (**42**)

The reaction was carried out according to general procedure C using **7** (1.94 mmol) dissolved in DMF (1 mL), 2-chlorophenol (2.12 mmol), and K_2_CO_3_ (3.82 mmol). Reaction time: 1 h. Purification: flash column chromatography (cyclohexane/EtOAc, gradient). Yield: 75%. X-ray: Single crystals were obtained from cyclohexane/ethyl acetate. ^1^H NMR (300 MHz, DMSO-*d*_6_): δ 11.49 (s, 1H), 8.66 (d, *J* = 2.2 Hz, 1H), 7.98 (d, *J* = 8.6 Hz, 1H), 7.49 (dd, *J* = 7.9, 1.6 Hz, 1H), 7.37–7.25 (m, 2H), 7.21 (dd, *J* = 8.3, 1.5 Hz, 1H), 7.04 (td, *J* = 7.6, 1.5 Hz, 1H), 4.88 (s, 2H), 3.84 (s, 3H). ^13^C NMR (75 MHz, DMSO-*d*_6_): δ 167.2, 166.5, 152.6, 140.3, 138.7, 132.4, 130.2, 128.3, 123.5, 122.9, 121.9, 119.9, 115.1, 114.7, 68.2, 52.6. HRMS (ESI) *m*/*z*: [M + H]^+^ calculated for C_16_H_14_O_4_NCl_2_ 354.0294, found 354.0296. Purity by HPLC: peak area >99% (detection at 254 nm).

#### 4-Chloro-2-(2-(2-chlorophenoxy)­acetamido)­benzoic Acid (**CBA**)

The reaction was carried out according to general procedure D using **42** (0.37 mmol) dissolved in MeOH (50 mL) and KOH (0.74 mmol) dissolved in H_2_O (10 mL). Reaction time: 1.5 h. Yield: 94%. X-ray: Single crystals were obtained from *n*-hexane/acetone. ^1^H NMR (300 MHz, DMSO-*d*_6_): δ 13.92 (s, 1H), 11.94 (s, 1H), 8.76 (d, *J* = 2.1 Hz, 1H), 8.01 (d, *J* = 8.6 Hz, 1H), 7.48 (dd, *J* = 7.9, 1.6 Hz, 1H), 7.35–7.25 (m, 2H), 7.18 (dd, *J* = 8.3, 1.3 Hz, 1H), 7.03 (td, *J* = 7.8, 1.4 Hz, 1H), 4.89 (s, 2H). ^13^C NMR (75 MHz, DMSO-*d*_6_): δ 168.4, 167.4, 152.7, 141.0, 138.5, 132.9, 130.2, 128.4, 123.2, 122.7, 121.8, 119.4, 115.3, 114.4, 68.2. HRMS (ESI) *m*/*z*: [M – H]^−^ calculated for C_15_H_10_O_4_NCl_2_ 337.9992, found 337.9990. Purity by HPLC: peak area >99% (detection at 254 nm).

#### Methyl 4-Chloro-2-(2-(naphthalen-1-yloxy)­acetamido)­benzoate (**43**)

The reaction was carried out according to general procedure C using **7** (1.92 mmol) dissolved in DMF (1 mL), 1-naphtol (2.17 mmol), and K_2_CO_3_ (3.87 mmol). Reaction time: 1 h. Purification: flash column chromatography (cyclohexane/EtOAc, gradient). Yield: 74%. ^1^H NMR (300 MHz, DMSO-*d*_6_): δ 11.89 (s, 1H), 8.80 (d, *J* = 2.1 Hz, 1H), 8.75–8.66 (m, 1H), 8.02 (d, *J* = 8.6 Hz, 1H), 7.96–7.85 (m, 1H), 7.65–7.52 (m, 3H), 7.44 (t, *J* = 8.0 Hz, 1H), 7.32 (dd, *J* = 8.6, 2.2 Hz, 1H), 7.07 (d, *J* = 7.3 Hz, 1H), 4.94 (s, 2H), 3.90 (s, 3H). ^13^C NMR (75 MHz, DMSO-*d*_6_): δ 167.4, 167.0, 152.5, 140.7, 139.0, 134.1, 132.5, 127.4, 126.7, 126.0, 125.5, 124.6, 123.4, 122.2, 121.3, 119.6, 114.7, 106.2, 67.6, 52.7. HRMS (ESI) *m*/*z*: [M + H]^+^ calculated for C_20_H_17_O_4_NCl 370.0841, found 370.0848. Purity by HPLC: peak area >99% (detection at 254 nm).

#### 4-Chloro-2-(2-(naphthalen-1-yloxy)­acetamido)­benzoic Acid (**NBA**)

The reaction was carried out according to general procedure D using **43** (1.40 mmol) dissolved in MeOH (85 mL) and KOH (4.22 mmol) dissolved in H_2_O (17 mL). Reaction time: 1 h. Yield: 95%. X-ray: Single crystals were obtained from *n*-hexane/acetone. ^1^H NMR (300 MHz, DMSO-*d*_6_): δ 13.98 (s, 1H), 12.25 (s, 1H), 8.84 (d, *J* = 2.1 Hz, 1H), 8.72–8.51 (m, 1H), 8.04 (d, *J* = 8.6 Hz, 1H), 7.97–7.84 (m, 1H), 7.64–7.50 (m, 3H), 7.44 (t, *J* = 8.0 Hz, 1H), 7.29 (dd, *J* = 8.6, 2.2 Hz, 1H), 7.06 (d, *J* = 7.4 Hz, 1H), 4.96 (s, 2H). ^13^C NMR (75 MHz, DMSO-*d*_6_): δ 168.8, 167.6, 152.6, 141.2, 138.6, 134.1, 133.0, 127.4, 126.6, 126.0, 125.6, 124.7, 123.2, 121.9, 121.3, 119.3, 115.2, 106.1, 67.8. HRMS (ESI) *m*/*z*: [M – H]^−^ calculated for C_19_H_13_O_4_NCl 354.0539, found 354.0535. Purity by HPLC: peak area >99% (detection at 254 nm).

#### 4-Chloro-2-(2-((5-(prop-2-yn-1-yloxy)­naphthalen-1-yl)­oxy)­acetamido)­benzoic Acid (**87**)

The reaction was carried out according to general procedure E using **7** (1.00 mmol) dissolved in DMF (2 mL), **30** (1.01 mmol), and K_2_CO_3_ (2.01 mmol) for the synthesis of methyl 4-chloro-2-(2-((5-(prop-2-yn-1-yloxy)­naphthalen-1-yl)­oxy)­acetamido)­benzoate (**44**)

Reaction time: 1.5 h. The reaction was then continued according to general procedure E by using **44** dissolved in MeOH (70 mL) and KOH (2.99 mmol) dissolved in H_2_O (14 mL) for the synthesis of **87**. Reaction time: 5 h. Purification: flash column chromatography (cyclohexane/THF + 5% AcOH, gradient). Yield: 70%. ^1^H NMR (300 MHz, DMSO-*d*_6_): δ 13.97 (s, 1H), 12.21 (s, 1H), 8.83 (d, *J* = 2.2 Hz, 1H), 8.20 (d, *J* = 8.5 Hz, 1H), 8.03 (d, *J* = 8.6 Hz, 1H), 7.78 (d, *J* = 8.5 Hz, 1H), 7.45 (dt, *J* = 14.3, 8.1 Hz, 2H), 7.29 (dd, *J* = 8.6, 2.2 Hz, 1H), 7.11 (ddd, *J* = 9.1, 7.9, 0.9 Hz, 2H), 5.02 (d, *J* = 2.4 Hz, 2H), 4.95 (s, 2H), 3.64 (t, *J* = 2.3 Hz, 1H). ^13^C NMR (75 MHz, DMSO-*d*_6_): δ 168.8, 167.6, 152.6, 152.5, 141.2, 138.6, 133.0, 126.0, 125.8, 125.6, 125.5, 123.2, 119.3, 115.1, 114.8, 114.8, 106.8, 79.2, 78.5, 67.9, 56.0. HRMS (ESI) *m*/*z*: [M – H]^−^ calculated for C_22_H_15_O_5_NCl 408.0644, found 408.0642. Purity by HPLC: peak area >99% (detection at 254 nm).

#### Methyl 4-Chloro-2-(2-((8-(prop-2-yn-1-yloxy)­naphthalen-1-yl)­oxy)­acetamido)­benzoate (**45**)

The reaction was carried out according to general procedure C using **7** (1.60 mmol) dissolved in DMF (5 mL), **31** (1.76 mmol), and K_2_CO_3_ (3.21 mmol). Reaction time: 2 h. Purification: flash column chromatography (cyclohexane/EtOAc, gradient). Yield: 27%. ^1^H NMR (300 MHz, DMSO-*d*_6_): δ 11.63 (s, 1H), 8.70 (d, *J* = 2.1 Hz, 1H), 8.03 (d, *J* = 8.6 Hz, 1H), 7.61 (dd, *J* = 8.5, 1.0 Hz, 1H), 7.54 (dd, *J* = 8.5, 1.0 Hz, 1H), 7.45 (td, *J* = 7.9, 2.0 Hz, 2H), 7.33 (dd, *J* = 8.6, 2.2 Hz, 1H), 7.16 (dd, *J* = 7.7, 0.9 Hz, 1H), 7.07 (dd, *J* = 7.9, 1.0 Hz, 1H), 4.79 (d, *J* = 2.3 Hz, 2H), 4.78 (s, 2H), 3.76 (s, 3H), 3.27 (t, *J* = 2.4 Hz, 1H). ^13^C NMR (75 MHz, DMSO-*d*_6_): δ 167.9, 166.3, 153.9, 153.8, 140.5, 138.7, 136.9, 132.5, 126.6, 123.5, 122.9, 121.2, 120.2, 117.7, 117.0, 115.5, 112.2, 108.2, 78.9, 77.8, 70.4, 56.5, 52.6. HRMS (ESI) *m*/*z*: [M + H]^+^ calculated for C_23_H_19_O_5_NCl 424.0946, found 424.0939.

#### 4-Chloro-2-(2-((8-(prop-2-yn-1-yloxy)­naphthalen-1-yl)­oxy)­acetamido)­benzoic Acid (**88**)

The reaction was carried out according to General procedure D using **45** (0.41 mmol) dissolved in MeOH (70 mL) and KOH (0.81 mmol) dissolved in H_2_O (14 mL). Reaction time: 1.5 h. Purification: The product was recrystallized from ACN. Yield: 93%. X-ray: Single crystals were obtained from ACN. ^1^H NMR (300 MHz, DMSO-*d*_6_): δ 13.74 (s, 1H), 12.08 (s, 1H), 8.77 (d, *J* = 2.1 Hz, 1H), 8.02 (d, *J* = 8.6 Hz, 1H), 7.64–7.50 (m, 2H), 7.43 (td, *J* = 7.9, 4.0 Hz, 2H), 7.28 (dd, *J* = 8.6, 2.2 Hz, 1H), 7.19–7.00 (m, 2H), 4.81 (d, *J* = 2.3 Hz, 2H), 4.78 (s, 2H), 3.28 (t, *J* = 2.4 Hz, 1H). ^13^C NMR (75 MHz, DMSO-*d*_6_): δ 168.3, 168.1, 154.1, 153.9, 141.1, 138.4, 136.9, 132.8, 126.6, 126.5, 123.1, 122.8, 121.3, 119.6, 117.8, 115.7, 111.9, 108.6, 79.0, 77.8, 70.6, 56.7. HRMS (ESI) *m*/*z*: [M – H]^−^ calculated for C_22_H_15_O_5_NCl 408.0644, found 408.0639. Purity by HPLC: peak area >97% (detection at 254 nm).

#### Methyl 4-Chloro-2-(2-(3-chlorophenoxy)­acetamido)­benzoate (**46**)

The reaction was carried out according to general procedure C using **7** (0.96 mmol) dissolved in DMF (1.2 mL), 3-chlorophenol (1.08 mmol), and K_2_CO_3_ (1.91 mmol). Reaction time: 1.5 h. Purification: flash column chromatography (cyclohexane/EtOAc, gradient). Yield: 75%. ^1^H NMR (300 MHz, DMSO-*d*_6_): δ 11.77 (s, 1H), 8.70 (d, *J* = 2.2 Hz, 1H), 8.02 (d, *J* = 8.6 Hz, 1H), 7.39 (t, *J* = 8.1 Hz, 1H), 7.31 (dd, *J* = 8.6, 2.2 Hz, 1H), 7.19 (t, *J* = 2.2 Hz, 1H), 7.09 (dtd, *J* = 8.2, 2.5, 0.9 Hz, 2H), 4.82 (s, 2H), 3.91 (s, 3H). ^13^C NMR (75 MHz, DMSO-*d*_6_): δ 167.2, 166.9, 157.8, 140.6, 138.9, 133.8, 132.6, 131.1, 123.4, 121.8, 119.4, 115.1, 114.7, 114.0, 67.4, 52.8. HRMS (ESI) *m*/*z*: [M + H]^+^ calculated for C_16_H_14_O_4_NCl_2_ 354.0294, found 354.0298.

#### 4-Chloro-2-(2-(3-chlorophenoxy)­acetamido)­benzoic Acid (**89**)

The reaction was carried out according to general procedure D using **46** (0.70 mmol) dissolved in MeOH (45 mL) and KOH (2.10 mmol) dissolved in H_2_O (9 mL). Reaction time: 2 h. Yield: 80%. ^1^H NMR (300 MHz, DMSO-*d*_6_): δ 14.08 (s, 1H), 12.25 (s, 1H), 8.77 (d, *J* = 2.2 Hz, 1H), 8.03 (d, *J* = 8.6 Hz, 1H), 7.38 (t, *J* = 8.2 Hz, 1H), 7.27 (dd, *J* = 8.6, 2.2 Hz, 1H), 7.18 (t, *J* = 2.2 Hz, 1H), 7.08 (dddd, *J* = 8.2, 6.2, 2.2, 0.9 Hz, 2H), 4.80 (s, 2H). ^13^C NMR (75 MHz, DMSO-*d*_6_): δ 168.8, 167.2, 157.8, 141.1, 138.7, 133.8, 133.1, 131.1, 123.1, 121.7, 118.8, 115.2, 114.9, 113.6, 67.3. HRMS (ESI) *m*/*z*: [M – H]^−^ calculated for C_15_H_10_O_4_NCl_2_ 337.9992, found 337.9991. Purity by HPLC: peak area >97% (detection at 254 nm).

#### Methyl 4-Chloro-2-(2-(4-chlorophenoxy)­acetamido)­benzoate (**47**)

The reaction was carried out according to general procedure C using **7** (0.84 mmol) dissolved in DMF (4 mL), 4-chlorophenol (0.92 mmol), and K_2_CO_3_ (1.68 mmol). Reaction time: 2.5 h. Purification: flash column chromatography (cyclohexane/EtOAc, gradient). Yield: 36%. ^1^H NMR (300 MHz, DMSO-*d*_6_): δ 11.78 (s, 1H), 8.71 (d, *J* = 2.1 Hz, 1H), 8.02 (d, *J* = 8.6 Hz, 1H), 7.47–7.36 (m, 2H), 7.30 (dd, *J* = 8.6, 2.2 Hz, 1H), 7.19–7.05 (m, 2H), 4.77 (s, 2H), 3.90 (s, 3H). ^13^C NMR (75 MHz, DMSO-*d*_6_): δ 167.3, 166.9, 155.8, 140.6, 138.9, 132.6, 129.5, 125.6, 123.3, 119.3, 116.7, 114.6, 67.5, 52.8. HRMS (ESI) *m*/*z*: [M + H]^+^ calculated for C_16_H_14_O_4_NCl_2_ 354.0294, found 354.0294.

#### 4-Chloro-2-(2-(4-chlorophenoxy)­acetamido)­benzoic Acid (**90**)

The reaction was carried out according to general procedure D using **47** (0.24 mmol) dissolved in MeOH (40 mL) and KOH (0.48 mmol) dissolved in H_2_O (5 mL). Reaction time: 2 h. Yield: 88%. ^1^H NMR (300 MHz, DMSO-*d*_6_): δ 14.06 (s, 1H), 12.24 (s, 1H), 8.76 (d, *J* = 2.1 Hz, 1H), 8.02 (d, *J* = 8.6 Hz, 1H), 7.48–7.34 (m, 2H), 7.26 (dd, *J* = 8.6, 2.2 Hz, 1H), 7.18–7.01 (m, 2H), 4.76 (s, 2H). ^13^C NMR (75 MHz, DMSO-*d*_6_): δ 168.8, 167.4, 155.9, 141.1, 138.7, 133.0, 129.4, 125.5, 123.0, 118.8, 116.6, 114.9, 67.4. HRMS (ESI) *m*/*z*: [M + H]^+^ calculated for C_15_H_12_O_4_NCl_2_ 340.0138, found 340.0130. Purity by HPLC: peak area >99% (detection at 254 nm).

#### 4-Chloro-2-(2-(2,3-dichlorophenoxy)­acetamido)­benzoic Acid (**91**)

The reaction was carried out according to general procedure E using **7** (0.84 mmol) dissolved in DMF (2 mL), 2,3-dichlorophenol (0.78 mmol), and K_2_CO_3_ (1.62 mmol) to obtain crude methyl 4-chloro-2-(2-(2,3-dichlorophenoxy)­acetamido)­benzoate (**48**). Reaction time: 1 h. The reaction was then continued according to general procedure E by using crude **48** dissolved in MeOH (50 mL) and KOH (2.53 mmol) dissolved in H_2_O (10 mL) for the synthesis of **91**. Reaction time: 2 h. Purification: flash column chromatography (H_2_O + 0.1% TFA/ACN + 0.1% TFA, gradient) and (DCM/MeOH, gradient). Yield: 64%. ^1^H NMR (300 MHz, DMSO-*d*_6_): δ 13.93 (s, 1H), 11.91 (s, 1H), 8.74 (d, *J* = 2.1 Hz, 1H), 8.00 (d, *J* = 8.6 Hz, 1H), 7.38–7.24 (m, 3H), 7.18 (dd, *J* = 7.9, 1.9 Hz, 1H), 4.94 (s, 2H). ^13^C NMR (75 MHz, DMSO-*d*_6_): δ 168.5, 167.0, 154.2, 140.9, 138.5, 132.9, 132.5, 128.5, 123.2, 123.2, 120.5, 119.4, 115.3, 112.8, 68.5. HRMS (ESI) *m*/*z*: [M – H]^−^ calculated for C_15_H_9_O_4_NCl_3_ 371.9603, found 371.9601. Purity by HPLC: peak area >97% (detection at 254 nm).

#### Methyl 4-Chloro-2-(2-(2,4-dichlorophenoxy)­acetamido)­benzoate (**49**)

The reaction was carried out according to general procedure C using **7** (1.17 mmol) dissolved in DMF (4 mL), 2,4-dichlorophenol (1.29 mmol) and K_2_CO_3_ (2.34 mmol). Reaction time: 1 h. Purification: flash column chromatography (cyclohexane/EtOAc, gradient). Yield: 54%. ^1^H NMR (300 MHz, DMSO-*d*_6_): δ 11.45 (s, 1H), 8.65 (d, *J* = 2.0 Hz, 1H), 8.00 (d, *J* = 8.6 Hz, 1H), 7.65 (d, *J* = 2.5 Hz, 1H), 7.40 (dd, *J* = 8.9, 2.5 Hz, 1H), 7.32 (dd, *J* = 8.6, 2.1 Hz, 1H), 7.24 (d, *J* = 8.9 Hz, 1H), 4.91 (s, 2H), 3.85 (s, 3H). ^13^C NMR (75 MHz, DMSO-*d*_6_): δ 166.9, 151.8, 140.3, 138.7, 132.5, 129.6, 128.1, 125.8, 123.6, 122.9, 120.0, 117.0, 115.9, 115.2, 68.3, 52.7. HRMS (ESI) *m*/*z*: [M + H]^+^ calculated for C_16_H_13_O_4_NCl_3_ 387.9905, found 387.9899.

#### 4-Chloro-2-(2-(2,4-dichlorophenoxy)­acetamido)­benzoic Acid (**92**)

The reaction was carried out according to general procedure D using **49** (0.60 mmol) dissolved in MeOH (100 mL) and KOH (1.20 mmol) dissolved in H_2_O (20 mL). Reaction time: 1.5 h. Purification: The product was recrystallized from ACN. Yield: 95%. ^1^H NMR (300 MHz, DMSO-*d*_6_): δ 13.93 (s, 1H), 11.90 (s, 1H), 8.74 (d, *J* = 2.1 Hz, 1H), 8.00 (d, *J* = 8.6 Hz, 1H), 7.63 (d, *J* = 2.5 Hz, 1H), 7.38 (dd, *J* = 8.9, 2.6 Hz, 1H), 7.27 (dd, *J* = 8.6, 2.2 Hz, 1H), 7.21 (d, *J* = 8.9 Hz, 1H), 4.91 (s, 2H). ^13^C NMR (75 MHz, DMSO-*d*_6_): δ 168.5, 167.0, 151.9, 141.0, 138.5, 132.9, 129.5, 128.1, 125.7, 123.2, 122.8, 119.4, 115.6, 115.3, 68.3. HRMS (ESI) *m*/*z*: [M – H]^−^ calculated for C_15_H_9_O_4_NCl_3_ 371.9603, found 371.9597. Purity by HPLC: peak area >98% (detection at 254 nm).

#### 4-Chloro-2-(2-(2,5-dichlorophenoxy)­acetamido)­benzoic Acid (**93**)

The reaction was carried out according to general procedure E using **7** (0.879 mmol) dissolved in DMF (4 mL), 2,5-dichlorophenol (0.98 mmol), and K_2_CO_3_ (1.77 mmol) for the synthesis of methyl 4-chloro-2-(2-(2,5-dichlorophenoxy)­acetamido)­benzoate (**50**). Reaction time: 120 min. The reaction was then continued according to general procedure E by using **50** dissolved MeOH (40 mL) and THF (10 mL) and KOH (2.638 mmol) dissolved in H_2_O (10 mL) for the synthesis of **93**. Reaction time: 60 min. Yield: 70%. ^1^H NMR (300 MHz, DMSO-*d*_6_): δ 13.93 (s, 1H), 11.88 (s, 1H), 8.74 (d, *J* = 2.2 Hz, 1H), 8.01 (d, *J* = 8.6 Hz, 1H), 7.51 (d, *J* = 8.5 Hz, 1H), 7.35 (d, *J* = 2.3 Hz, 1H), 7.28 (dd, *J* = 8.6, 2.2 Hz, 1H), 7.11 (dd, *J* = 8.5, 2.3 Hz, 1H), 4.96 (s, 2H). ^13^C NMR (75 MHz, DMSO-*d*_6_): δ 168.5, 166.8, 153.3, 141.0, 138.5, 132.9, 132.3, 131.1, 123.2, 122.5, 120.7, 119.4, 115.3, 114.8, 68.2. HRMS (ESI) *m*/*z*: [M – H]^−^ calculated for C_15_H_9_O_4_NCl_3_ 371.9592, found 371.9610. Purity by HPLC: peak area >99% (detection at 254 nm).

#### 4-Chloro-2-(2-(2,6-dichlorophenoxy)­acetamido)­benzoic Acid (**94**)

The reaction was carried out according to general procedure E using **7** (1.17 mmol) dissolved in DMF (6 mL), 2,6-dichlorophenol (1.30 mmol), and K_2_CO_3_ (2.33 mmol) for the synthesis of methyl 4-chloro-2-(2-(2,6-dichlorophenoxy)­acetamido)­benzoate (**51**). Reaction time: 60 min. The reaction was then continued according to general procedure E by using **51** dissolved in MeOH (80 mL) and KOH (3.51 mmol) dissolved in H_2_O (16 mL) for the synthesis of **94**. Reaction time: 30 min. Purification: Recrystallization from cyclohexane/THF. Yield: 74%. ^1^H NMR (300 MHz, DMSO-*d*_6_): δ 13.91 (s, 1H), 12.33 (s, 1H), 8.78 (d, *J* = 2.1 Hz, 1H), 8.04 (d, *J* = 8.6 Hz, 1H), 7.55 (d, *J* = 8.1 Hz, 2H), 7.36–7.19 (m, 2H), 4.67 (s, 2H). ^13^C NMR (75 MHz, DMSO-*d*_6_): δ 168.4, 166.6, 149.3, 140.9, 138.6, 133.0, 129.4, 128.3, 127.0, 123.3, 119.1, 115.3, 71.3. HRMS (ESI) *m*/*z*: [M – H]^−^ calculated for C_15_H_9_O_4_NCl_3_ 371.9603, found 371.9601. Purity by HPLC: peak area >98% (detection at 254 nm).

#### Methyl 4-Chloro-2-(2-(3,4-dichlorophenoxy)­acetamido)­benzoate (**52**)

The reaction was carried out according to general procedure C using **7** (1.55 mmol) dissolved in DMF (5 mL), 3,4-dichlorophenol (1.70 mmol), and K_2_CO_3_ (3.09 mmol). Reaction time: 60 min. Purification: flash column chromatography (cyclohexane/EtOAc, gradient). Yield: 84%. ^1^H NMR: (300 MHz, CDCl_3_) δ 12.15 (s, 1H), 8.87 (d, *J* = 2.0 Hz, 1H), 7.99 (d, *J* = 8.6 Hz, 1H), 7.40 (d, *J* = 8.9 Hz, 1H), 7.20 (d, *J* = 2.9 Hz, 1H), 7.12 (dd, *J* = 8.6, 2.1 Hz, 1H), 7.00–6.94 (m, 1H), 4.62 (s, 2H), 3.96 (s, 3H). HRMS (ESI) *m*/*z*: [M + H]^+^ calculated for C_16_H_13_O_4_NCl_3_ 387.9905, found 387.9900.

#### 4-Chloro-2-(2-(3,4-dichlorophenoxy)­acetamido)­benzoic Acid (**95**)

The reaction was carried out according to general procedure D using (1.25 mmol) dissolved in MeOH (150 mL) and KOH (2.51 mmol) dissolved in H_2_O (30 mL). Reaction time: 60 min. Purification: The product was recrystallized from ACN. Yield: 77%. X-ray: Single crystals were obtained from DMSO-*d*_6_. ^1^H NMR (300 MHz, DMSO-*d*_6_): δ 14.09 (s, 1H), 12.22 (s, 1H), 8.74 (d, *J* = 2.1 Hz, 1H), 8.01 (d, *J* = 8.6 Hz, 1H), 7.60 (d, *J* = 8.9 Hz, 1H), 7.36 (d, *J* = 2.9 Hz, 1H), 7.26 (dd, *J* = 8.6, 2.2 Hz, 1H), 7.09 (dd, *J* = 8.9, 2.9 Hz, 1H), 4.80 (s, 2H). ^13^C NMR (75 MHz, DMSO-*d*_6_): δ 168.8, 166.9, 156.3, 141.1, 138.7, 133.0, 131.7, 131.2, 123.7, 123.1, 118.8, 117.0, 115.5, 114.9, 67.6. HRMS (ESI) *m*/*z*: [M – H]^−^ calculated for C_15_H_9_O_4_NCl_3_ 371.9603, found 371.9595. Purity by HPLC: peak area >99% (detection at 254 nm).

#### 4-Chloro-2-(2-(3,5-dichlorophenoxy)­acetamido)­benzoic Acid (**96**)

The reaction was carried out according to general procedure E using **7** (1.19 mmol) dissolved in DMF (4 mL), 3,5-dichlorophenol (1.32 mmol), and K_2_CO_3_ (2.45 mmol) for the synthesis of crude methyl 4-chloro-2-(2-(3,5-dichlorophenoxy)­acetamido)­benzoate (**53**). Reaction time: 90 min. The reaction was then continued according to general procedure E by using crude **53** dissolved in MeOH (75 mL) and KOH (3.58 mmol) dissolved in H_2_O (15 mL) for the synthesis of **96**. Reaction time: 180 min. Yield: 37%. ^1^H NMR (300 MHz, DMSO-*d*_6_): δ 14.11 (s, 1H), 12.23 (s, 1H), 8.75 (d, *J* = 2.1 Hz, 1H), 8.03 (d, *J* = 8.6 Hz, 1H), 7.31–7.23 (m, 2H), 7.18 (d, *J* = 1.8 Hz, 2H), 4.84 (s, 2H). ^13^C NMR (75 MHz, DMSO-*d*_6_): δ 168.8, 166.8, 158.2, 141.1, 138.7, 134.7, 133.1, 123.1, 121.5, 118.8, 115.0, 114.3, 67.6. HRMS (ESI) *m*/*z*: [M – H]^−^ calculated for C_15_H_9_O_4_NCl_3_ 371.9603, found 371.9602. Purity by HPLC: peak area >97% (detection at 254 nm).

#### 4-Chloro-2-(2-(2,6-dichloro-3-methylphenoxy)­acetamido)­benzoic Acid (**97**)

The reaction was carried out according to general procedure E using **7** (1.21 mmol) dissolved in DMF (6 mL), 2,6-dichloro-3-methylphenol (1.41 mmol), and K_2_CO_3_ (2.51 mmol) for the synthesis of crude methyl 4-chloro-2-(2-(2,6-dichloro-3-methylphenoxy)­acetamido)­benzoate (**54**). Reaction time: 75 min. The reaction was then continued according to general procedure E by using crude **54** dissolved in MeOH (50 mL) and KOH (3.68 mmol) dissolved in H_2_O (10 mL) for the synthesis of **97**. Reaction time: 60 min. Purification: flash column chromatography (DCM/MeOH, gradient) and a second precipitation. Yield: 33%. ^1^H NMR (300 MHz, DMSO-*d*_6_): δ 13.89 (s, 1H), 12.34 (s, 1H), 8.78 (d, *J* = 2.1 Hz, 1H), 8.04 (d, *J* = 8.6 Hz, 1H), 7.44 (d, *J* = 8.3 Hz, 1H), 7.30 (dd, *J* = 8.6, 2.2 Hz, 1H), 7.27–7.21 (m, 1H), 4.64 (s, 2H), 2.34 (s, 3H). ^13^C NMR (75 MHz, DMSO-*d*_6_): δ 168.4, 166.7, 149.2, 141.0, 138.6, 136.9, 133.0, 128.6, 128.2, 127.8, 125.3, 123.2, 119.1, 115.3, 71.1, 19.6. HRMS (ESI) *m*/*z*: [M – H]^−^ calculated for C_16_H_11_O_4_NCl_3_ 385.9759, found 385.9757. Purity by HPLC: peak area >98% (detection at 254 nm).

#### 4-Chloro-2-(2-(3-chloro-4-nitrophenoxy)­acetamido)­benzoic Acid (**98**)

The reaction was carried out according to general procedure E using **7** (1.55 mmol) dissolved in DMF (10 mL), 3-chloro-4-nitrophenol (1.70 mmol), and K_2_CO_3_ (3.09 mmol) for the synthesis of methyl 4-chloro-2-(2-(3-chloro-4-nitrophenoxy)­acetamido)­benzoate (**55**). Reaction time: 1 h. Purification: flash column chromatography (cyclohexane/EtOAc, gradient). The reaction was then continued according to general procedure E by using **55** dissolved in MeOH (50 mL) and KOH (2.58 mmol) dissolved in H_2_O (10 mL) for the synthesis of **98**. Reaction time: 20 min. Purification: flash column chromatography (cyclohexane/THF, gradient). Yield (over 2 steps): 27%. ^1^H NMR (300 MHz, DMSO-*d*_6_) δ 14.12 (s, 1H), 12.25 (s, 1H), 8.73 (d, *J* = 2.1 Hz, 1H), 8.18 (d, *J* = 9.1 Hz, 1H), 8.03 (d, *J* = 8.6 Hz, 1H), 7.43 (d, *J* = 2.6 Hz, 1H), 7.26 (ddd, *J* = 10.5, 8.9, 2.4 Hz, 2H), 4.96 (s, 2H). ^13^C NMR (75 MHz, DMSO-*d*_6_) δ 168.8, 166.4, 160.3, 141.2, 141.0, 138.7, 133.1, 128.3, 127.7, 123.2, 118.8, 117.7, 115.1, 114.6, 67.8. HRMS (ESI) *m*/*z*: [M – H]^−^ calculated for C_15_H_9_O_6_N_2_Cl_2_ 382.9843, found 382.9846. Purity by HPLC: peak area >98% (detection at 254 nm).

#### Methyl 2-(2-(2-Bromophenoxy)­acetamido)-4-chlorobenzoate (**56**)

The reaction was carried out according to general procedure C using **7** (0.96 mmol) dissolved in DMF (1 mL), 2-bromophenol (1.12 mmol), and K_2_CO_3_ (1.92 mmol). Reaction time: 120 min. Purification: flash column chromatography (cyclohexane/EtOAc, gradient). Yield: 56%. ^1^H NMR (300 MHz, DMSO-*d*_6_): δ 11.41 (s, 1H), 8.64 (d, *J* = 2.1 Hz, 1H), 7.98 (d, *J* = 8.6 Hz, 1H), 7.64 (dd, *J* = 7.9, 1.6 Hz, 1H), 7.40–7.28 (m, 2H), 7.18 (dd, *J* = 8.3, 1.4 Hz, 1H), 6.98 (td, *J* = 7.4, 1.4 Hz, 1H), 4.88 (s, 2H), 3.84 (s, 3H). ^13^C NMR (75 MHz, DMSO-*d*_6_): δ 167.2, 166.5, 153.6, 140.3, 138.7, 133.3, 132.4, 129.0, 123.5, 123.4, 120.0, 115.3, 114.8, 111.4, 68.4, 52.7.

#### 4-Chloro-2-(2-(2-bromophenoxy)­acetamido)­benzoic Acid (**99**)

The reaction was carried out according to general procedure D using **56** (0.49 mmol) dissolved in MeOH (30 mL) and KOH (1.68 mmol) dissolved in H_2_O (8 mL). Reaction time: 60 min. Yield: 89%. ^1^H NMR (300 MHz, DMSO-*d*_6_): δ 13.90 (s, 1H), 11.88 (s, 1H), 8.74 (d, *J* = 2.2 Hz, 1H), 8.00 (d, *J* = 8.6 Hz, 1H), 7.63 (dd, *J* = 7.9, 1.6 Hz, 1H), 7.35 (ddd, *J* = 8.3, 7.4, 1.6 Hz, 1H), 7.27 (dd, *J* = 8.5, 2.2 Hz, 1H), 7.14 (dd, *J* = 8.4, 1.4 Hz, 1H), 6.97 (td, *J* = 7.6, 1.4 Hz, 1H), 4.88 (s, 2H). ^13^C NMR (75 MHz, DMSO-*d*_6_): δ 168.4, 167.4, 153.7, 141.0, 138.5, 133.3, 132.9, 129.0, 123.3, 123.2, 119.5, 115.3, 114.5, 111.3, 68.5. HRMS (ESI) *m*/*z*: [M – H]^−^ calculated for C_15_H_10_O_4_NBrCl 381.9487, found 381.9487. Purity by HPLC: peak area >98% (detection at 254 nm).

#### Methyl 2-(2-(3-Bromophenoxy)­acetamido)-4-chlorobenzoate (**57**)

The reaction was carried out according to general procedure C using **7** (0.38 mmol) dissolved in DMF (1 mL), 3-bromophenol (0.48 mmol), and K_2_CO_3_ (0.80 mmol). Reaction time: 120 min. Purification: flash column chromatography (cyclohexane/EtOAc, gradient). Yield: 43%. ^1^H NMR (300 MHz, DMSO-*d*_6_): δ 11.76 (s, 1H), 8.70 (d, *J* = 2.2 Hz, 1H), 8.02 (d, *J* = 8.6 Hz, 1H), 7.37–7.28 (m, 3H), 7.23 (ddd, *J* = 7.9, 1.8, 1.0 Hz, 1H), 7.13 (ddd, *J* = 8.3, 2.5, 1.1 Hz, 1H), 4.81 (s, 2H), 3.91 (s, 3H). ^13^C NMR (75 MHz, DMSO-*d*_6_): δ 167.2, 166.9, 157.9, 140.6, 138.9, 132.6, 131.4, 124.7, 123.4, 122.1, 119.4, 117.9, 114.7, 114.4, 67.4, 52.8. HRMS (ESI) *m*/*z*: [M + H]^+^ calculated for C_16_H_14_O_4_NBrCl 397.9789, found 397.9785.

#### 4-Chloro-2-(2-(3-bromophenoxy)­acetamido)­benzoic Acid (**100**)

The reaction was carried out according to general procedure D using **57** (0.15 mmol) dissolved in MeOH (10 mL) and KOH (0.45 mmol) dissolved in H_2_O (1 mL). Reaction time: 60 min. Yield: 71%. ^1^H NMR (300 MHz, DMSO-*d*_6_): δ 14.09 (s, 1H), 12.25 (s, 1H), 8.77 (d, *J* = 2.1 Hz, 1H), 8.03 (d, *J* = 8.5 Hz, 1H), 7.36–7.25 (m, 3H), 7.22 (dd, *J* = 6.9, 1.6 Hz, 1H), 7.11 (dd, *J* = 8.3, 3.3 Hz, 1H), 4.79 (s, 2H). ^13^C NMR (75 MHz, DMSO-*d*_6_): δ 168.8, 167.2, 157.8, 141.1, 138.7, 133.1, 131.4, 124.6, 123.1, 122.1, 118.8, 118.1, 114.9, 113.9, 67.3. HRMS (ESI) *m*/*z*: [M – H]^−^ calculated for C_15_H_10_O_4_NBrCl 381.9487, found 381.9490. Purity by HPLC: peak area >99% (detection at 254 nm).

#### Methyl 2-(2-(4-Bromophenoxy)­acetamido)-4-chlorobenzoate (**58**)

The reaction was carried out according to general procedure C using **7** (1.31 mmol) dissolved in DMF (5 mL), 4-bromophenol (1.44 mmol), and K_2_CO_3_ (2.61 mmol). Reaction time: 60 min. Purification: flash column chromatography (cyclohexane/EtOAc, gradient). Yield: 71%. ^1^H NMR (300 MHz, DMSO-*d*_6_): δ 11.77 (s, 1H), 8.70 (d, *J* = 2.1 Hz, 1H), 8.01 (d, *J* = 8.6 Hz, 1H), 7.59–7.47 (m, 2H), 7.30 (dd, *J* = 8.6, 2.2 Hz, 1H), 7.13–7.00 (m, 2H), 4.77 (s, 2H), 3.90 (s, 3H). ^13^C NMR (75 MHz, DMSO-*d*_6_): δ 167.3, 166.9, 156.3, 140.6, 138.9, 132.6, 132.4, 123.3, 119.3, 117.2, 114.6, 113.3, 67.4, 52.8. HRMS (ESI) *m*/*z*: [M + H]^+^ calculated for C_16_H_14_O_4_NBrCl 397.9789, found 397.9779.

#### 2-(2-(4-Bromophenoxy)­acetamido)-4-chlorobenzoic Acid (**101**)

The reaction was carried out according to general procedure D using **58** (0.88 mmol) dissolved in MeOH (70 mL) and KOH (1.77 mmol) dissolved in H_2_O (14 mL). Reaction time: 1 h. Purification: The precipitate was recrystallized from ACN. Yield: 94%. X-ray: Single crystals were obtained from ACN. ^1^H NMR (300 MHz, DMSO-*d*_6_): δ 14.08 (s, 1H), 12.24 (s, 1H), 8.77 (d, *J* = 2.1 Hz, 1H), 8.02 (d, *J* = 8.6 Hz, 1H), 7.58–7.47 (m, 2H), 7.26 (dd, *J* = 8.6, 2.2 Hz, 1H), 7.10–7.01 (m, 2H), 4.76 (s, 2H). ^13^C NMR (75 MHz, DMSO-*d*_6_): δ 168.8, 167.3, 156.3, 141.1, 138.7, 133.0, 132.3, 123.1, 118.8, 117.1, 114.9, 113.2, 67.4. HRMS (ESI) *m*/*z*: [M – H]^−^ calculated for C_15_H_10_O_4_NBrCl 381.9487, found 381.9485. Purity by HPLC: peak area >99% (detection at 254 nm).

#### 4-Chloro-2-(2-(3,5-dibromophenoxy)­acetamido)­benzoic Acid (**102**)

The reaction was carried out according to general procedure E using **7** (0.87 mmol) dissolved in DMF (4 mL), 3,5-dibromophenol (0.96 mmol), and K_2_CO_3_ (1.73 mmol) for the synthesis of methyl 4-chloro-2-(2-(3,5-dibromophenoxy)­acetamido)­benzoate (**59**). Reaction time: 150 min. The reaction was then continued according to General Procedure E by using **59** dissolved in THF (150 mL) and KOH (6.31 mmol) dissolved in H_2_O (60 mL) for the synthesis of **102**. Reaction time: 1 h. Yield: 80%. X-ray: Single crystals were obtained from DMSO-*d*_6_. ^1^H NMR (300 MHz, DMSO-*d*_6_): δ 14.13 (s, 1H), 12.23 (s, 1H), 8.76 (d, *J* = 2.2 Hz, 1H), 8.03 (d, *J* = 8.6 Hz, 1H), 7.48 (t, *J* = 1.6 Hz, 1H), 7.34 (d, *J* = 1.6 Hz, 2H), 7.27 (dd, *J* = 8.5, 2.2 Hz, 1H), 4.83 (s, 2H). ^13^C NMR (75 MHz, DMSO-*d*_6_): δ 168.8, 166.8, 158.3, 141.1, 138.7, 133.1, 126.8, 123.1, 122.9, 118.8, 117.4, 115.0, 67.5. HRMS (ESI) *m*/*z*: [M – H]^−^ calculated for C_15_H_9_O_4_NBr_3_Cl 459.8592, found 459.8596. Purity by HPLC: peak area >98% (detection at 254 nm).

#### Methyl 4-Chloro-2-(2-(3-iodophenoxy)­acetamido)­benzoate (**60**)

The reaction was carried out according to general procedure C using **7** (1.25 mmol) dissolved in DMF (12 mL), 3-iodophenol (1.37 mmol), and K_2_CO_3_ (2.51 mmol). Reaction time: 60 min. Purification: flash column chromatography (cyclohexane/EtOAc, gradient). Yield: 84%. ^1^H NMR (300 MHz, DMSO-*d*_6_) δ 11.76 (s, 1H), 8.70 (d, *J* = 2.1 Hz, 1H), 8.01 (d, *J* = 8.6 Hz, 1H), 7.48 (t, *J* = 1.9 Hz, 1H), 7.40 (ddd, *J* = 6.4, 2.5, 1.6 Hz, 1H), 7.30 (dd, *J* = 8.6, 2.2 Hz, 1H), 7.15–7.09 (m, 2H), 4.79 (s, 2H), 3.91 (s, 3H). ^13^C NMR (75 MHz, DMSO-*d*_6_) δ 167.2, 166.9, 157.6, 140.6, 138.9, 132.6, 131.5, 130.7, 123.7, 123.3, 119.3, 114.8, 114.6, 95.0, 67.4, 52.8. HRMS (ESI) *m*/*z*: [M + H]^+^ calculated for C_16_H_13_ClINO_4_ 445.9651, found 445.9647.

#### 4-Chloro-2-(2-(3-iodophenoxy)­acetamido)­benzoic Acid (**103**)

The reaction was carried out according to general procedure D using **60** (1.02 mmol) dissolved in MeOH (100 mL) and KOH (3.11 mmol) dissolved in H_2_O (20 mL). Reaction time: 30 min. Yield 79%. ^1^H NMR (300 MHz, DMSO-*d*_6_) δ 14.08 (s, 1H), 12.25 (s, 1H), 8.77 (d, *J* = 2.1 Hz, 1H), 8.03 (d, *J* = 8.6 Hz, 1H), 7.48–7.44 (m, 1H), 7.38 (dt, *J* = 7.0, 1.7 Hz, 1H), 7.27 (dd, *J* = 8.6, 2.2 Hz, 1H), 7.19–7.08 (m, 2H), 4.77 (s, 2H). ^13^C NMR (75 MHz, DMSO-*d*_6_): δ 168.8, 167.2, 157.6, 141.1, 138.7, 133.1, 131.5, 130.5, 123.7, 123.1, 118.8, 114.9, 114.3, 95.1, 67.2. HRMS (ESI) *m*/*z*: [M – H]^−^ calculated for C_15_H_10_O_4_NClI 429.9349, found 429.9348. Purity by HPLC: peak area >98% (detection at 254 nm).

#### Methyl 4-Chloro-2-(2-(3-fluorophenoxy)­acetamido)­benzoate (**61**)

The reaction was carried out according to general procedure C using **7** (1.00 mmol) dissolved in DMF (2 mL), 3-fluorophenol (1.10 mmol), and K_2_CO_3_ (2.07 mmol). Reaction time: 60 min. Purification: flash column chromatography (cyclohexane/EtOAc, gradient). Yield: 70%. ^1^H NMR (300 MHz, DMSO-*d*_6_): δ 11.78 (s, 1H), 8.69 (d, *J* = 2.2 Hz, 1H), 7.99 (d, *J* = 8.6 Hz, 1H), 7.44–7.34 (m, 1H), 7.28 (dd, *J* = 8.6, 2.2 Hz, 1H), 7.01–6.92 (m, 2H), 6.86 (tdd, *J* = 8.5, 2.3, 1.0 Hz, 1H), 4.78 (s, 2H), 3.90 (s, 3H). ^13^C NMR (75 MHz, DMSO-*d*_6_): δ 167.2, 166.9, 140.6, 139.0, 132.5, 131.0, 123.3, 119.3, 114.5, 111.3, 108.6, 108.3, 102.8, 102.4, 67.4, 52.8. HRMS (ESI) *m*/*z*: [M + H]^+^ calculated for C_16_H_14_O_4_NClF 338.0590, found 338.0589.

#### 4-Chloro-2-(2-(3-fluorophenoxy)­acetamido)­benzoic Acid (**104**)

The reaction was carried out according to general procedure D using **61** (0.64 mmol) dissolved in MeOH (20 mL) and KOH (2.00 mmol) dissolved in H_2_O (7 mL). Reaction time: 90 min. Yield: 86%. ^1^H NMR (300 MHz, DMSO-*d*_6_): δ 14.06 (s, 1H), 12.23 (s, 1H), 8.77 (d, *J* = 2.2 Hz, 1H), 8.02 (d, *J* = 8.6 Hz, 1H), 7.38 (td, *J* = 8.3, 7.0 Hz, 1H), 7.26 (dd, *J* = 8.6, 2.2 Hz, 1H), 7.00–6.91 (m, 2H), 6.90–6.81 (m, 1H), 4.78 (s, 2H). ^13^C NMR (75 MHz, DMSO-*d*_6_): δ 168.8, 167.2, 158.4, 158.3, 141.1, 138.7, 133.0, 131.0, 130.9, 123.1, 118.8, 115.0, 111.1, 108.6, 108.3, 102.8, 102.5, 67.4. ^19^F NMR (282 MHz, DMSO-*d*_6_): δ −111.40 (q, *J* = 9.2 Hz). HRMS (ESI) *m*/*z*: [M – H]^−^ calculated for C_15_H_10_O_4_NClF 322.0277, found 322.0285. Purity by HPLC: peak area >99% (detection at 254 nm).

#### Methyl 4-Chloro-2-(2-(3-(trifluoromethyl)­phenoxy)­acetamido)­benzoate (**62**)

The reaction was carried out according to general procedure C using **7** (0.90 mmol) dissolved in DMF (2 mL), 3-(trifluoromethyl)­phenol (0.99 mmol), and K_2_CO_3_ (1.84 mmol). Reaction time: 90 min. Purification: flash column chromatography (cyclohexane/EtOAc, gradient). Yield: 65%. ^1^H NMR (300 MHz, DMSO-*d*_6_): δ 11.80 (s, 1H), 8.69 (d, *J* = 2.2 Hz, 1H), 8.01 (d, *J* = 8.6 Hz, 1H), 7.61 (tt, *J* = 7.6, 0.9 Hz, 1H), 7.45–7.37 (m, 3H), 7.30 (dd, *J* = 8.6, 2.2 Hz, 1H), 4.89 (s, 2H), 3.90 (s, 3H). ^13^C NMR (75 MHz, DMSO-*d*_6_): δ 167.1, 166.9, 157.3, 140.6, 139.0, 132.6, 131.0, 123.4, 119.3, 119.1, 118.4, 114.7, 111.9, 111.8, 67.5, 52.7.

#### 4-Chloro-2-(2-(3-(trifluoromethyl)­phenoxy)­acetamido)­benzoic Acid (**105**)

The reaction was carried out according to general procedure D using **62** (0.56 mmol) dissolved in MeOH (37 mL) and KOH (2.02 mmol) dissolved in H_2_O (7 mL). Reaction time: 120 min. Yield: 85%. ^1^H NMR (300 MHz, DMSO-*d*_6_): δ 14.08 (s, 1H), 12.26 (s, 1H), 8.78 (d, *J* = 2.2 Hz, 1H), 8.03 (d, *J* = 8.5 Hz, 1H), 7.65–7.56 (m, 1H), 7.43–7.36 (m, 3H), 7.28 (dd, *J* = 8.5, 2.2 Hz, 1H), 4.88 (s, 2H). ^13^C NMR (75 MHz, DMSO-*d*_6_): δ 168.8, 167.1, 157.2, 141.1, 138.7, 133.1, 131.0, 123.1, 118.8, 118.3, 115.0, 111.8, 67.4. ^19^F NMR (282 MHz, DMSO-*d*_6_): δ −61.07. HRMS (ESI) *m*/*z*: [M – H]^−^ calculated for C_16_H_10_O_4_NClF_3_ 372.0256, found 372.0253. Purity by HPLC: peak area >99% (detection at 254 nm).

#### Methyl 4-Chloro-2-(2-(2-nitrophenoxy)­acetamido)­benzoate (**63**)

The reaction was carried out according to general procedure C using **7** (1.94 mmol), 2-nitrophenol (2.140 mmol), and K_2_CO_3_ (3.88 mmol) dissolved in DMF (10 mL). Reaction time: 100 min. Purification: flash column chromatography (cyclohexane/EtOAc, gradient). Yield: 22%. ^1^H NMR (300 MHz, DMSO-*d*_6_): δ 11.35 (s, 1H), 8.56 (d, *J* = 2.2 Hz, 1H), 8.00–7.93 (m, 2H), 7.68 (ddd, *J* = 8.5, 7.4, 1.7 Hz, 1H), 7.42 (dd, *J* = 8.6, 1.2 Hz, 1H), 7.32 (dd, *J* = 8.6, 2.2 Hz, 1H), 7.22 (ddd, *J* = 8.3, 7.4, 1.1 Hz, 1H), 5.03 (s, 2H), 3.83 (s, 3H). ^13^C NMR (75 MHz, DMSO-*d*_6_): δ 166.6, 166.5, 150.0, 140.1, 139.7, 138.7, 134.6, 132.4, 125.4, 123.6, 122.0, 120.0, 115.9, 115.4, 68.3, 52.6. HRMS (ESI) *m*/*z*: [M + H]^+^ calculated for C_16_H_14_O_6_N_2_Cl 365.0535, found 365.0527.

#### 4-Chloro-2-(2-(2-nitrophenoxy)­acetamido)­benzoic Acid (**106**)

The reaction was carried out according to general procedure D using **63** (0.32 mmol) dissolved in MeOH (50 mL) and KOH (0.96 mmol) dissolved in H_2_O (10 mL). Reaction time: 20 min. Purification: flash column chromatography (DCM/MeOH, gradient). Yield: 78%. ^1^H NMR (300 MHz, DMSO-*d*_6_) δ 13.89 (s, 1H), 11.82 (s, 1H), 8.69 (s, 1H), 7.98 (t, *J* = 8.9 Hz, 2H), 7.68 (t, *J* = 8.1 Hz, 1H), 7.46–7.33 (m, 1H), 7.33–7.15 (m, 2H), 5.02 (s, 2H). ^13^C NMR (75 MHz, DMSO-*d*_6_): δ 168.4, 166.8, 150.2, 140.9, 139.6, 138.5, 134.7, 132.9, 125.4, 123.3, 122.0, 119.4, 115.9, 115.3, 68.5. ^1^H- and ^13^C NMR peaks were extracted from a crude NMR. HRMS (ESI) *m*/*z*: [M – H]^−^ calculated for C_15_H_10_O_6_N_2_Cl 349.0233, found 349.0229. Purity by HPLC: peak area >95% (detection at 254 nm).

#### Methyl 4-Chloro-2-(2-(3-nitrophenoxy)­acetamido)­benzoate (**64**)

The reaction was carried out according to general procedure C using **7** (1.91 mmol) dissolved in DMF (1 mL), 3-nitrophenol (2.14 mmol), and K_2_CO_3_ (3.86 mmol). Reaction time: 120 min. Purification: flash column chromatography (cyclohexane/EtOAc, gradient). Yield: 59%. ^1^H NMR (300 MHz, DMSO-*d*_6_): δ 11.79 (s, 1H), 8.67 (d, *J* = 2.2 Hz, 1H), 8.01 (d, *J* = 8.6 Hz, 1H), 7.93–7.88 (m, 2H), 7.71–7.63 (m, 1H), 7.58 (ddd, *J* = 8.3, 2.4, 1.2 Hz, 1H), 7.30 (dd, *J* = 8.6, 2.2 Hz, 1H), 4.94 (s, 2H), 3.91 (s, 3H). ^13^C NMR (75 MHz, DMSO-*d*_6_): δ 166.9, 166.9, 157.5, 148.7, 140.5, 138.9, 132.6, 131.0, 123.4, 122.1, 119.4, 116.7, 114.8, 109.8, 67.7, 52.8. HRMS (ESI) *m*/*z*: [M + H]^+^ calculated for C_16_H_14_O_6_N_2_Cl 365.0535, found 365.0536.

#### 4-Chloro-2-(2-(3-nitrophenoxy)­acetamido)­benzoic Acid (**107**)

The reaction was carried out according to general procedure D using **64** (0.17 mmol) dissolved in MeOH (10 mL) and KOH (0.50 mmol) dissolved in H_2_O (2 mL). Reaction time: 60 min. Yield: 55%. ^1^H NMR (300 MHz, DMSO-*d*_6_): δ 14.10 (s, 1H), 12.27 (s, 1H), 8.75 (d, *J* = 2.1 Hz, 1H), 8.02 (d, *J* = 8.6 Hz, 1H), 7.93–7.82 (m, 2H), 7.66 (t, *J* = 8.2 Hz, 1H), 7.55 (dd, *J* = 8.0, 1.8 Hz, 1H), 7.26 (dd, *J* = 8.6, 2.2 Hz, 1H), 4.92 (s, 2H). ^13^C NMR (75 MHz, DMSO-*d*_6_): δ 168.8, 166.8, 157.4, 148.7, 141.1, 138.7, 133.0, 131.0, 123.1, 121.6, 118.8, 116.6, 115.0, 109.8, 67.6. HRMS (ESI) *m*/*z*: [M – H]^−^ calculated for C_15_H_10_O_6_N_2_Cl 349.0233, found 349.0231. Purity by HPLC: peak area >99% (detection at 254 nm).

#### Methyl 4-Chloro-2-(2-(4-nitrophenoxy)­acetamido)­benzoate (**65**)

The reaction was carried out according to general procedure C using **7** (1.83 mmol) dissolved in DMF (1 mL), 4-nitrophenol (2.01 mmol) and K_2_CO_3_ (4.28 mmol). Reaction time: 150 min. Purification: flash column chromatography (cyclohexane/EtOAc, gradient). Yield: 20%. ^1^H NMR (300 MHz, DMSO-*d*_6_) δ 11.74 (s, 1H), 8.68 (d, *J* = 2.2 Hz, 1H), 8.34–8.25 (m, 2H), 8.03 (d, *J* = 8.6 Hz, 1H), 7.39–7.25 (m, 3H), 4.98 (s, 2H), 3.91 (s, 3H).

#### 4-Chloro-2-(2-(4-nitrophenoxy)­acetamido)­benzoic Acid (**108**)

The reaction was carried out according to general procedure D using **65** (0.38 mmol) dissolved in MeOH (50 mL) and KOH (0.77 mmol) dissolved in H_2_O (10 mL). Reaction time: 120 min. Yield: not determined. ^1^H NMR (300 MHz, DMSO-*d*_6_): δ 14.12 (s, 1H), 12.25 (s, 1H), 8.75 (d, *J* = 2.1 Hz, 1H), 8.34–8.22 (m, 2H), 8.03 (d, *J* = 8.6 Hz, 1H), 7.32–7.22 (m, 3H), 4.95 (s, 2H). ^13^C NMR (75 MHz, DMSO-*d*_6_): δ 168.9, 166.7, 162.0, 141.7, 141.1, 138.7, 133.1, 126.0, 123.2, 118.9, 115.4, 115.0, 67.6. HRMS (ESI) *m*/*z*: [M – H]^−^ calculated for C_15_H_10_O_6_N_2_Cl 349.0233, found 349.0228. Purity by HPLC: peak area >97% (detection at 254 nm).

#### 4-Chloro-2-(2-(3-(diethylamino)­phenoxy)­acetamido)­benzoic Acid (**109**)

The reaction was carried out according to general procedure E using **7** (1.55 mmol) dissolved in DMF (8 mL), 3-diethylaminophenol (2.61 mmol), and K_2_CO_3_ (3.11 mmol) for the synthesis of methyl 4-chloro-2-(2-(3-(diethylamino)­phenoxy)­acetamido)­benzoate (**66**). Reaction time: 90 min. Purification: flash column chromatography (cyclohexane/EtOAc, gradient). The reaction was then continued according to general procedure E by using **66** dissolved in MeOH (100 mL) and KOH (1.11 mmol) dissolved in H_2_O (20 mL) for the synthesis of **109**. Reaction time: 90 min. Purification: flash column chromatography (DCM/MeOH, gradient). Yield: 24%. ^1^H NMR (300 MHz, DMSO-*d*_6_): δ 14.02 (s, 1H), 12.31 (s, 1H), 8.78 (d, *J* = 2.1 Hz, 1H), 8.03 (d, *J* = 8.6 Hz, 1H), 7.25 (dd, *J* = 8.6, 2.2 Hz, 1H), 7.06 (t, *J* = 8.2 Hz, 1H), 6.38–6.21 (m, 3H), 4.69 (s, 2H), 3.31 (q, *J* = 7.0 Hz, 4H), 1.07 (t, *J* = 7.0 Hz, 6H). ^13^C NMR (75 MHz, DMSO-*d*_6_): δ 168.5, 168.1, 158.5, 148.8, 141.1, 138.4, 133.0, 129.9, 122.9, 118.8, 115.5, 105.5, 101.6, 98.3, 67.3, 43.7, 12.4. HRMS (ESI) *m*/*z*: [M – H]^−^ calculated for C_19_H_20_O_4_N_2_Cl 375.1117, found 375.1113. Purity by HPLC: peak area >97% (detection at 254 nm).

#### Methyl 4-Chloro-2-(2-(3-cyanophenoxy)­acetamido)­benzoate (**67**)

The reaction was carried out according to general procedure C using **7** (1.55 mmol) dissolved in DMF (13 mL), 3-cyanophenol (1.70 mmol), and K_2_CO_3_ (3.10 mmol). Reaction time: 20 min. Purification: flash column chromatography (cyclohexane/EtOAc, gradient). Yield: 79%. ^1^H NMR (300 MHz, DMSO-*d*_6_): δ 11.74 (s, 1H), 8.68 (d, *J* = 2.1 Hz, 1H), 8.02 (d, *J* = 8.6 Hz, 1H), 7.63–7.54 (m, 2H), 7.51 (dt, *J* = 7.8, 1.3 Hz, 1H), 7.46 (ddd, *J* = 8.3, 2.5, 1.3 Hz, 1H), 7.31 (dd, *J* = 8.6, 2.2 Hz, 1H), 4.87 (s, 2H), 3.91 (s, 3H). ^13^C NMR (75 MHz, DMSO-*d*_6_): δ 167.0, 157.8, 157.1, 140.5, 138.9, 132.6, 131.1, 125.8, 123.4, 122.8, 120.8, 119.4, 117.8, 114.8, 112.4, 67.5, 52.8. HRMS (ESI) *m*/*z*: [M + H]^+^ calculated for C_17_H_14_O_4_N_2_Cl 345.0637, found 345.0635.

#### 4-Chloro-2-(2-(3-cyanophenoxy)­acetamido)­benzoic Acid (**110**)

The reaction was carried out according to general procedure D using **67** (1.18 mmol) dissolved in MeOH (70 mL) and KOH (3.55 mmol) dissolved in H_2_O (14 mL). Reaction time: 30 min. Yield: 73%. ^1^H NMR (300 MHz, DMSO-*d*_6_): δ 14.09 (s, 1H), 12.22 (s, 1H), 8.75 (d, *J* = 2.1 Hz, 1H), 8.02 (d, *J* = 8.6 Hz, 1H), 7.61–7.52 (m, 2H), 7.49 (dt, *J* = 7.3, 1.3 Hz, 1H), 7.43 (ddd, *J* = 8.0, 2.7, 1.2 Hz, 1H), 7.26 (dd, *J* = 8.6, 2.2 Hz, 1H), 4.85 (s, 2H). ^13^C NMR (75 MHz, DMSO-*d*_6_): δ 168.7, 167.0, 157.1, 141.0, 138.7, 133.1, 131.1, 125.6, 123.1, 120.4, 118.8, 118.4, 118.1, 115.0, 112.3, 67.4. HRMS (ESI) *m*/*z*: [M – H]^−^ calculated for C_16_H_10_O_4_N_2_Cl 329.0335, found 329.0334. Purity by HPLC: peak area >97% (detection at 254 nm).

#### 4-Chloro-2-(2-(3-(methylcarbamoyl)­phenoxy)­acetamido)­benzoic Acid (**111**)

The reaction was carried out according to general procedure E using **7** (0.73 mmol) dissolved in DMF (2 mL), **39** (0.80 mmol), and K_2_CO_3_ (1.46 mmol) for the synthesis of methyl 4-chloro-2-(2-(3-(methylcarbamoyl)­phenoxy)­acetamido)­benzoate (**68**). Reaction time: 90 min. The reaction was then continued according to general procedure E by using **68** dissolved in MeOH (50 mL) and KOH (2.20 mmol) dissolved in H_2_O (10 mL) for the synthesis of **111**. Reaction time: 120 min. Purification: The product was precipitated twice. Yield: 62%. ^1^H NMR (300 MHz, DMSO-*d*_6_): δ 14.13 (s, 1H), 12.31 (s, 1H), 8.80 (d, *J* = 2.2 Hz, 1H), 8.45 (d, *J* = 4.8 Hz, 1H), 8.03 (d, *J* = 8.6 Hz, 1H), 7.58–7.36 (m, 3H), 7.34–7.16 (m, 2H), 4.81 (s, 2H), 2.78 (d, *J* = 4.5 Hz, 3H). ^13^C NMR (75 MHz, DMSO-*d*_6_): δ 168.9, 167.5, 166.1, 156.9, 141.2, 138.7, 136.2, 133.0, 129.6, 123.1, 120.3, 118.8, 117.4, 114.9, 113.5, 67.2, 26.3. HRMS (ESI) *m*/*z*: [M – H]^−^ calculated for C_17_H_14_O_5_N_2_Cl 361.0597, found 361.0593. Purity by HPLC: peak area >99% (detection at 254 nm).

#### Methyl 4-Chloro-2-(2-(3-(methoxycarbonyl)­phenoxy)­acetamido)­benzoate (**69**)

The reaction was carried out according to general procedure C using **7** (1.53 mmol) dissolved in DMF (2 mL), methyl 3-hydroxybenzoate (1.68 mmol), and K_2_CO_3_ (3.06 mmol). Reaction time: 60 min. Purification: flash column chromatography (cyclohexane/EtOAc, gradient). Yield: 77%. ^1^H NMR (300 MHz, CDCl_3_): δ 12.18 (s, 1H), 8.89 (d, *J* = 2.1 Hz, 1H), 7.98 (d, *J* = 8.6 Hz, 1H), 7.76–7.69 (m, 2H), 7.42 (t, *J* = 7.6 Hz, 1H), 7.32 (ddd, *J* = 8.3, 2.6, 1.1 Hz, 1H), 7.11 (dd, *J* = 8.6, 2.1 Hz, 1H), 4.69 (s, 2H), 3.94 (d, *J* = 7.3 Hz, 6H). ^13^C NMR (75 MHz, CDCl_3_): δ 167.7, 167.2, 166.7, 157.4, 141.3, 141.0, 132.1, 132.0, 129.9, 123.6, 123.6, 120.6, 120.4, 115.3, 68.5, 67.9, 52.7, 52.4. HRMS (ESI) *m*/*z*: [M – H]^−^ calculated for C_15_H_11_O_5_NCl 320.0331, found 320.0332.

#### 4-Chloro-2-(2-(3-carboxyphenoxy)­acetamido)­benzoic Acid (**112**)

The reaction was carried out according to general procedure D using **69** (1.03 mmol) dissolved in MeOH (50 mL) and KOH (5.31 mmol) dissolved in H_2_O (12 mL). Reaction time: 60 min. Yield: 58%. ^1^H NMR (300 MHz, DMSO-*d*_6_): δ 14.07 (s, 1H), 13.10 (s, 1H), 12.33 (s, 1H), 8.79 (d, *J* = 2.2 Hz, 1H), 8.03 (d, *J* = 8.6 Hz, 1H), 7.60 (dq, *J* = 4.9, 1.5 Hz, 2H), 7.48 (t, *J* = 8.1 Hz, 1H), 7.34 (ddd, *J* = 8.2, 2.7, 1.2 Hz, 1H), 7.27 (dd, *J* = 8.6, 2.2 Hz, 1H), 4.82 (s, 2H). ^13^C NMR (75 MHz, DMSO-*d*_6_): δ 168.9, 167.4, 166.9, 157.0, 141.2, 138.7, 133.0, 132.4, 130.0, 123.1, 122.6, 118.9, 118.8, 115.7, 114.9, 67.2. HRMS (ESI) *m*/*z*: [M – H]^−^ calculated for C_16_H_11_O_6_NCl 348.0280, found 348.0282. Purity by HPLC: peak area >99% (detection at 254 nm).

#### Methyl 4-Chloro-2-(2-(3-hydroxyphenoxy)­acetamido)­benzoate (**70**)

The reaction was carried out according to general procedure C using 7 (0.94 mmol) dissolved in DMF (10 mL), resorcinol (0.94 mmol), and K_2_CO_3_ (1.87 mmol). The mixture was stirred at rt instead of 80 °C. Reaction time: 16 h. Purification: flash column chromatography (cyclohexane/EtOAc, gradient) and (H_2_O + 0.1% TFA/ACN + 0.1% TFA, gradient). Yield: 22%. X-ray: Single crystals were obtained from H_2_O + 0.1% TFA/ACN + 0.1% TFA. ^1^H NMR (300 MHz, DMSO-*d*_6_): δ 11.77 (s, 1H), 9.55 (s, 1H), 8.73 (d, *J* = 2.1 Hz, 1H), 8.01 (d, *J* = 8.6 Hz, 1H), 7.30 (dd, *J* = 8.6, 2.2 Hz, 1H), 7.12 (t, *J* = 8.0 Hz, 1H), 6.60–6.37 (m, 3H), 4.68 (s, 2H), 3.91 (s, 3H). ^13^C NMR (75 MHz, DMSO-*d*_6_): δ 167.7, 166.8, 158.6, 158.1, 140.6, 138.9, 132.6, 130.1, 123.3, 119.3, 114.6, 109.1, 105.3, 102.4, 67.2, 52.8. HRMS (ESI) *m*/*z*: [M + H]^+^ calculated for C_16_H_15_O_5_NCl 336.0633, found 336.0633.

#### 4-Chloro-2-(2-(3-hydroxyphenoxy)­acetamido)­benzoic Acid (**113**)

The reaction was carried out according to general procedure D using 70 (0.25 mmol) dissolved in MeOH (50 mL) and KOH (0.51 mmol) dissolved in H_2_O (10 mL). Reaction time: 210 min. Yield: 79%. ^1^H NMR (300 MHz, DMSO-*d*_6_): δ 14.02 (s, 1H), 12.23 (s, 1H), 9.51 (s, 1H), 8.78 (d, *J* = 2.1 Hz, 1H), 8.02 (d, *J* = 8.6 Hz, 1H), 7.26 (dd, *J* = 8.6, 2.1 Hz, 1H), 7.10 (t, *J* = 8.1 Hz, 1H), 6.59–6.33 (m, 3H), 4.68 (s, 2H). ^13^C NMR (75 MHz, DMSO-*d*_6_): δ 168.7, 167.8, 158.6, 158.2, 141.2, 138.7, 133.0, 130.0, 123.0, 118.8, 114.9, 109.0, 105.4, 102.2, 67.1. HRMS (ESI) *m*/*z*: [M – H]^−^ calculated for C_15_H_11_O_5_NCl 320.0331, found 320.0332. Purity by HPLC: peak area >99% (detection at 254 nm).

#### Methyl 4-Chloro-2-(2-(2-methoxyphenoxy)­acetamido)­benzoate (**71**)

The reaction was carried out according to general procedure C using **7** (1.14 mmol), 2-methoxyphenol (0.16 mmol), and K_2_CO_3_ (2.29 mmol) dissolved in DMF (1 mL). Reaction time: 60 min. Purification: flash column chromatography (cyclohexane/EtOAc, gradient). Yield: 77%. ^1^H NMR (300 MHz, DMSO-*d*_6_): δ 11.64 (s, 1H), 8.73 (d, *J* = 2.2 Hz, 1H), 8.01 (d, *J* = 8.6 Hz, 1H), 7.30 (dd, *J* = 8.6, 2.2 Hz, 1H), 7.10–6.98 (m, 3H), 6.89 (ddd, *J* = 7.8, 7.1, 2.0 Hz, 1H), 4.70 (s, 2H), 3.86 (s, 3H), 3.83 (s, 3H). ^13^C NMR (75 MHz, DMSO-*d*_6_): δ 168.1, 166.5, 149.7, 146.7, 140.5, 138.8, 132.6, 123.3, 123.0, 120.6, 119.6, 115.7, 114.8, 112.7, 69.0, 55.6, 52.6.

#### 4-Chloro-2-(2-(2-methoxyphenoxy)­acetamido)­benzoic Acid (**114**)

The reaction was carried out according to general procedure D using **71** (0.86 mmol) dissolved in MeOH (55 mL) and KOH (2.58 mmol) dissolved in H_2_O (11 mL). Reaction time: 60 min. Purification: flash column chromatography (cyclohexane/EtOAc, gradient). Yield: 40%. ^1^H NMR (300 MHz, DMSO-*d*_6_): δ 13.87 (s, 1H), 12.04 (s, 1H), 8.77 (d, *J* = 2.1 Hz, 1H), 8.01 (d, *J* = 8.5 Hz, 1H), 7.27 (dd, *J* = 8.6, 2.2 Hz, 1H), 7.10–6.97 (m, 3H), 6.88 (ddd, *J* = 7.8, 7.0, 2.1 Hz, 1H), 4.70 (s, 2H), 3.81 (s, 3H). ^13^C NMR (75 MHz, DMSO-*d*_6_): δ 168.4, 168.3, 149.8, 146.9, 141.1, 138.5, 132.9, 123.1, 123.0, 120.7, 119.2, 116.0, 115.2, 112.9, 69.4, 55.8. HRMS (ESI) *m*/*z*: [M – H]^−^ calculated for C_16_H_13_O_5_NCl 334.0488, found 334.0491. Purity by HPLC: peak area >99% (detection at 254 nm).

#### Methyl 4-Chloro-2-(2-(3-methoxyphenoxy)­acetamido)­benzoate (**72**)

The reaction was carried out according to general procedure C using **7** (1.53 mmol), 3-methoxyphenol (1.69 mmol), and K_2_CO_3_ (3.07 mmol) dissolved in DMF (12 mL). Reaction time: 30 min. Purification: flash column chromatography (cyclohexane/EtOAc, gradient). Yield: 93%.

#### 4-Chloro-2-(2-(3-methoxyphenoxy)­acetamido)­benzoic Acid (**115**)

The reaction was carried out according to general procedure D using **72** (1.21 mmol) dissolved in MeOH (75 mL) and KOH (3.64 mmol) dissolved in H_2_O (16 mL). Reaction time: 60 min. Purification: recrystallization from EtOAc. Yield: 30%. ^1^H NMR (300 MHz, DMSO-*d*_6_): δ 14.04 (s, 1H), 12.22 (s, 1H), 8.78 (d, *J* = 2.2 Hz, 1H), 8.02 (d, *J* = 8.6 Hz, 1H), 7.30–7.18 (m, 2H), 6.71–6.56 (m, 3H), 4.74 (s, 2H), 3.75 (s, 3H). ^13^C NMR (75 MHz, DMSO-*d*_6_): δ 168.7, 167.7, 160.5, 158.2, 141.1, 138.7, 133.0, 130.2, 123.0, 118.8, 115.0, 107.2, 107.1, 101.3, 67.3, 55.2. HRMS (ESI) *m*/*z*: [M – H]^−^ calculated for C_16_H_13_O_5_NCl 334.0488, found 334.0490. Purity by HPLC: peak area >99% (detection at 254 nm).

#### Methyl 4-Chloro-2-(2-(4-methoxyphenoxy)­acetamido)­benzoate (**73**)

The reaction was carried out according to general procedure C using **7** (1.93 mmol), 4-methoxyphenol (2.12 mmol), and K_2_CO_3_ (3.93 mmol) dissolved in DMF (1 mL). Reaction time: 120 min. Purification: flash column chromatography (cyclohexane/EtOAc, gradient). Yield: 59%. HRMS (ESI) *m*/*z*: [M + H]^+^ calculated for C_17_H_17_O_5_NCl 350.0790, found 350.0804.

#### 4-Chloro-2-(2-(4-methoxyphenoxy)­acetamido)­benzoic Acid (**116**)

The reaction was carried out according to general procedure D using **73** (1.11 mmol) dissolved in MeOH (67 mL) and KOH (3.30 mmol) dissolved in H_2_O (13 mL). Reaction time: 120 min. Yield: 53%. ^1^H NMR (300 MHz, DMSO-*d*_6_): δ 13.96 (s, 1H), 12.24 (s, 1H), 8.79 (d, *J* = 2.2 Hz, 1H), 8.02 (d, *J* = 8.6 Hz, 1H), 7.26 (dd, *J* = 8.6, 2.2 Hz, 1H), 7.06–6.98 (m, 2H), 6.94–6.86 (m, 2H), 4.68 (s, 2H), 3.71 (s, 3H). ^13^C NMR (75 MHz, DMSO-*d*_6_): δ 168.7, 168.0, 154.1, 151.1, 141.2, 138.7, 133.0, 123.0, 118.8, 115.7, 114.9, 114.7, 67.8, 55.4. HRMS (ESI) *m*/*z*: [M – H]^−^ calculated for C_16_H_13_O_5_NCl 334.0488, found 334.0491. Purity by HPLC: peak area >99% (detection at 254 nm).

#### Methyl 4-Chloro-2-(2-(2-(prop-2-yn-1-yloxy)­phenoxy)­acetamido)­benzoate (**74**)

The reaction was carried out according to general procedure C using **7** (2.29 mmol), **24** (2.52 mmol), and K_2_CO_3_ (4.59 mmol) dissolved in DMF (2 mL). Reaction time: 60 min. Purification: flash column chromatography (cyclohexane/EtOAc, gradient). Yield: 59%. ^1^H NMR (300 MHz, DMSO-*d*_6_): δ 11.64 (s, 1H), 8.72 (d, *J* = 2.2 Hz, 1H), 8.00 (d, *J* = 8.6 Hz, 1H), 7.30 (dd, *J* = 8.6, 2.2 Hz, 1H), 7.13 (ddd, *J* = 9.3, 7.8, 1.8 Hz, 2H), 7.03 (td, *J* = 7.7, 1.8 Hz, 1H), 6.95 (td, *J* = 7.6, 1.7 Hz, 1H), 4.86 (d, *J* = 2.4 Hz, 2H), 4.73 (s, 2H), 3.87 (s, 3H), 3.53 (t, *J* = 2.4 Hz, 1H). ^13^C NMR (75 MHz, DMSO-*d*_6_): δ 167.9, 166.5, 147.5, 147.3, 140.5, 138.8, 132.5, 123.3, 122.7, 121.8, 119.6, 116.0, 115.1, 114.8, 79.3, 78.2, 69.1, 56.3, 52.7. HRMS (ESI) *m*/*z*: [M + H]^+^ calculated for C_19_H_17_O_5_NCl 374.0790, found 374.0790.

#### 4-Chloro-2-(2-(2-(prop-2-yn-1-yloxy)­phenoxy)­acetamido)­benzoic Acid (**117**)

The reaction was carried out according to general procedure D using **74** (1.30 mmol) dissolved in MeOH (50 mL) and KOH (3.92 mmol) dissolved in H_2_O (17 mL). Reaction time: 120 min. Yield: quant. ^1^H NMR (300 MHz, DMSO-*d*_6_): δ 12.91 (s, 1H), 8.72 (d, *J* = 2.2 Hz, 1H), 8.01 (d, *J* = 8.5 Hz, 1H), 7.20 (dd, *J* = 8.5, 2.2 Hz, 1H), 7.12 (dt, *J* = 7.6, 1.8 Hz, 2H), 6.97 (dtd, *J* = 18.1, 7.5, 1.8 Hz, 2H), 4.85 (d, *J* = 2.4 Hz, 2H), 4.70 (s, 2H), 3.54 (t, *J* = 2.4 Hz, 1H). ^13^C NMR (75 MHz, DMSO-*d*_6_): δ 168.3, 167.9, 147.8, 147.6, 141.1, 137.1, 132.9, 122.6, 122.0, 118.8, 117.0, 116.3, 115.6, 79.4, 78.3, 69.5, 56.6. HRMS (ESI) *m*/*z*: [M – H]^−^ calculated for C_18_H_13_O_5_NCl 358.0488, found 358.0491. Purity by HPLC: peak area >98% (detection at 254 nm).

#### Methyl 4-Chloro-2-(2-(3-(prop-2-yn-1-yloxy)­phenoxy)­acetamido)­benzoate (**75**)

The reaction was carried out according to general procedure C using **7** (2.06 mmol), **25** (2.34 mmol), and K_2_CO_3_ (4.13 mmol) dissolved in DMF (2 mL). Reaction time: 60 min. Purification: flash column chromatography (cyclohexane/EtOAc, gradient). Yield: 78%. X-ray: Single crystals were obtained from cyclohexane/EtOAc. HRMS (ESI) *m*/*z*: [M + H]^+^ calculated for C_19_H_17_O_5_NCl 374.0790, found 374.0783.

#### 4-Chloro-2-(2-(3-(prop-2-yn-1-yloxy)­phenoxy)­acetamido)­benzoic Acid (**118**)

The reaction was carried out according to general procedure D using **75** (1.34 mmol) dissolved in MeOH (50 mL) and KOH (4.02 mmol) dissolved in H_2_O (10 mL). Reaction time: 60 min. Yield: 96%. ^1^H NMR (300 MHz, DMSO-*d*_6_): δ 14.04 (s, 1H), 12.22 (s, 1H), 8.78 (d, *J* = 2.1 Hz, 1H), 8.03 (d, *J* = 8.6 Hz, 1H), 7.31–7.21 (m, 2H), 6.76–6.63 (m, 3H), 4.79 (d, *J* = 2.4 Hz, 2H), 4.74 (s, 2H), 3.57 (s, 1H). ^13^C NMR (75 MHz, DMSO-*d*_6_): δ 168.7, 167.6, 158.5, 158.1, 141.1, 138.7, 133.0, 130.2, 123.0, 118.8, 117.0, 115.0, 107.9, 102.4, 79.1, 78.3, 67.3, 55.6. HRMS (ESI) *m*/*z*: [M – H]^−^ calculated for C_18_H_13_O_5_NCl 358.0488, found 358.0485. Purity by HPLC: peak area >99% (detection at 254 nm).

#### Methyl 4-Chloro-2-(2-(4-(prop-2-yn-1-yloxy)­phenoxy)­acetamido)­benzoate (**76**)

The reaction was carried out according to general procedure C using **7** (2.37 mmol), **26** (2.73 mmol), and K_2_CO_3_ (4.82 mmol) dissolved in DMF (2 mL).

#### 4-Chloro-2-(2-(4-(prop-2-yn-1-yloxy)­phenoxy)­acetamido)­benzoic Acid (**119**)

The reaction was carried out according to general procedure D using **76** (2.37 mmol) dissolved in MeOH (90 mL) and KOH (7.18 mmol) dissolved in H_2_O (30 mL). Reaction time: 90 min. Yield: 71%. ^1^H NMR (300 MHz, DMSO-*d*_6_): δ 14.05 (s, 1H), 12.24 (s, 1H), 8.79 (d, *J* = 2.2 Hz, 1H), 8.02 (d, *J* = 8.5 Hz, 1H), 7.27 (dd, *J* = 8.6, 2.2 Hz, 1H), 7.12–6.89 (m, 4H), 4.74 (d, *J* = 2.4 Hz, 2H), 4.70 (s, 2H), 3.53 (t, *J* = 2.4 Hz, 1H). ^13^C NMR (75 MHz, DMSO-*d*_6_): δ 168.7, 167.9, 152.0, 151.6, 141.2, 138.7, 133.0, 123.0, 118.8, 116.0, 115.7, 114.9, 79.4, 78.1, 67.8, 55.9. HRMS (ESI) *m*/*z*: [M – H]^−^ calculated for C_18_H_13_O_5_NCl 358.0488, found 358.0493. Purity by HPLC: peak area >98% (detection at 254 nm).

#### Methyl 2-(2-(3-(But-2-yn-1-yloxy)­phenoxy)­acetamido)-4-chlorobenzoate (**77**)

The reaction was carried out according to general procedure C using **7** (1.60 mmol), **27** (1.76 mmol) and K_2_CO_3_ (3.20 mmol) dissolved in DMF (10 mL). Reaction time: 120 min. Purification: flash column chromatography (cyclohexane/EtOAc, gradient).

#### 2-(2-(3-(But-2-yn-1-yloxy)­phenoxy)­acetamido)-4-chlorobenzoic Acid (**120**)

The reaction was carried out according to general procedure D using **77** (1.60 mmol) dissolved in MeOH (50 mL) and KOH (4.80 mmol) dissolved in H_2_O (10 mL). Reaction time: 30 min. Purification: 2× flash column chromatography (cyclohexane/THF + 5% AcOH, gradient and DCM/MeOH, gradient). Yield: 50% (over two steps). ^1^H NMR (300 MHz, DMSO-*d*_6_): δ 14.03 (s, 1H), 12.23 (s, 1H), 8.78 (d, *J* = 2.2 Hz, 1H), 8.03 (d, *J* = 8.6 Hz, 1H), 7.28–7.22 (m, 2H), 6.75–6.60 (m, 3H), 4.80–4.68 (m, 4H), 1.82 (t, *J* = 2.3 Hz, 3H). ^13^C NMR (75 MHz, DMSO-*d*_6_): δ 168.7, 167.6, 158.7, 158.1, 141.1, 138.6, 133.1, 130.1, 123.0, 118.8, 115.0, 107.8, 107.7, 102.3, 83.6, 74.6, 67.3, 56.0, 3.1. HRMS (ESI) *m*/*z*: [M – H]^−^ calculated for C_19_H_15_O_5_NCl 372.0644, found 372.0631. Purity by HPLC: peak area >97% (detection at 254 nm).

#### Methyl 4-Chloro-2-(2-(2-methyl-3-(prop-2-yn-1-yloxy)­phenoxy)­acetamido)­benzoate (**78**)

The reaction was carried out according to general procedure C using **7** (0.80 mmol), **29** (0.88 mmol), and K_2_CO_3_ (1.60 mmol) dissolved in DMF (10 mL). Reaction time: 90 min. Purification: flash column chromatography (cyclohexane/EtOAc, gradient). Yield: 35%. ^1^H NMR (300 MHz, DMSO-*d*_6_): δ 11.64 (s, 1H), 8.73 (d, *J* = 2.1 Hz, 1H), 8.00 (d, *J* = 8.6 Hz, 1H), 7.30 (dd, *J* = 8.6, 2.2 Hz, 1H), 7.14 (t, *J* = 8.3 Hz, 1H), 6.76 (d, *J* = 8.3 Hz, 1H), 6.69 (d, *J* = 8.3 Hz, 1H), 4.81 (d, *J* = 2.4 Hz, 2H), 4.75 (s, 2H), 3.85 (s, 3H), 3.55 (t, *J* = 2.4 Hz, 1H), 2.23 (s, 3H). ^13^C NMR (75 MHz, DMSO-*d*_6_): δ 167.8, 166.8, 156.1, 155.7, 140.6, 138.9, 132.5, 126.5, 123.3, 119.5, 114.7, 114.6, 106.4, 105.6, 79.5, 78.1, 67.8, 56.0, 52.7, 8.4. HRMS (ESI) *m*/*z*: [M + H]^+^ calculated for C_20_H_19_O_5_NCl 388.0946, found 388.0959.

#### 4-Chloro-2-(2-(2-methyl-3-(prop-2-yn-1-yloxy)­phenoxy)­acetamido)­benzoic Acid (**121**)

The reaction was carried out according to general procedure D using **78** (0.23 mmol) dissolved in MeOH (60 mL) and KOH (0.68 mmol) dissolved in H_2_O (14 mL). Reaction time: 60 min. Yield: 97%. ^1^H NMR (300 MHz, DMSO-*d*_6_): δ 13.94 (s, 1H), 11.99 (s, 1H), 8.79 (d, *J* = 2.1 Hz, 1H), 8.01 (d, *J* = 8.6 Hz, 1H), 7.27 (dd, *J* = 8.6, 2.2 Hz, 1H), 7.13 (t, *J* = 8.3 Hz, 1H), 6.75 (d, *J* = 8.3 Hz, 1H), 6.67 (d, *J* = 8.3 Hz, 1H), 4.81 (d, *J* = 2.3 Hz, 2H), 4.75 (s, 2H), 3.55 (t, *J* = 2.3 Hz, 1H), 2.21 (s, 3H). ^13^C NMR (75 MHz, DMSO-*d*_6_): δ 168.6, 167.9, 156.0, 155.8, 141.1, 138.6, 133.0, 126.5, 123.1, 119.2, 115.1, 114.5, 106.4, 105.4, 79.5, 78.1, 67.9, 56.0, 8.5. HRMS (ESI) *m*/*z*: [M – H]^−^ calculated for C_19_H_15_O_5_NCl 372.0644, found 372.0641. Purity by HPLC: peak area >97% (detection at 254 nm).

#### Methyl 4-Chloro-2-(2-(3-isobutoxyphenoxy)­acetamido)­benzoate (**79**)

The reaction was carried out according to general procedure C using **7** (0.75 mmol), **36** (0.83 mmol), and K_2_CO_3_ (1.51 mmol) dissolved in DMF (10 mL). Reaction time: 110 min. Purification: flash column chromatography (cyclohexane/EtOAc, gradient). Yield: not determined. ^1^H NMR (300 MHz, DMSO-*d*_6_): δ 11.81 (s, 1zH), 8.72 (d, *J* = 2.1 Hz, 1H), 8.01 (d, *J* = 8.6 Hz, 1H), 7.30 (dd, *J* = 8.6, 2.2 Hz, 1H), 7.27–7.19 (m, 1H), 6.69–6.65 (m, 2H), 6.63–6.56 (m, 1H), 4.74 (s, 2H), 3.90 (s, 3H), 3.75 (d, *J* = 6.6 Hz, 2H), 2.01 (n, *J* = 6.6 Hz, 1H), 0.97 (d, *J* = 6.7 Hz, 6H). ^13^C NMR (75 MHz, DMSO-*d*_6_): δ 168.1, 167.3, 160.5, 158.7, 141.1, 139.4, 133.1, 130.6, 123.8, 119.7, 115.0, 108.6, 107.6, 102.2, 74.3, 67.8, 53.2, 28.1, 19.5. HRMS (ESI) *m*/*z*: [M + H]^+^ calculated for C_20_H_23_O_5_NCl 392.1259, found 392.1247.

#### 4-Chloro-2-(2-(3-isobutoxyphenoxy)­acetamido)­benzoic Acid (**122**)

The reaction was carried out according to general procedure D using **79** (0.16 mmol) dissolved in MeOH (50 mL) and KOH (0.16 mmol) dissolved in H_2_O (10 mL). Reaction time: 5 h. Purification: flash column chromatography (DCM/MeOH, gradient) Yield: 77%. ^1^H NMR (300 MHz, DMSO-*d*_6_): δ 14.18 (s, 1H), 12.59 (s, 1H), 8.77 (d, *J* = 2.1 Hz, 1H), 8.03 (d, *J* = 8.5 Hz, 1H), 7.29–7.14 (m, 2H), 6.75–6.49 (m, 3H), 4.72 (s, 2H), 3.73 (d, *J* = 6.5 Hz, 2H), 2.00 (n, *J* = 6.6 Hz, 1H), 0.97 (d, *J* = 6.7 Hz, 6H). ^13^C NMR (75 MHz, DMSO-*d*_6_): δ 171.0, 168.6, 167.6, 160.0, 158.2, 141.1, 138.0, 133.0, 130.1, 122.8, 118.7, 107.6, 107.2, 101.8, 73.8, 67.3, 27.7, 19.1. HRMS (ESI) *m*/*z*: [M – H]^−^ calculated for C_19_H_19_O_5_NCl 376.0957, found 376.0950. Purity by HPLC: peak area >97% (detection at 254 nm).

#### Methyl 4-Chloro-2-(2-(3-ethynylphenoxy)­acetamido)­benzoate (**80**)

The reaction was carried out according to general procedure C using **7** (1.28 mmol), 3-ethynylphenol (1.41 mmol), and K_2_CO_3_ (2.56 mmol) dissolved in DMF (5 mL). Reaction time: 60 min. Purification: flash column chromatography (pentane/Et_2_O, gradient). Yield: 71%. ^1^H NMR (300 MHz, DMSO-*d*_6_): δ 11.78 (s, 1H), 8.70 (d, *J* = 2.1 Hz, 1H), 8.00 (d, *J* = 8.6 Hz, 1H), 7.38 (t, *J* = 7.9 Hz, 1H), 7.29 (dd, *J* = 8.6, 2.2 Hz, 1H), 7.24–7.05 (m, 3H), 4.79 (s, 2H), 4.23 (s, 1H), 3.90 (s, 3H). ^13^C NMR (75 MHz, DMSO-*d*_6_): δ 167.3, 166.9, 156.8, 140.6, 139.0, 132.5, 130.1, 125.3, 123.3, 123.0, 119.3, 117.7, 116.2, 114.6, 83.1, 81.1, 67.3, 52.8. HRMS (ESI) *m*/*z*: [M + H]^+^ calculated for C_18_H_15_O_4_NCl 344.0684, found 344.0681.

#### 4-Chloro-2-(2-(3-ethynylphenoxy)­acetamido)­benzoic Acid (**123**)

The reaction was carried out according to general procedure D using **80** (0.86 mmol) dissolved in MeOH (70 mL) and KOH (1.73 mmol) dissolved in H_2_O (14 mL). Reaction time: 60 min. Purification: The product was recrystallized from ACN. Yield: 95%. ^1^H NMR (300 MHz, DMSO-*d*_6_): δ 14.07 (s, 1H), 12.28 (s, 1H), 8.78 (d, *J* = 2.1 Hz, 1H), 8.02 (d, *J* = 8.6 Hz, 1H), 7.43–7.32 (m, 1H), 7.26 (dd, *J* = 8.6, 2.2 Hz, 1H), 7.22–7.06 (m, 3H), 4.78 (s, 2H), 4.23 (s, 1H). ^13^C NMR (75 MHz, DMSO-*d*_6_): δ 168.8, 167.4, 156.8, 141.2, 138.7, 133.0, 130.1, 125.2, 123.0, 122.9, 118.8, 118.0, 115.7, 114.9, 83.1, 81.1, 67.2. HRMS (ESI) *m*/*z*: [M – H]^−^ calculated for C_17_H_11_O_4_NCl 328.0382, found 328.0377. Purity by HPLC: peak area >99% (detection at 254 nm).

#### 4-Chloro-2-(2-(*m*-tolyloxy)­acetamido)­benzoic Acid (**124**)

The reaction was carried out according to general procedure E using **7** (0.78 mmol), *m*-cresol (0.85 mmol), and K_2_CO_3_ (1.60 mmol) dissolved in DMF (2 mL) to obtain methyl 4-chloro-2-(2-(*m*-tolyloxy)­acetamido)­benzoate (**81**). Reaction time: 120 min. The reaction was further carried out according to general procedure E by using **81** dissolved in MeOH (75 mL) and KOH (2.33 mmol) dissolved in H_2_O (10 mL). Reaction time: 4 h. Purification: flash column chromatography (DCM/MeOH, gradient). Yield: 62% (over two steps). ^1^H NMR (300 MHz, DMSO-*d*_6_): δ 14.03 (s, 1H), 12.24 (s, 1H), 8.79 (d, *J* = 2.2 Hz, 1H), 8.02 (d, *J* = 8.5 Hz, 1H), 7.29–7.17 (m, 2H), 6.94–6.78 (m, 3H), 4.71 (s, 2H), 2.29 (s, 3H). ^13^C NMR (75 MHz, DMSO-*d*_6_): δ 168.7, 167.7, 157.0, 141.2, 139.2, 138.7, 133.0, 129.4, 123.0, 122.4, 118.8, 115.4, 114.9, 111.7, 67.1, 21.1. HRMS (ESI) *m*/*z*: [M – H]^−^ calculated for C_16_H_13_O_4_NCl 318.0528, found 318.0543. Purity by HPLC: peak area >99% (detection at 254 nm).

#### Methyl 4-Chloro-2-(2-(3-ethylphenoxy)­acetamido)­benzoate (**82**)

The reaction was carried out according to general procedure C using **7** (1.59 mmol), 3-ethylphenol (1.74 mmol) and K_2_CO_3_ (3.17 mmol) dissolved in DMF (5 mL). Reaction time: 60 min. Purification: 2× flash column chromatography (cyclohexane/EtOAc, gradient and DCM/MeOH, gradient). Yield: 51%. ^1^H NMR (300 MHz, DMSO-*d*_6_): δ 11.81 (s, 1H), 8.73 (d, *J* = 2.1 Hz, 1H), 8.01 (d, *J* = 8.6 Hz, 1H), 7.34–7.21 (m, 2H), 6.99–6.95 (m, 1H), 6.94–6.84 (m, 2H), 4.74 (s, 2H), 3.90 (s, 3H), 2.60 (q, *J* = 7.6 Hz, 2H), 1.19 (t, *J* = 7.6 Hz, 3H). ^13^C NMR (75 MHz, DMSO-*d*_6_): δ 167.7, 166.8, 157.1, 145.6, 140.7, 138.9, 132.6, 129.5, 123.3, 121.3, 119.3, 114.6, 114.4, 112.1, 67.2, 52.7, 28.1, 15.4. HRMS (ESI) *m*/*z*: [M + H]^+^ calculated for C_18_H_18_O_4_NCl 348.0997, found 348.0991.

#### 4-Chloro-2-(2-(3-ethylphenoxy)­acetamido)­benzoic Acid (**125**)

The reaction was carried out according to general procedure D using **82** (0.78 mmol) dissolved in MeOH (50 mL) and KOH (1.56 mmol) dissolved in H_2_O (10 mL). Reaction time: 60 min. Purification: The product was recrystallized from ACN. Yield: 96%. ^1^H NMR (300 MHz, DMSO-*d*_6_): δ 14.03 (s, 1H), 12.24 (s, 1H), 8.79 (d, *J* = 2.1 Hz, 1H), 8.02 (d, *J* = 8.6 Hz, 1H), 7.32–7.18 (m, 2H), 6.99–6.79 (m, 3H), 4.72 (s, 2H), 2.58 (q, *J* = 7.6 Hz, 2H), 1.17 (t, *J* = 7.6 Hz, 3H). ^13^C NMR (75 MHz, DMSO-*d*_6_): δ 168.7, 167.8, 157.1, 145.6, 141.2, 138.7, 133.0, 129.4, 123.0, 121.2, 118.8, 115.0, 114.4, 112.0, 67.1, 28.2, 15.5. HRMS (ESI) *m*/*z*: [M + H]^+^ calculated for C_17_H_17_O_4_NCl 334.0841, found 334.0845. Purity by HPLC: peak area >98% (detection at 254 nm).

#### Methyl 4-Chloro-2-(2-(3-propylphenoxy)­acetamido)­benzoate (**83**)

The reaction was carried out according to general procedure C using **7** (1.61 mmol), 3-propylphenol (1.77 mmol), and K_2_CO_3_ (3.21 mmol) dissolved in DMF (4 mL). Reaction time: 60 min. Purification: 2× flash column chromatography (2x cyclohexane/EtOAc, gradient). Yield: 35%. ^1^H NMR (300 MHz, DMSO-*d*_6_): δ 11.80 (s, 1H), 8.72 (d, *J* = 2.1 Hz, 1H), 8.01 (d, *J* = 8.6 Hz, 1H), 7.32–7.20 (m, 2H), 6.97–6.82 (m, 3H), 4.73 (s, 2H), 3.89 (s, 3H), 2.60–2.52 (m, 2H), 1.59 (h, *J* = 7.3 Hz, 2H), 0.89 (t, *J* = 7.3 Hz, 3H). ^13^C NMR (75 MHz, DMSO-*d*_6_): δ 167.7, 166.8, 157.0, 144.0, 140.6, 138.9, 132.6, 129.4, 123.3, 121.9, 119.3, 114.9, 114.5, 112.1, 67.2, 52.7, 37.2, 23.9, 13.6. HRMS (ESI) *m*/*z*: [M + H]^+^ calculated for C_19_H_21_O_4_NCl 362.1154, found 362.1154.

#### 4-Chloro-2-(2-(3-propylphenoxy)­acetamido)­benzoic Acid (**126**)

The reaction was carried out according to general procedure D using **83** (0.52 mmol) dissolved in MeOH (50 mL) and KOH (1.05 mmol) dissolved in H_2_O (10 mL). Reaction time: 60 min. Purification: The product was recrystallized from ACN. Yield: 95%. ^1^H NMR (300 MHz, DMSO-*d*_6_): δ 14.03 (s, 1H), 12.23 (s, 1H), 8.79 (d, *J* = 2.1 Hz, 1H), 8.03 (d, *J* = 8.6 Hz, 1H), 7.31–7.16 (m, 2H), 6.99–6.73 (m, 3H), 4.72 (s, 2H), 2.55–2.53 (m, 2H), 1.58 (h, *J* = 7.3 Hz, 2H), 0.88 (t, *J* = 7.3 Hz, 3H). ^13^C NMR (75 MHz, DMSO-*d*_6_): δ 168.6, 167.8, 157.0, 144.0, 141.1, 138.6, 133.0, 129.3, 123.0, 121.8, 118.8, 115.0, 114.9, 112.0, 67.1, 37.3, 24.0, 13.6. HRMS (ESI) *m*/*z*: [M – H]^−^ calculated for C_18_H_17_O_4_NCl 346.0852, found 346.0847. Purity by HPLC: peak area >99% (detection at 254 nm).

#### Methyl 4-Chloro-2-(2-(3-isopropylphenoxy)­acetamido)­benzoate (**84**)

The reaction was carried out according to general procedure C using **7** (1.55 mmol), 3-isopropylphenol (1.71 mmol), and K_2_CO_3_ (3.11 mmol) dissolved in DMF (4 mL). Reaction time: 60 min. Purification: flash column chromatography (cyclohexane/EtOAc, gradient). Yield: 37%. ^1^H NMR (300 MHz, DMSO-*d*_6_): δ 11.83 (s, 1H), 8.73 (d, *J* = 2.1 Hz, 1H), 8.01 (d, *J* = 8.6 Hz, 1H), 7.36–7.19 (m, 2H), 7.04–6.98 (m, 1H), 6.92–6.89 (m, 2H), 4.75 (s, 2H), 3.90 (s, 3H), 2.88 (hept, *J* = 6.8 Hz, 1H), 1.21 (d, *J* = 6.9 Hz, 6H). ^13^C NMR (75 MHz, DMSO-*d*_6_): δ 167.8, 166.8, 157.1, 150.3, 140.7, 138.9, 132.6, 129.5, 123.3, 119.9, 119.3, 114.5, 113.2, 112.1, 67.3, 52.7, 33.4, 23.7. HRMS (ESI) *m*/*z*: [M + H]^+^ calculated for C_19_H_21_O_4_NCl 362.1154, found 362.1157.

#### 4-Chloro-2-(2-(3-isopropylphenoxy)­acetamido)­benzoic Acid (**127**)

The reaction was carried out according to general procedure D using **84** (0.55 mmol) dissolved in MeOH (50 mL) and KOH (1.09 mmol) dissolved in H_2_O (10 mL). Reaction time: 60 min. Purification: The product was recrystallized from ACN. Yield: 96%. X-ray: Single crystals were obtained from ACN. ^1^H NMR (300 MHz, DMSO-*d*_6_): δ 14.02 (s, 1H), 12.22 (s, 1H), 8.79 (d, *J* = 2.1 Hz, 1H), 8.03 (d, *J* = 8.6 Hz, 1H), 7.29–7.20 (m, 2H), 6.98–6.85 (m, 3H), 4.73 (s, 2H), 2.86 (hept, *J* = 6.9 Hz, 1H), 1.20 (d, *J* = 6.9 Hz, 6H). ^13^C NMR (75 MHz, DMSO-*d*_6_): δ 168.6, 167.8, 157.1, 150.3, 141.1, 138.6, 133.0, 129.4, 123.0, 119.7, 118.9, 115.0, 113.1, 112.1, 67.2, 33.5, 23.8. HRMS (ESI) *m*/*z*: [M – H]^−^ calculated for C_18_H_17_O_4_NCl 346.0852, found 346.0848. Purity by HPLC: peak area >99% (detection at 254 nm).

#### Methyl 2-(2-(3-(*tert*-Butyl)­phenoxy)­acetamido)-4-chlorobenzoate (**85**)

The reaction was carried out according to general procedure C using **7** (1.58 mmol), 3-*tert*-butylphenol (1.74 mmol), and K_2_CO_3_ (3.16 mmol) dissolved in DMF (4 mL). Reaction time: 60 min. Purification: flash column chromatography (cyclohexane/EtOAc, gradient). Yield: 42%. ^1^H NMR (300 MHz, DMSO-*d*_6_): δ 11.87 (s, 1H), 8.74 (d, *J* = 2.1 Hz, 1H), 8.02 (d, *J* = 8.6 Hz, 1H), 7.34–7.23 (m, 2H), 7.20–7.14 (m, 1H), 7.06 (ddd, *J* = 7.9, 1.9, 1.0 Hz, 1H), 6.90 (ddd, *J* = 8.2, 2.6, 0.9 Hz, 1H), 4.76 (s, 2H), 3.90 (s, 3H), 1.29 (s, 9H). ^13^C NMR (75 MHz, DMSO-*d*_6_): δ 167.8, 166.8, 156.9, 152.6, 140.7, 139.0, 132.6, 129.2, 123.3, 119.2, 118.8, 114.5, 112.7, 111.4, 67.3, 52.6, 34.5, 31.0. HRMS (ESI) *m*/*z*: [M + H]^+^ calculated for C_20_H_23_O_4_NCl 376.1310, found 376.1309.

#### 2-(2-(3-(*tert*-Butyl)­phenoxy)­acetamido)-4-chlorobenzoic Acid (**128**)

The reaction was carried out according to general procedure D using **85** (0.65 mmol) dissolved in MeOH (50 mL) and KOH (1.29 mmol) dissolved in H_2_O (10 mL). Reaction time: 30 min. Purification: The product was recrystallized from ACN. Yield: 96%. X-ray: Single crystals were obtained from ACN. ^1^H NMR (300 MHz, DMSO-*d*_6_): δ 14.00 (s, 1H), 12.21 (s, 1H), 8.79 (d, *J* = 2.1 Hz, 1H), 8.03 (d, *J* = 8.6 Hz, 1H), 7.31–7.21 (m, 2H), 7.12–7.07 (m, 1H), 7.07–7.01 (m, 1H), 6.90 (ddd, *J* = 8.1, 2.6, 0.8 Hz, 1H), 4.75 (s, 2H), 1.28 (s, 9H). ^13^C NMR (75 MHz, DMSO-*d*_6_): δ 168.5, 167.8, 156.9, 152.7, 141.1, 138.6, 133.1, 129.1, 123.0, 118.9, 118.7, 115.1, 112.3, 111.7, 67.3, 34.5, 31.1. HRMS (ESI) *m*/*z*: [M – H]^−^ calculated for C_19_H_19_O_4_NCl 360.1008, found 360.1005. Purity by HPLC: peak area >99% (detection at 254 nm).

#### Methyl 2-(2-Chloroacetamido)-4-iodobenzoate (**129**)

The reaction was carried out according to general procedure A using methyl 2-amino-4-iodobenzoate (3.61 mmol), chloroacetyl chloride (3.61 mmol), and K_2_CO_3_ (7.22 mmol) dissolved in THF (25 mL). Reaction time: 90 min. Purification: flash column chromatography (cyclohexane/EtOAc, gradient). Yield: 98%. ^1^H NMR (300 MHz, DMSO-*d*_6_) δ 11.31 (s, 1H), 8.82 (d, *J* = 1.6 Hz, 1H), 7.71 (d, *J* = 8.4 Hz, 1H), 7.63 (dd, *J* = 8.4, 1.7 Hz, 1H), 4.46 (s, 2H), 3.88 (s, 3H). ^13^C NMR (75 MHz, DMSO-*d*_6_) δ 167.1, 165.5, 139.7, 132.6, 132.1, 128.9, 116.4, 102.3, 52.8, 43.3. HRMS (ESI) *m*/*z*: [M + H]^+^ calculated for C_10_H_10_O_3_NClI 353.9388, found 353.9384.

#### Methyl 4-Iodo-2-(2-(naphthalen-1-yloxy)­acetamido)­benzoate (**130**)

The reaction was carried out according to general procedure C using **129** (1.44 mmol), 1-naphtol (1.58 mmol), and K_2_CO_3_ (2.88 mmol) dissolved in DMF (10 mL). Reaction time: 4 h. Purification: flash column chromatography (cyclohexane/EtOAc, gradient). Yield: 49%. ^1^H NMR (300 MHz, DMSO-*d*_6_) δ 11.78 (s, 1H), 9.13 (d, *J* = 1.7 Hz, 1H), 8.76–8.65 (m, 1H), 7.96–7.87 (m, 1H), 7.76 (d, *J* = 8.4 Hz, 1H), 7.68–7.52 (m, 4H), 7.44 (t, *J* = 7.9 Hz, 1H), 7.08 (dd, *J* = 7.7, 1.0 Hz, 1H), 4.94 (s, 2H), 3.89 (s, 3H). ^13^C NMR (75 MHz, DMSO-*d*_6_) δ 167.4, 167.2, 152.5, 140.2, 134.1, 132.3, 132.0, 128.6, 127.4, 126.7, 126.0, 125.5, 124.6, 122.2, 121.3, 115.4, 106.2, 102.7, 67.6, 52.7. HRMS (ESI) *m*/*z*: [M + H]^+^ calculated for C_20_H_17_O_4_NI 462.0197, found 462.0190. Purity by HPLC: peak area >99% (detection at 254 nm).

#### 4-Iodo-2-(2-(naphthalen-1-yloxy)­acetamido)­benzoic Acid (**131**)

The reaction was carried out according to general procedure D using **130** (0.32 mmol) dissolved in MeOH (50 mL) and KOH (0.97 mmol) dissolved in H_2_O (10 mL). Reaction time: 3 h. Purification: flash column chromatography (DCM/MeOH, gradient). Yield: 94%. ^1^H NMR (300 MHz, DMSO-*d*_6_) δ 13.92 (s, 1H), 12.15 (s, 1H), 9.17 (d, *J* = 1.7 Hz, 1H), 8.65–8.52 (m, 1H), 7.90 (dt, *J* = 7.8, 2.5 Hz, 1H), 7.76 (d, *J* = 8.3 Hz, 1H), 7.65–7.50 (m, 4H), 7.43 (t, *J* = 7.9 Hz, 1H), 7.12–6.99 (m, 1H), 4.95 (s, 2H). ^13^C NMR (75 MHz, DMSO-*d*_6_) δ 169.2, 167.4, 152.6, 140.8, 134.1, 132.6, 132.0, 128.3, 127.4, 126.6, 126.0, 125.6, 124.7, 121.9, 121.2, 115.8, 106.1, 102.3, 67.8. HRMS (ESI) *m*/*z*: [M – H]^−^ calculated for C_19_H_13_O_4_NI 445.9895, found 445.9889. Purity by HPLC: peak area >98% (detection at 254 nm).

### Determination of Aqueous Solubility

Solubility of compounds in PBS (pH = 7.4) containing phosphate buffer components (10 mM), NaCl (138 mM), and KCl (2.7 mM) with 1% v/v DMSO at 21 °C was measured. For this, a saturated compound solution in DMSO was centrifuged at 19,776*g* for 2 min to spin down the solid compound parts. An aliquot of the saturated DMSO supernatant was then added to the PBS buffer to reach 1% v/v DMSO in PBS, resulting in an immediate precipitation of the compound. The suspension was then vigorously shaken for 15 min, sonicated for 5 min, and once more vigorously shaken for 15 min at 21 °C. This suspension was then centrifuged at 19,776*g* for 2 min at 21 °C, and an aliquot of the supernatant was taken, filtered through a polypropylene syringe filter (0.22 μm), and measured three times in the HPLC. This process was repeated three times for each compound (*n* = 3 × 3 = 9). A calibration line for each compound was measured by dissolving a 4 mM DMSO stock solution in PBS to reach 1% v/v DMSO in PBS and a compound concentration of 40 μM. Three dilution series were then generated with 40, 20, 10, and 5 μM concentrations (1% v/v DMSO). Aliquots of these solutions were taken, filtered through a polypropylene syringe filter (0.22 μm), and measured by HPLC three times (*n* = 3 × 3 = 9). Simple linear regression calculated by GraphPad Prism gave the slope of these calibration lines, which allowed calculation of the compound concentrations from the measurements described above using the following formula:

$$c=\frac{\left(\mathrm{mAU}\right)}{m}$$

where *c* is the concentration, mAU is the area of HPLC absorption peaks, and *m* is the slope of the calibration line.

### Determination of Distribution Coefficient log *D*_7.4_

The distribution coefficient (log *D*_7.4_) was measured using the shake-flask method. PBS and *n*-octanol were combined, vigorously shaken in a separating funnel, and later left to separate for 72 h to generate PBS saturated with *n*-octanol and *n*-octanol saturated with PBS. Three independent 2-fold dilution series of compounds in the range of 40–5 μM (+1% DMSO) were prepared in PBS buffer (sat. with *n*-octanol) and *n*-octanol (sat. with PBS) for the measurement of calibration lines. UV–vis absorption spectra of the samples in both solvents were recorded from 200 to 800 nm using a Cary 60 UV–vis from Agilent Technologies to identify the absorption maxima (λ_max_) for each compound in each solvent. Absolute absorbance values at a fixed wavelength (λ_max_) were then measured for each compound concentration (40, 20, 10, and 5 μM) in PBS (sat. with *n*-octanol) or *n*-octanol (sat. with PBS) and a simple linear regression of calibration lines were calculated using the GraphPad Prism software (Figure S18). Compound stock solutions (4 mM in DMSO) were diluted 100-fold in PBS (sat. with *n*-octanol) and *n*-octanol (sat. with PBS). The two phases were then combined, vigorously shaken for 1 h, and later left to separate for 24 h. The two phases were then collected and filtered through a Nylon-66 syringe filter (0.22 μm), and absorbance of the samples was measured at the same fixed wavelengths (λ_max_) used for the calibration line. This process was repeated three times and each sample itself was measured three times (*n* = 3 × 3 = 9). Compound concentrations were then calculated using the following formula:

$$c=\frac{\left(A-b\right)}{m}$$

where *c* is the concentration, *A* is the absorbance of the sample at λ_max_, *b* is the *y* intercept of the calibration line, and *m* is the slope of the calibration line. The distribution coefficient log *D*_7.4_ was then calculated using the formula:

$$\log D_{7.4}=\log_{10}\left(\frac{c{®}_{\mathrm{octanol}}}{c{®}_{\mathrm{PBS}}}\right)$$

where *c̅*_octanol_ is the mean concentration of compound in the *n*-octanol (sat. with PBS) phase and *c̅*_PBS_ is the mean concentration of compound in the PBS (sat. with *n*-octanol) phase.

### Cell Culture

HEK293 or tsA201 cells were cultured in Dulbecco’s modified Eagle’s medium (DMEM) containing additional 10% of fetal bovine serum (FBS) at a stable temperature of 37 °C, saturated air humidity and a stable CO_2_ concentration of 5% in the air. Accutase was used as the cell detachment agent and PBS buffer w/o Mg^2+^/Ca^2+^ as the washing solution. HEK293 cells, stably overexpressing hTRPM4, were cultured with additional 140 μg/mL hygromycin B from *Streptomyces hygroscopicus*. HEK293 cells, stably overexpressing hTRPM5 and tsA201 cells, stably overexpressing mTRPM4 were cultured with additional 200 μg/mL zeocin. HEK293 wild-type cells (ECACC) were cultured without any selection antibiotic. The human colorectal carcinoma cell line HCT116 (ATCC) was cultured in McCoy’s 5A (Modified) medium with GlutaMAX (Gibco), supplemented with 10% heat-inactivated fetal bovine serum (FBS; Sigma). Cells were maintained at 37 °C in a humidified atmosphere with 5% CO_2_.

### Na^+^ Influx Assay

An assay procedure, which has been implemented in previous works conducted at our institute,^23^ was adopted and adjusted for this study. HEK293 cells, stably overexpressing hTRPM4 or hTRPM5, tsA201 cells, stably overexpressing mTRPM4, or HEK293 wild type cells in the cell culture medium were seeded in each well (80,000 cells/well) of Costar 96-well tissue culture-treated black plates with clear flat bottoms and with low evaporation lids (Corning 3603), which had been coated with poly-d-lysine hydrobromide beforehand. The cell plates were then incubated in the cell culture conditions described above for 16–24 h until satisfactory confluency of the cells (ca. 95%) was observed. The cells were then incubated twice for 30 min at 37 °C in a sodium free buffer (pH = 7.2, 100 μL/well) containing KCl (10 mM), CaCl_2_ (1 mM), MgCl_2_ (1 mM), *N*-methyl-d-glucamine (140 mM), HEPES (10 mM), and d-glucose (10 mM) for sodium depletion. The sodium-free buffer was then replaced by a dye loading solution containing the sodium-sensitive dye Ion NaTRIUM Green-2 AM (2.5 μg/mL), 0.1% w/v Pluronic F-127, and 1% v/v DMSO in the sodium-free buffer (95 μL/well), and the cell plate was incubated for 45 min at 30 °C in the dark. The dye loading solution was then replaced by a fresh sodium-free buffer containing 1% v/v DMSO (60 μL/well) for the measurement. TRPM4 activity measurements were conducted using a FLIPR Tetra High Throughput Screening System from Molecular Devices. Ion NaTRIUM Green-2 AM was excited at wavelengths of 470–495 nm, and emissions were recorded at wavelengths of 515–575 nm. 50 s after the start of the measurement, sodium-free buffer containing the compounds to be measured with 1% v/v DMSO was added to each well (20 μL/well) by the FLIPR and the cells were incubated for 250 s with the corresponding compounds at concentrations of 10 and 5 μM for activity screenings and in an 2-fold dilution series from 10–0.078125 μM (+ 0 μM) for the measurement of IC_50_ values. Then, a TRPM4 stimulus buffer (pH = 7.2) containing NaCl (700 mM), CaCl_2_ (4 mM), MgCl_2_ (4 mM), HEPES (40 mM), and 1% DMSO was added to each well by the FLIPR (20 μL/well) and the response signals were recorded for a further 270 s. The corresponding compounds at the appropriate concentrations were included in the stimulus buffer as well to maintain the final compound concentration of the compounds in the cell plate. Ionomycin (50 μM) was included in the stimulus buffer for the positive controls (full TRPM4 activation), or ionomycin was omitted for the negative controls (no TRPM4 activation). Both the compound solutions and stimulus solutions were prepared in 96-well clear polypropylene microplates with a V-bottom (Greiner Bio-One 651201). A third control with ionomycin and NBA (10 μM) for full TRPM4 activation and simultaneous full inhibition was included as well. A response baseline correction of fluorescence traces was conducted before stimulus addition. All measured areas under the curves (AUC) were normalized to 25 μM ionomycin + 1% DMSO as the fully activated TRPM4 control and 25 μM ionomycin + 10 μM NBA as the full-block control, defined as 100% TRPM4 inhibition, according to the formula:

$$\mathrm{inhibition}\%=1-\left(\frac{{\overset{®}{\mathrm{AUC}}}_{\mathrm{sample}}-{\overset{®}{\mathrm{AUC}}}_{\mathrm{NBA}}}{{\overset{®}{\mathrm{AUC}}}_{\mathrm{DMSO}}-{\overset{®}{\mathrm{AUC}}}_{\mathrm{NBA}}}\right)\times 100$$

where AUC̅_sample_ is the mean area under the curve for the compound of interest (sample), AUC̅_NBA_ is the mean area under the curve for the full-block NBA control, and AUC̅_DMSO_ is the mean area under the curve for the fully activated DMSO control. All compounds were measured in at least 9 technical replicates from at least 3 independent biological replicates. An outlier test with the ROUT method (*Q* = 1), an ordinary one-way ANOVA test, and Dunnett’s post test were conducted for statistical significance using GraphPad Prism.

### Generation of TRPM4 Knockout HCT116 Cell Lines

CRISPR/Cas9-mediated genome editing was used to generate HCT116 TRPM4 knockout (KO) cell lines. Two guide RNAs (gRNAs) were designed to target exon 2 and exon 3 of the human TRPM4 gene, respectively, in order to induce a double-strand break and excise the intervening genomic region (expected deletion ∼7,947 bp). The gRNA target sequences were as follows: Hs.Cas9.TRPM4.1.AA (GTCAACTATGAACGTCGTGC; exon 2) and Hs.Cas9.TRPM4.1.AD (CGTGAAGTCCAGCTCTCCGT; exon 3). The gRNAs were generated using Alt-R CRISPR-Cas9 reagents (Integrated DNA Technologies, IDT). Briefly, Alt-R CRISPR-Cas9 crRNA Hs.Cas9 (IDT, Cat. No. 138805) was mixed with Alt-R CRISPR-Cas9 tracrRNA, ATTO-550 (IDT, Cat. No. 1075928) to form two distinct gRNA duplexes. Each gRNA was subsequently complexed with Alt-R S.p. HiFi Cas9 Nuclease V3 (IDT, Cat. No. 1081060) according to manufacturer’s instructions. HCT116 cells were transfected using Lipofectamine CRISPRMAX Transfection Reagent (Thermo Fisher Scientific, Cat. No. CMAX00003). 24 h post-transfection, ATTO-550–positive cells were sorted by fluorescence-activated cell sorting (FACS) and seeded into 96-well plates for clonal isolation. Single-cell-derived clones were expanded and screened by genotyping PCR to identify successful genomic deletions. Two validated knockout clones, designated KO1 and KO2, were selected for further characterization. Loss of TRPM4 expression was confirmed at both the mRNA and functional level using RT-qPCR and patch-clamp analysis. The TaqMan assays used for the qPCR experiments were as follows: TRPM4 (Hs01026061_m1) and TATA-box binding protein, used as the reference gene (Hs00427621_m1).

### Patch-clamp Electrophysiology

For the primary compound evaluation experiments, parental HCT116 cells were trypsinized (Gibco) and sparsely seeded into 35 mm cell culture dishes (Falcon) 24 h prior to patch-clamp recordings. In control experiments using TRPM4 knockout HCT116 cells, a subset of cells was additionally transfected with a TRPM4-encoding plasmid using Lipofectamine 2000 (Thermo Fisher Scientific) before being seeded onto patch-clamp dishes. Patch clamp experiments were conducted at rt using a whole-cell configuration. Patch pipettes had resistances ranging between 2 and 3 MΩ. After establishing whole-cell access, voltage ramps were applied every 2 s (0.5 Hz) for 400 s. Each ramp lasted 50 ms and spanned from −100 to +100 mV, starting from a holding potential of 0 mV. Currents were recorded using an EPC-10 amplifier, digitized, and logged via Patchmaster v2 × 53 software (HEKA). Capacitive currents were measured and corrected before each voltage ramp. All voltages were adjusted to account for a liquid junction potential of 10 mV. The currents were filtered at 1 kHz and sampled at 3 kHz. For analysis, currents were extracted at −80 and +80 mV, normalized to cell capacitance, and plotted over time. The bath solution contained the following (in mM): 160 sodium glutamate, 0.5 CaCl_2_, 3 MgCl_2_, and 10 HEPES, adjusted to pH 7.2 with NaOH. The internal solution consisted of the following (in mM): 140 cesium glutamate, 8 NaCl, 5 HEDTA, and 10 HEPES, also adjusted to pH 7.2 with CsOH. Free Mg^2+^ and Ca^2+^ concentrations were maintained at 4 mM and 10 μM, respectively, by calculating total ion concentrations using the WEBMAXC STANDARD tool (https://somapp.ucdmc.ucdavis.edu/pharmacology/bers/maxchelator/webmaxc/webmaxcS.htm). Osmolarity was set to 320 mOsm for the bath solution and 310 mOsm for the internal solution, adjusted with glucose when necessary. All data analysis and graphical representations of the patch clamp experiments were performed using Igor Pro 6.37 software (Wavemetrics). When indicated, after 120 s of baseline recording, the test compounds were applied for 120 s using the perfusion system. Subsequently, the cells were washed with drug-free bath solution.

### Calculation of Inhibitory Potencies

IC_50_ values were calculated using the GraphPad Prism software using the “*log­(inhibitor) vs. normalized response -- variable slope*” function with the following four-parameter logistic model:

$$Y=\frac{100}{\left(1+10^{\left(\log_{10}({\mathrm{IC}}_{50})-X\times \mathrm{Hill}\;\mathrm{slope}\right)}\right)}$$

Ligand efficiencies (LE) of compounds were calculated using the formula:

$$\mathrm{LE}=1.37\cdot \left(\frac{{-\log}_{10}\left({\mathrm{IC}}_{50}\right)}{N_{\mathrm{non}-H}}\right)$$

where *N*_non‑H_ equals the total number of non-hydrogen atoms per molecule. Lipophilic ligand efficiencies (LLE) were then calculated using the following formula:

$$\mathrm{LLE}=p{\mathrm{IC}}_{50}-\log D_{7.4}$$

### Cytotoxicity Assay

HEK293 wild-type cells in the culture medium described above were resuspended in phenol red-free DMEM and seeded to the wells (3000 cells/well, 100 μL/well) of Costar 96-well tissue culture-treated black plates with clear flat bottoms and with low evaporation lids (Corning 3603), which had been coated with poly-d-lysine hydrobromide beforehand. The cell plates were incubated overnight at the cell culture conditions described above to allow for complete cell attachment. The medium in each well was then replaced by fresh phenol red free DMEM + 10% FBS containing either only DMSO (0.1%, full viability control), Triton X-100 (0.1 v/v %, full cell death control), staurosporine (1 μM, 0.1% DMSO, full cell death control), or the compounds to be tested in dilution series (100, 50, 25, 12.5, 6.25, and 3.125 μM, 0.1% DMSO). Wells without cells were included as well. The plates were then incubated at the cell culture conditions described above for 72 h. Phenol red-free DMEM + 10% FBS containing resazurin (0.3 mg/mL) was added to each well (50 μL/well) to reach a final resazurin concentration of 0.1 mg/mL, and the plates were incubated for an additional 2 h. The plates were shaken for 10 s (300 shakes/min, 2 mm shaking diameter), and resorufin fluorescence was measured (λ_ex_ = 520 nm; λ_em_ = 580–640 nm) using a Promega GloMax Explorer plate reader.

### *In Vitro* ADMET Determinations

PBA (**118**) was profiled in selected, commercially available ADME (half-life in human plasma, intrinsic clearance in human liver microsomes, human plasma protein binding), drug–drug interaction (CYP450 inhibition panel, including CYP1A2, 2B6, 2C8, 2C9, 2C19, 2D6, and 3A4), and cardiotoxicity assays (CardiacProfiler Ion Channel Core Panel, including hERG (K_V_11.1), hNa_V_1.5 and hCa_V_1.2); Eurofins Discovery Services North America, LLC, St. Charles, MO, U.S.A; Study ID: US034–0028668.

### Molecular docking

The crystal structure of NBA (Table S8) was first redocked into human TRPM4 bound with NBA (PDB ID: 8RD9) using GOLD (as part of the CCDC Hermes 2025.3.3 package) to validate the docking parameters (Figure S25A). The same parameters were then used to dock PBA (**118**), whose structure was generated and energy minimized (MM2 force field) using Chem3D (v25.5.0.5789, Revvity Signals Software, Inc.), into human TRPM4 (PDB ID: 8RD9).

## Supplementary Material





## Notes

Representative traces from fluorescence-based assay and its validation, single concentration activities of compounds measured in the fluorescence-based assay, dose-inhibition curves from fluorescence-based assay, additional patch-clamp electrophysiology data, *in vitro* toxicity data, *in vitro* ADME data, docking models, UV–vis absorption spectra, X-ray crystal structures, representative HPLC traces, HPLC purities of all final compounds, relevant NMR spectra (^1^H, ^13^C, NOESY and temperature dependent ^1^H-NMR), and ^1^H- and ^13^C-NMR spectra of final compounds (PDF)
Molecular formula strings of all tested compounds (CSV)

## ABBREVIATIONS

ACN: acetonitrile
FBS: fetal bovine serum
FLIPR: fluorometric imaging plate reader
HEPES: 4-(2-hydroxyethyl)-1-piperazineethanesulfonic acid
LLE: lipophilic ligand efficiency
TPSA: topical polar surface area
TRPM4: transient receptor potential melastatin 4
