# Supplementary material for: Potent Anthranilic Anilide-Based TRPM4 Channel Inhibitors Identified by a Structure–Activity Relationship Study
Source: J Med Chem. 2026 Apr 13;69(8):8763–89. doi: 10.1021/acs.jmedchem.5c02015 (PMC13126687; doi:10.1021/acs.jmedchem.5c02015)

SUPPORTING INFORMATION

Potent Anthranilic Anilide-based TRPM4 Channel Inhibitors Identified by SAR Study

Christian E. Gerber, Bartlomiej S. Augustynek, Philipp Grossenbacher, Barbara Hauert, Simon A. Singer, Christine Peinelt, Martin Lochner*

Institute of Biochemistry and Molecular Medicine, University of Bern, Bühlstrasse 28, 3012 Bern, Switzerland

*Corresponding author: martin.lochner@unibe.ch

Table of Contents

1. BIOLOGY ... S2
  1.1 Fluorescence-based Sodium Influx Assay ... S2
  1.2 Patch-clamp Electrophysiology ... S8
  1.3 *In vitro* Toxicity ... S10
  1.4 *In vitro* ADME Properties ... S12
2. CHEMISTRY ... S15
  2.1 UV-Vis Absorption Spectra ... S15
  2.2 X-ray Crystal Structures ... S15
  2.3 HPLC Traces and Purities ... S18
  2.4 NMR Data for Conformational Analysis ... S20
  2.5 Molecular Docking ... S24
  2.6 ^{1}H- and ^{13}C-NMR Spectra ... S25

1. BIOLOGY

1.1 Fluorescence-based Sodium Influx Assay

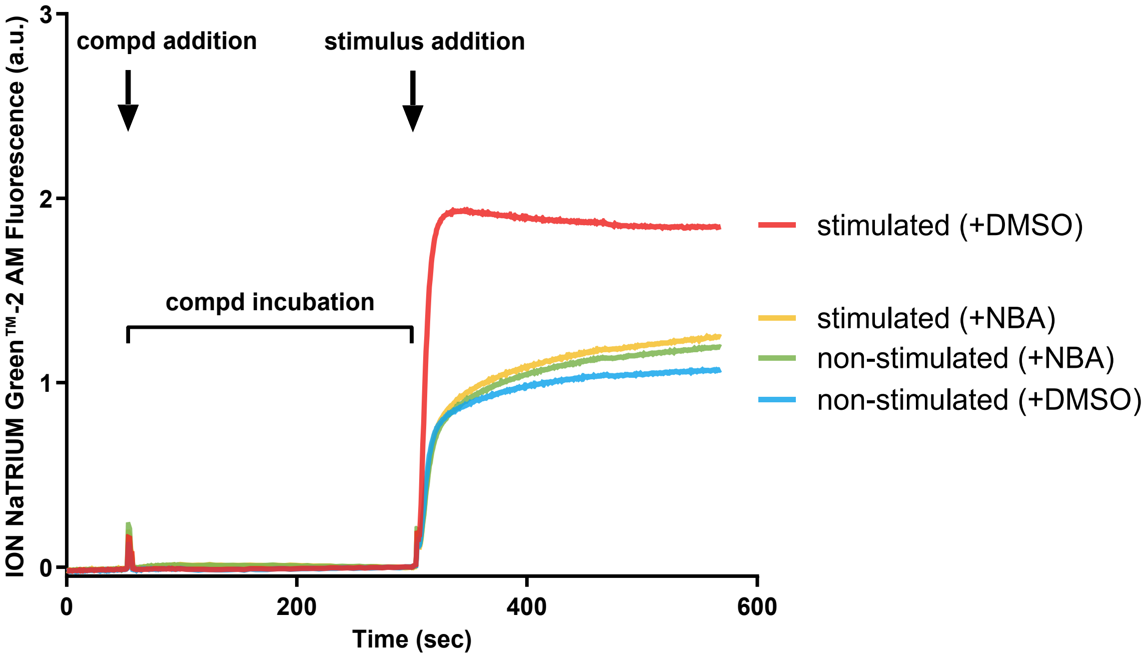

Figure S1. Representative ION NaTRIUM Green™-2 AM fluorescence intensity traces measured in HEK293 cells stably overexpressing hTRPM4, incubated either with NBA (10 μM, full-block control, yellow trace) or DMSO (full-activated control, red trace) and stimulated with ionomycin (arrow indicates stimulus addition). Traces of non-stimulated NBA control (green trace) and non-stimulated DMSO control (blue trace) are displayed for comparison.

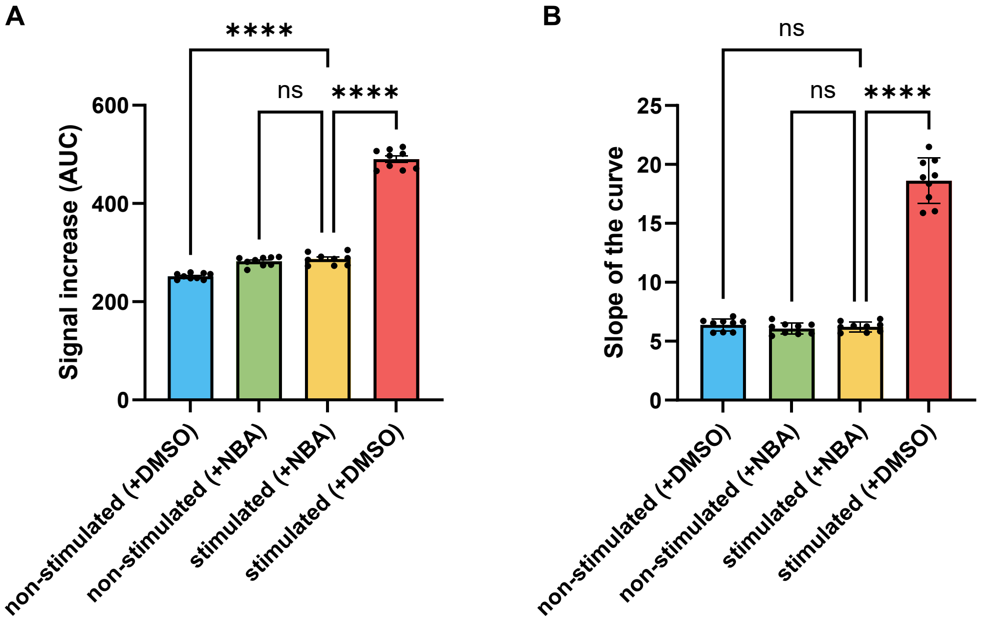

Figure S2. Validation of NBA controls by comparison of (A) ION NaTRIUM Green™-2 AM fluorescence signal increase (AUC) and (B) curve slopes in stimulated cells incubated with 10 μM NBA (yellow) with stimulated cells incubated with DMSO (red) and non-stimulated cells incubated with either DMSO (blue) or 10 μM NBA (green). Ordinary one-way ANOVA tests and Dunnett's multiple comparisons tests were conducted using GraphPad Prism for statistical significance (****: $p \leq 0.0001$, ns: $p \geq 0.05$).

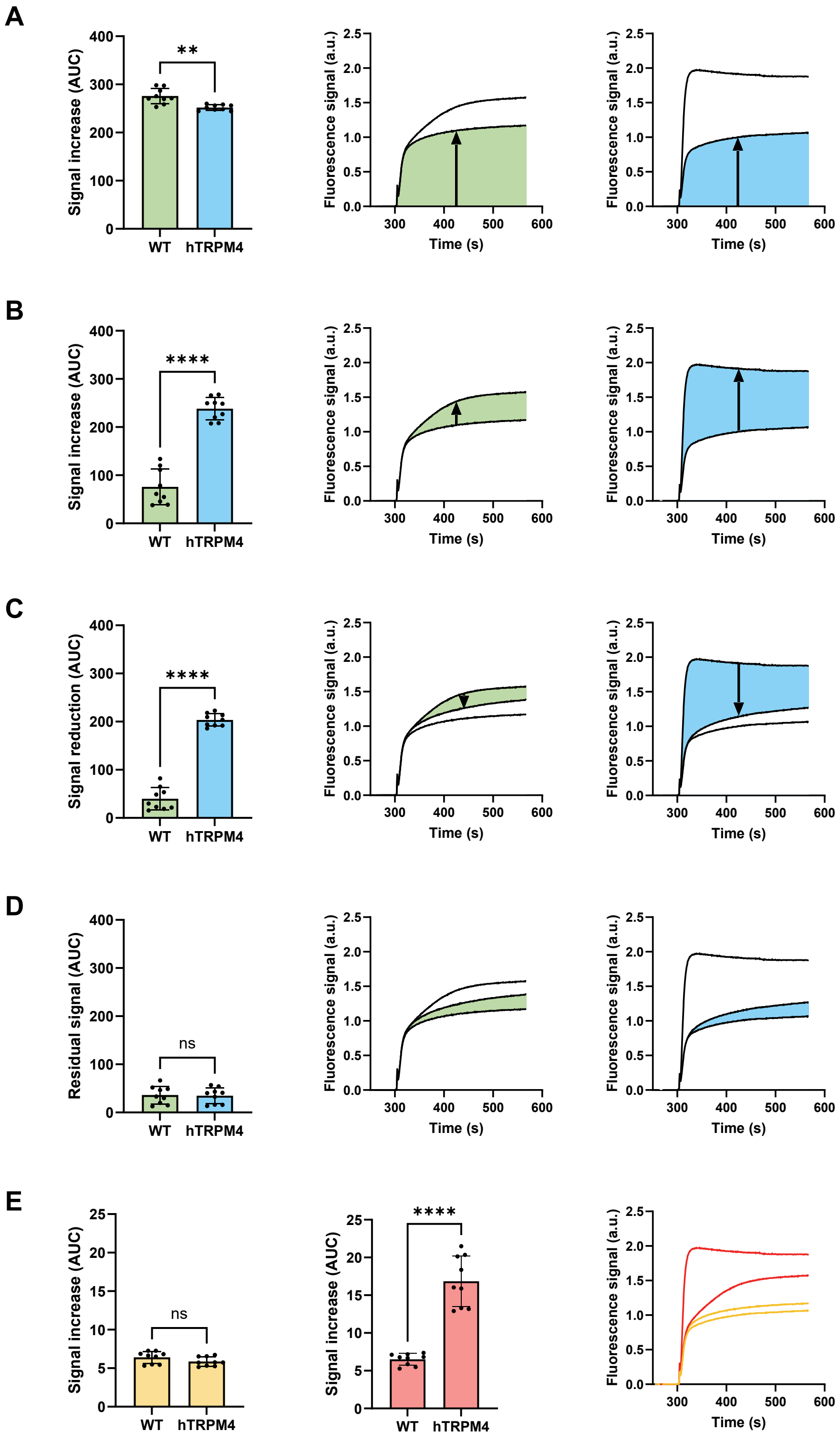

Figure S3. Validation of target engagement in the fluorescence-based Na^+ influx assay by the comparison of HEK293 WT cells (green) with HEK293 cells stably overexpressing hTRPM4 (blue). Welch's *t*-tests were performed using GraphPad Prism for statistical significance (****: $p \leq 0.0001$, **: $p \leq 0.01$, ns: $p \geq 0.05$). (A) Fluorescence signal increase measured in non-stimulated WT and TRPM4 cells upon Na^+ buffer addition. (B) Additional fluorescence signal increase in stimulated WT and TRPM4 cells upon Na^+ buffer addition. Area under the curve of signals from non-stimulated WT and TRPM4 cells were subtracted. (C) Fluorescence signal reduction mediated by 10 µM NBA in stimulated WT and TRPM4 cells upon Na^+ buffer addition. (D) Area between the curves of stimulated WT and TRPM4 cells treated with 10 µM NBA and non-stimulated WT and TRPM4 cells upon Na^+ buffer addition. (E) Comparison of curve slopes measured in either non-stimulated (yellow) or stimulated (red) WT and hTRPM4 cells.

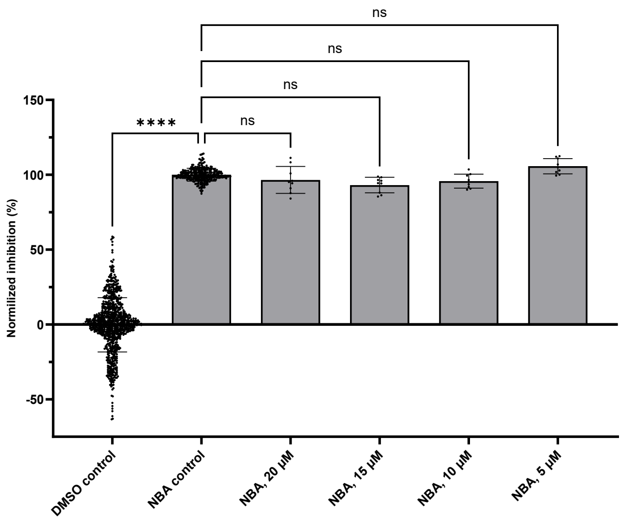

Figure S4. TRPM4 inhibition of control compound NBA tested *in vitro* at different concentrations of 20, 15, 10 and 5 µM in the fluorescence-based Na^+-influx assay on HEK293 cells, stably overexpressing hTRPM4. Fluorescence responses were normalized to 10 µM NBA as the full-block control to calculate TPRM4 % inhibition, where 100% ≙ full inhibition. Bar plots show mean ± SEM and scattered dots show values from at least three independent biological replicates with at least three technical replicates each (n = 3 × 3 = 9). An ordinary one-way ANOVA test and a Dunnett's multiple comparisons test was conducted using GraphPad Prism for statistical significance (****: $p \leq 0.0001$, ns: $p \geq 0.05$).

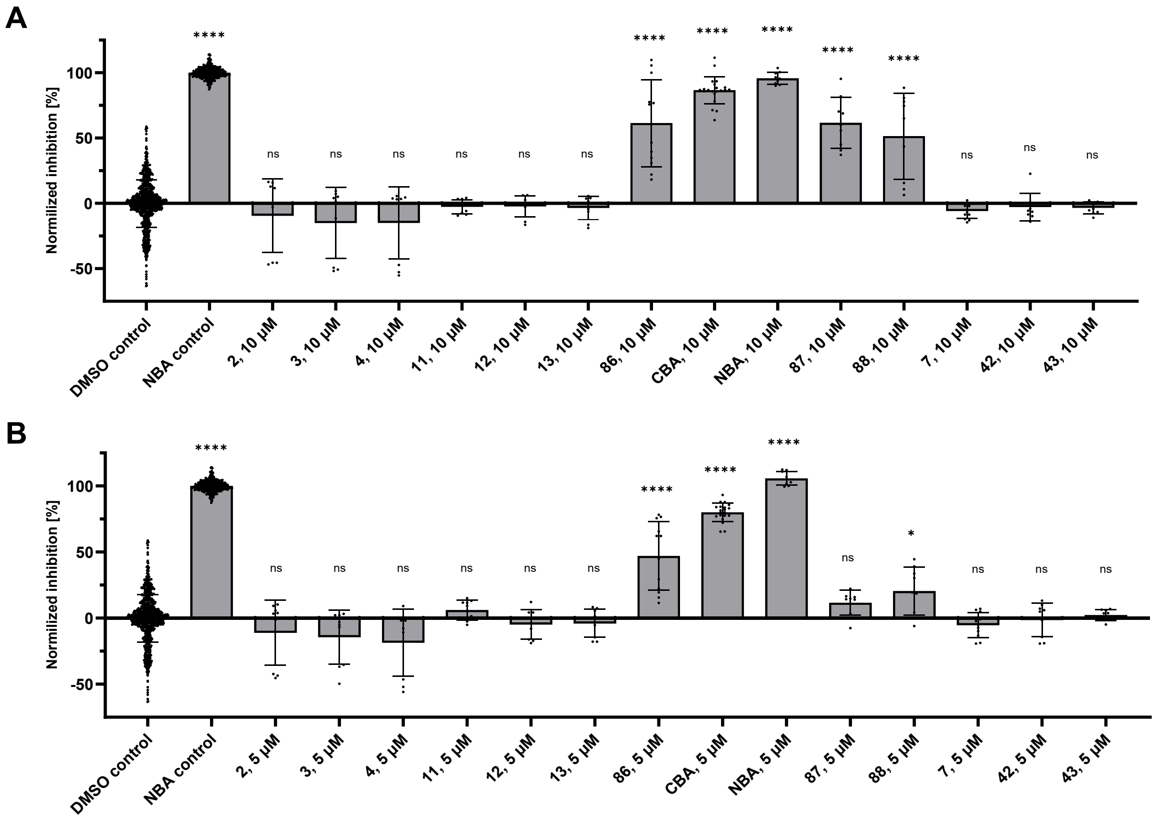

Figure S5. TRPM4 inhibition of truncated compounds **2** – **4**, truncated anthranilic anilides **11** – **13**, anthranilic anilides **86** – **88**, CBA, NBA and methyl anthranilates **7**, **42**, **43** tested *in vitro* at (A) 10 µM and (B) 5 µM concentrations in the fluorescence-based Na^+-influx assay on HEK293 cells, stably overexpressing hTRPM4. Fluorescence responses were normalized to 10 µM NBA as the full-block control to calculate TPRM4 % inhibition, where 100% ≙ full inhibition. Bar plots show mean ± SEM and scattered dots show values from at least three independent biological replicates with at least three technical replicates each (n = 3 × 3 = 9). Ordinary one-way ANOVA tests and Dunnett's multiple comparisons tests were conducted using GraphPad Prism for statistical significance (****: $p \leq 0.0001$, *: $p \leq 0.05$, ns: $p \geq 0.05$).

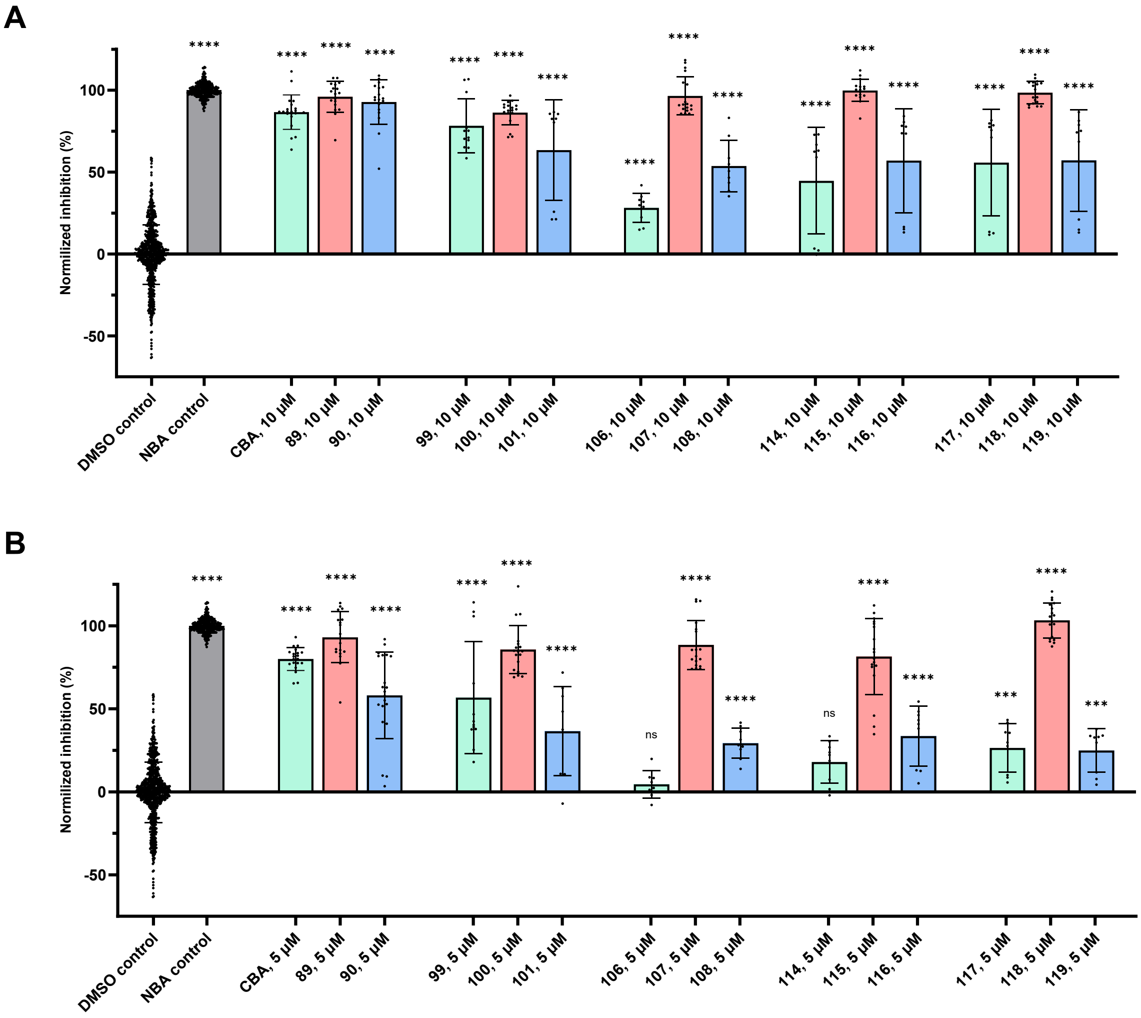

Figure S6. TRPM4 inhibition of anthranilic anilides CBA, **89**, **90**, **99** – **101**, **106** – **108** and **114** – **119** with substituents either in *ortho* (green)-, *meta* (red)- or *para* (blue)-position of the phenoxy ring, tested *in vitro* at (A) 10 µM and (B) 5 µM concentrations in the fluorescence-based Na^+-influx assay on HEK293 cells, stably overexpressing hTRPM4. Fluorescence responses were normalized to 10 µM NBA as the full-block control to calculate TPRM4 % inhibition, where 100% ≙ full inhibition. Bar plots show mean ± SEM and scattered dots show values from at least three independent biological replicates with at least three technical replicates each ($n = 3 \times 3 = 9$). Ordinary one-way ANOVA tests and Dunnett's multiple comparisons tests were conducted using GraphPad Prism for statistical significance (****: $p \leq 0.0001$, ***: $p \leq 0.001$, ns: $p \geq 0.05$).

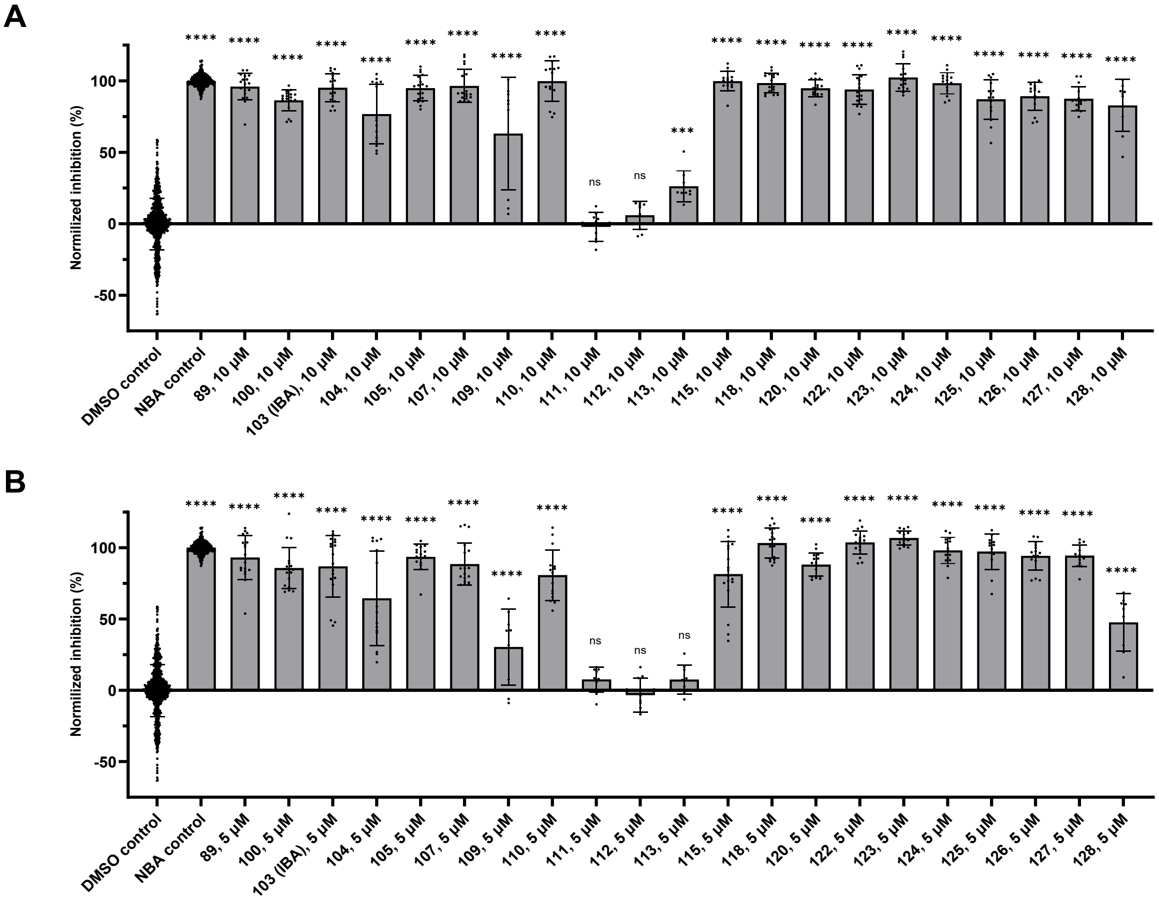

Figure S7. TRPM4 inhibition of anthranilic anilides **89**, **100**, **103** – **105**, **107**, **109** – **113**, **115**, **118**, **120** and **122** – **128** with various substituents in *meta*-position tested *in vitro* at (A) 10 µM and (B) 5 µM concentrations in the fluorescence-based Na^+-influx assay on HEK293 cells, stably overexpressing hTRPM4. Fluorescence responses were normalized to 10 µM NBA as the full-block control to calculate TPRM4 % inhibition, where 100% ≙ full inhibition. Bar plots show mean ± SEM and scattered dots show values from at least three independent biological replicates with at least three technical replicates each (n = 3 × 3 = 9). Ordinary one-way ANOVA tests and Dunnett's multiple comparisons tests were conducted using GraphPad Prism for statistical significance (****: $p \leq 0.0001$, ***: $p \leq 0.001$, ns: $p \geq 0.05$).

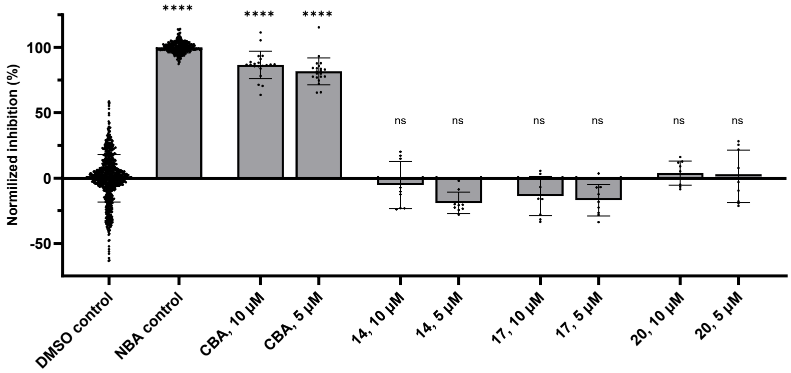

Figure S8. TRPM4 inhibition of compounds CBA, **14**, **17** and **20** with various numbers of hydrogen bond acceptors adjacent to the amide-H measured at 10 and 5 µM concentrations, respectively, in the fluorescence-based Na^+-influx assay on HEK293 cells, stably overexpressing hTRPM4. Fluorescence responses were normalized to 10 µM NBA as the full-block control to calculate TPRM4 % inhibition, where 100% ≙ full inhibition. Bar plots show mean ± SEM and scattered dots show values from at least three independent biological replicates with at least three technical replicates each (n = 3 × 3 = 9). An ordinary one-way ANOVA test and a Dunnett's multiple comparisons test was conducted using GraphPad Prism for statistical significance (****: $p \leq 0.0001$, ns: $p \geq 0.05$).

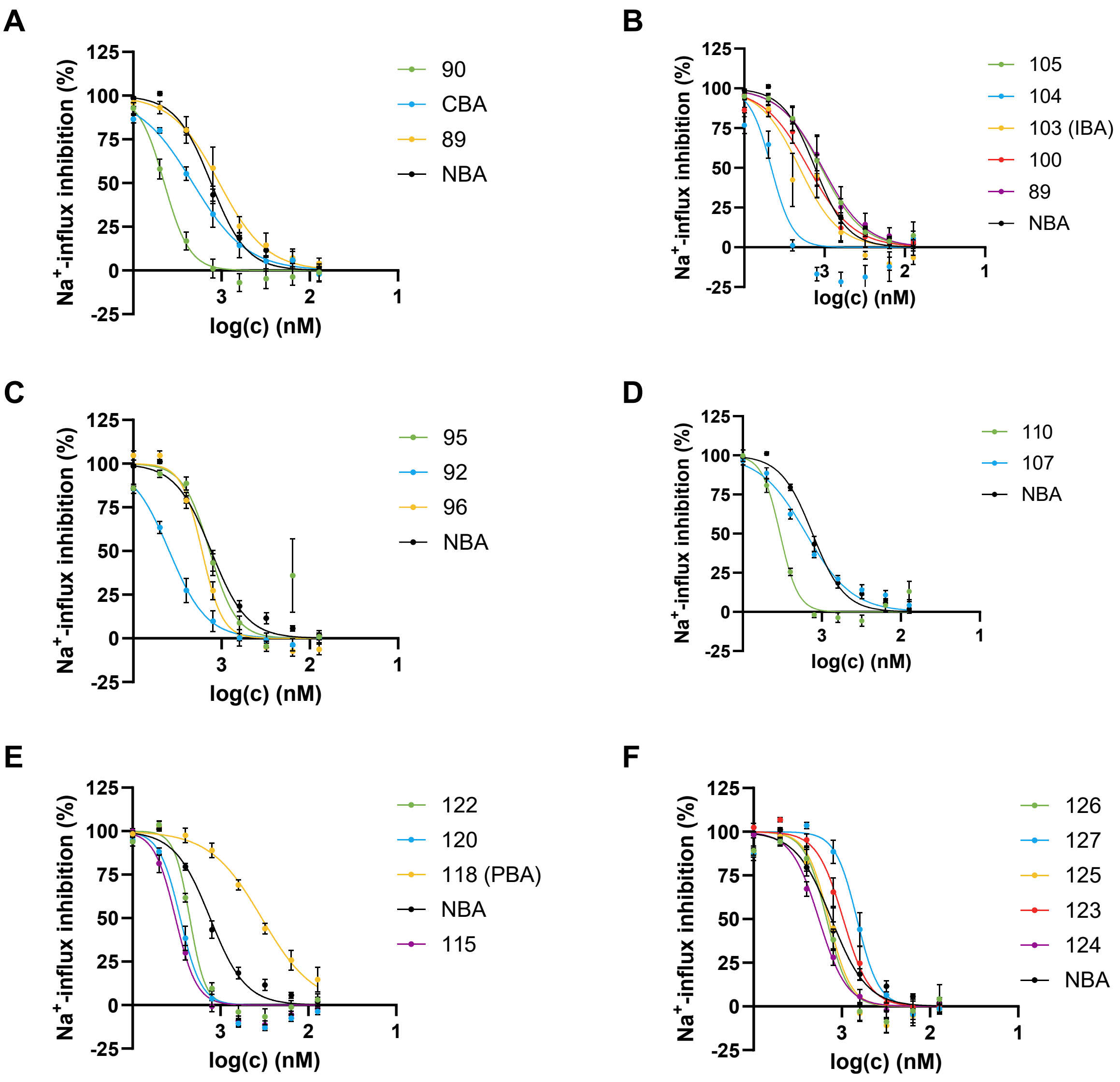

Figure S9. Dose-response curves from the fluorescence-based Na^+-influx assay on HEK293 cells, stably overexpressing hTRPM4 for the determination of IC_{50}-values of phenoxy ring-substituted derivatives. Coloured lines show nonlinear curve fits calculated with the Hill equation (variable slope) in GraphPad Prism. Data points show mean ± SEM from at least three independent biological replicates with at least three technical replicates each ($n = 3 \times 3 = 9$). All plots include the dose-response curve of reference compound NBA (black) for comparison. (A) Dose-response curves of the compounds chlorinated either in *ortho*-, *meta*-, or *para*-position: CBA (2-Cl), **89** (3-Cl) and **90** (4-Cl). (B) Dose-response curves of the compounds halogenated in *meta*-position: **104** (3-F), **89** (3-Cl), **100** (3-Br), **103 / IBA** (3-I) and **105** (3-CF_3). (C) Dose-response curves of the dichlorinated compounds **92** (2,4-Cl_2), **95** (3,4-Cl_2) and **96** (3,5-Cl_2). (D) Dose-response curves of the compounds with nitrogen containing substituents: **107** (3-NO_2) and **110** (3-CN). (E) Dose-response curves of the compounds with ether substituents: **115** (3-OMe), **118 / PBA** (3-propargyloxy), **120** (3-(but-2-yn-1-yloxy)) and **122** (3-O*i*Bu). (F) Dose-response curves of the compounds with aliphatic substituents: **123** (3-ethynyl), **124** (3-Me), **125** (3-Et), **126** (3-*n*Pr) and **127** (3-*i*Pr).

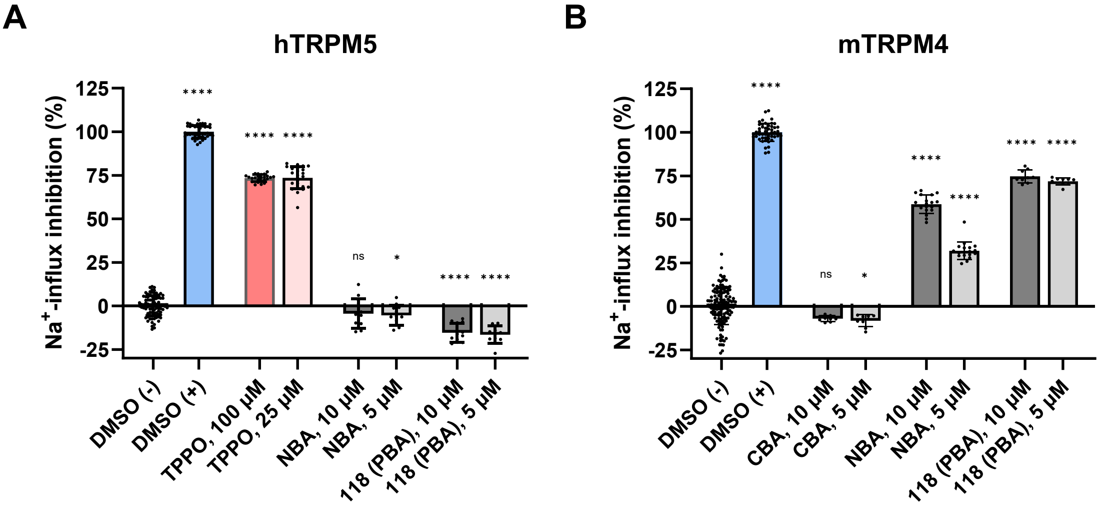

Figure S10. (A) Na^+-influx inhibition into HEK293 cells, stably overexpressing hTRPM5, treated with compounds CBA, NBA or **118** (PBA), at 10 and 5 µM concentrations, respectively, and measured using the fluorescence-based Na^+-influx assay. TRPM5 inhibitor triphenylphosphine oxide (TPPO) at 100 and 25 µM concentrations, respectively, was included as a full-block control. (B) Na^+-influx inhibition into TsA-201 cells, stably overexpressing mTRPM4, treated with compounds CBA, NBA or **118** (PBA) at 10 and 5 µM concentrations, respectively, and measured using the fluorescence-based Na^+-influx assay. Fluorescence responses were normalized to non-stimulated cells (no ionomycin) to calculate Na^+-influx inhibition %. Bar plots show mean ± SD and scattered dots show values from at least three independent biological replicates with at least three technical replicates each (n = 3 × 3 = 9). Ordinary one-way ANOVA tests and Dunnett's multiple comparisons tests were conducted using GraphPad Prism for statistical significance (****: $p \leq 0.0001$, *: $p \leq 0.05$, ns: $p \geq 0.05$).

1.2 Patch-clamp Electrophysiology

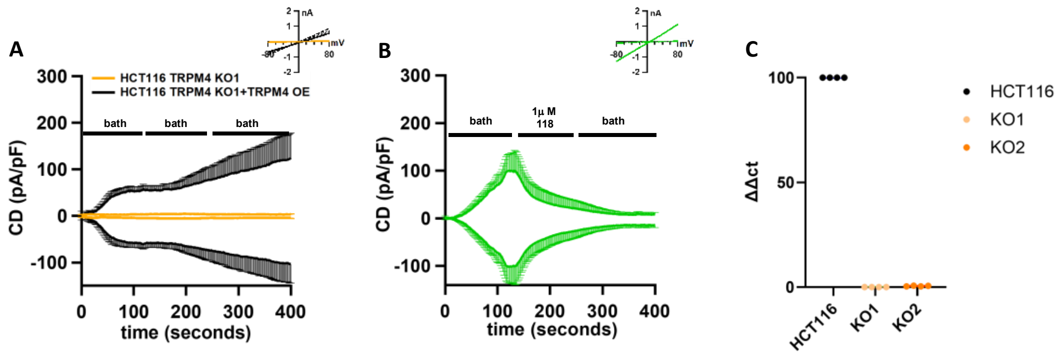

Figure S11. Evaluation of the TRMP4 KO HCT116 cells. (A+B) Whole-cell patch-clamp recordings from HCT116 cells with 10 µM free Ca^{2+} in the pipette. Currents were normalized to cell size and plotted as current densities (CD; mean ± SEM) over time. Insets show current–voltage (I/V) relationships recorded before drug application (solid line, t = 100 s), during drug application (dotted line, t = 220 s), and after washout with bath solution (dashed line, t = 300 s). (A) Control (bath solution applied from 120-240 s; golden traces from HCT116 TRPM4 KO cells (n = 11), black traces from TRPM4-overexpressing HCT116 TRPM4 KO cells (n = 11). (B) 1 µM compound **118** (PBA) applied from 120-240 s to TRPM4-overexpressing HCT116 TRPM4 KO cells (n = 11). (C) TRPM4 expression relative to HCT116. qPCR analysis of the TRPM4 gene expression in the KO cell lines KO1 and KO2, relative to parental HCT116 (n = 4).

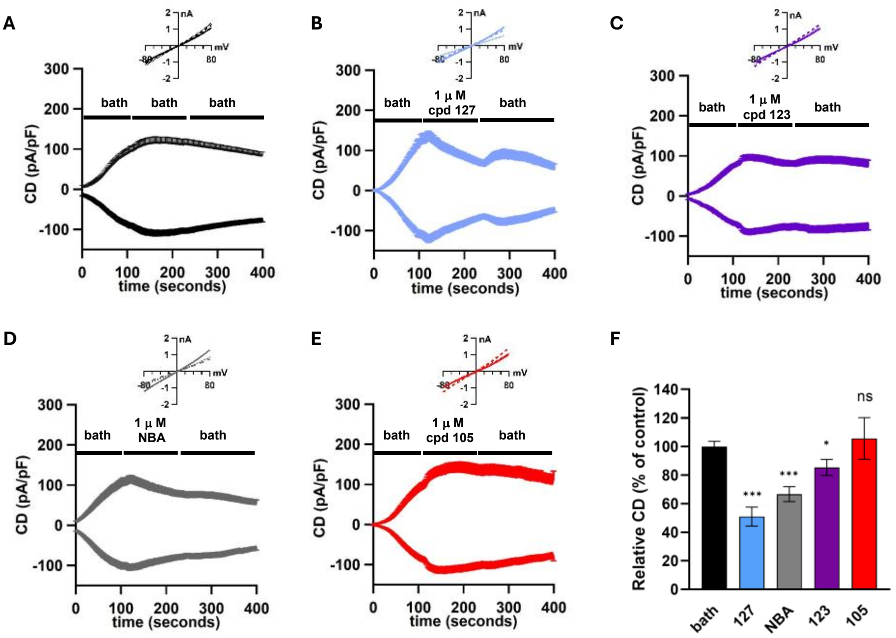

Figure S12. Determination of the potency of novel compounds in inhibiting endogenous TRPM4 currents in HCT116 cells. (A–E) Whole-cell patch-clamp recordings from HCT116 cells with 10 µM free Ca^{2+} in the pipette. Currents were normalized to cell size and plotted as current densities (CD; mean ± SEM) over time. Insets show current–voltage (I/V) relationships recorded before drug application (solid line, t = 100 s), during drug application (dotted line, t = 220 s), and after washout with bath solution (dashed line, t = 300 s). (A) Control (bath solution applied from 120–240 s; n = 13). (B) 1 µM compound **127** applied from 120–240 s (n = 9). (C) 1 µM compound **123** applied from 120–240 s (n = 7). (D) 1 µM NBA applied from 120–240 s (n = 22). (E) 1 µM compound **105** applied from 120–240 s (n = 10). (F) Corresponding TRPM4 current inhibition by the compounds NBA, **105**, **123** and **127**, expressed as a percentage of control. Student's t-tests were conducted for statistical significance (***: $p \leq 0.001$, *: $p \leq 0.05$, ns: $p \geq 0.05$).

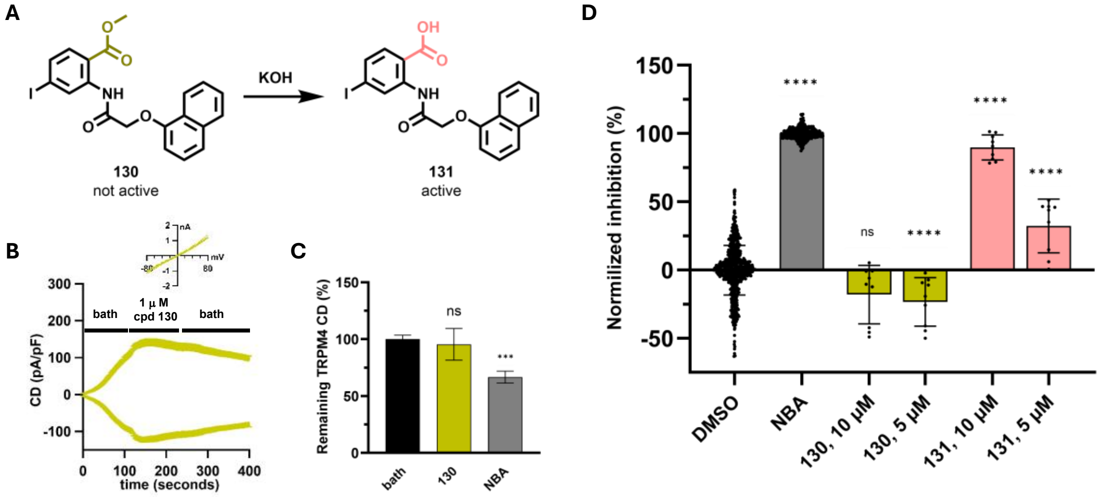

Figure S13. (A) Structures of published[32] methyl anthranilate **130** and its corresponding carboxylic acid **131** obtained by basic hydrolysis. (B) Whole-cell patch-clamp recordings from HCT116 cells with 10 µM free Ca^{2+} in the pipette. Currents were normalized to cell size and plotted as current densities (CD; mean ± SEM) over time. Insets show current–voltage (I/V) relationships recorded before drug application (solid line, t = 100 s), during drug application (dotted line, t = 220 s), and after washout with bath solution (dashed line, t = 300 s). 1 µM compound **130** applied from 120–240 s (n = 12). (C) Corresponding TRPM4 current inhibition by compound **130**, expressed as a percentage of control compared to the TRPM4 current inhibition by NBA. Student's t-tests were conducted for statistical significance (***: $p \leq 0.001$, ns: $p \geq 0.05$). (D) Activities of methyl anthranilate **130** and corresponding carboxylic acid **131** measured in the fluorescence-based Na^+-influx assay on HEK293 cells overexpressing hTRPM4. Fluorescence responses were normalized to 10 µM NBA as the full-block control to calculate TPRM4 % inhibition, where 100% ≙ full inhibition. Bar plots show mean ± SEM and scattered dots show values from at least three independent biological replicates with at least three technical replicates each (n = 3 × 3 = 9). Ordinary one-way ANOVA tests and Dunnett's multiple comparisons tests were conducted using GraphPad Prism for statistical significance (****: $p \leq 0.0001$, ns: $p \geq 0.05$).

1.3 *In vitro* Toxicity

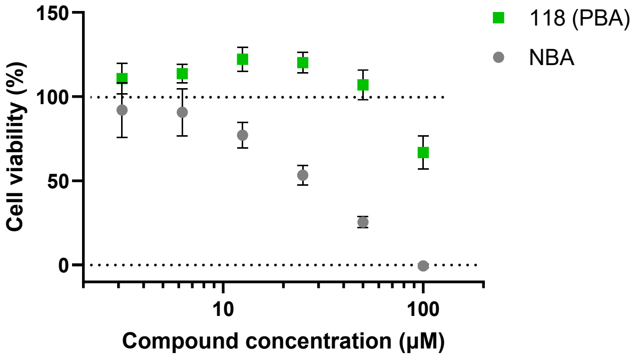

Figure S14. Cell viability of HEK293 WT cells treated with either NBA (grey dots) or **118** (PBA, green squares) in 2-fold dilution series from 100 µM – 3.125 µM and incubated for 72 h under cell culture conditions (see *Experimental*). DMSO (0.1%) controls, representing full cell viability, and staurosporine (1 µM) controls, representing zero cell viability, were included. Resorufin (converted from resazurin) fluorescence from compound treated wells was normalized to the mean fluorescence from DMSO controls and staurosporine controls. 100% and 0% cell viability are indicated with dotted lines. Coloured symbols show mean ± SD from at least three independent biological replicates with at least three technical replicates each (n = 3 × 3 = 9).

Table S1. Activity of 118 (PBA) on hNa$_v$1.5.

Test compound	Concentration (µM)	hNa$_v$1.5 inhibition (%)		
		n_1	n_2	mean
**118** (PBA)	0.3125	-3.77	-0.41	-2.09
**118** (PBA)	0.625	1.08	1.88	1.48
**118** (PBA)	1.25	-1.97	0.69	-0.64
**118** (PBA)	2.5	4.03	1.89	2.96
**118** (PBA)	5	1.01	0.92	0.97
**118** (PBA)	10	4.32	0.62	2.47
Time-Matched Vehicle Control	0.33% DMSO	0.13	0.15	0.14

Table S2. Activity of 118 (PBA) on hCa$_v$1.2.

Test compound	Concentration (µM)	hCa$_v$1.2 inhibition (%)		
		n_1	n_2	mean
**118** (PBA)	0.3125	14.65	9.24	11.95
**118** (PBA)	0.625	4.86	21.41	13.14
**118** (PBA)	1.25	9.72	11.02	10.37
**118** (PBA)	2.5	3.05	2.26	2.66
**118** (PBA)	5	7.22	7.23	7.23
**118** (PBA)	10	8	8.19	8.09
Time-Matched Vehicle Control	0.33% DMSO	8.61	9.96	9.28

Table S3. Activity of 118 (PBA) on hERG.

Test compound	Concentration (μM)	hERG inhibition (%)		
		n_1	n_2	mean
**118** (PBA)	0.3125	-0.99	-3.02	-2
**118** (PBA)	0.625	5.99	-3.66	1.16
**118** (PBA)	1.25	-2.08	2.9	0.41
**118** (PBA)	2.5	2.46	5.2	3.83
**118** (PBA)	5	-6.82	2.22	-2.3
**118** (PBA)	10	-3.32	-8.27	-5.8
Time-Matched Vehicle Control	0.33% DMSO	0.44	2.06	1.25

Table S4. Inhibition of Various CYP Enzymes by 118 (PBA) in Human Liver Microsome.

CYP (HLM)	Substrate	Test compound at 10 μM	CYP inhibition (% of control values)		
			n_1	n_2	mean
CYP1A2	phenacetin	**118** (PBA)	19.2	3	11.1
CYP2B6	bupropion	**118** (PBA)	9.2	14.1	11.6
CYP2C8	amodiaquine	**118** (PBA)	53	54.4	53.7
CYP2C9	diclofenac	**118** (PBA)	87	90.2	88.6
CYP2C19	omeprazole	**118** (PBA)	36.9	38.2	37.6
CYP2D6	dextromethorphan	**118** (PBA)	-1.5	-5.7	-3.6
CYP3A4	midazolam	**118** (PBA)	33	30.3	31.7
CYP3A4	testosterone	**118** (PBA)	30.9	38.5	34.7

1.4 *In vitro* ADME Properties

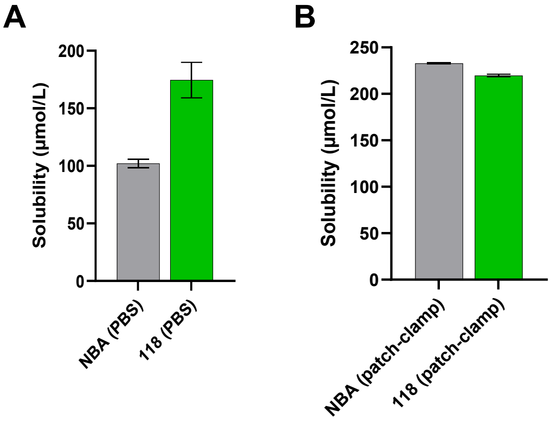

Figure S15. Solubility of NBA and **118** (PBA) in different aqueous buffer solutions. (A) Solubility in phosphate-buffered saline (PBS) containing phosphate buffer components (10 mM), NaCl (138 mM) and KCl (2.7 mM), pH 7.4. (B) Solubility in the bath solution used during patch-clamp measurements on HCT116 cells, endogenously expressing TRPM4 containing HEPES (10 mM), sodium glutamate (160 mM), $CaCl_2$ (0.5 mM) and $MgCl_2$ (3 mM), adjusted to pH 7.2 with NaOH.

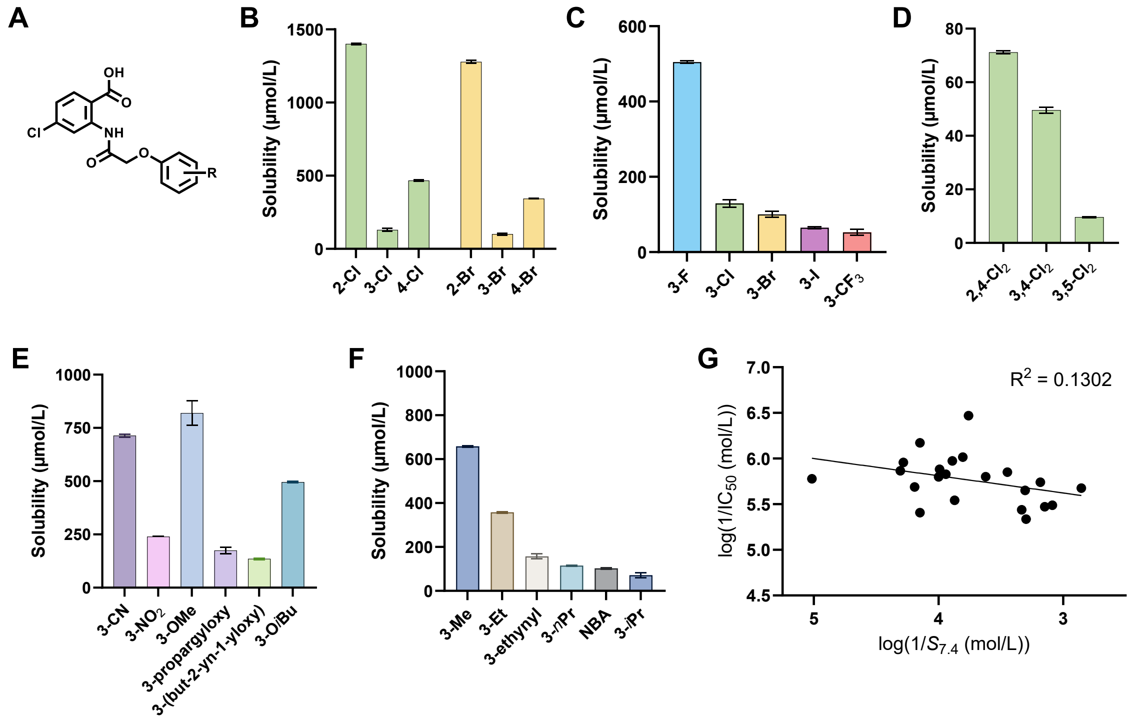

Figure S16. (A) Anthranilic anilide scaffold with R = substituents at the phenoxy ring. (B – F) Bar plots show mean solubilities (n = 3 x 3 = 9) ± SD of active anthranilic anilides from the SAR-study measured in PBS (pH 7.4) containing phosphate buffer components (10 mM), NaCl (138 mM) and KCl (2.7 mM) +1% DMSO. (B) Solubilities of anthranilic anilides CBA (2-Cl), **89** (3-Cl), **90** (4-Cl), **99** (2-Br), **100** (3-Br) and **101** (4-Br) with halogens either in *ortho*-, *meta*-, or *para*-position of the phenoxy ring. (C) Solubilities of anthranilic anilides **104** (3-F), **89** (3-Cl), **100** (3-Br), **103** (3-I) and **105** (3-CF_3) with halogen substituents in *meta*-position. (D) Solubilities of dichlorinated anthranilic anilides **92** (2,4-Cl_2), **95** (3,4-Cl_2) and **96** (3,5-Cl_2). (E) Solubilities of anthranilic anilides **110** (3-CN), **107** (3-NO_2), **115** (3-OMe), **118** (3-propargyloxy), **120** (3-(but-2-yn-1-yloxy)) and **122** (3-O*i*Bu) with heteroatom-containing substituents in *meta*-position. (F) Solubilities of anthranilic anilides **124** (3-Me), **125** (3-Et), **123** (3-ethynyl), **126** (3-*n*Pr), NBA and **127** (3-*i*Pr) with aliphatic substituents in *meta*-position. (G) Biological activity of compounds measured in the fluorescence-based Na^+-influx assay on HEK293 cells, stably overexpressing TRPM4 plotted against respective measured aqueous solubilities shows no correlation ($R^2 = 0.1302$).

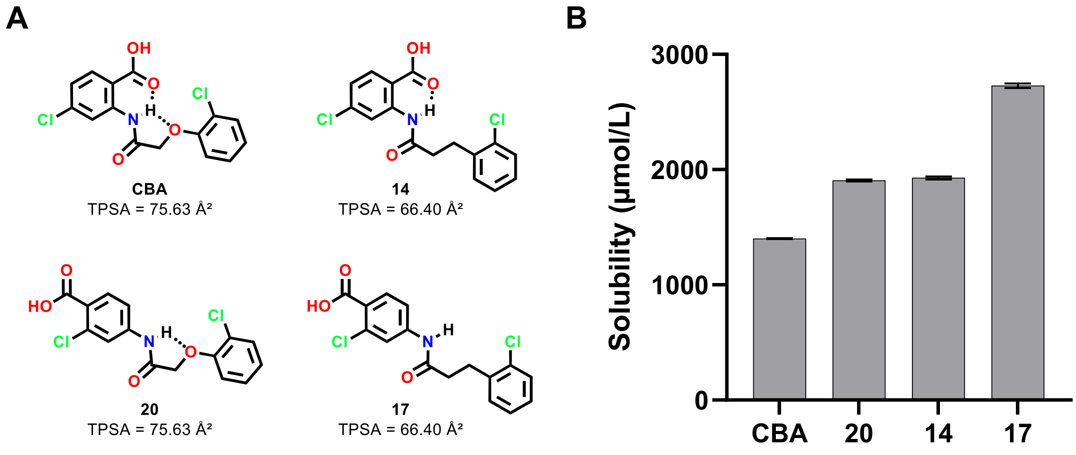

Figure S17. (A) Structures of compounds CBA, **14**, **17** and **20** with various numbers of hydrogen bond acceptors adjacent to the amide-H. The corresponding calculated topical polar surface area (TPSA) of the compounds are specified. (B) Bar plot shows mean solubilities (n = 3 x 3 = 9) ± SD measured in PBS +1% DMSO of CBA, **14**, **17** and **20**.

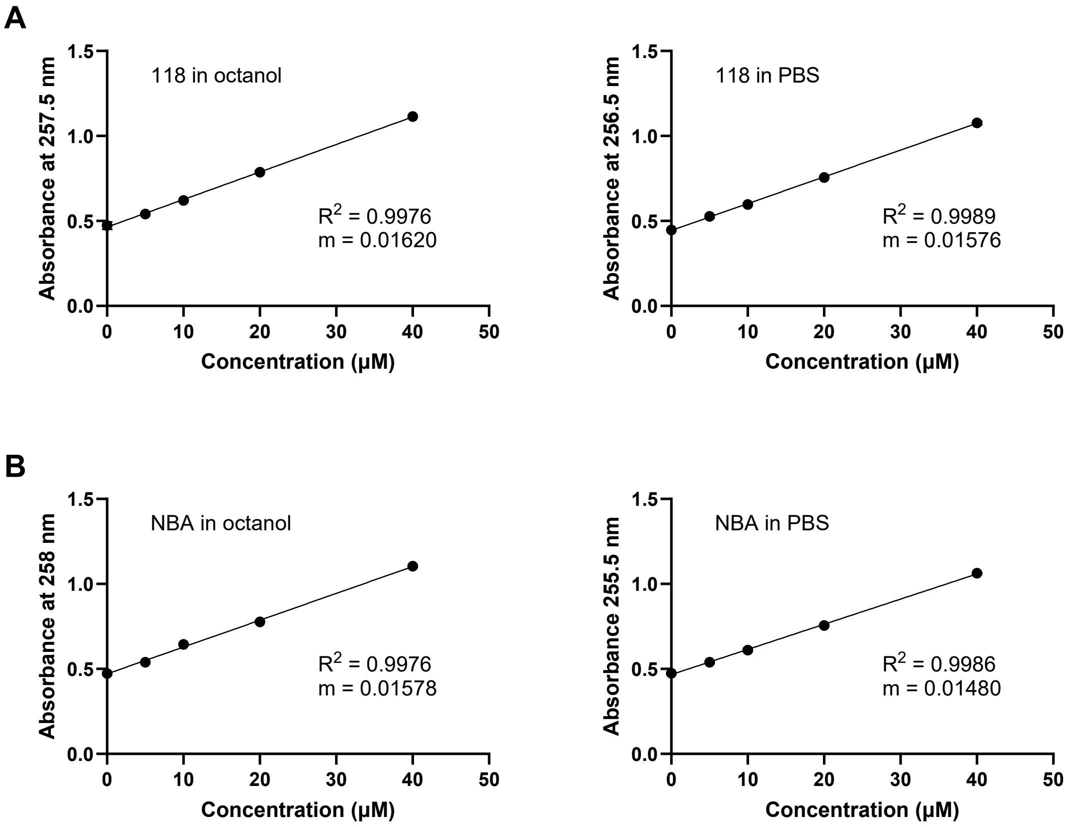

Figure S18. Calibration lines for the calculation of log $D_{7.4}$ values for compound **118** (A) and NBA (B) were determined by UV-Vis absorption and absorbance was measured at the corresponding λ_{max} at compound concentrations of 40, 20, 10 and 5 µM in octanol (left) and in PBS (right). Simple linear regression was calculated using GraphPad Prism and dots show mean absorbance ± SD (n = 9). Due to small SD, plotted error bars are not visible.

Table S5. Human Plasma Protein Binding of 118 (PBA).

Test compound	Concentration (μM)	% protein bound (human plasma)		
		n_1	n_2	mean
**118** (PBA)	10	99.65	99.75	99.70

Table S6. Stability Data to Determine the Half-Life of 118 (PBA) in Human Plasma.

Test compound	Concentration (μM)	Incubation time (min)	% compound remaining (human plasma)		
			n_1	n_2	mean
**118** (PBA)	1	0	100	100	100
**118** (PBA)	1	30	96.04	90.68	93.36
**118** (PBA)	1	60	103.3	94.37	98.84
**118** (PBA)	1	90	97.17	94.4	95.78
**118** (PBA)	1	120	99.54	95.05	97.3

Table S7. Stability Data to Determine the Half-Life of 118 (PBA) in Human Liver Microsomes.

Test compound	Concentration (μM)	Incubation time (min)	% compound remaining (human liver microsomes)		
			n_1	n_2	mean
**118** (PBA)	0.1	0	100	100	100
**118** (PBA)	0.1	15	77.07	73.4	75.24
**118** (PBA)	0.1	30	48.07	44.61	46.34
**118** (PBA)	0.1	45	24.45	25.87	25.16
**118** (PBA)	0.1	60	14.67	13.22	13.95

2. CHEMISTRY

2.1 UV-Vis Absorption Spectra

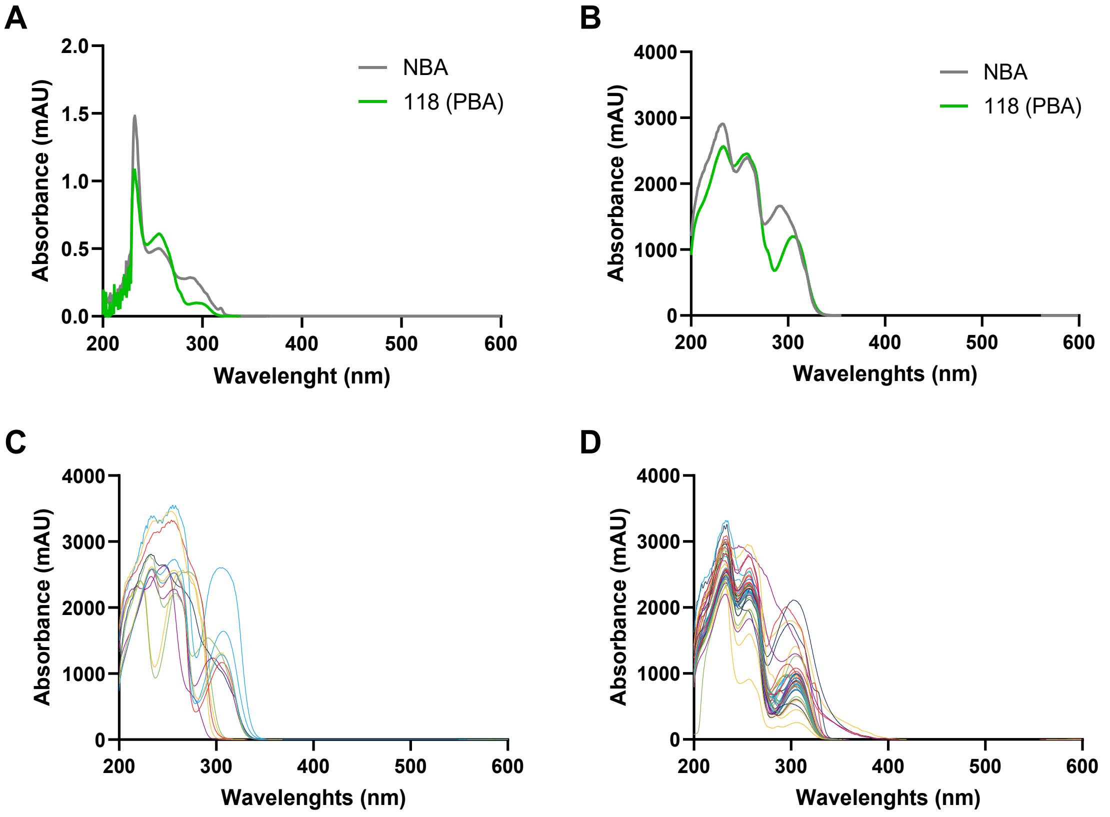

Figure S19. UV-Vis absorption spectra recorded between 200 – 600 nm. Absorption maxima were observed in the region between 200 – 350 nm for all compounds. (A) UV-Vis absorption spectra of NBA and **118** (PBA) measured using a UV-Vis spectrophotometer at 40 µM concentrations in the aqueous assay buffer present during FLIPR measurements (Na^{+}-free buffer / stimulus buffer, 4:1 + 1% DMSO). Blanks of the used assay buffer were subtracted from the spectra. (B-D) UV-Vis absorption spectra of (B) anthranilic anilides NBA and **118** (PBA), (C) truncated compounds and derivatives **2** – **4**, **7**, **11** – **14**, **17**, **20**, **42** – **43**, **130**, and (C) anthranilic anilides CBA, **86** – **117**, **119** – **128**, **131** measured using the HPLC system; eluents used were either H_2O (+ 0.1% TFA) / ACN (+ 0.1% TFA) or H_2O (+ 0.1% TFA) / THF (+ 0.1% TFA). Gradient of solvent ratio over time and distinct retention times of compounds resulted in various solvent ratios at the point of measurement.

2.2 X-ray Crystal Structures

Table S8. X-ray Structures from Single Crystals of Methyl Anthranilates 6, 7, 42, 70 and 75, Anthranilic Anilides CBA, NBA, 88, 95, 101, 102, 127 and 128.

Compound	X-ray structure (top view)	X-ray structure (side view)
**6**	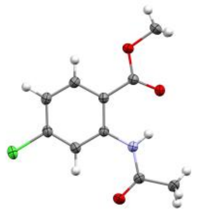	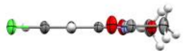

**7**	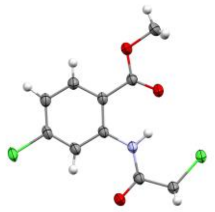	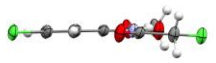
**42**	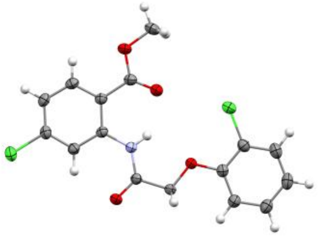	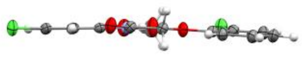
**70**	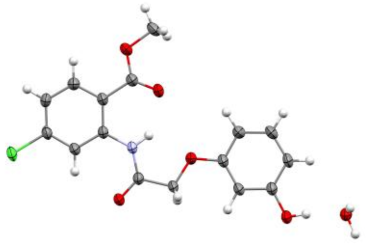	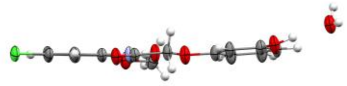
**75**	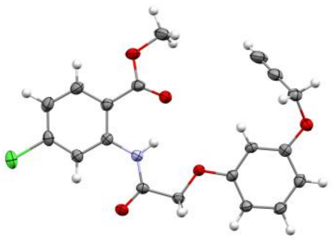	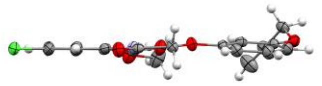
**CBA**	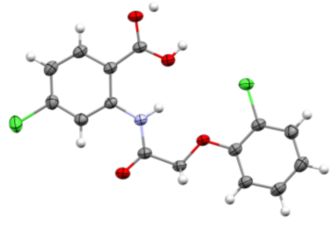	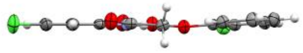
**NBA**	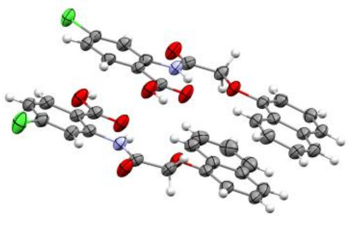	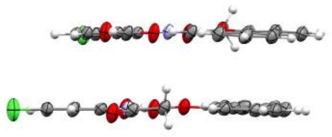

**88**	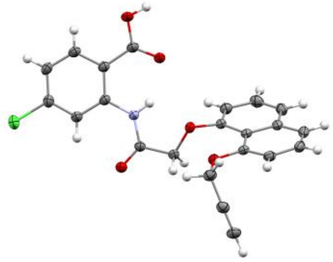	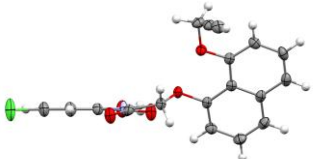
**95**	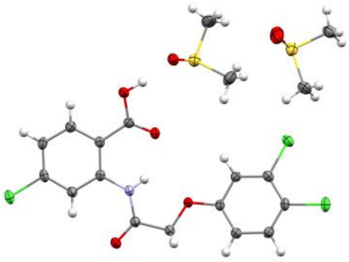	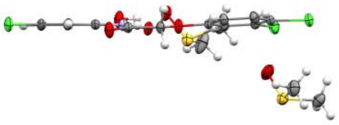
**101**	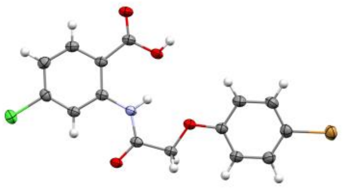	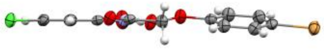
**102**	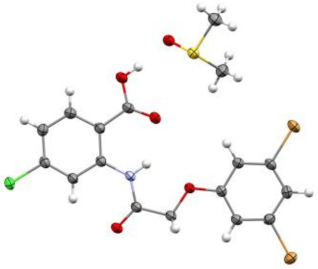	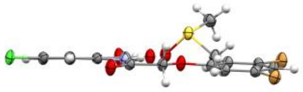
**127**	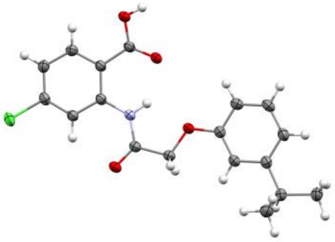	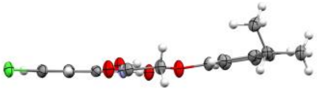
**128**	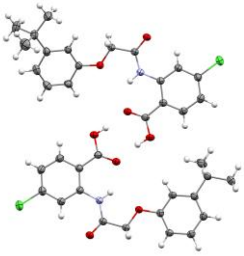	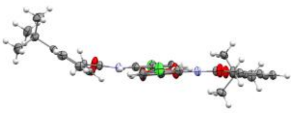

2.3 HPLC Traces and Purities

HPLC chromatogram of **118** (eluent: ACN + 0.1% TFA / THF + 0.1% TFA, gradient):

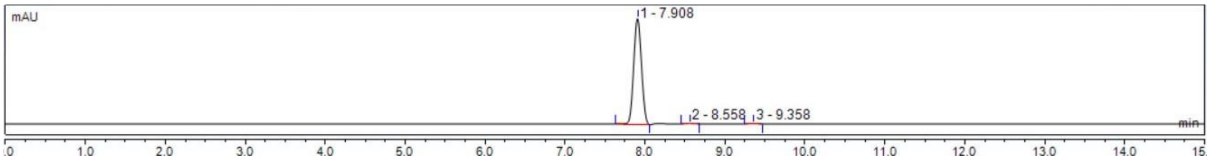

HPLC chromatogram of **127** (eluent: ACN + 0.1% TFA / THF + 0.1% TFA, gradient):

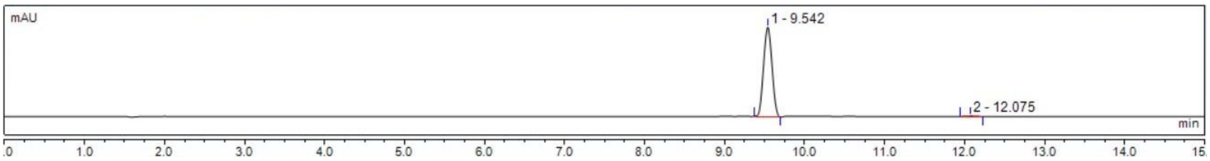

HPLC chromatogram of **123** (eluent: ACN + 0.1% TFA / THF + 0.1% TFA, gradient):

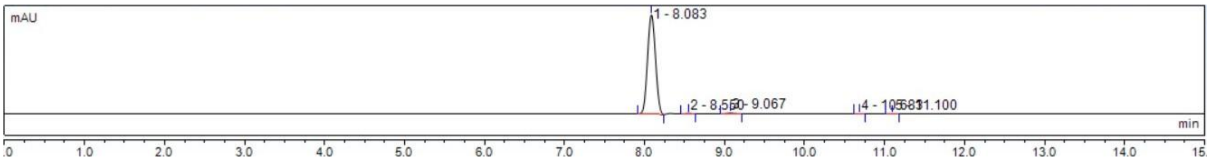

HPLC chromatogram of **105** (eluent: ACN + 0.1% TFA / THF + 0.1% TFA, gradient):

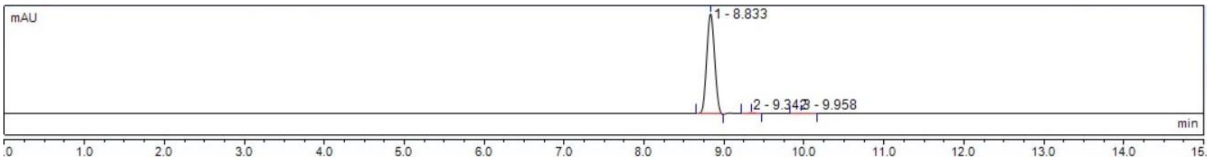

HPLC chromatogram of **89** (eluent: ACN + 0.1% TFA / THF + 0.1% TFA, gradient):

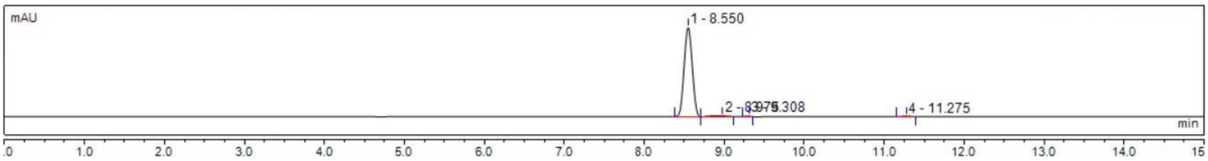

HPLC chromatogram of NBA (eluent: ACN + 0.1% TFA / THF + 0.1% TFA, gradient):

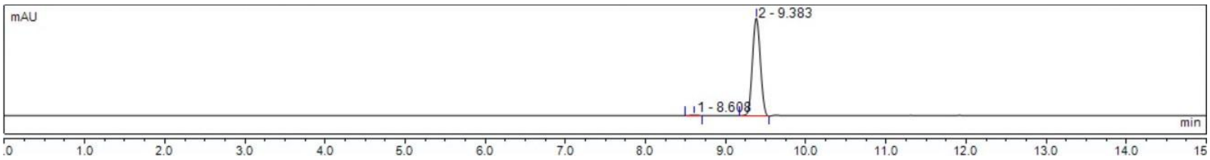

HPLC chromatogram of CBA (eluent: ACN + 0.1% TFA / THF + 0.1% TFA, gradient):

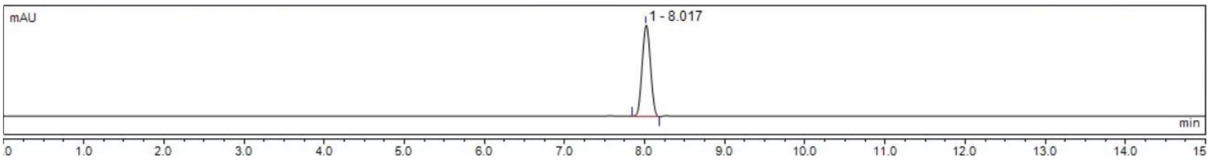

Table S9. HPLC Purities of Final Compounds Used in Biological Evaluations During this Study.

Compound	Purity [%][a]
**2**	> 99
**3**	> 99
**4**	> 99
**7**	> 99
**11**	> 99
**12**	> 99
**13**	> 98
**14**	> 99
**17**	> 99
**20**	> 99
**42**	> 99
**43**	> 99
**86**	> 96
CBA	> 99
NBA	> 99
**87**	> 99
**88**	> 97
**89**	> 97
**90**	> 99
**91**	> 97
**92**	> 98
**93**	> 99
**94**	> 99
**95**	> 99
**96**	> 97
**97**	> 98
**98**	> 98
**99**	> 98
**100**	> 99
**101**	> 99

Compound	Purity [%][a]
**102**	> 98
**103** (IBA)	> 98
**104**	> 99
**105**	> 99
**106**	> 95
**107**	> 99
**108**	> 97
**109**	> 97
**110**	> 97
**111**	> 99
**112**	> 99
**113**	> 99
**114**	> 99
**115**	> 99
**116**	> 99
**122**	> 97
**117**	> 98
**118** (PBA)	> 99
**119**	> 98
**120**	> 98
**121**	> 97
**123**	> 99
**124**	> 99
**125**	> 98
**126**	> 99
**127**	> 99
**128**	> 99
**130**	> 99
**131**	> 98

[a]Purities of compounds in % were determined using HPLC measurements. Blank run traces were subtracted from sample run traces and absorption peaks at λ_{abs} = 254 nm of compounds and impurities were integrated using the Chromeleon 7 software.

2.4 NMR Data for Conformational Analysis

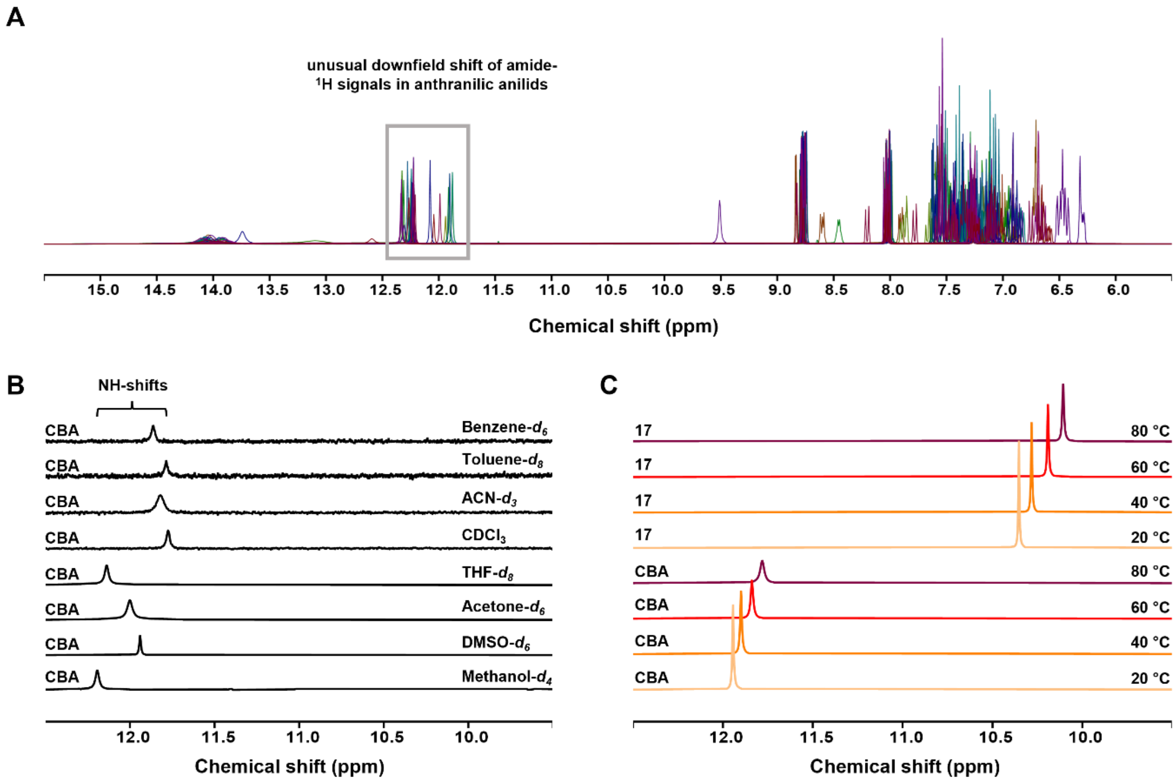

Figure S20. (A) Overlay of ^{1}H-NMR spectra from anthranilic anilide final compounds measured in DMSO-d_6 with highlighted amide-^{1}H signals (grey box). (B) Amide ^{1}H chemical shifts of CBA measured in MeOH-d_4, DMSO-d_6, acetone-d_6, THF-d_8, $CDCl_3$, ACN-d_3, toluene-d_8, and benzene-d_6. (C) Amide ^{1}H chemical shifts of CBA and **17** measured in DMSO-d_6 at 20 °C, 40 °C, 60 °C and 80 °C.

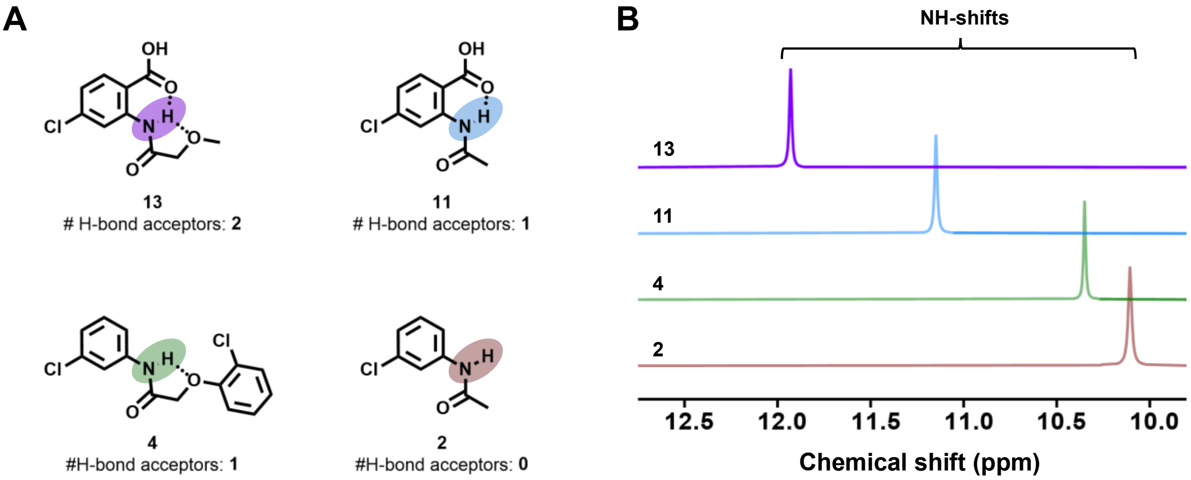

Figure S21. (A) Compounds with various numbers of hydrogen bond acceptors in the vicinity of the amide hydrogen. **13** with two H-bond acceptors, **11** with a carboxyl-oxygen as the only H-bond acceptor, **4** with a phenoxy oxygen as the only H-bond acceptor and **2** with no H-bond acceptors. (B) ^{1}H-NMR shifts (ppm) of the amide hydrogen in DMSO-d_6 observed for the compounds **13**, **11**, **4** and **2** with various numbers of hydrogen bond acceptors.

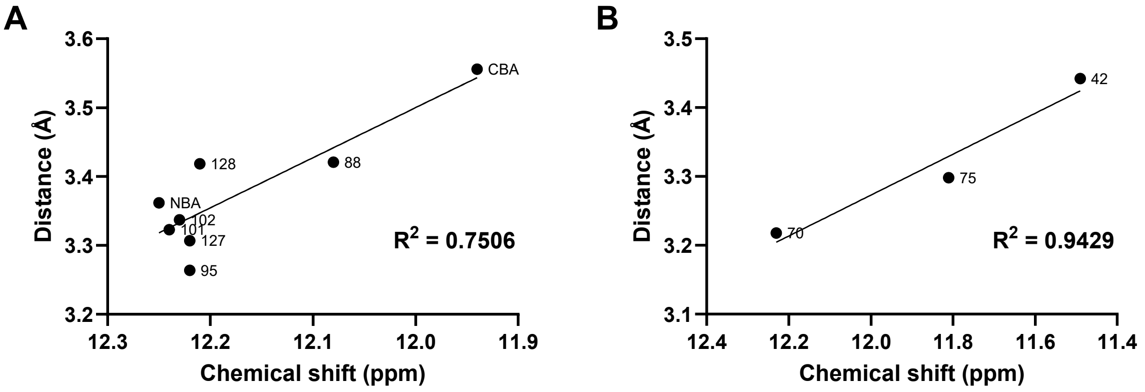

Figure S22. (A) Correlation ($R^2 = 0.7506$) of atomic distances from crystal structures between the phenoxy-oxygens and the carboxyl-oxygens with the corresponding amide ^{1}H chemical shifts for NBA, CBA, **88**, **95**, **101**, **102**, **127** and **128**. (B) Correlation ($R^2 = 0.9429$) of atomic distances from crystal structures between the phenoxy-oxygens and the carboxyl-oxygens with the corresponding amide ^{1}H chemical shifts for **42**, **70** and **75**.

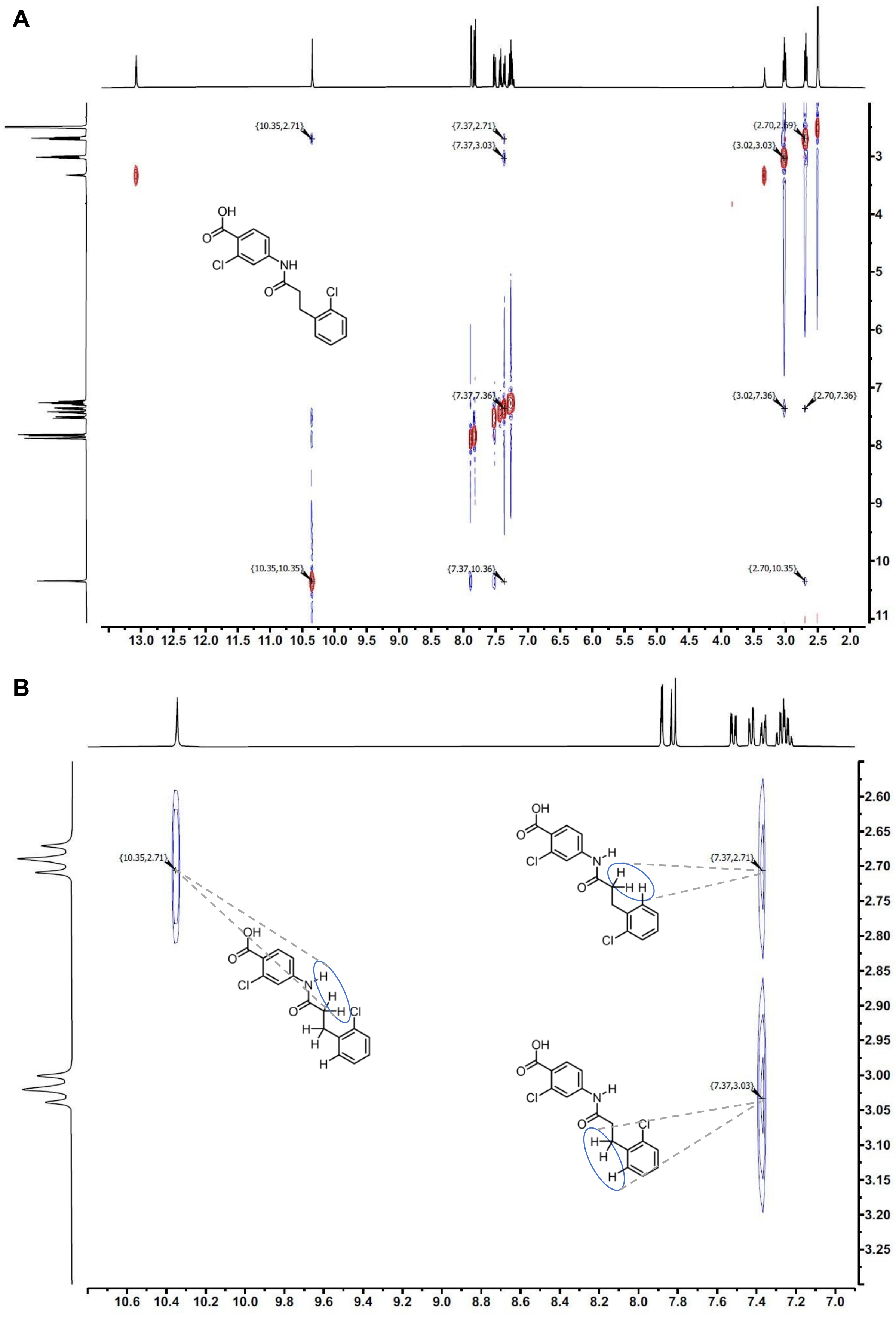

Figure S23. (A) ^{1}H/^{1}H-NOESY spectrum of **17** measured in DMSO-d_6. (B) Magnification of the spectral area showing the strong couplings of CαH$_2$ and CβH$_2$ with aromatic-H as well as the strong coupling of C$_\alpha$H$_2$ and with amide-H.

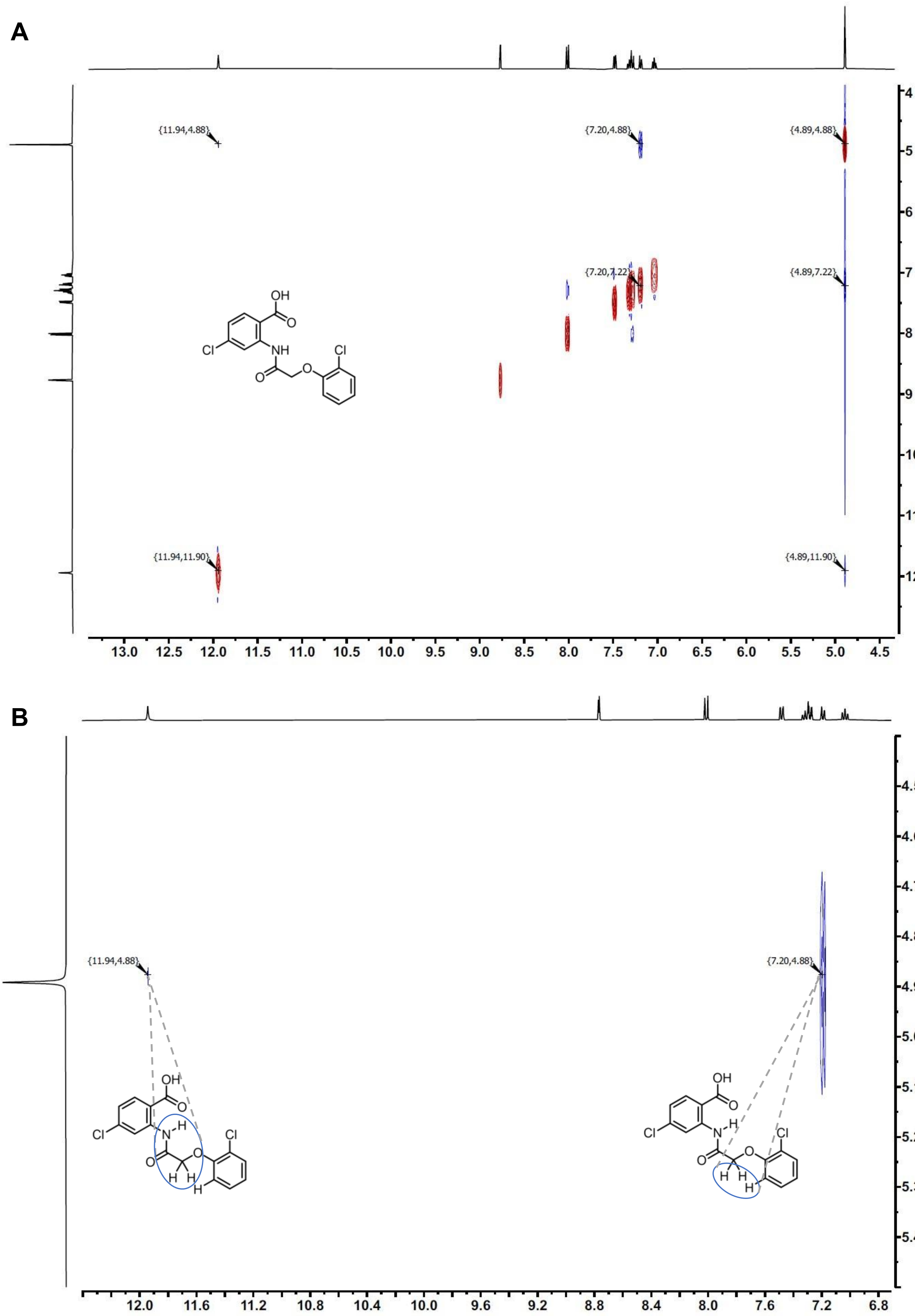

Figure S24. (A) ^{1}H/^{1}H-NOESY spectrum of CBA measured in DMSO-*d*6. (B) Magnification of the spectral area showing the strong coupling of $C_{\alpha}H_2$ with aromatic-H and the weak coupling of $C_{\alpha}H_2$ with amide-H.

2.5 Molecular Docking

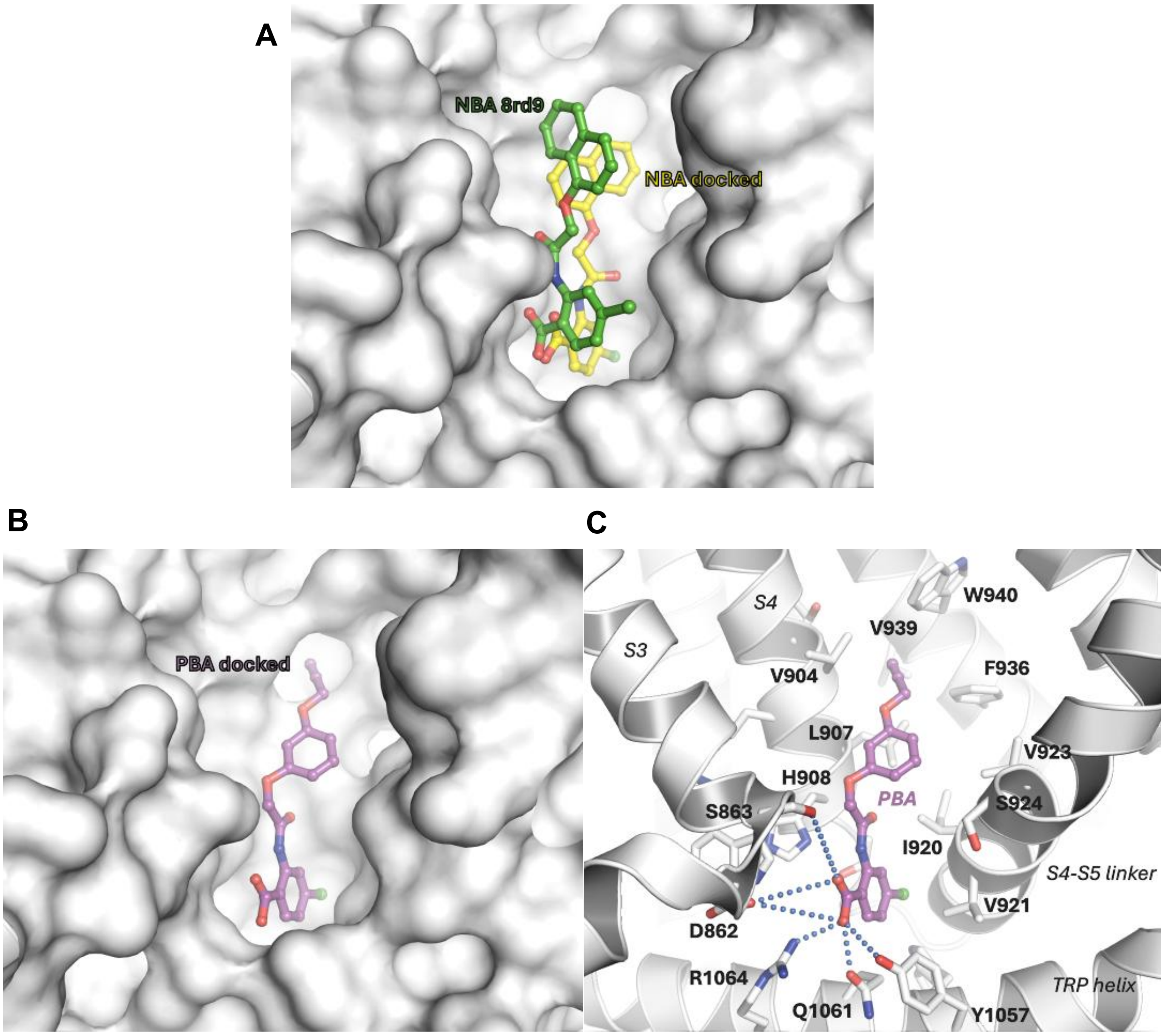

Figure S25. Docking of PBA (**118**) into human TRPM4. Protein shown as white surface or cartoon, respectively, and ligands as ball-and-stick models. (A) NBA was docked into hTRPM4 complexed with NBA (PDB ID: 8RD9) for validation of the docking conditions. Predicted binding pose of NBA in yellow, NBA binding pose deduced from cryo-EM density maps in green as reference. (B) Predicted binding pose of PBA (**118**) in TRPM4. The terminal alkyne points into a "hydrophobic hole". (C) Details of the predicted PBA binding pose. Residues within 5Å of bound ligand are shown as sticks, potential hydrogen or ionic bonds as blue dots. The carboxylate of PBA forms interactions with several polar functional groups (from D862, S863, Y1057, Q1061, R1064), the phenoxy ring is surrounded by hydrophobic residues (L907, I920, V923) and the terminal alkyne is located in a tunnel lined with hydrophobic residues (V904, F936, V393, W940). Figure was generated using PyMOL 3.1.3.1.

2.6 ^{1}H- and ^{13}C-NMR Spectra

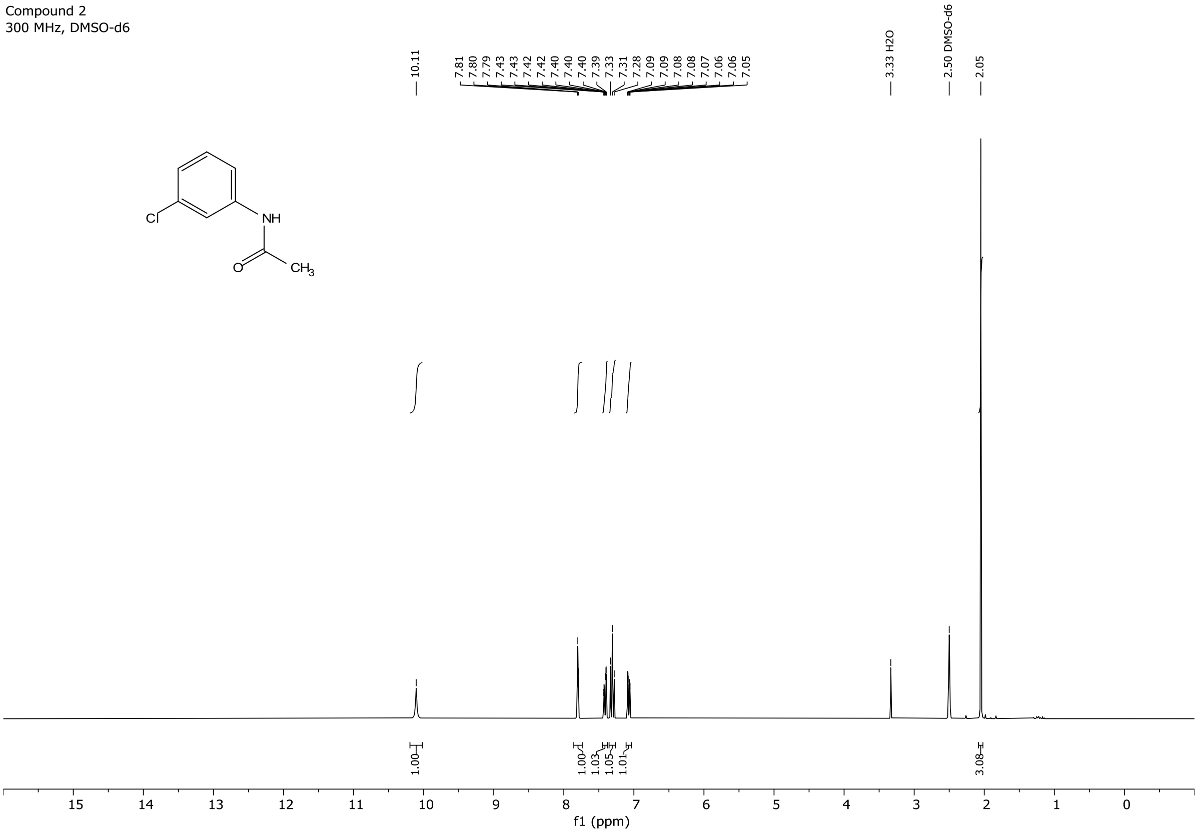

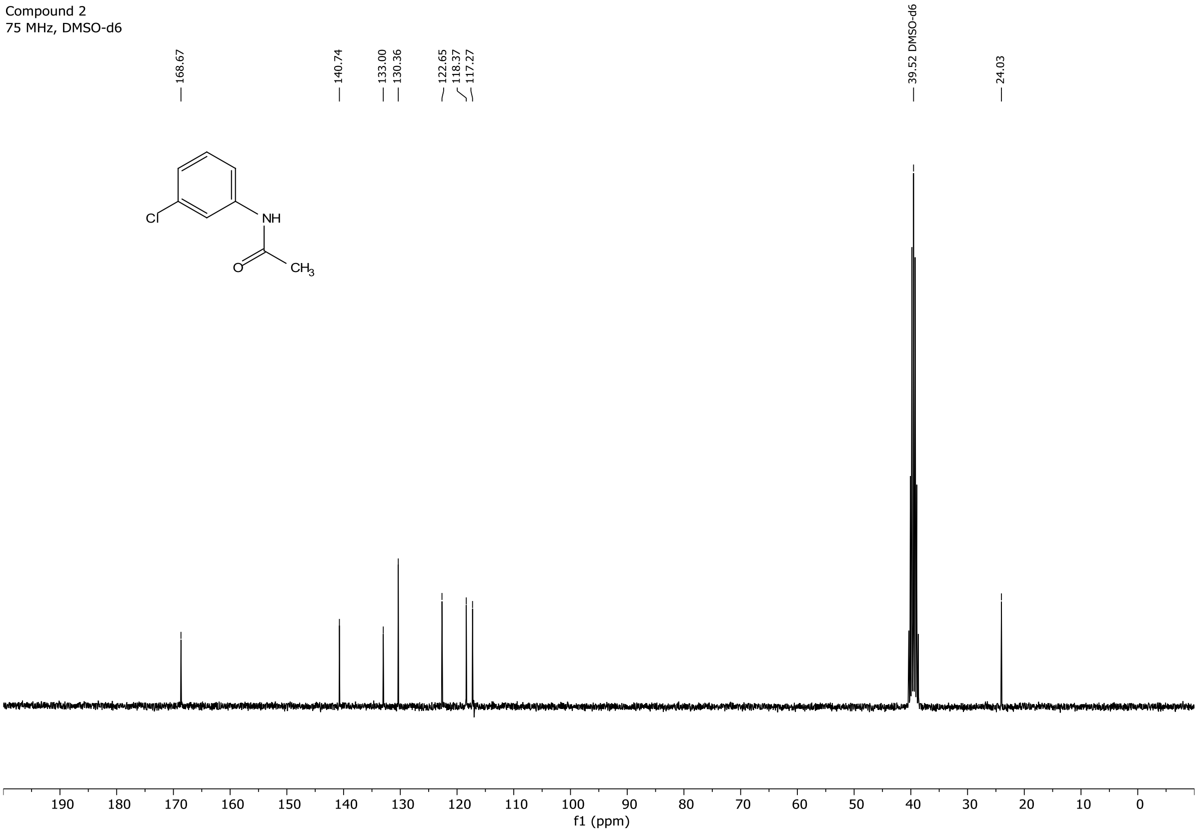

Compound 3
300 MHz, DMSO-d6

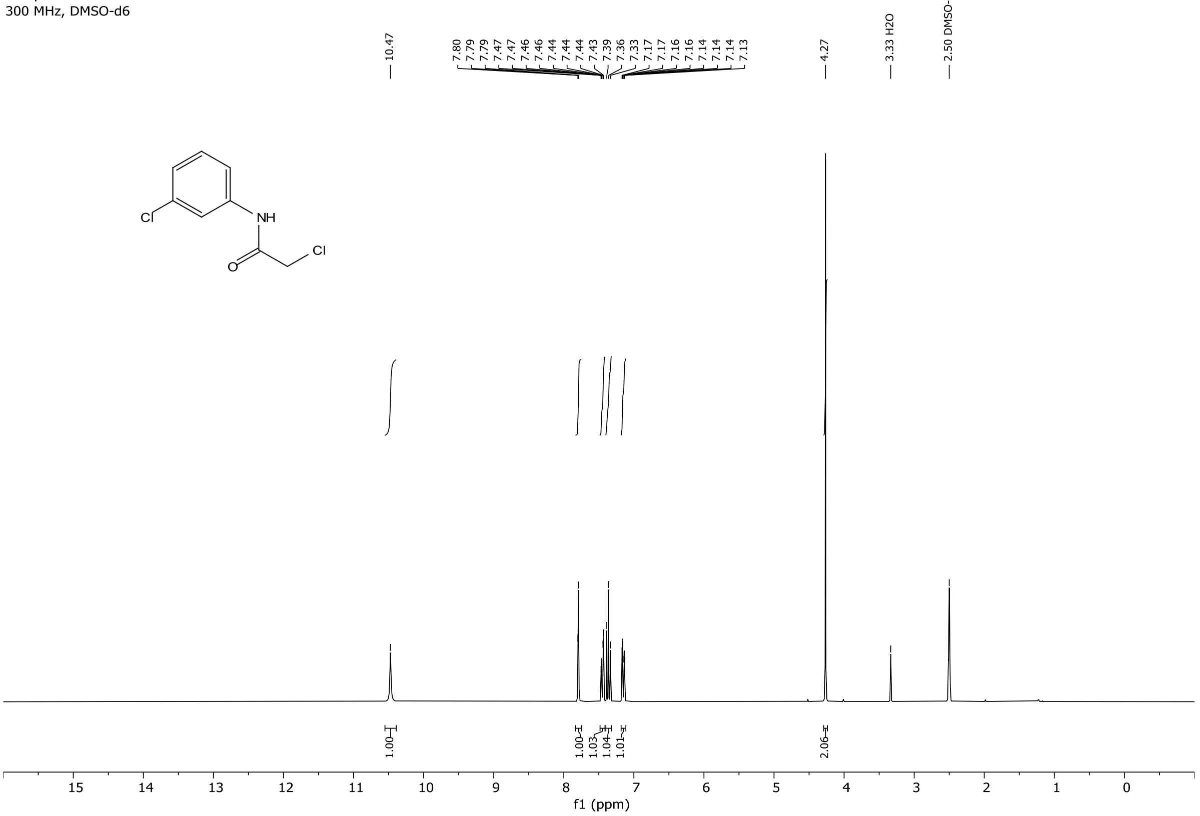

Compound 3
75 MHz, DMSO-d6

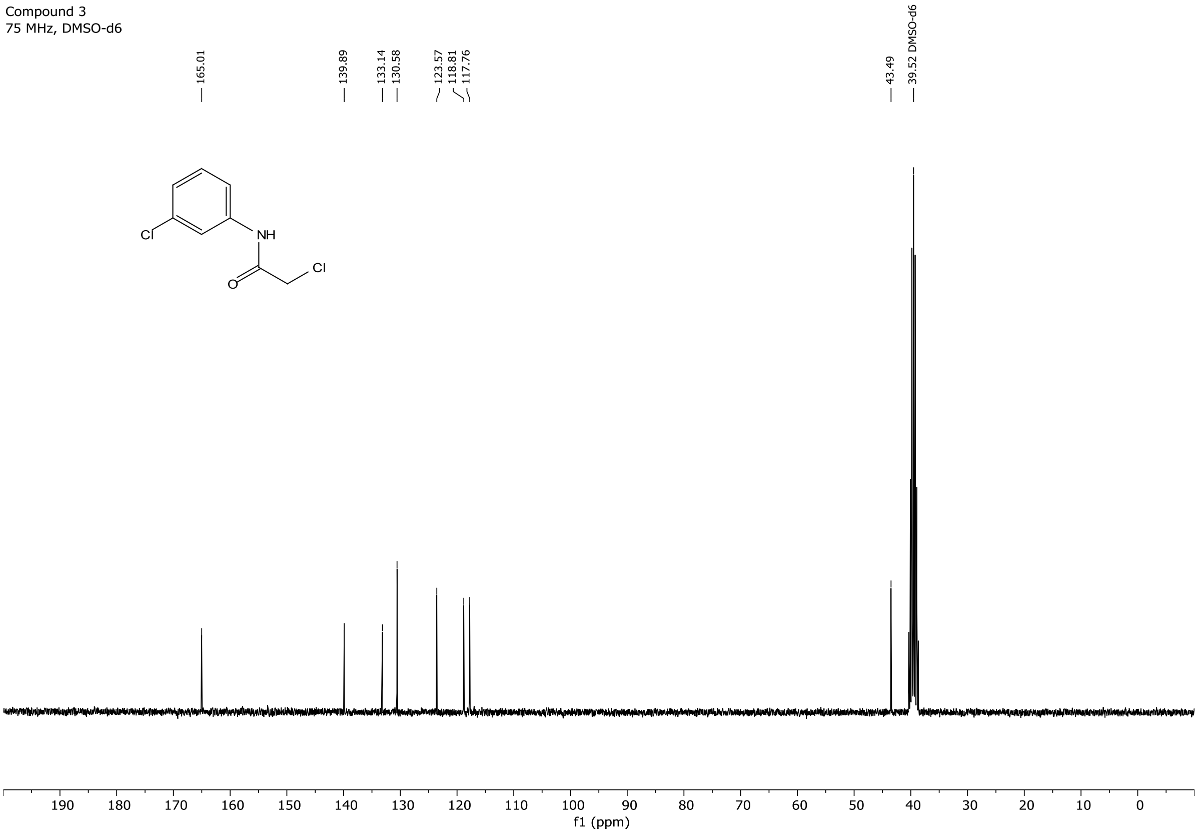

Compound 4
300 MHz, DMSO-d6

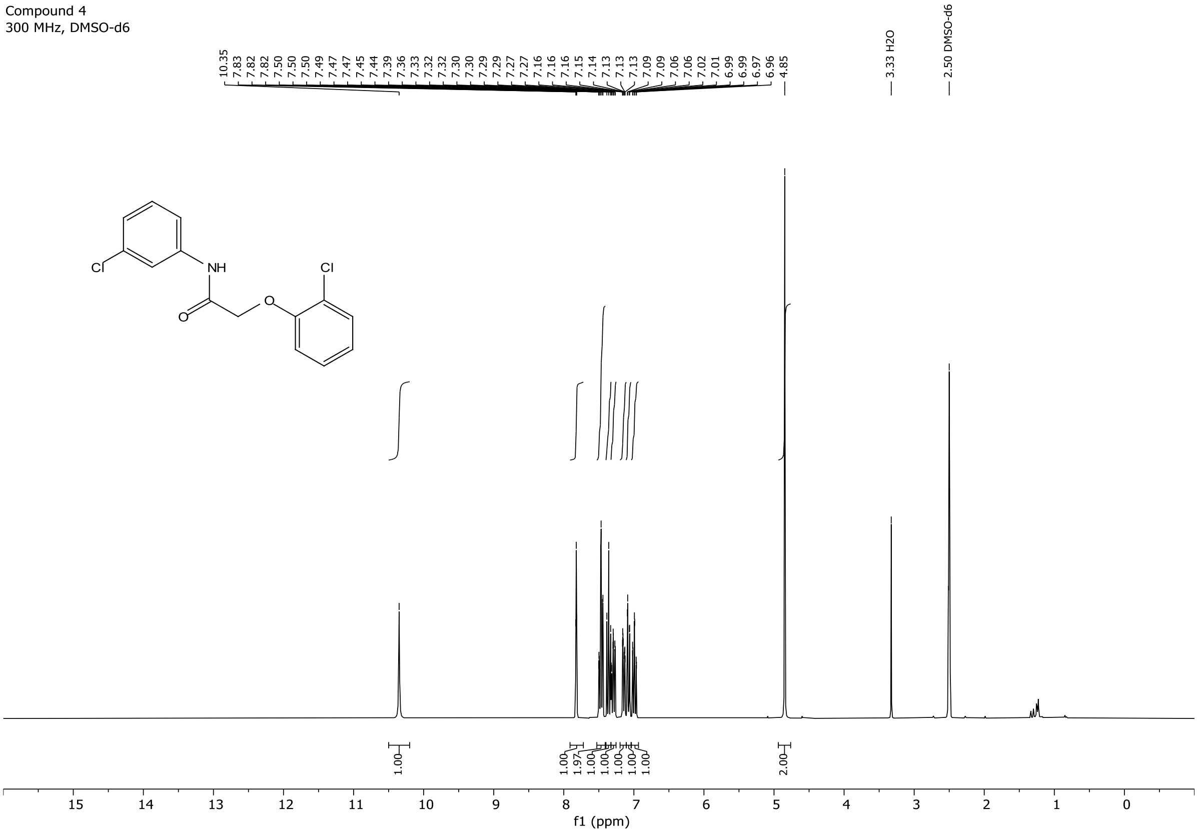

Compound 4
75 MHz, DMSO-d6

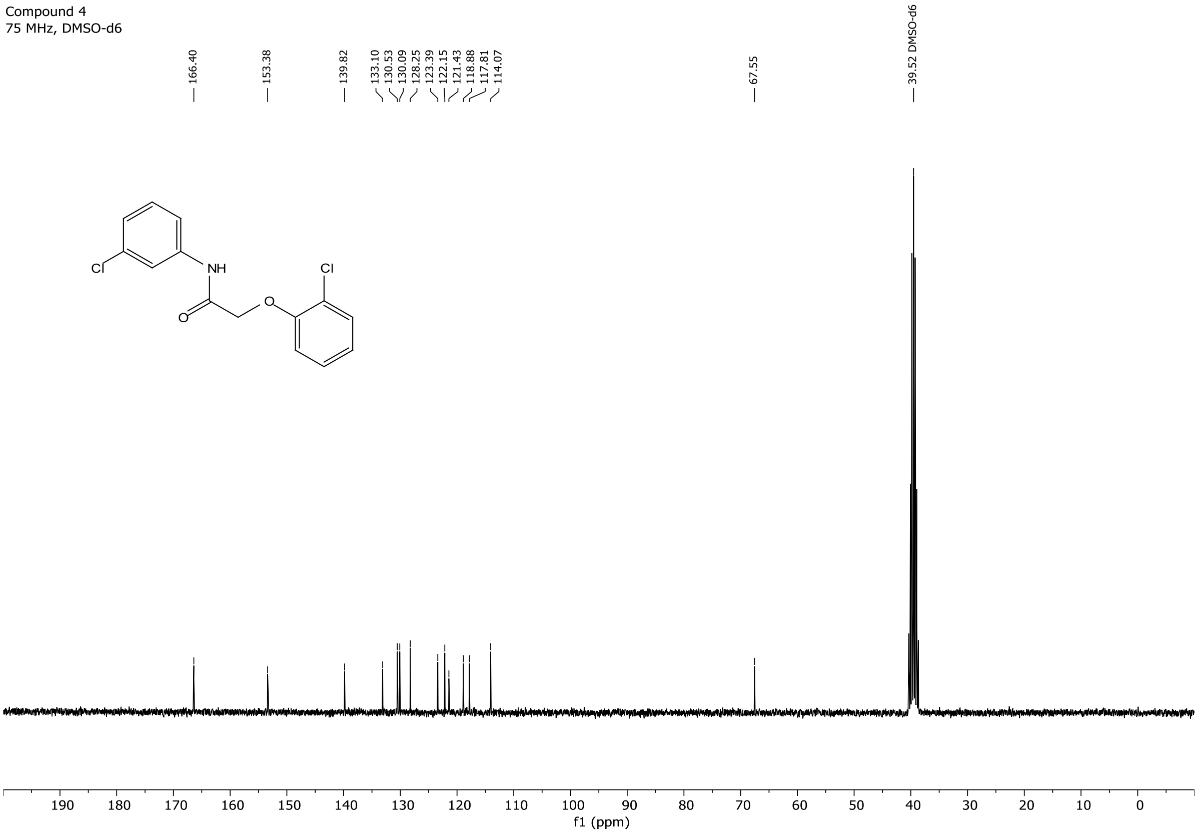

Compound 11
300 MHz, DMSO-d6

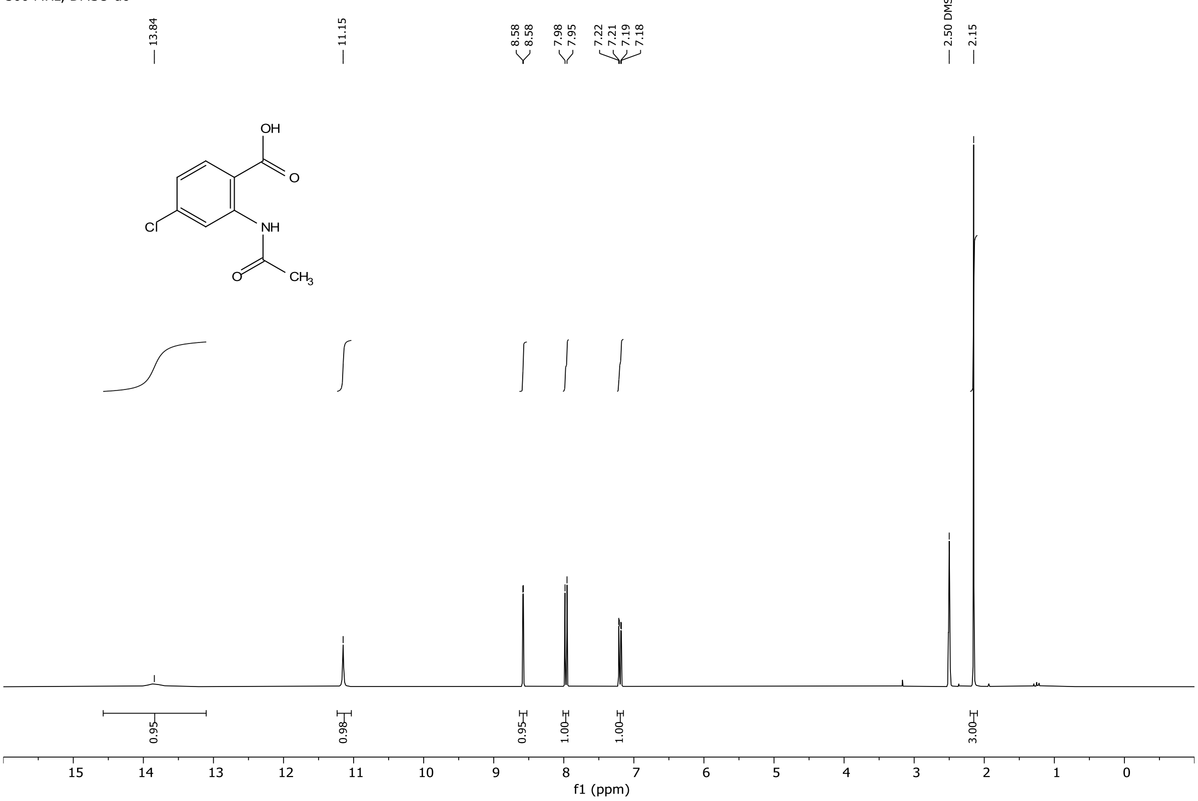

Compound 11
75 MHz, DMSO-d6

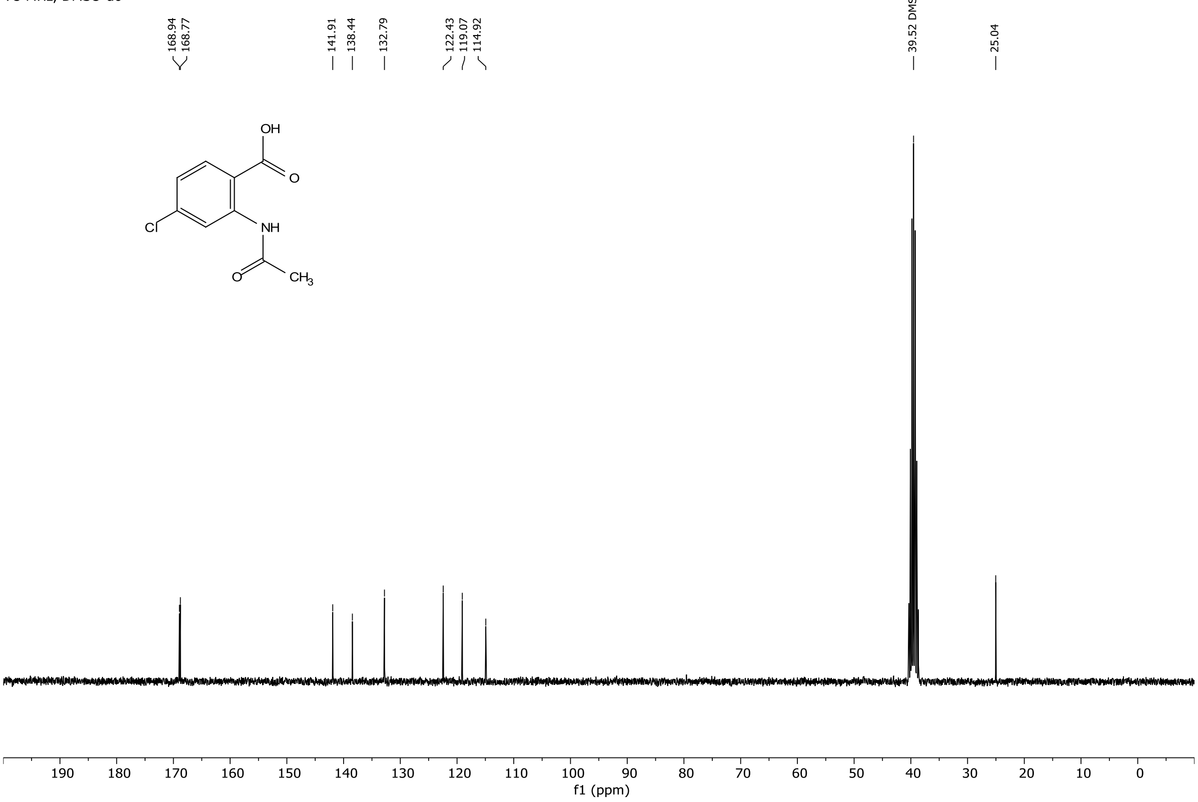

Compound 12
300 MHz, DMSO-d6

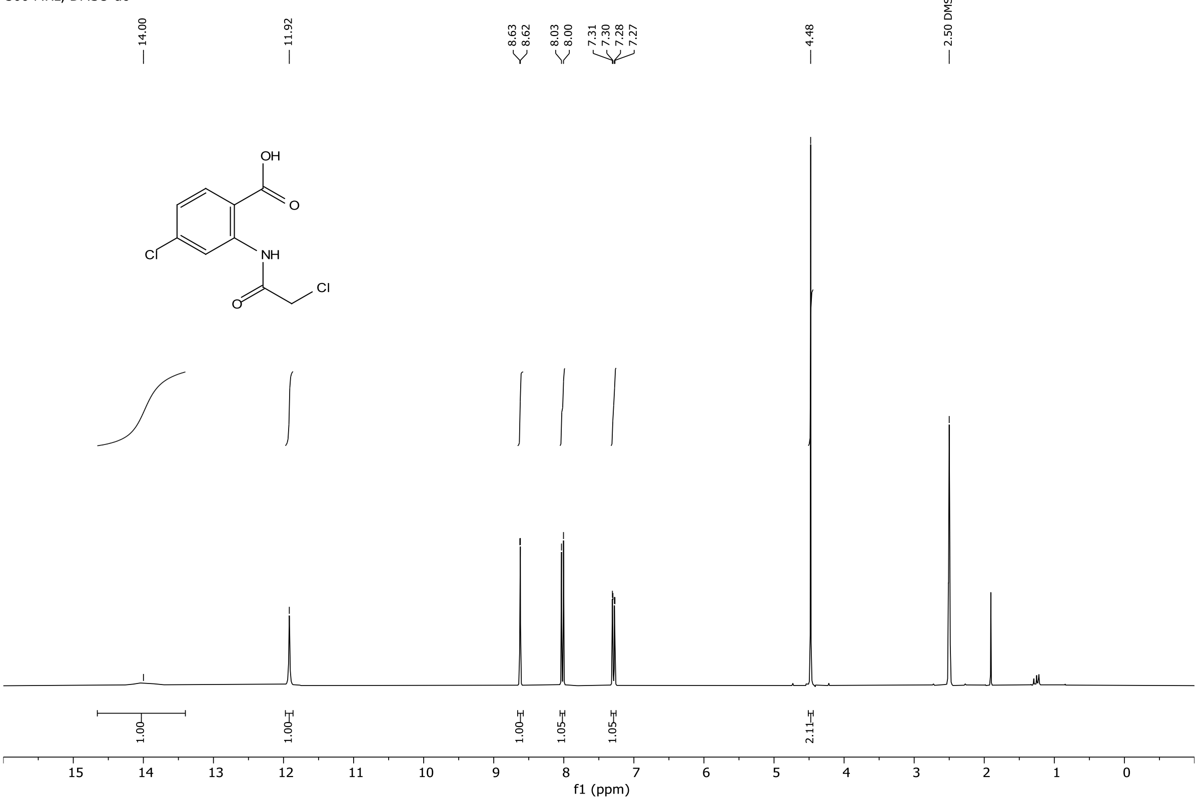

Compound 12
75 MHz, DMSO-d6

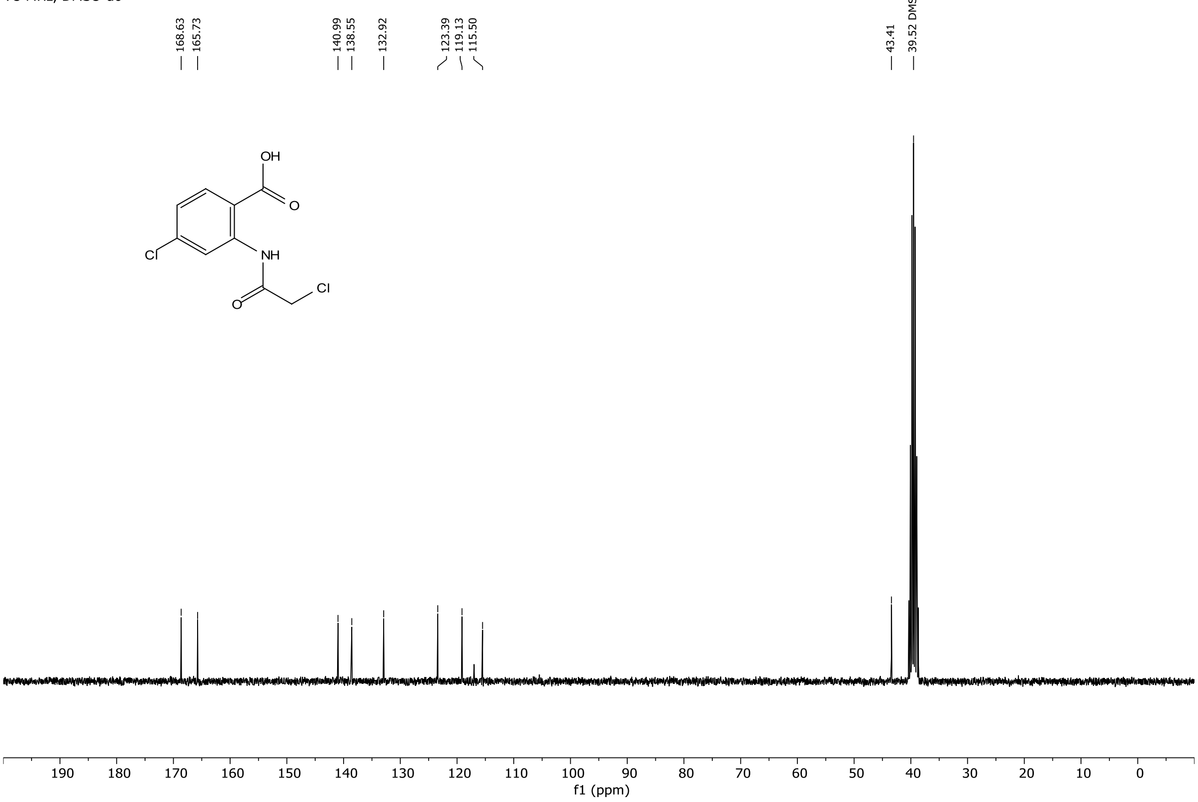

Compound 13
300 MHz, DMSO-d6

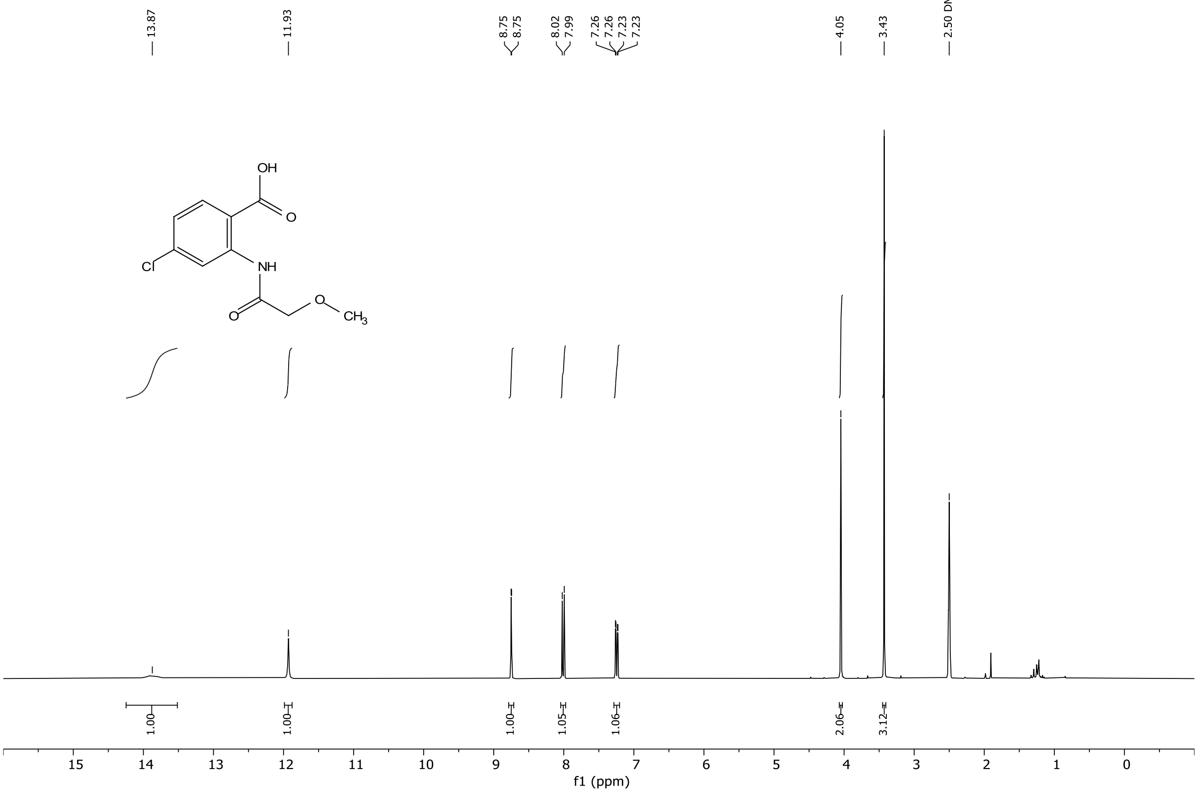

Compound 13
75 MHz, DMSO-d6

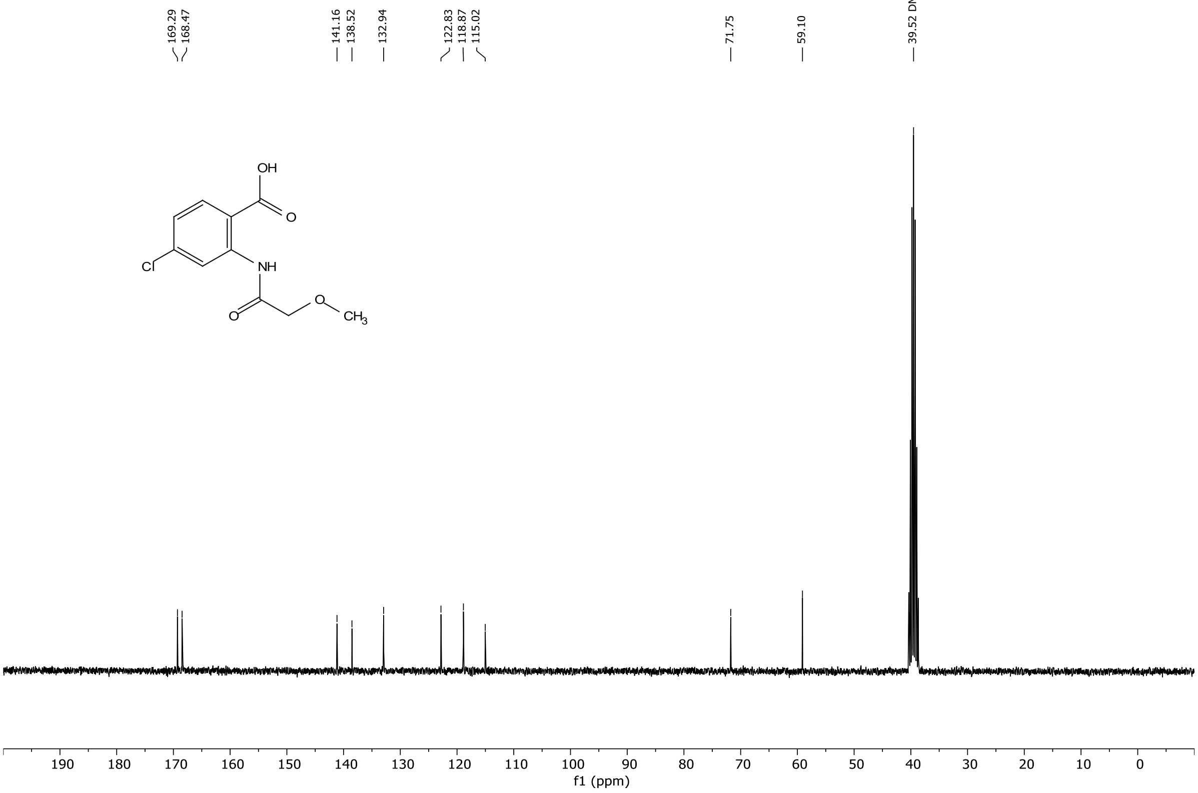

Compound 17
300 MHz, DMSO-d6

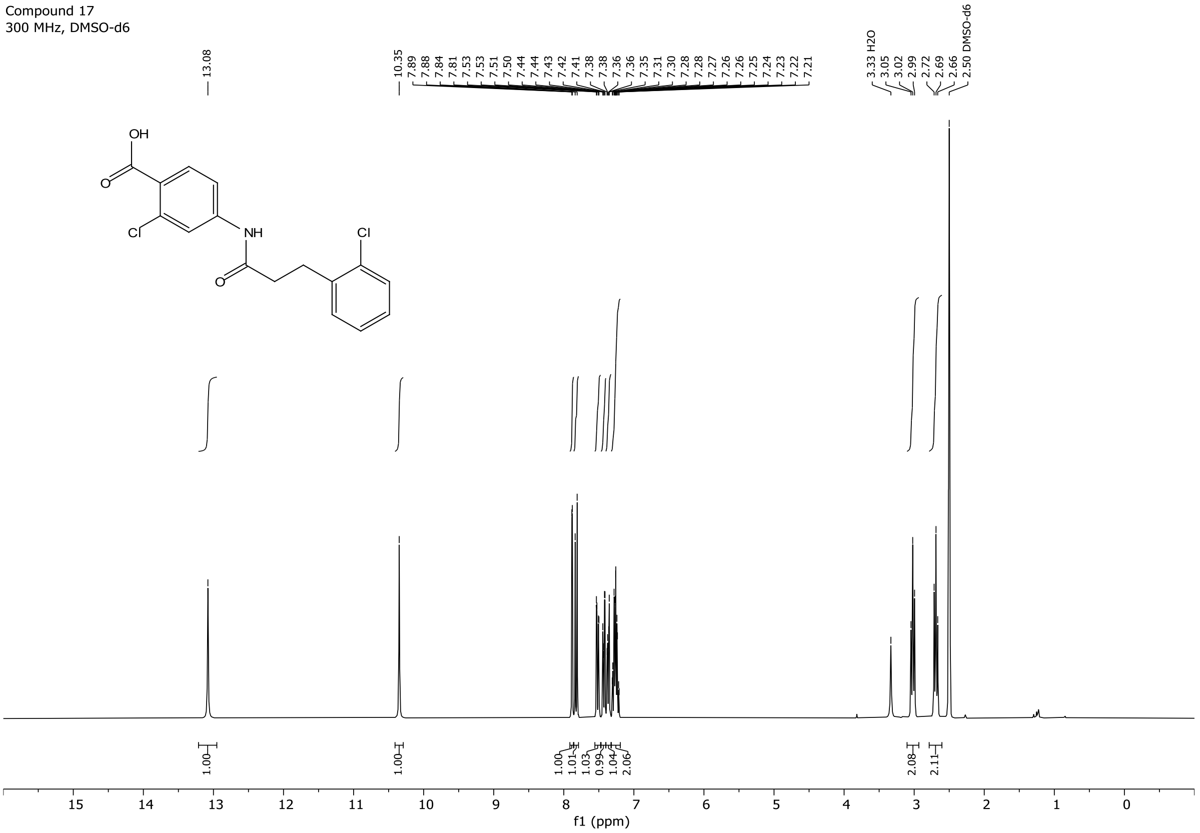

Compound 17
75 MHz, DMSO-d6

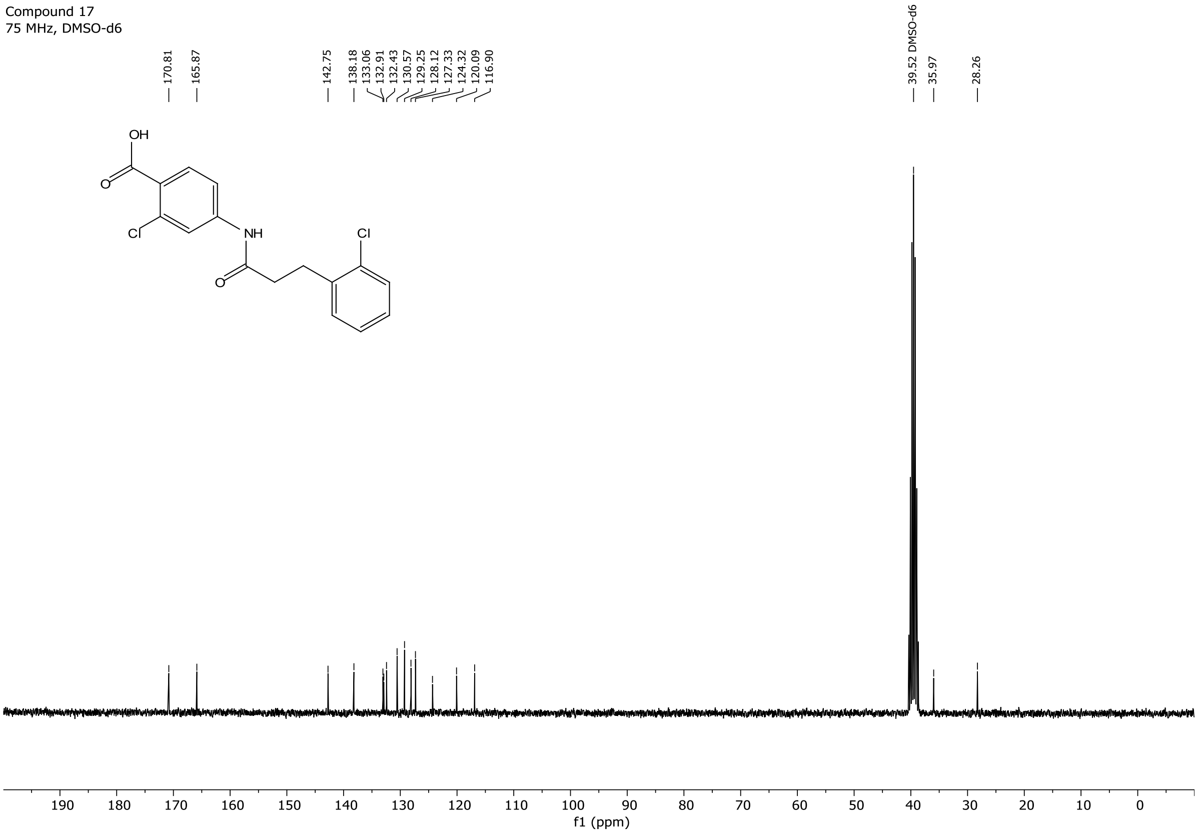

Compound 86
300 MHz, DMSO-d6

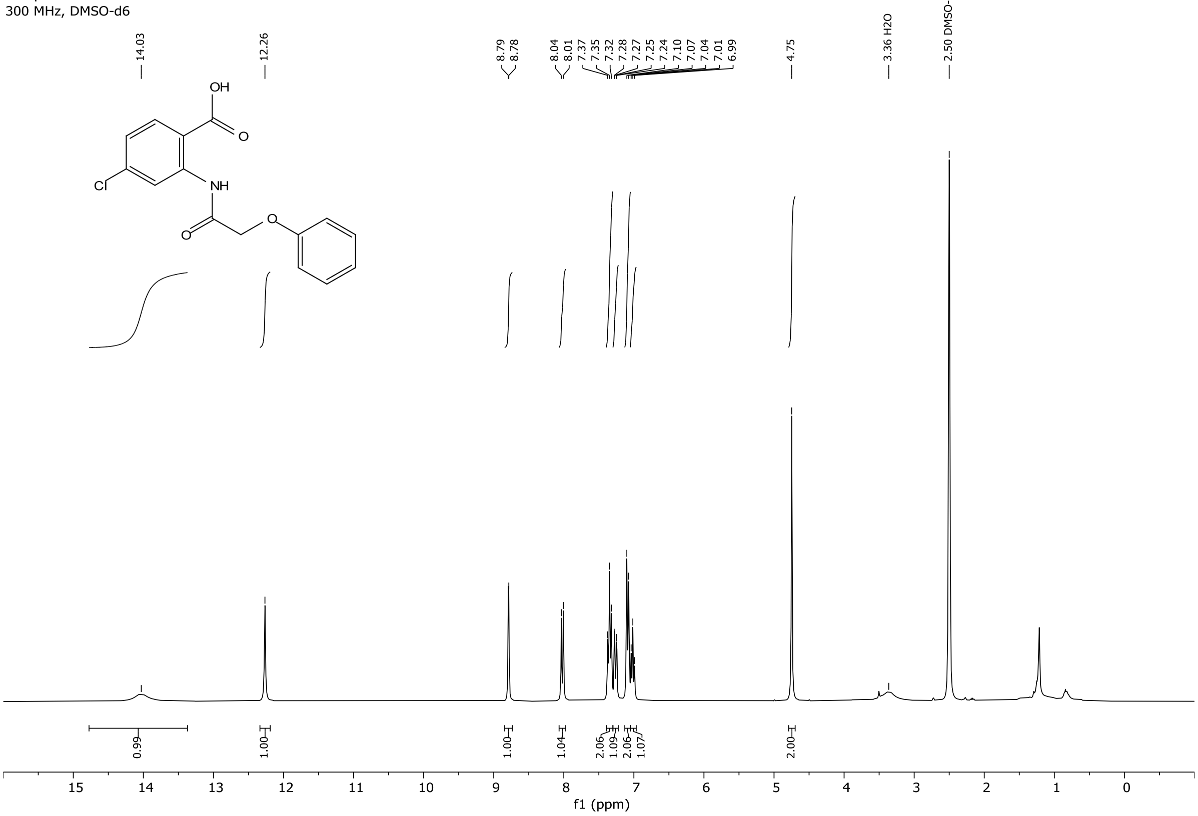

Compound 86
75 MHz, DMSO-d6

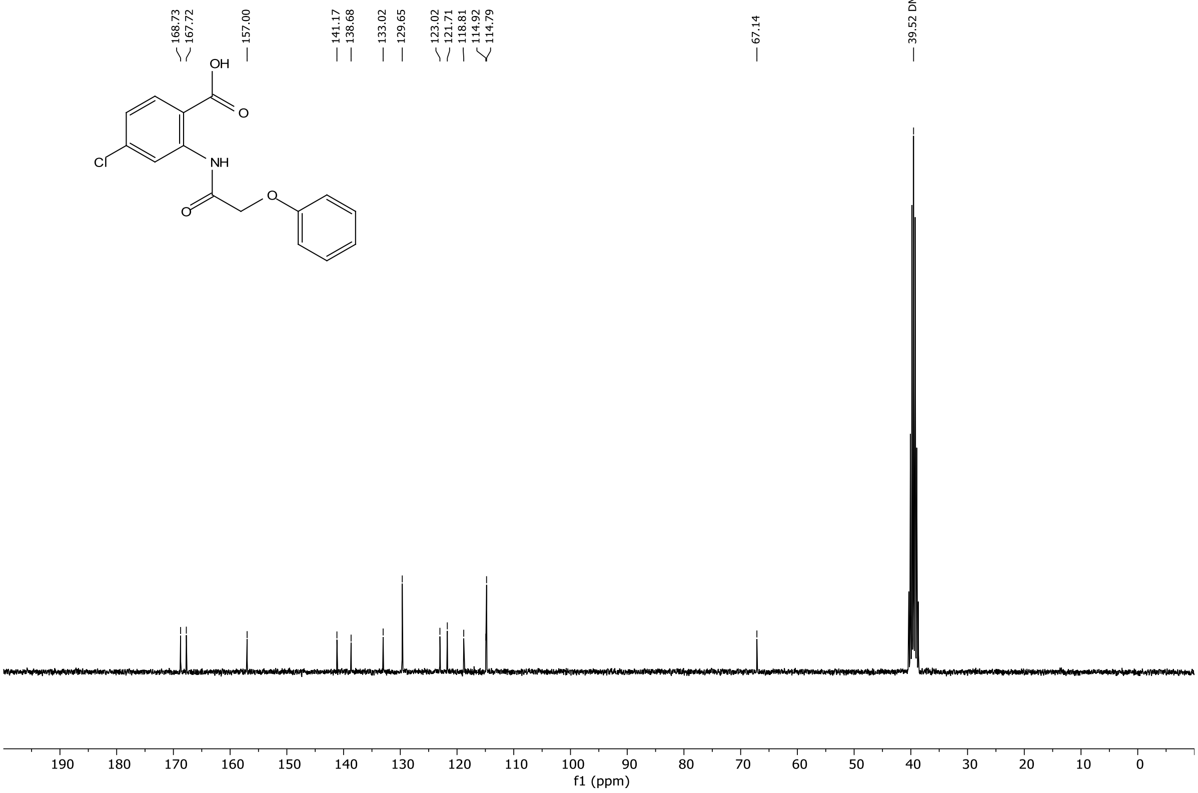

Compound 87
300 MHz, DMSO-d6

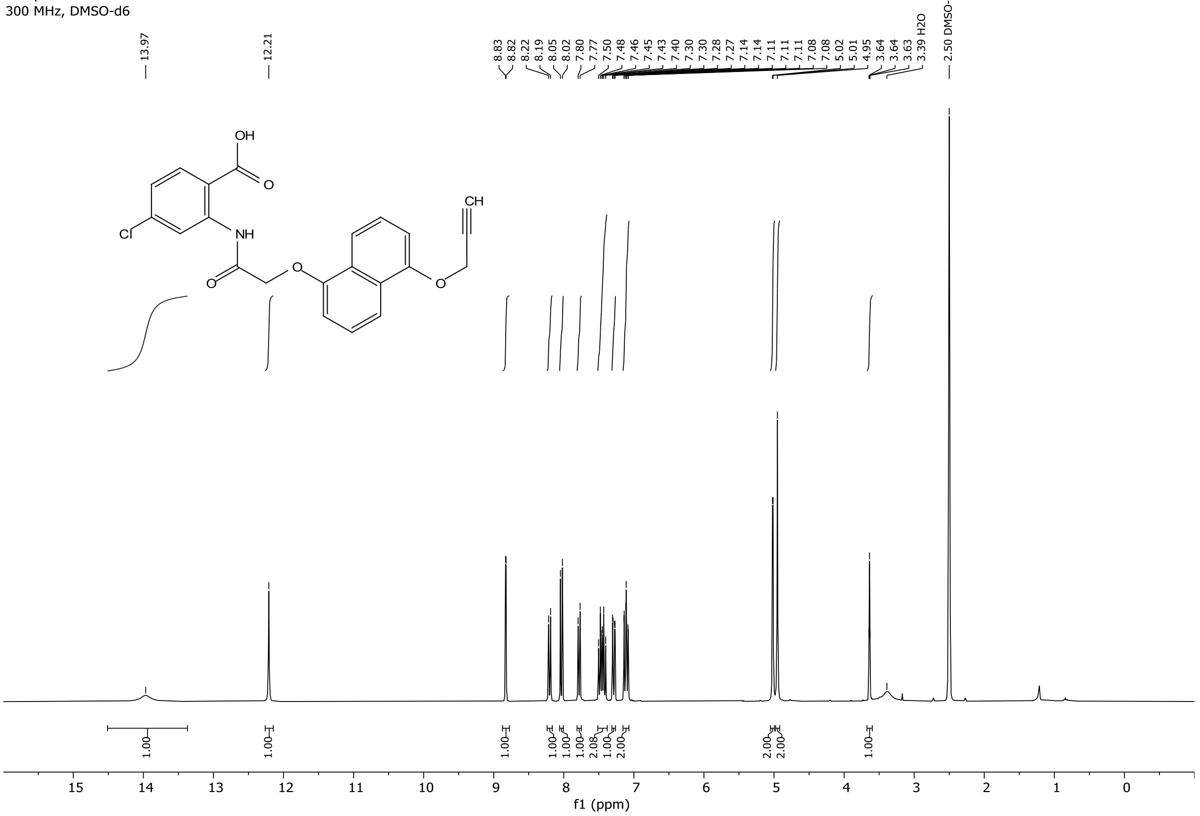

Compound 87
75 MHz, DMSO-d6

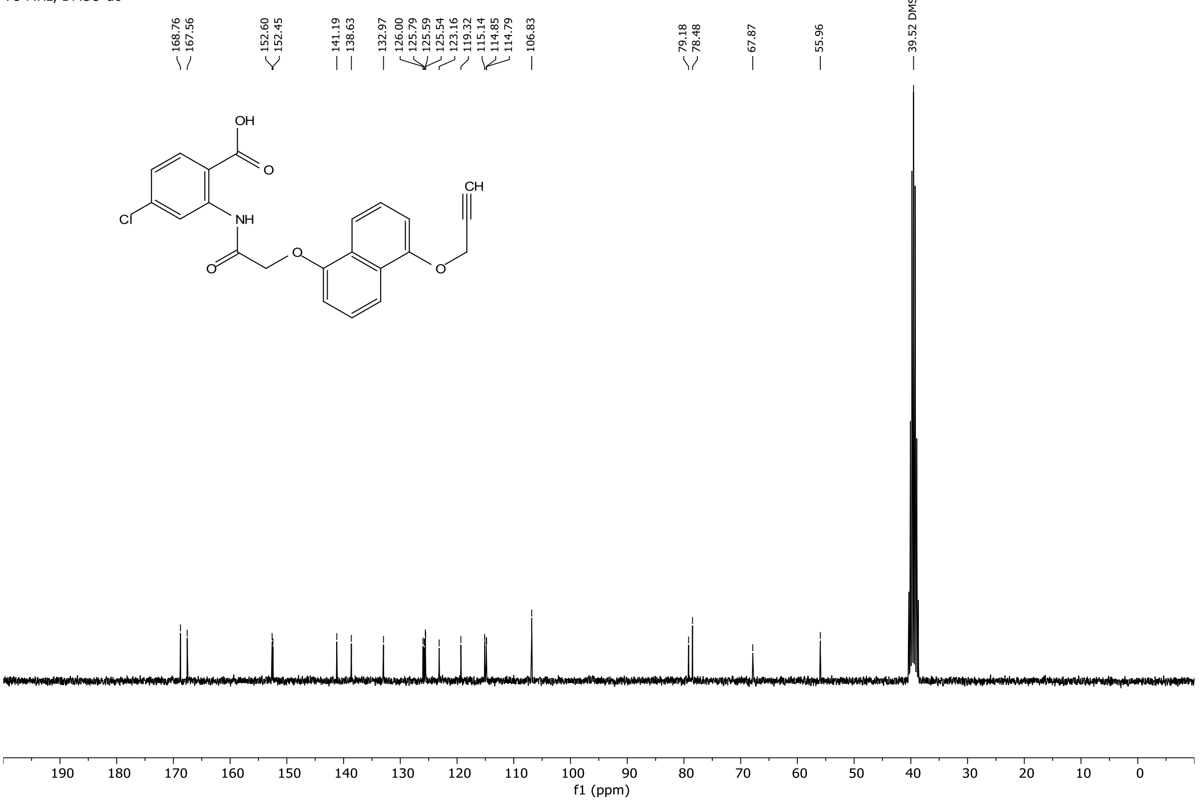

Compound 88
300 MHz, DMSO-d6

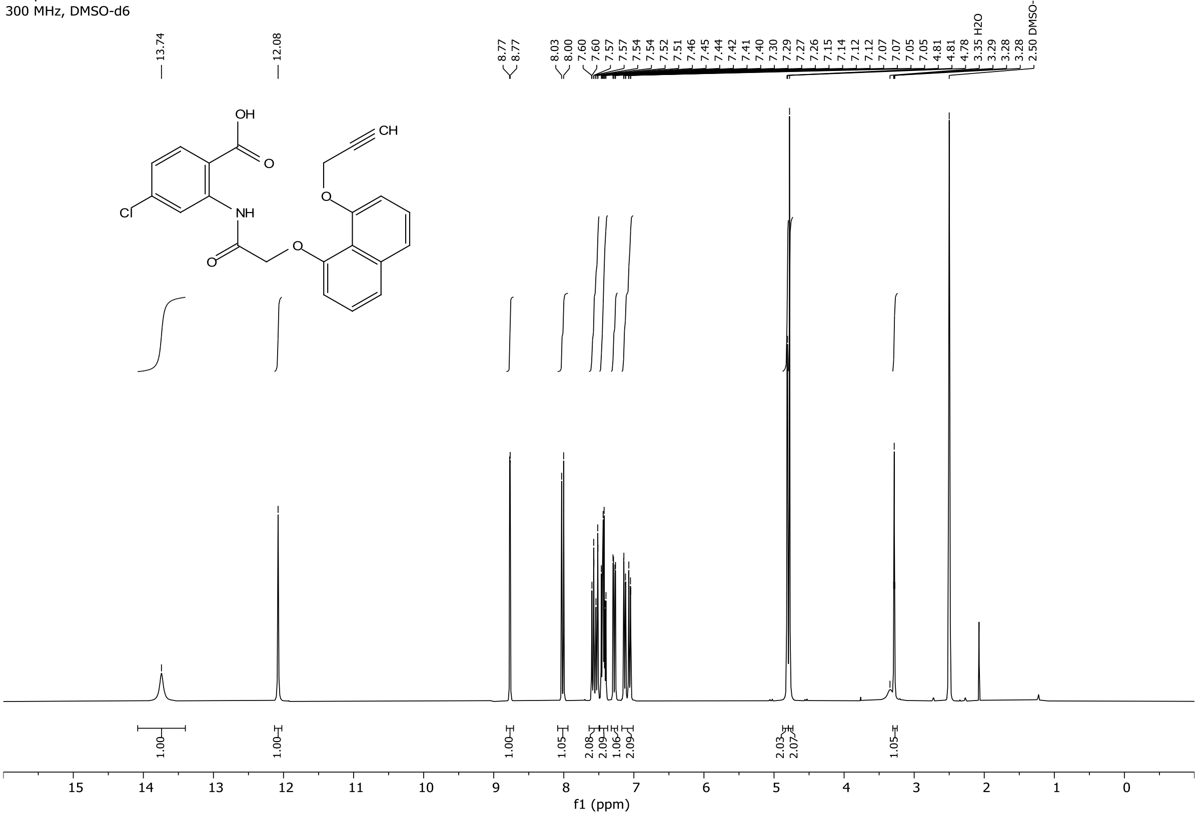

Compound 88
75 MHz, DMSO-d6

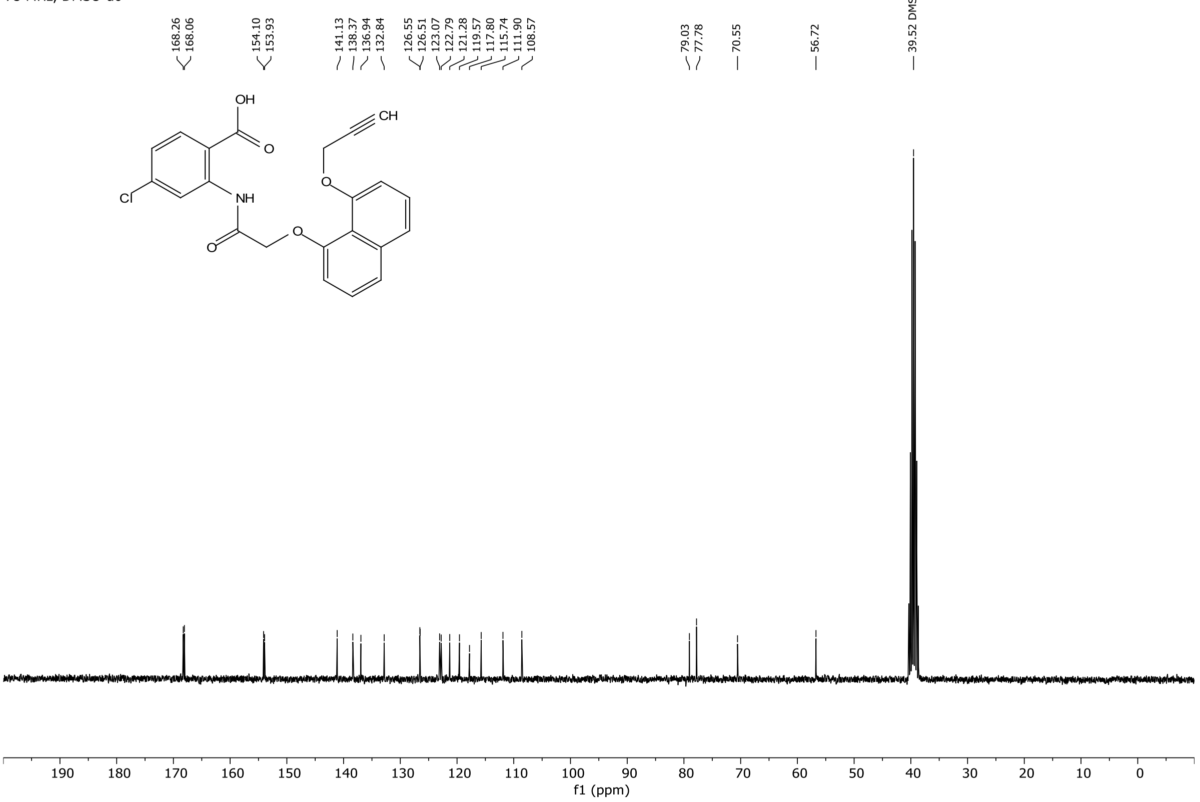

Compound 89
300 MHz, DMSO-d6

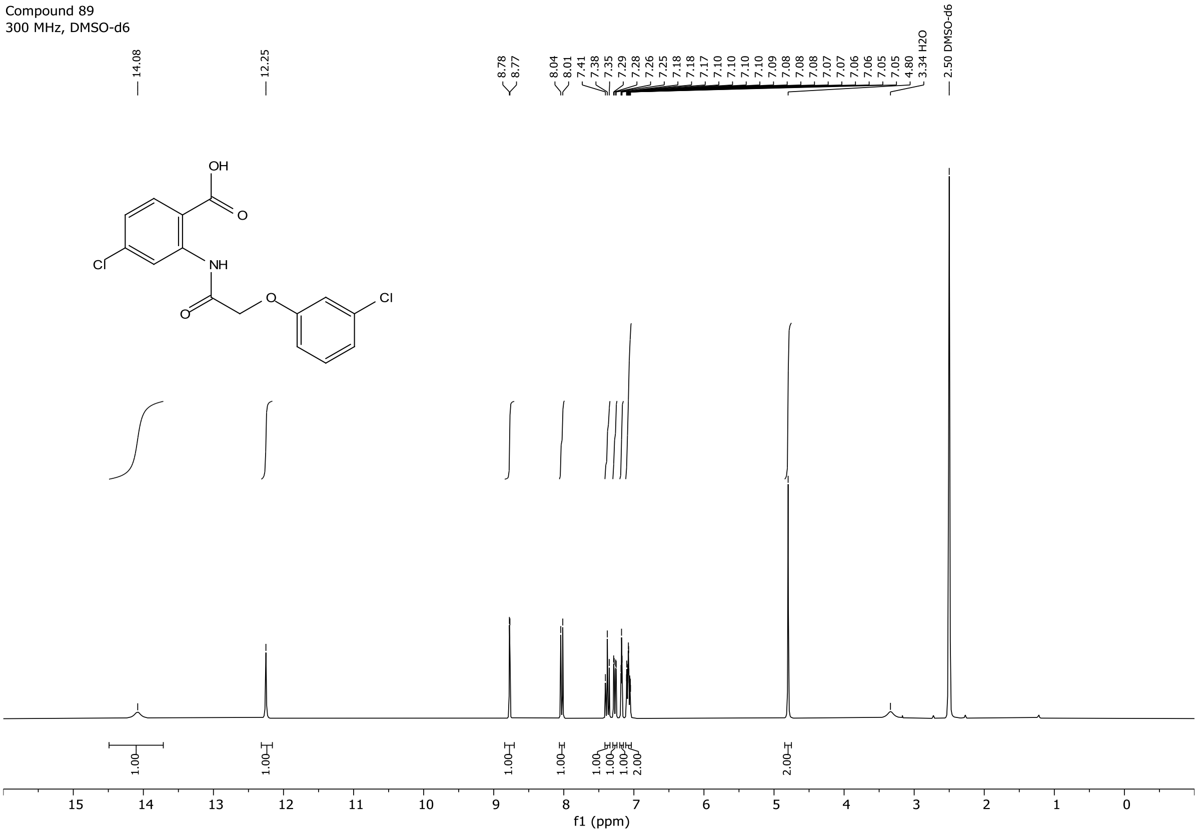

Compound 89
75 MHz, DMSO-d6

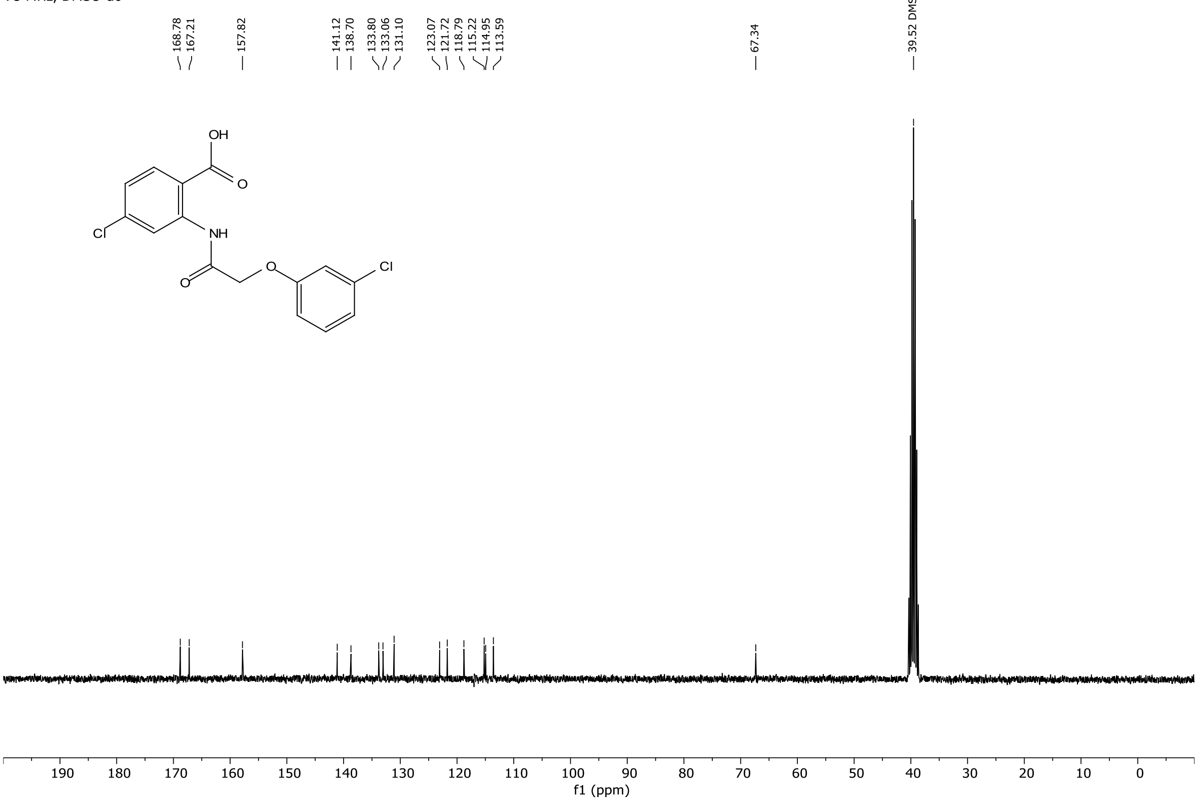

Compound 90
300 MHz, DMSO-d6

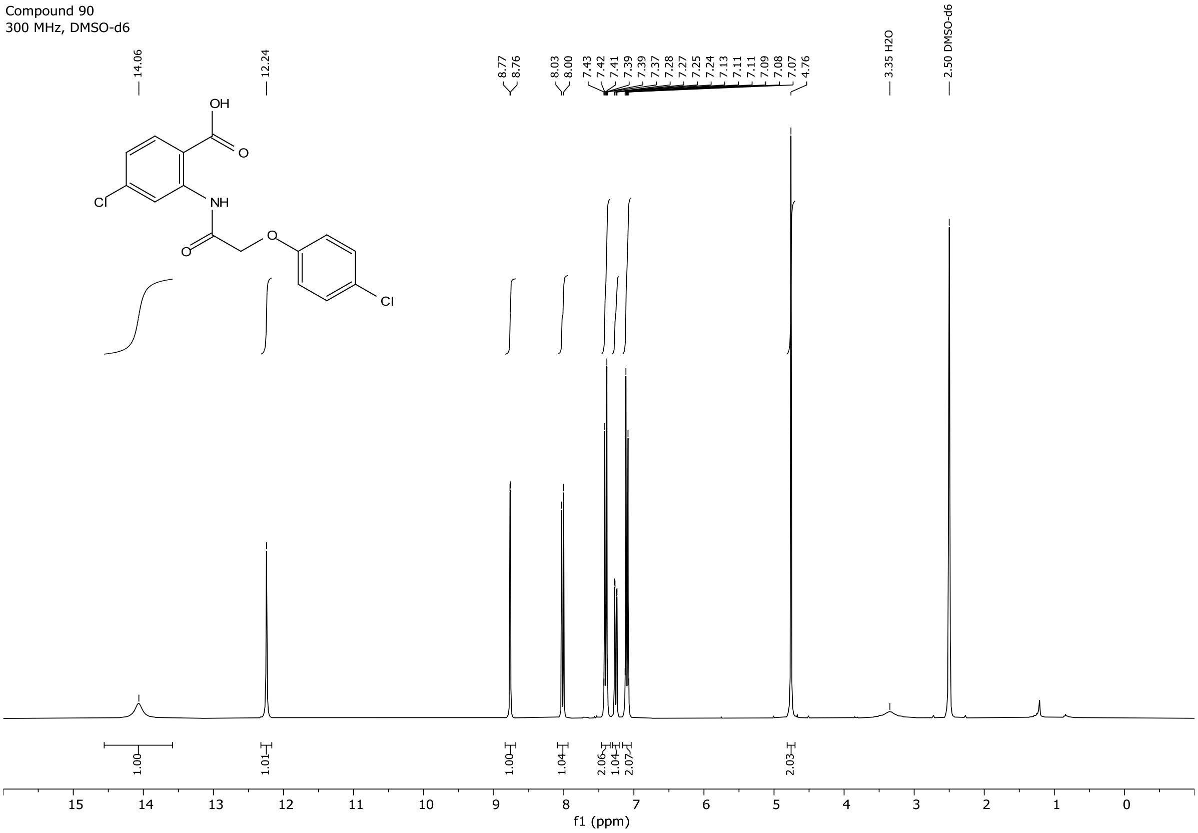

Compound 90
75 MHz, DMSO-d6

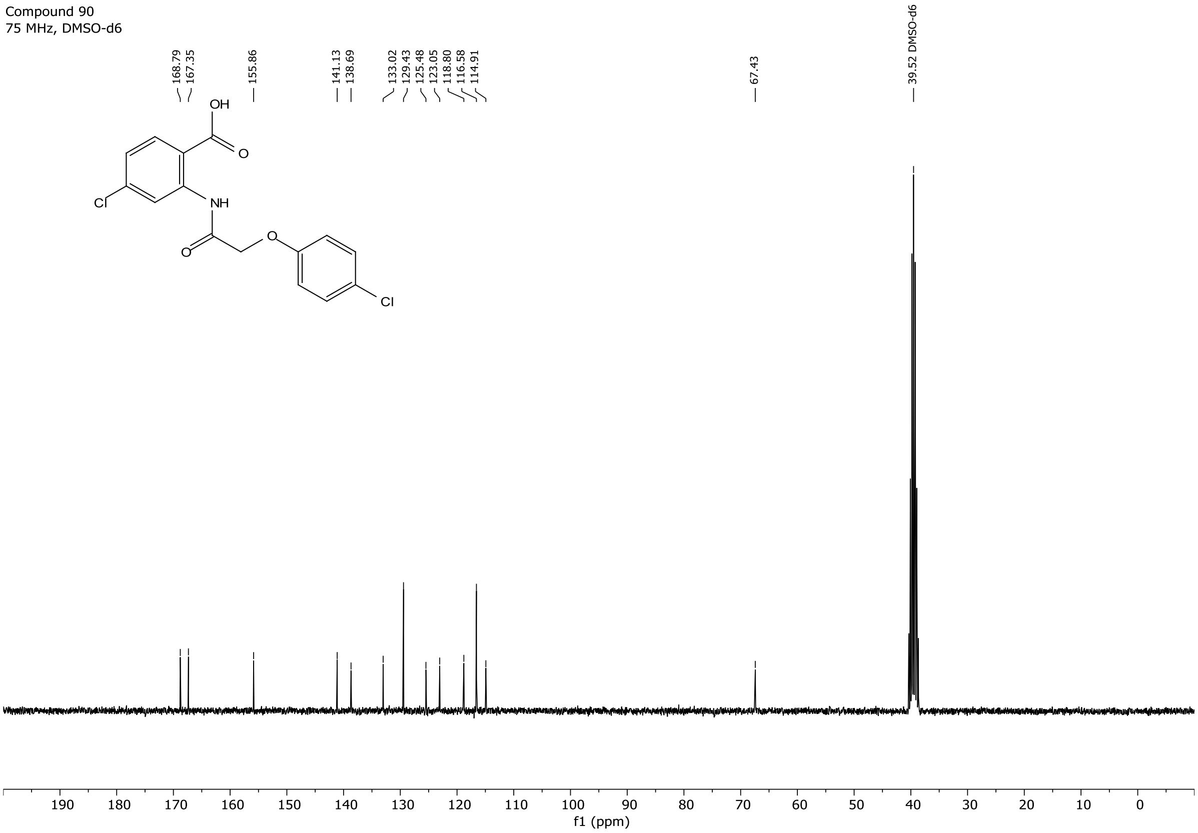

Compound 91
300 MHz, DMSO-d6

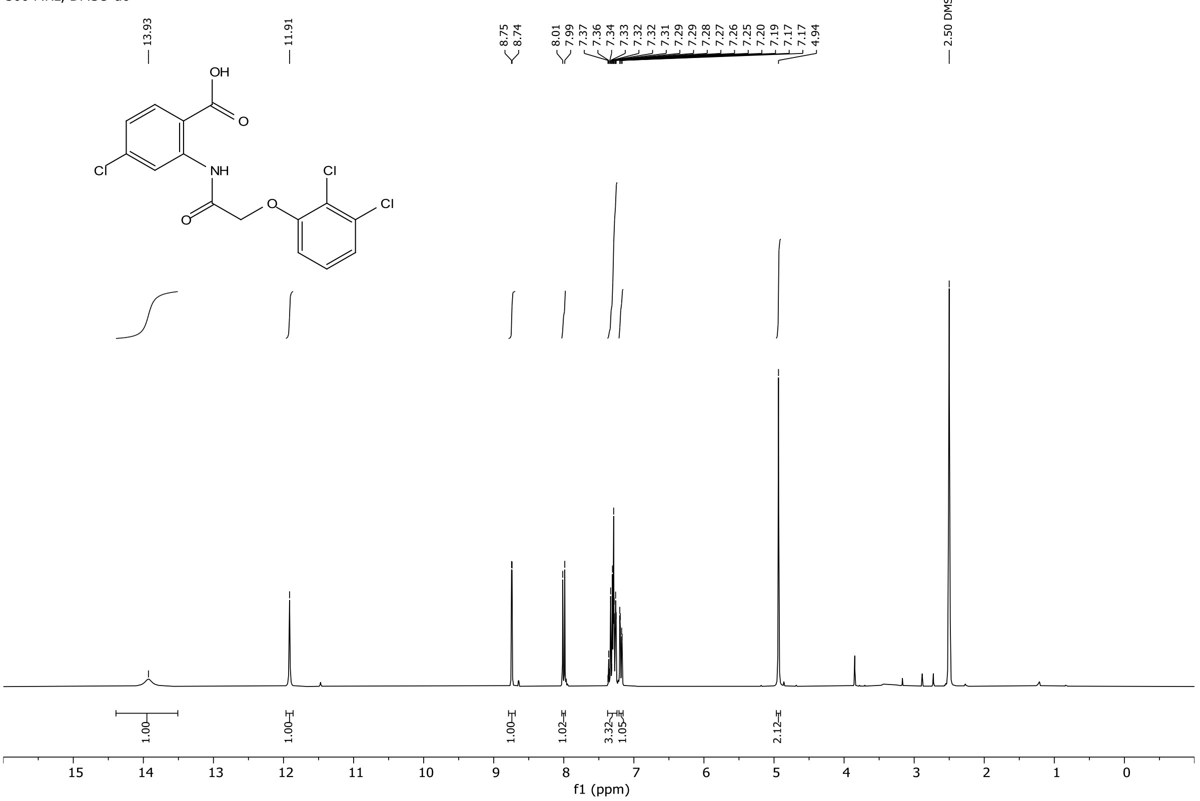

Compound 91
75 MHz, DMSO-d6

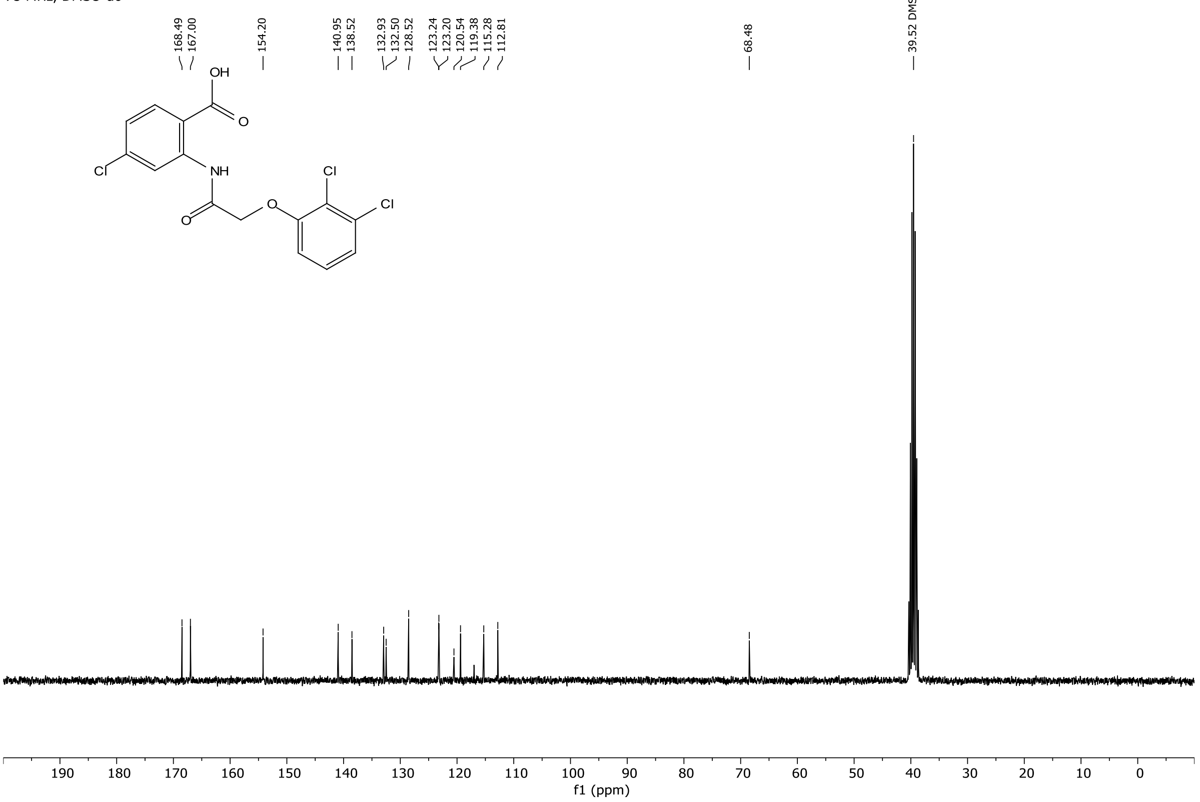

Compound 92
300 MHz, DMSO-d6

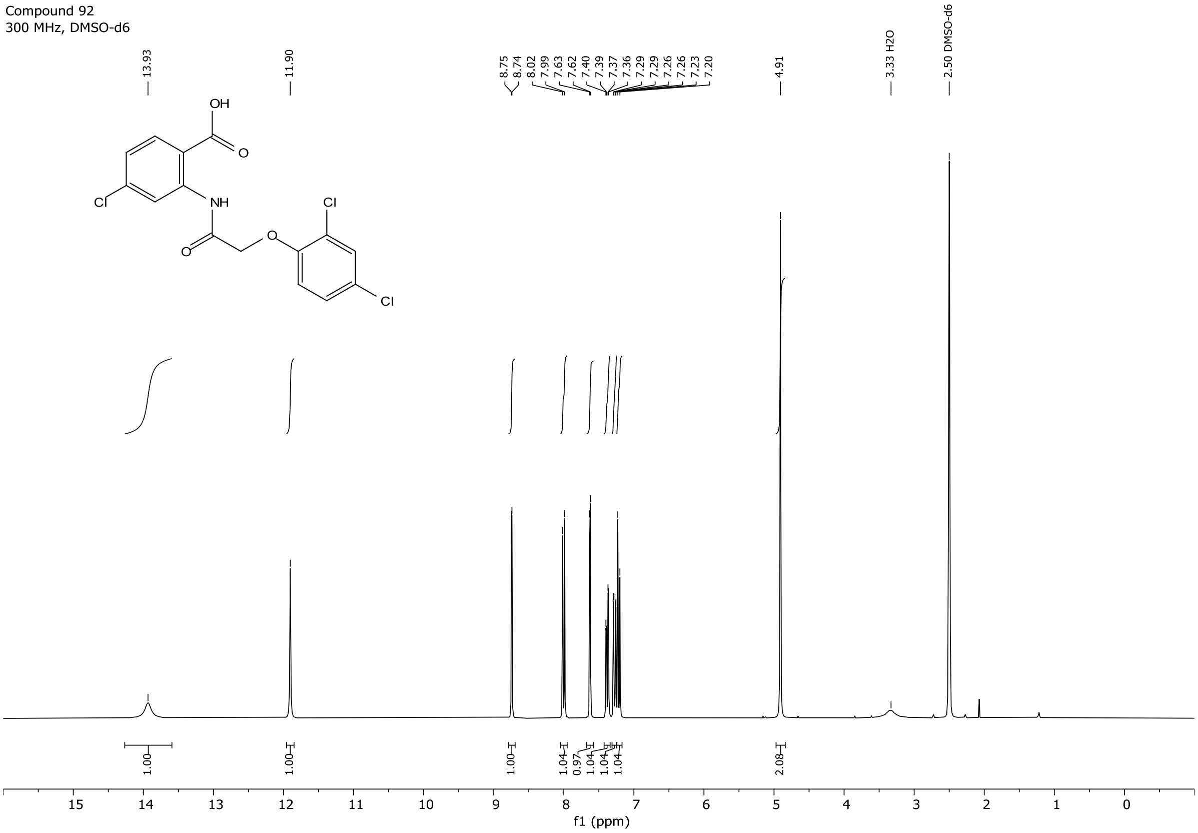

Compound 92
75 MHz, DMSO-d6

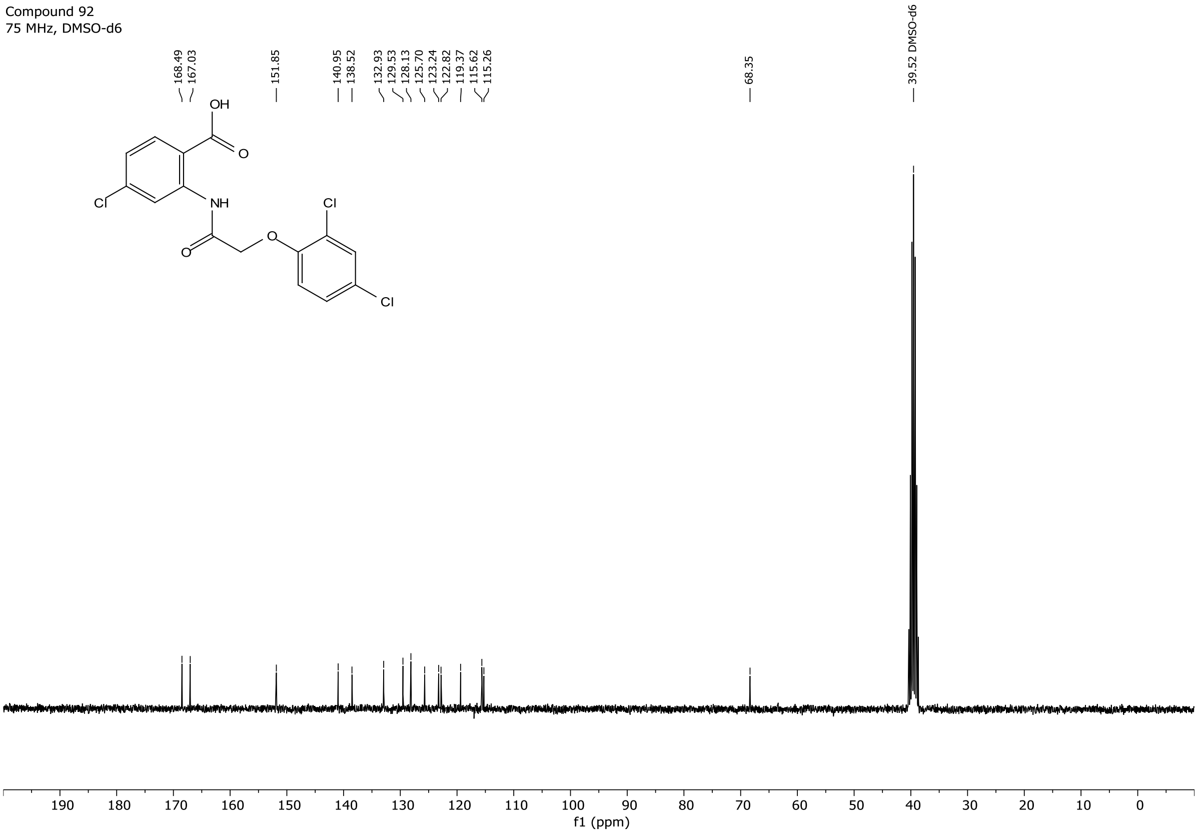

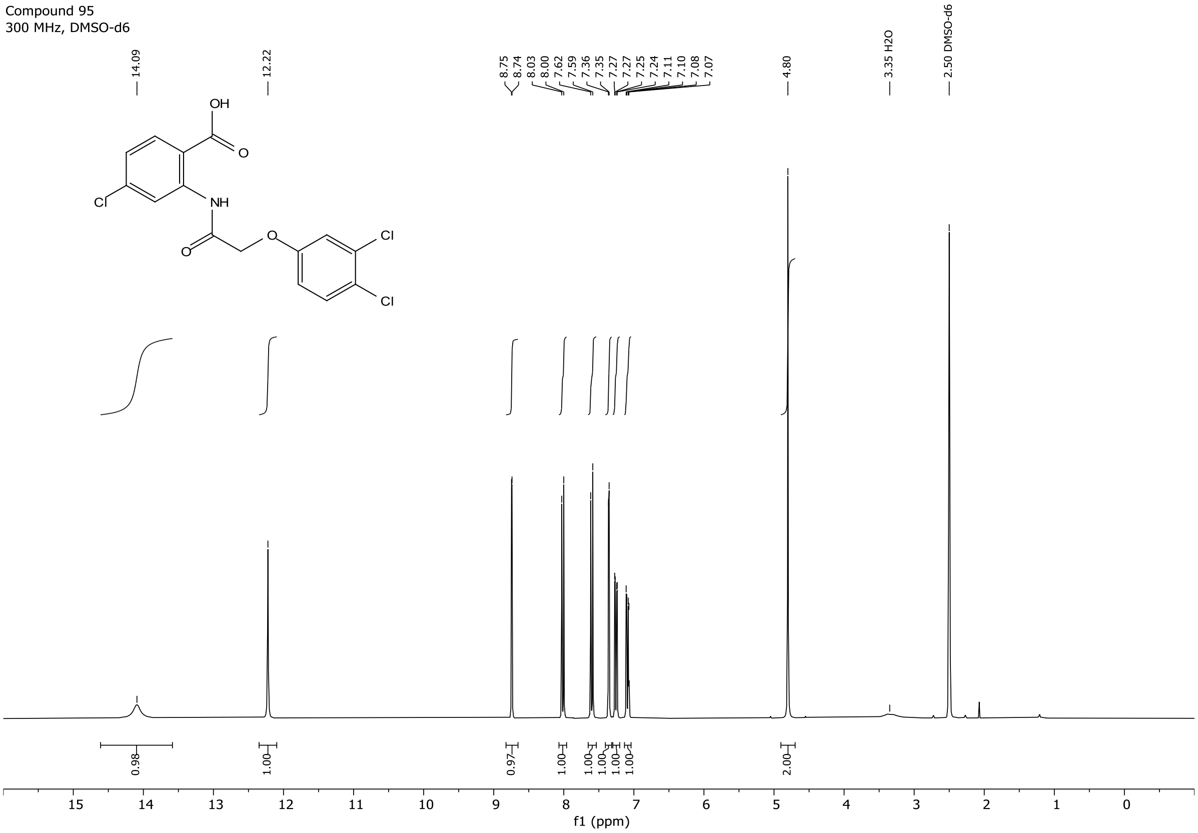

Compound 95
300 MHz, DMSO-d6
14.09
12.22
8.75
8.74
8.03
8.00
7.62
7.59
7.36
7.35
7.27
7.27
7.25
7.24
7.11
7.10
7.08
7.07
4.80
3.35 H2O
2.50 DMSO-d6
OH
O
Cl
NH
O
O
Cl
Cl
0.98
1.00
0.97
1.00
1.00
1.00
1.00
1.00
2.00
15
14
13
12
11
10
9
8
7
6
5
4
3
2
1
0
f1 (ppm)

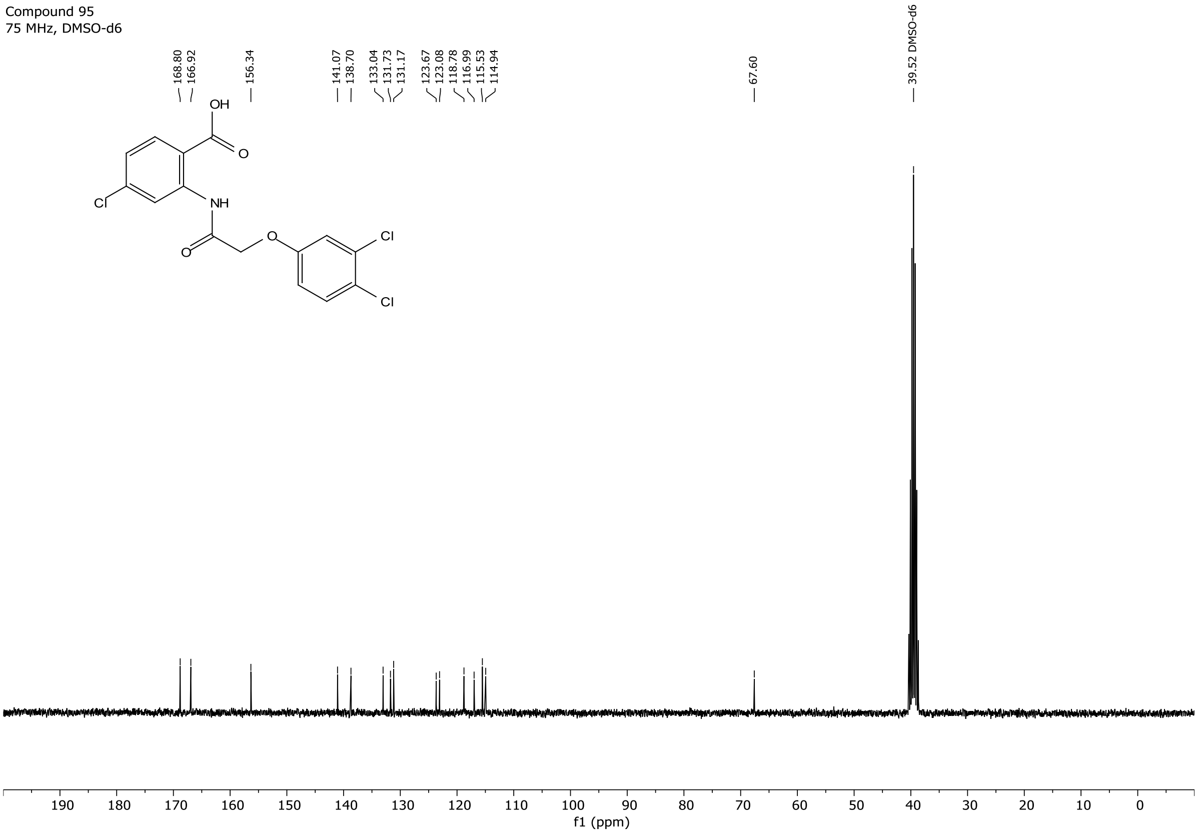

Compound 95
75 MHz, DMSO-d6
168.80
166.92
156.34
141.07
138.70
133.04
131.73
131.17
123.67
123.08
118.78
116.99
115.53
114.94
67.60
39.52 DMSO-d6
OH
O
Cl
NH
O
O
Cl
Cl
190
180
170
160
150
140
130
120
110
100
90
80
70
60
50
40
30
20
10
0
f1 (ppm)

Compound 96
300 MHz, DMSO-d6

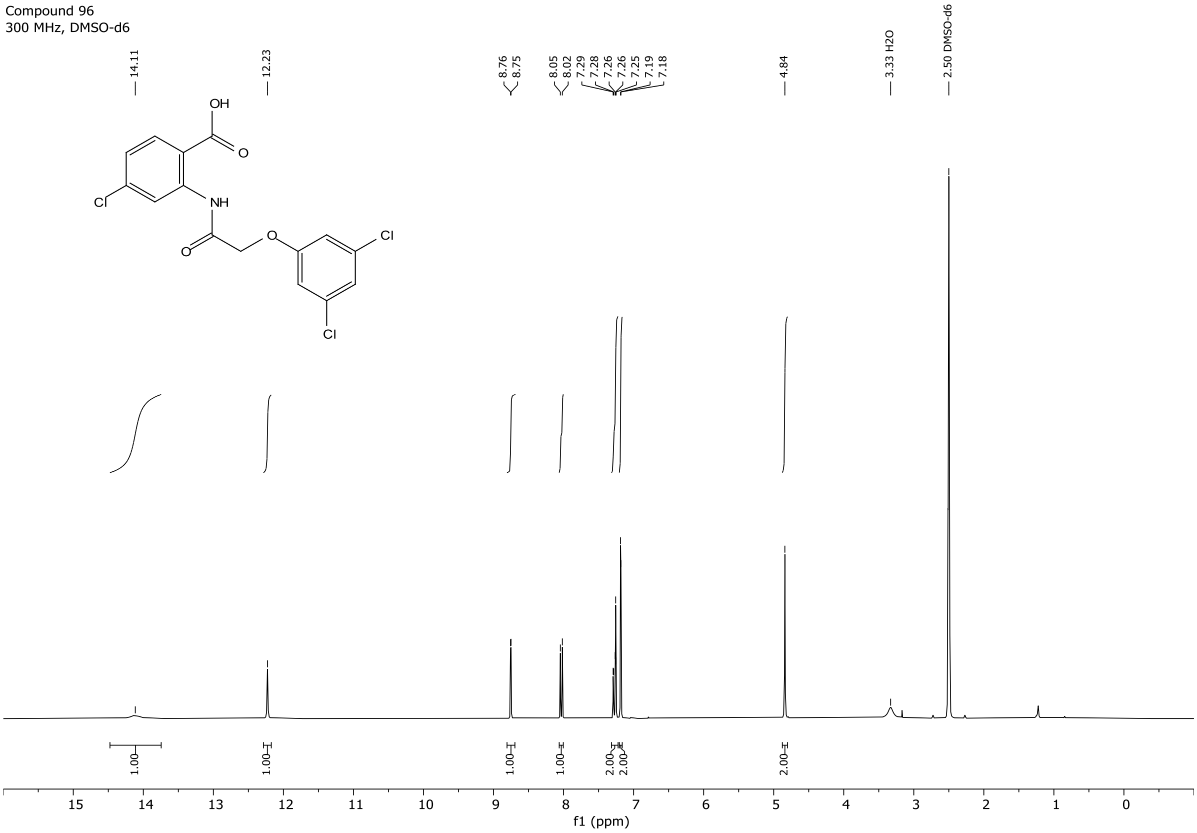

Compound 96
75 MHz, DMSO-d6

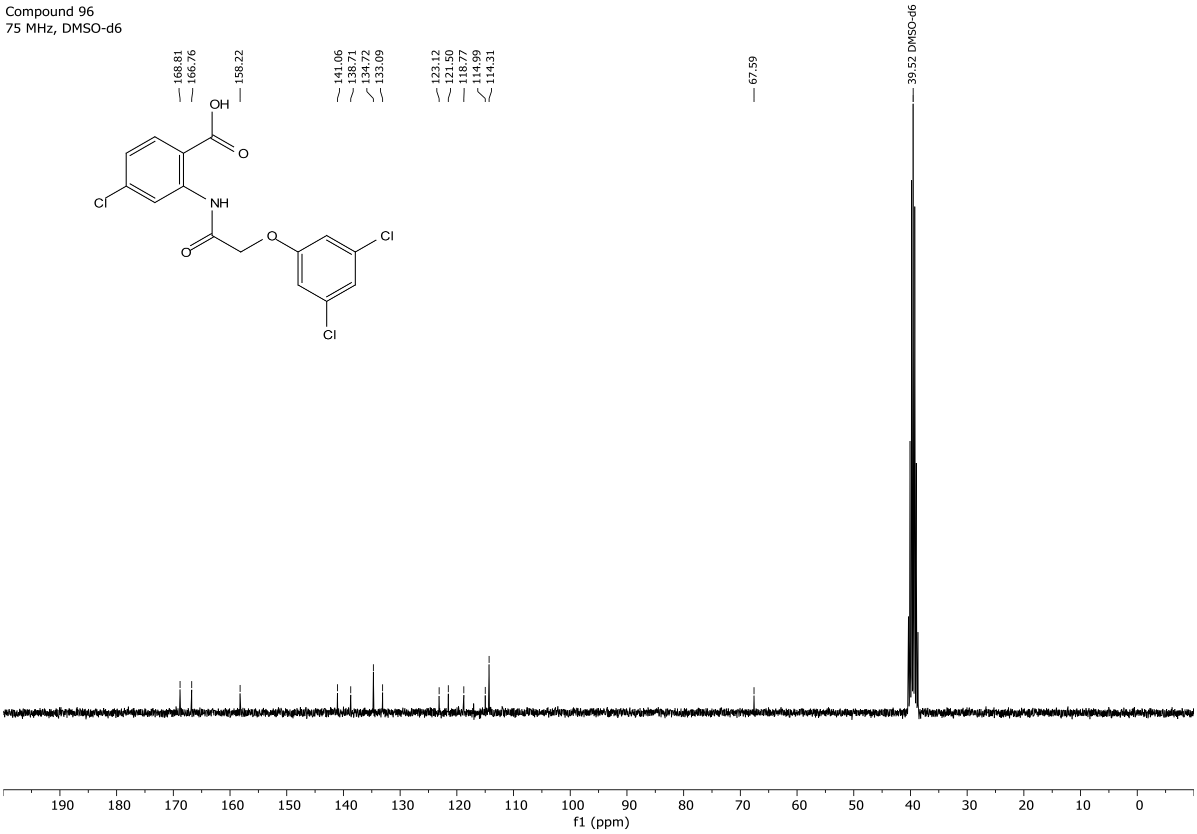

Compound 97
300 MHz, DMSO-d6

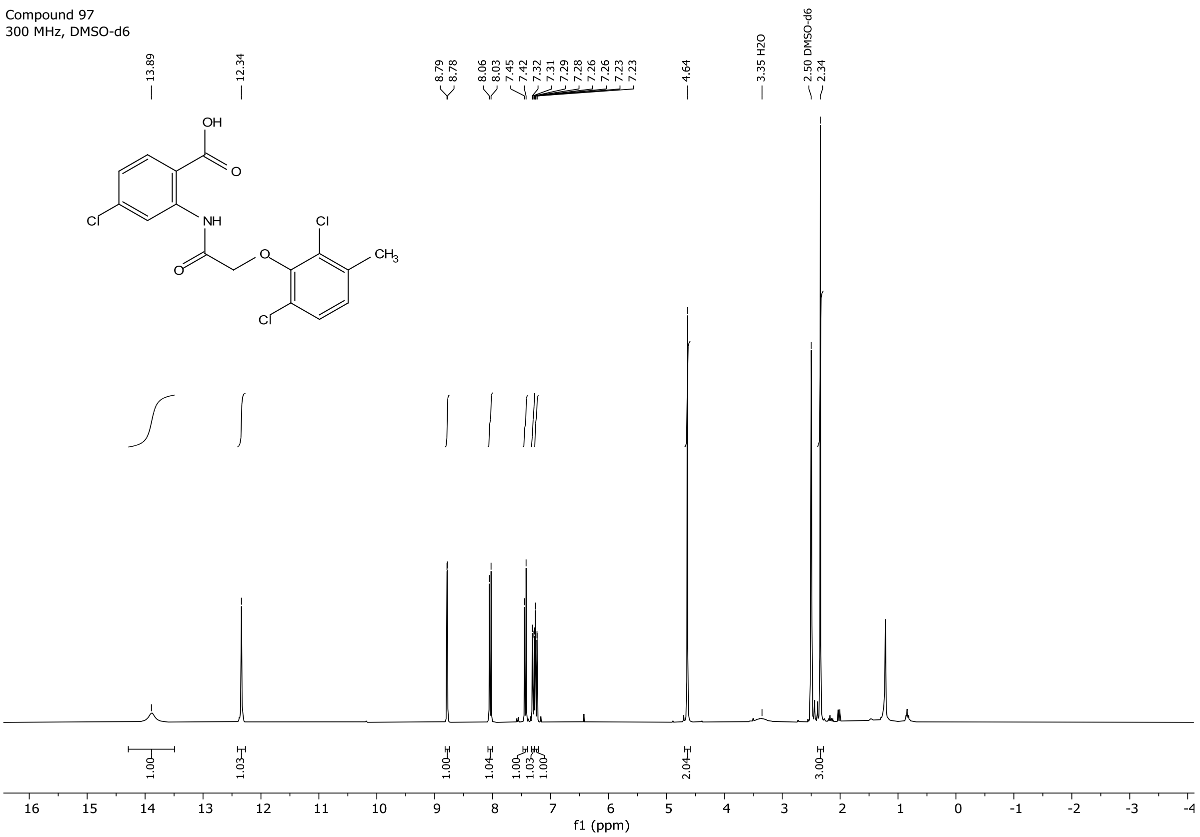

Compound 97
75 MHz, DMSO-d6

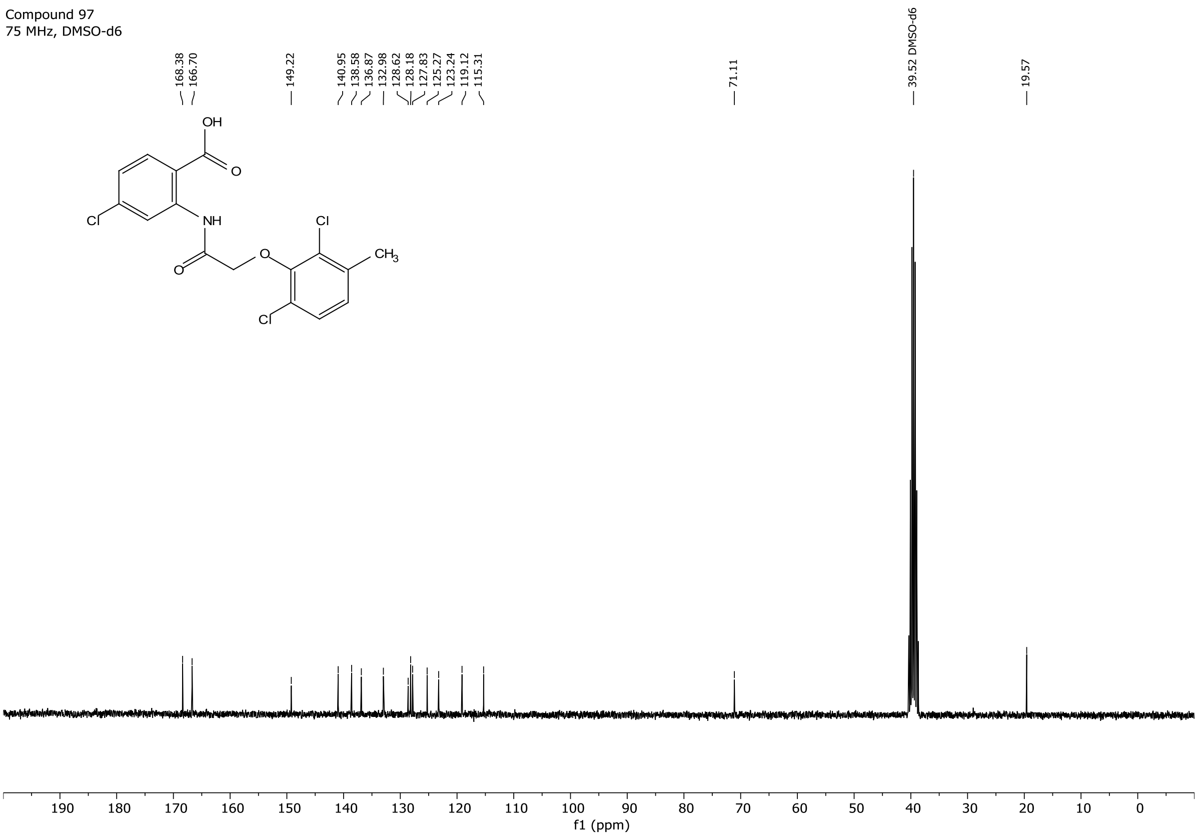

Compound 98
300 MHz, DMSO-d6

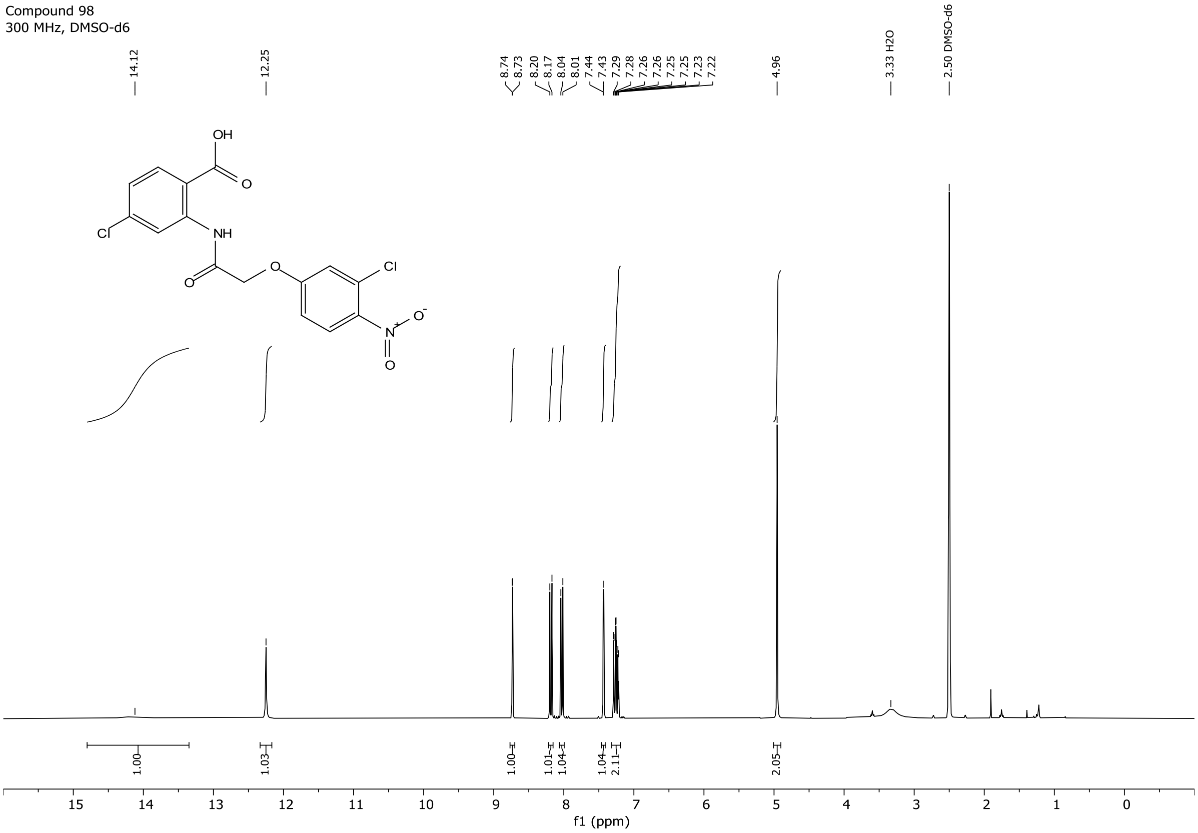

Compound 98
75 MHz, DMSO-d6

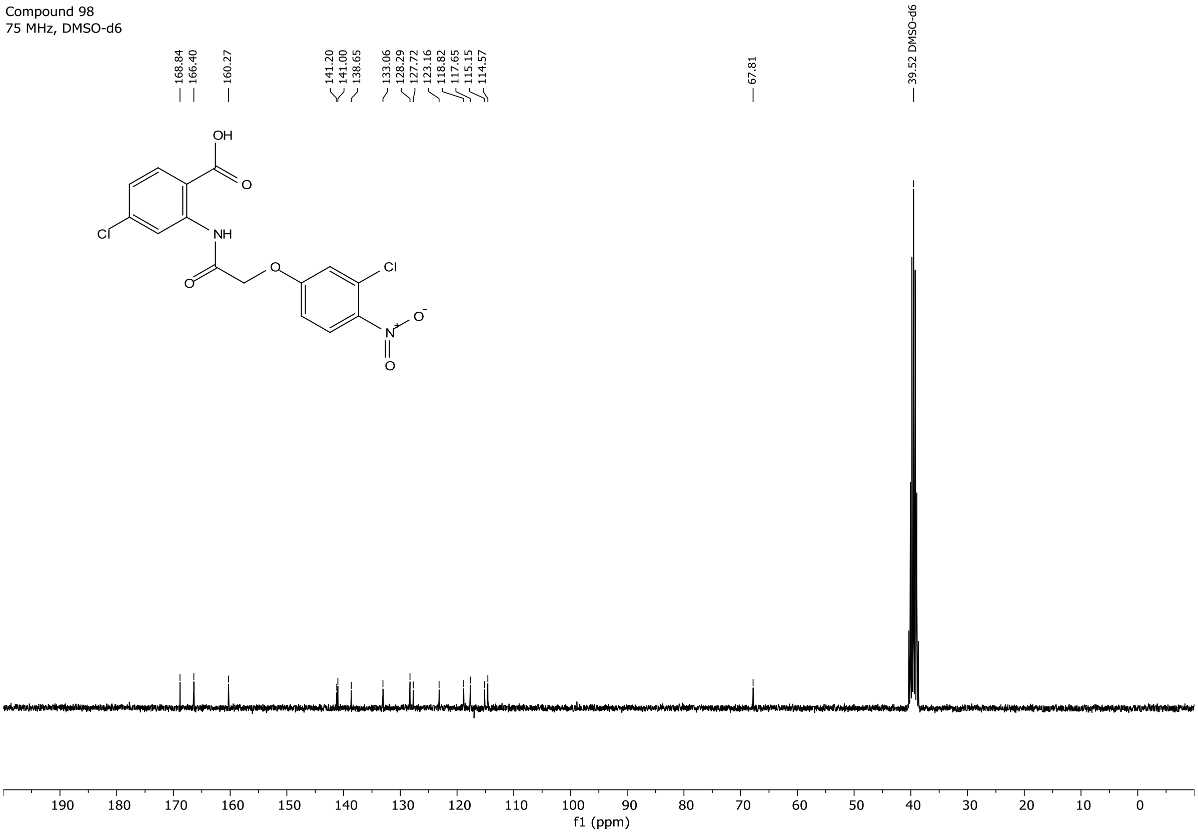

Compound 99
300 MHz, DMSO-d6

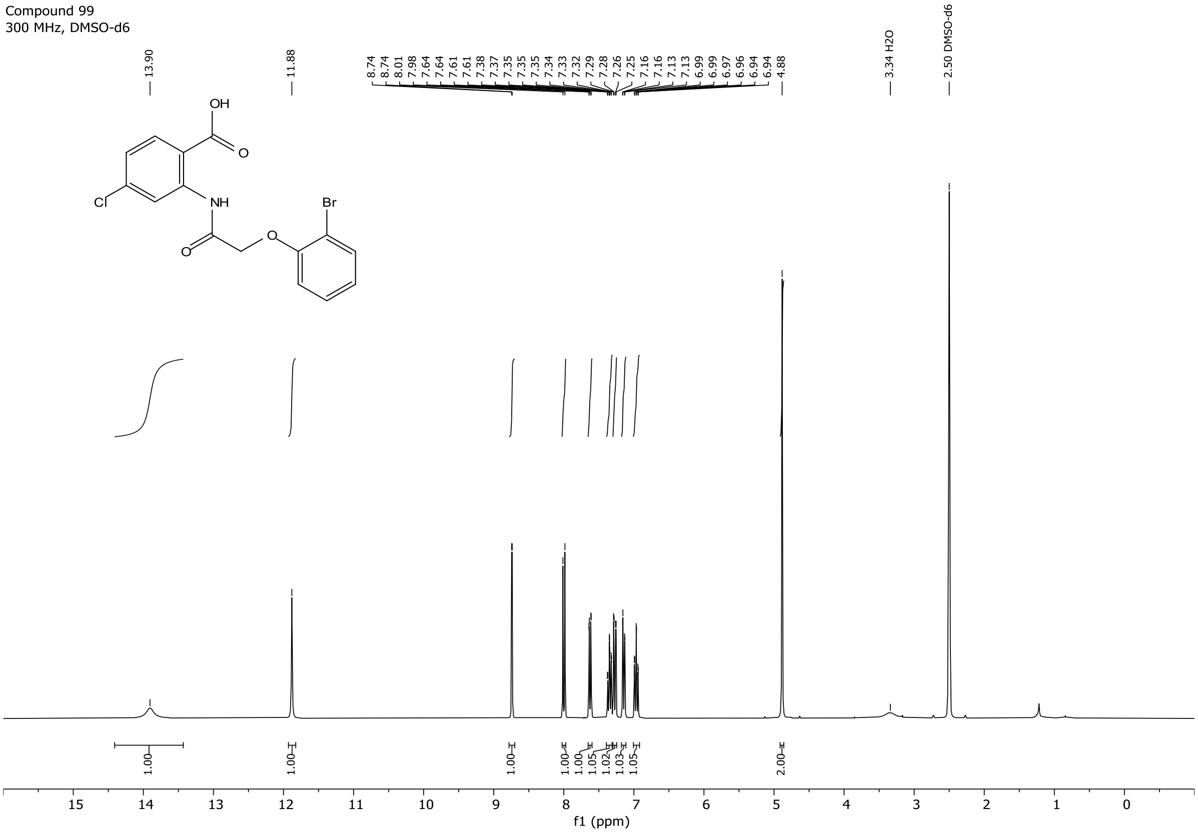

Compound 99
75 MHz, DMSO-d6

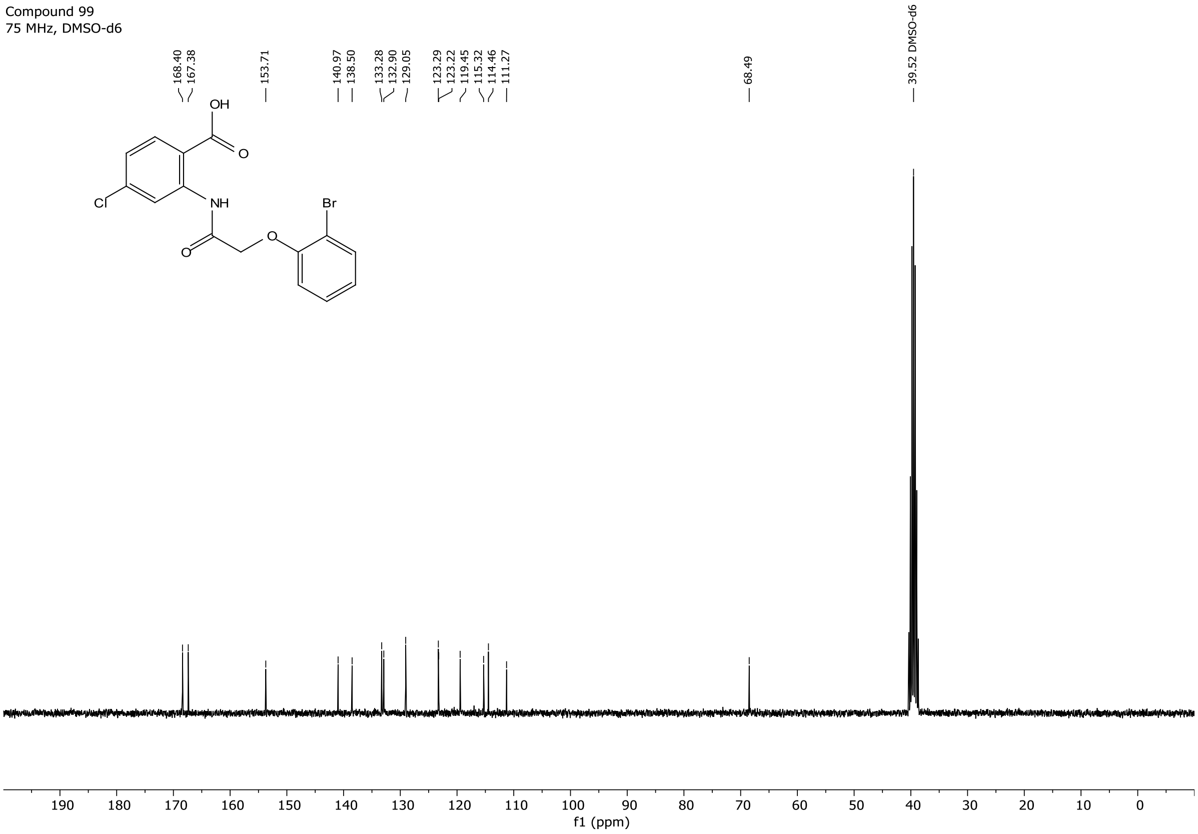

Compound 100
300 MHz, DMSO-d6

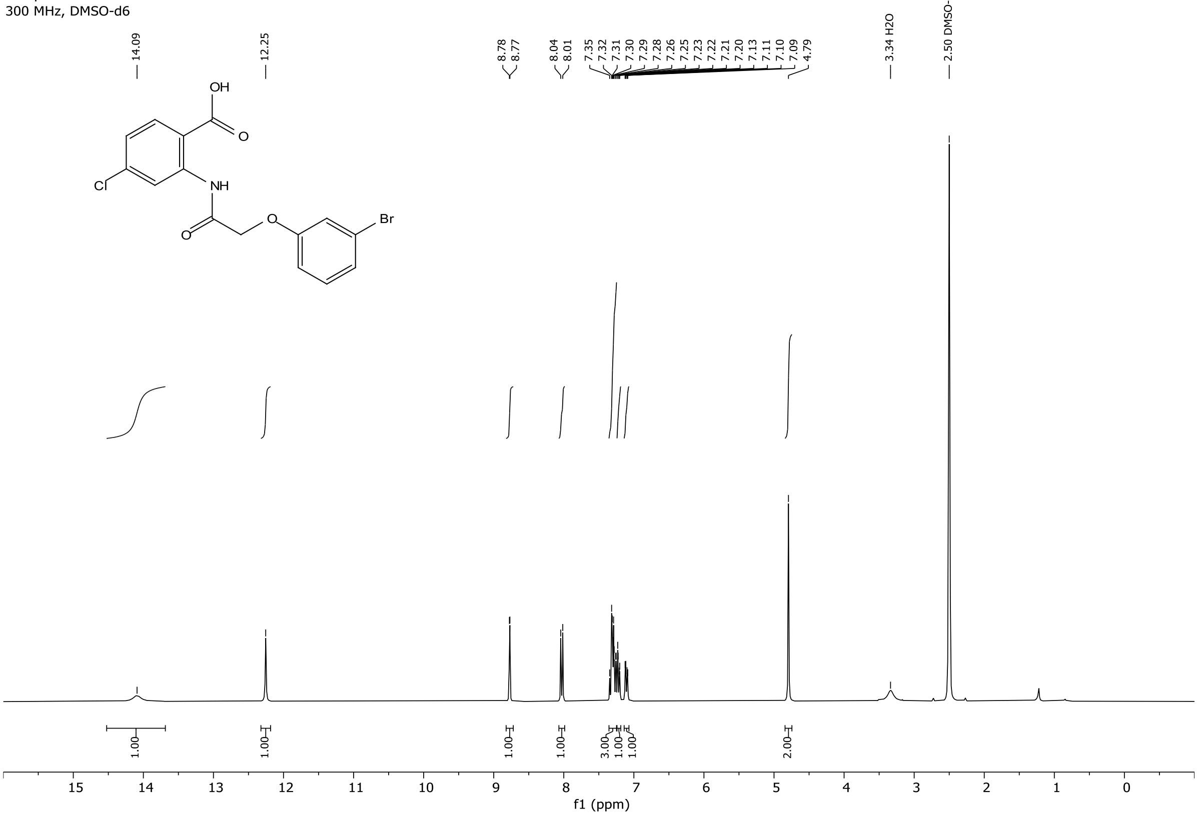

Compound 100
75 MHz, DMSO-d6

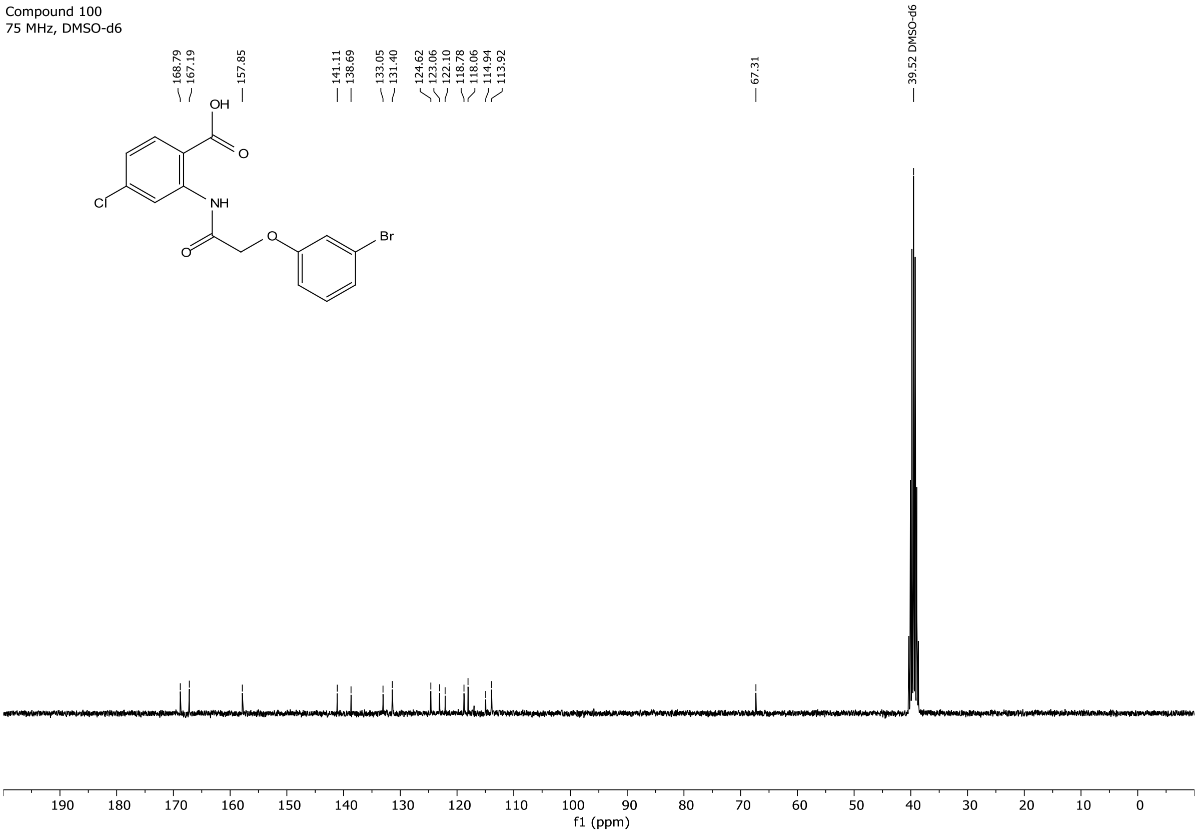

Compound 101
300 MHz, DMSO-d6

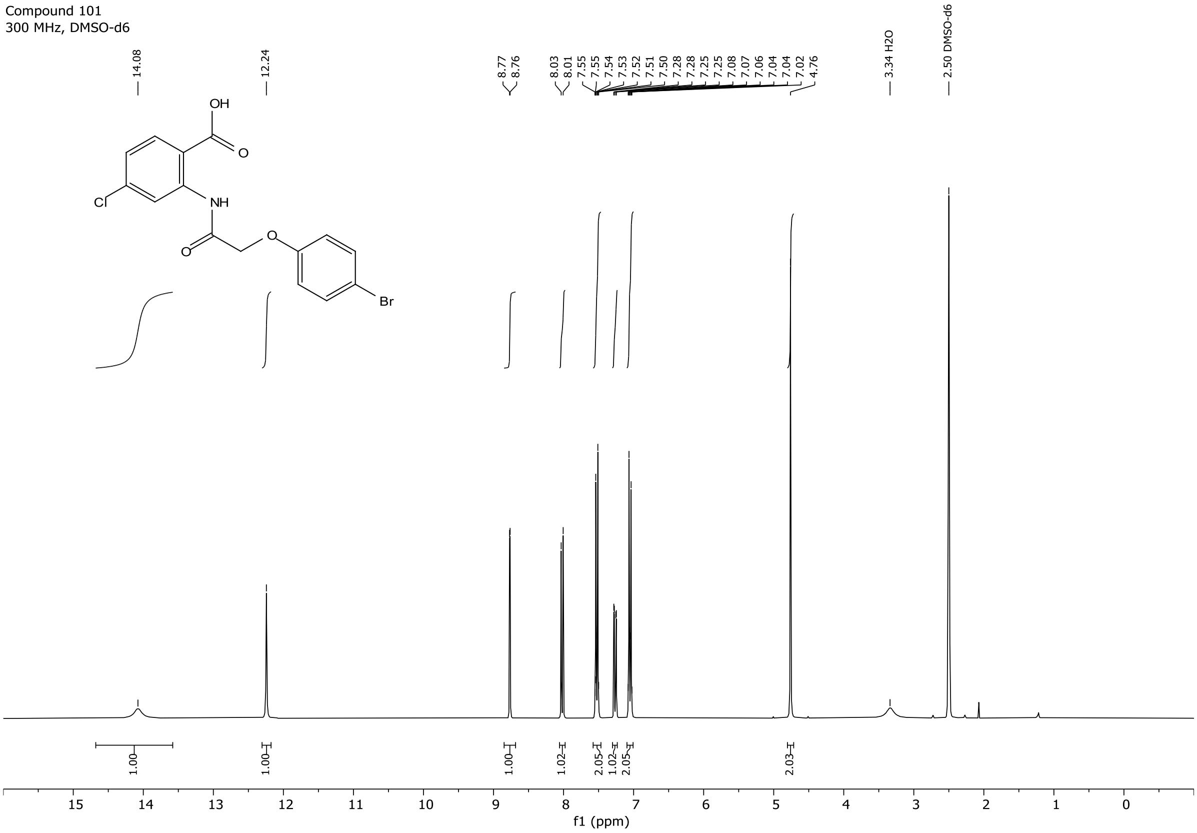

Compound 101
75 MHz, DMSO-d6

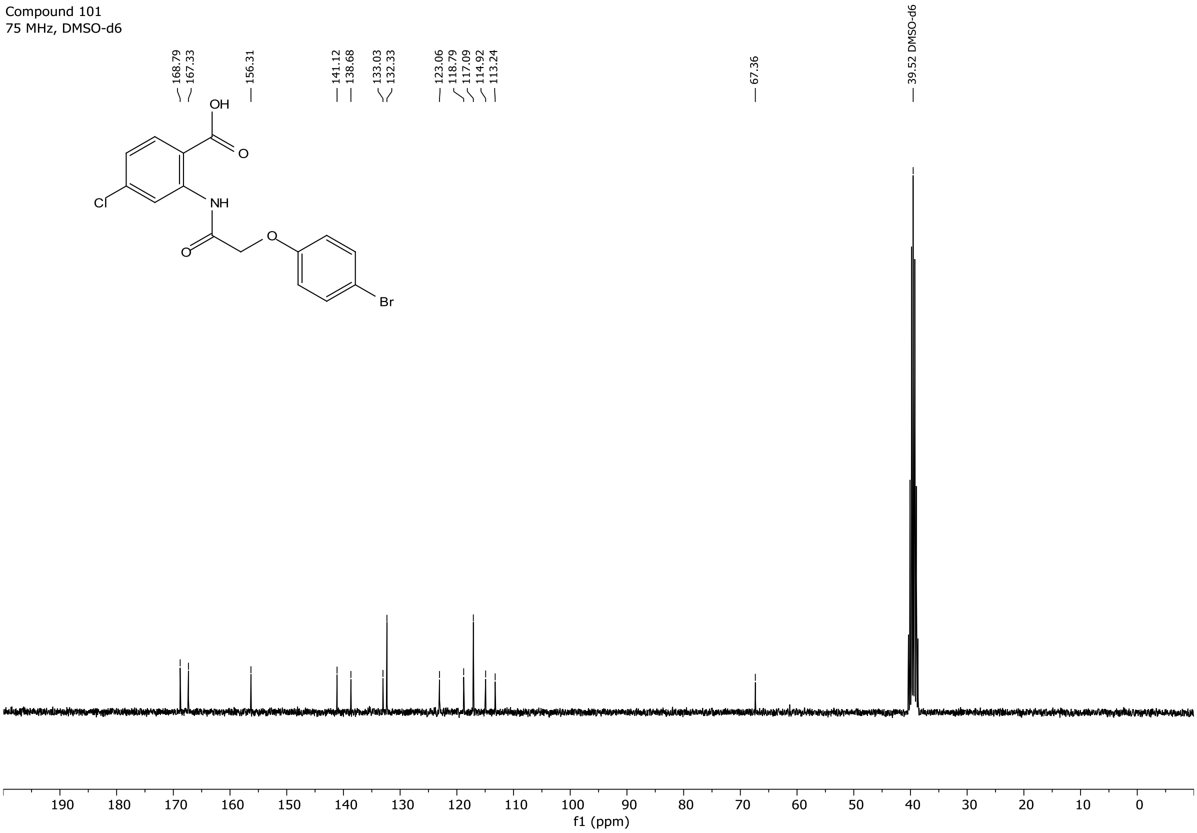

Compound 102
300 MHz, DMSO-d6

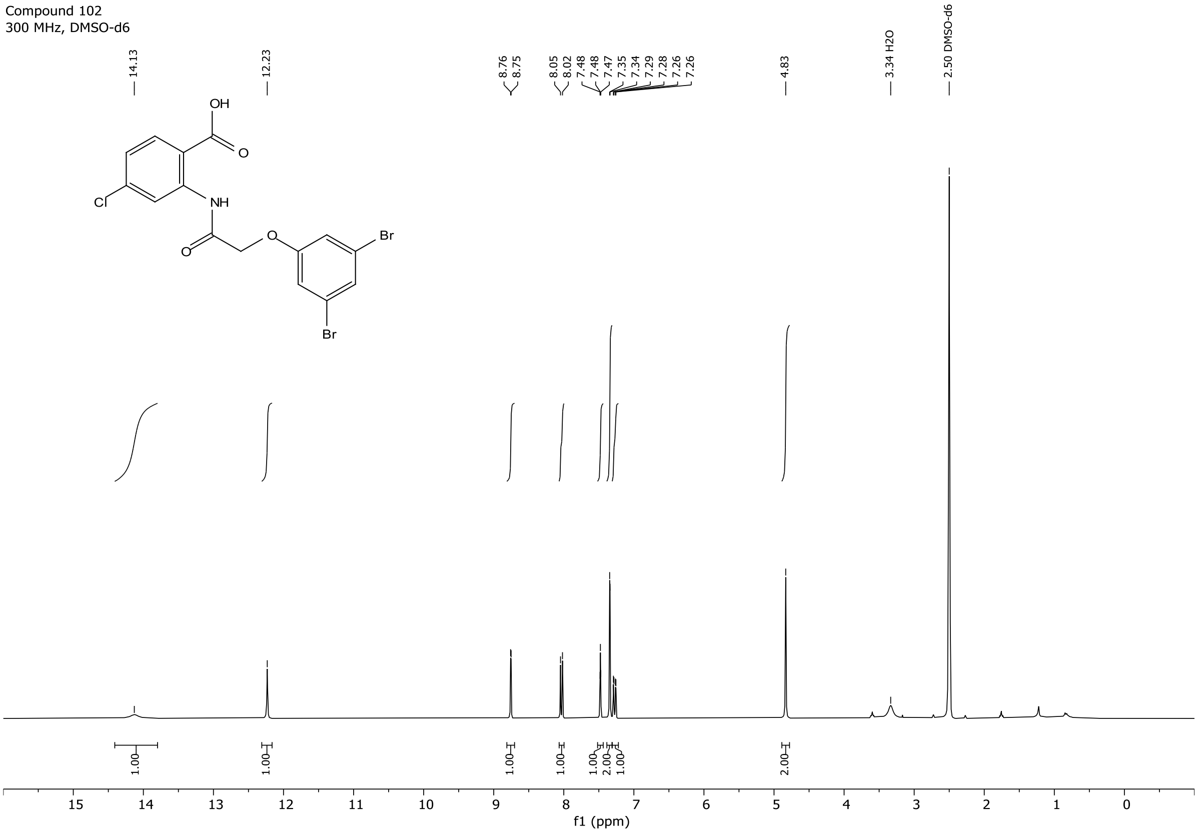

Compound 102
75 MHz, DMSO-d6

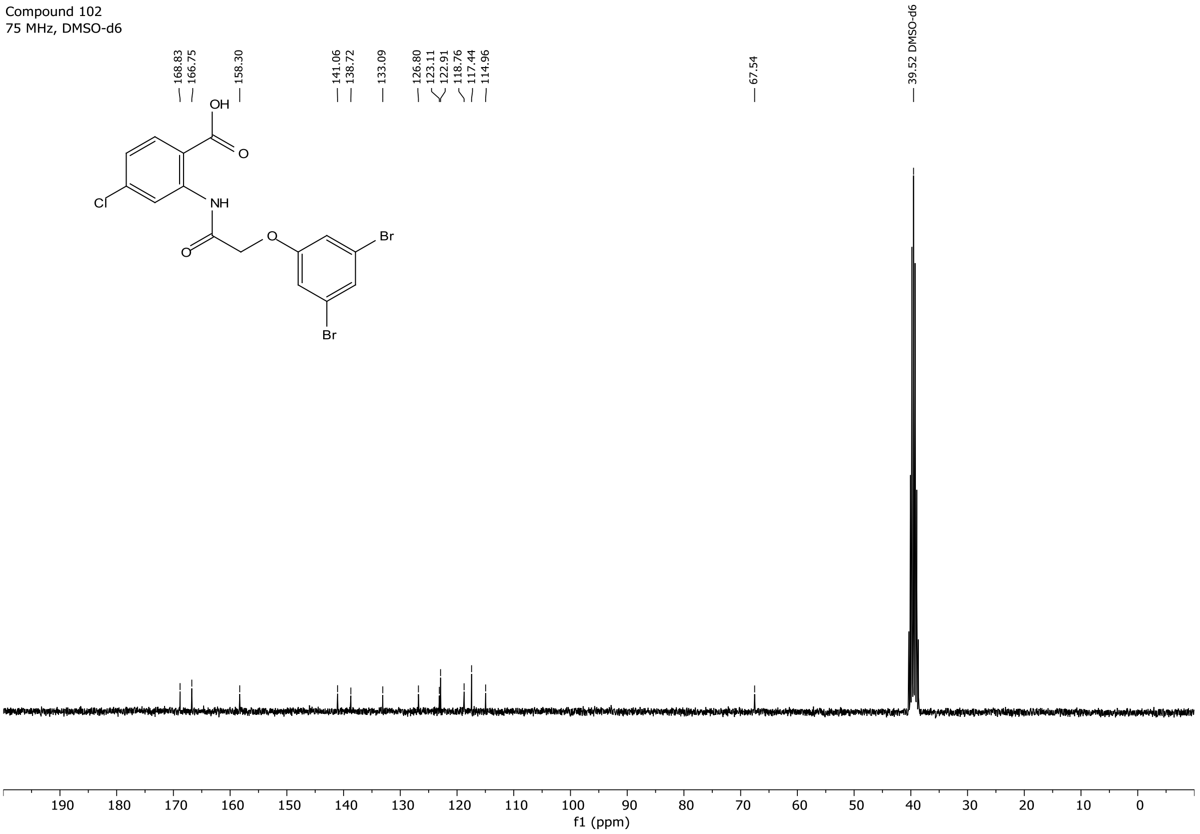

Compound 103 (IBA)
300 MHz, DMSO-d6

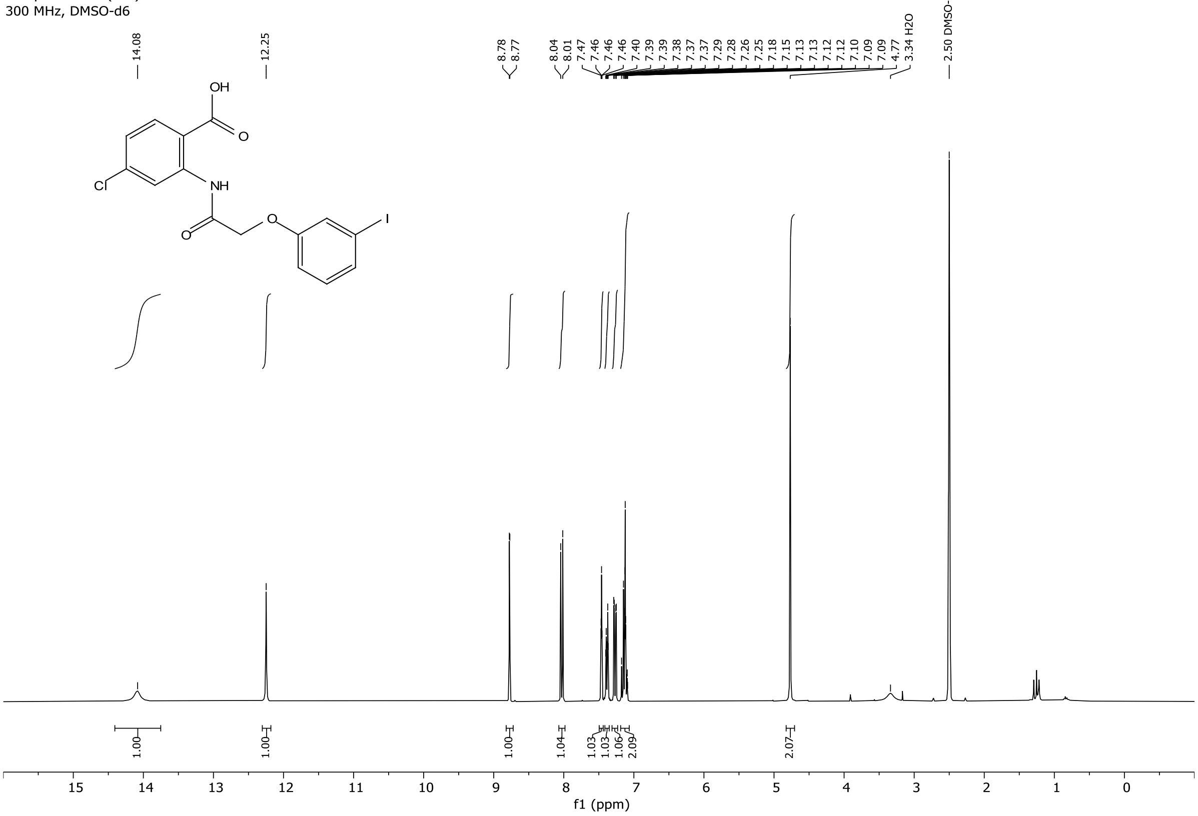

Compound 103 (IBA)
75 MHz, DMSO-d6

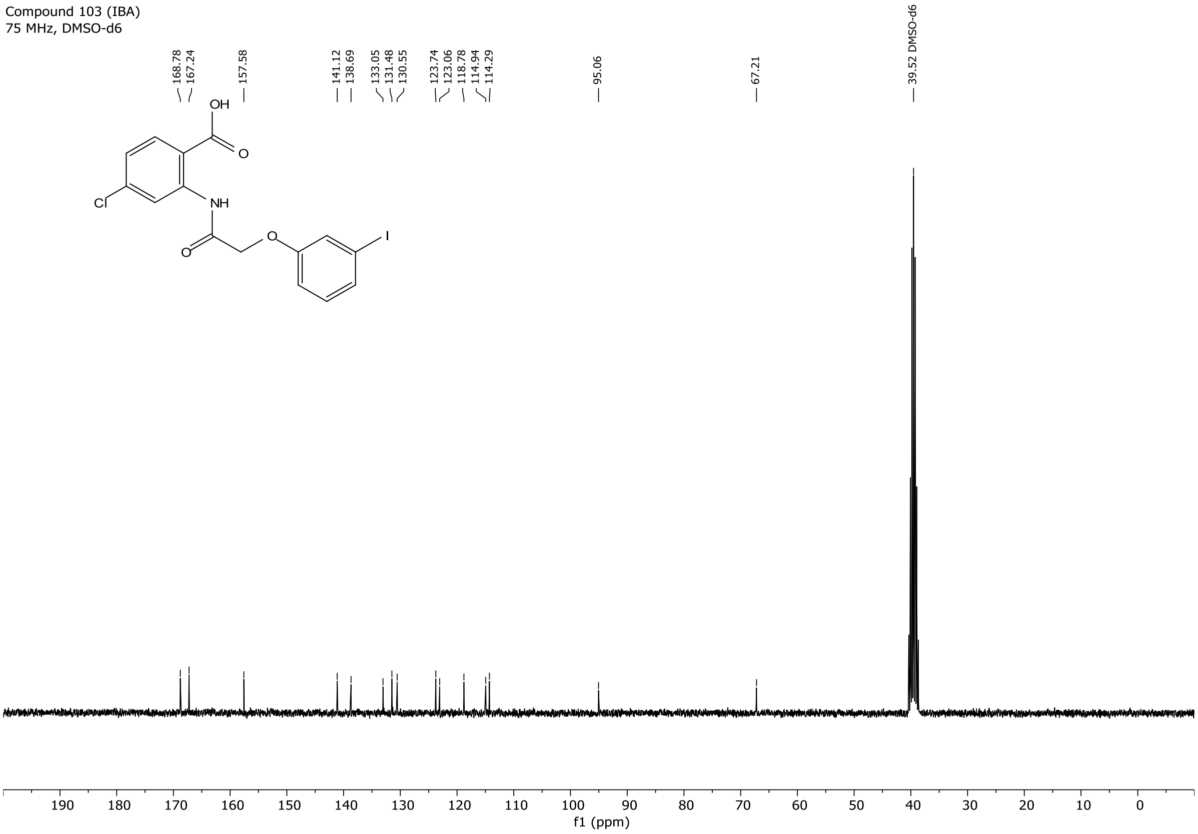

Compound 104
300 MHz, DMSO-d6

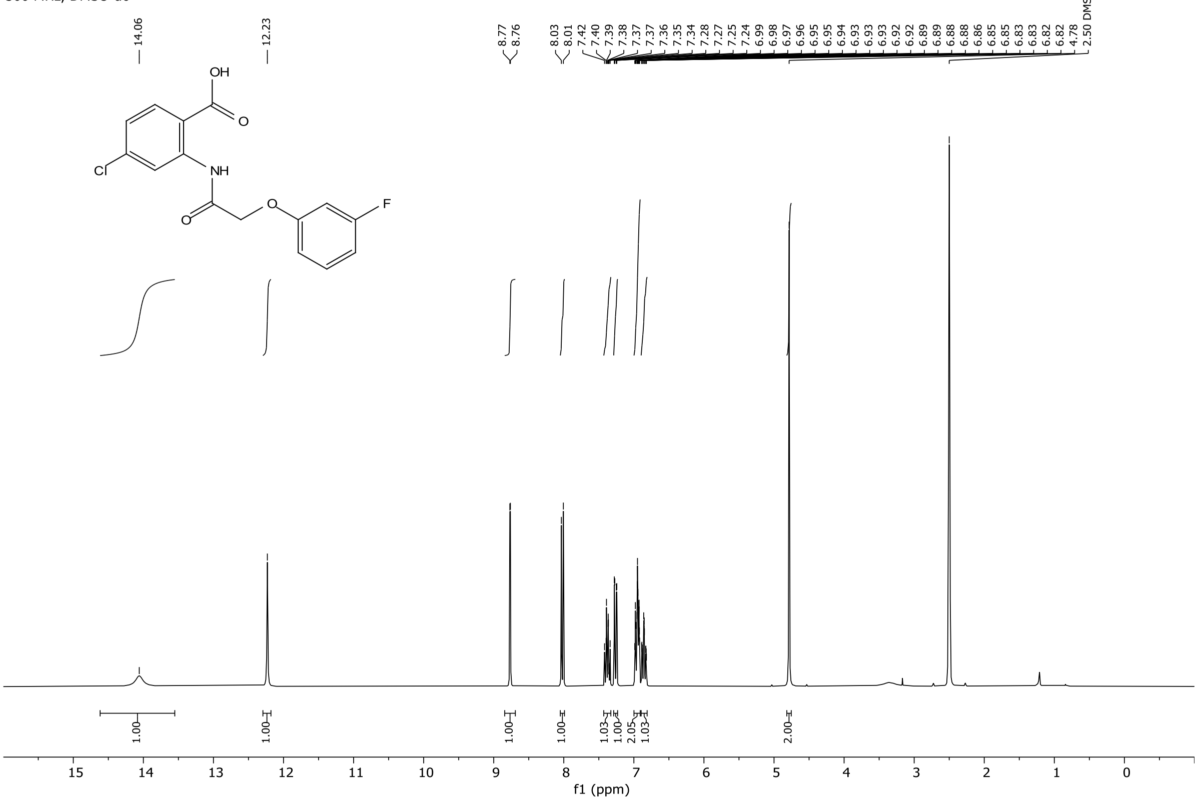

Compound 104
75 MHz, DMSO-d6

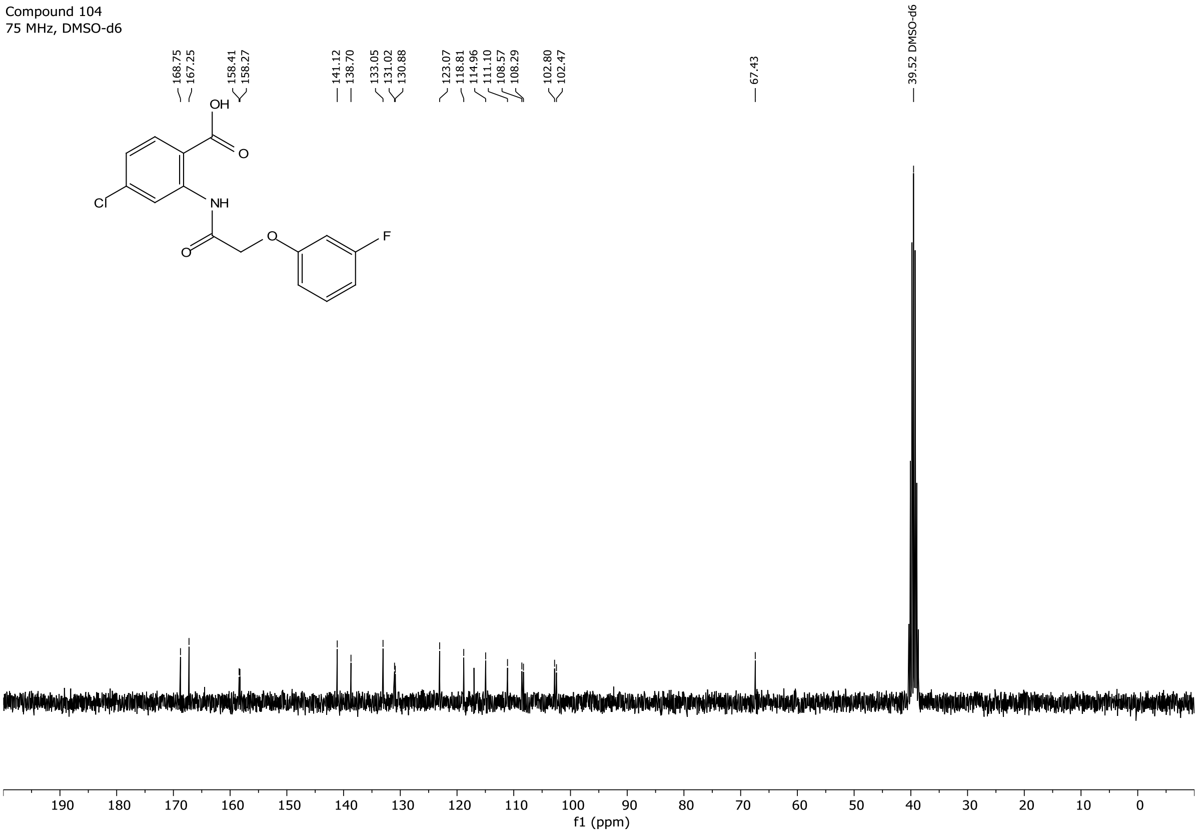

Compound 105
300 MHz, DMSO-d6

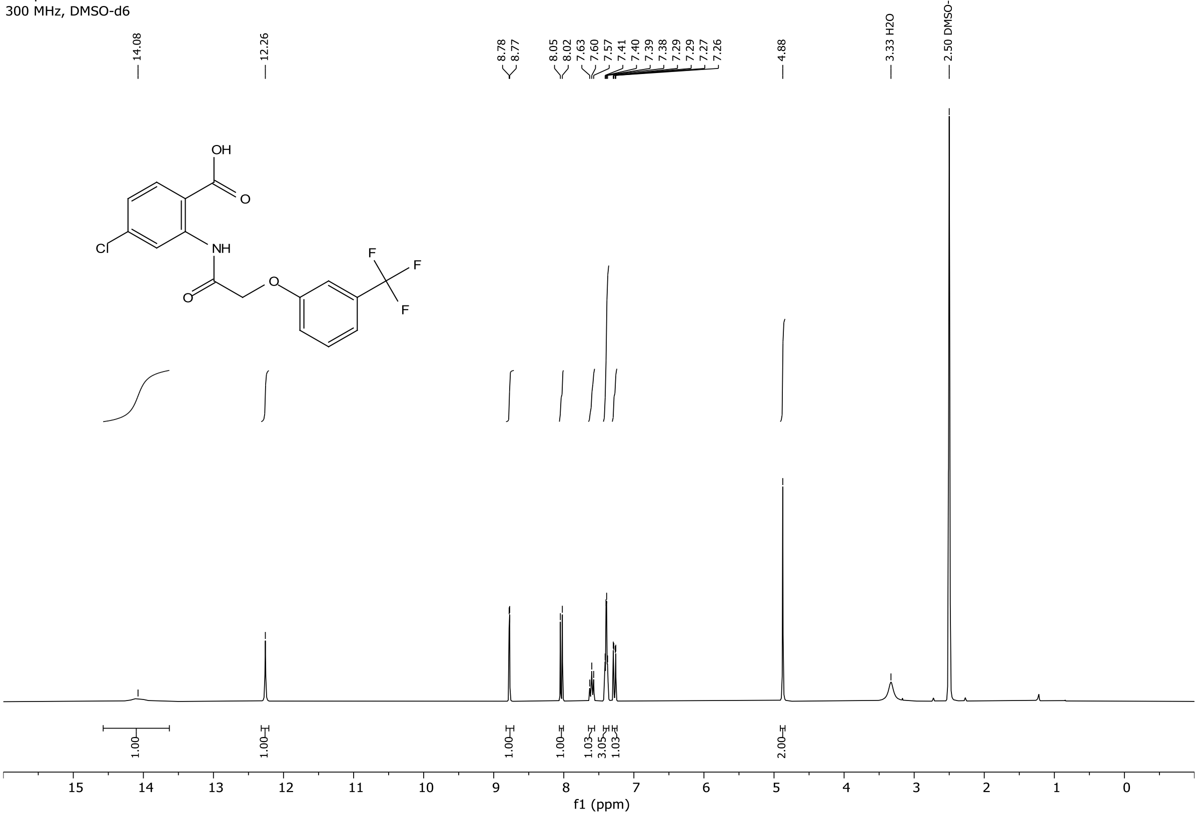

Compound 105
75 MHz, DMSO-d6

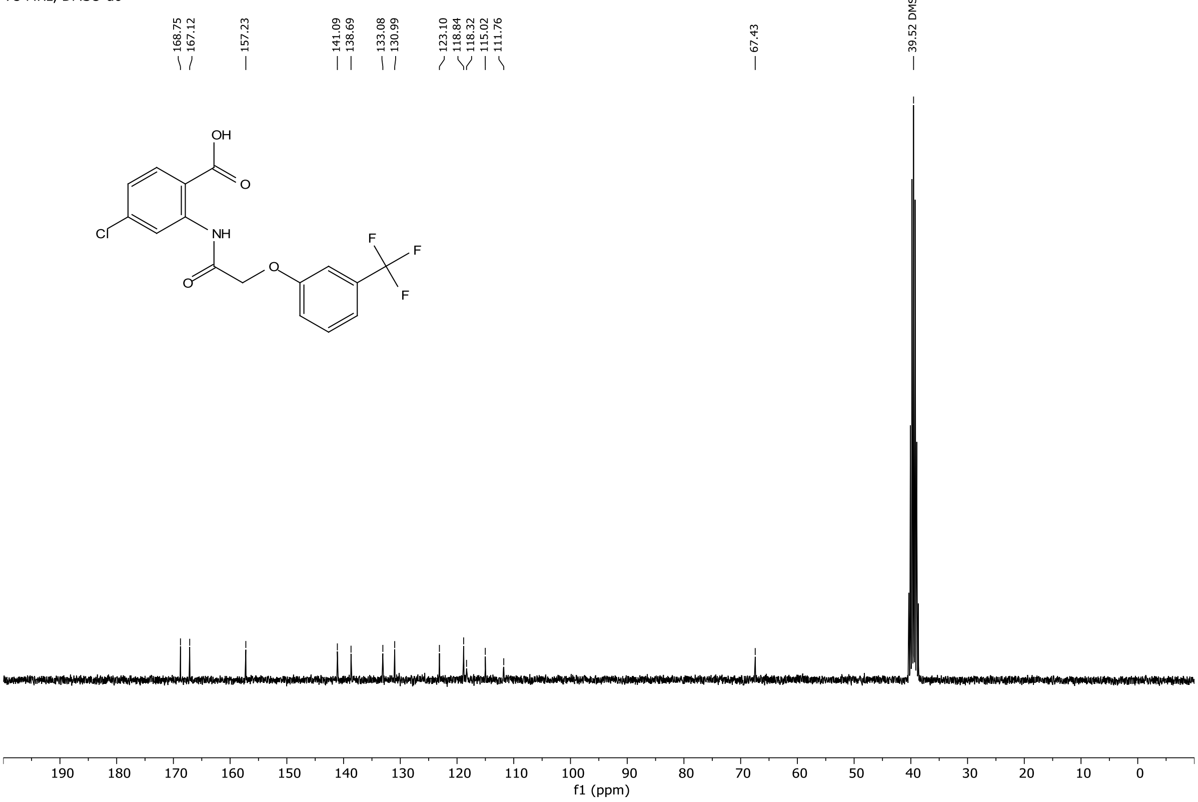

Compound 106
300 MHz, DMSO-d6

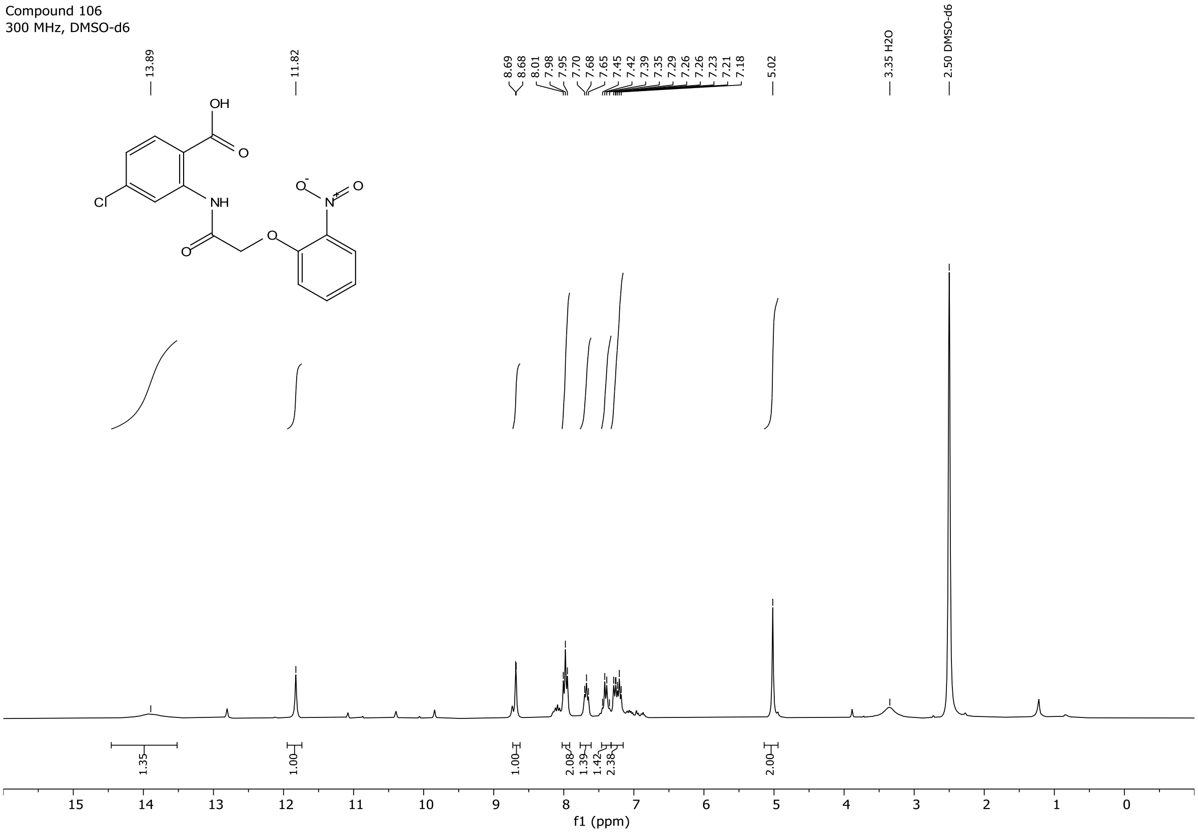

Compound 106
75 MHz, DMSO-d6

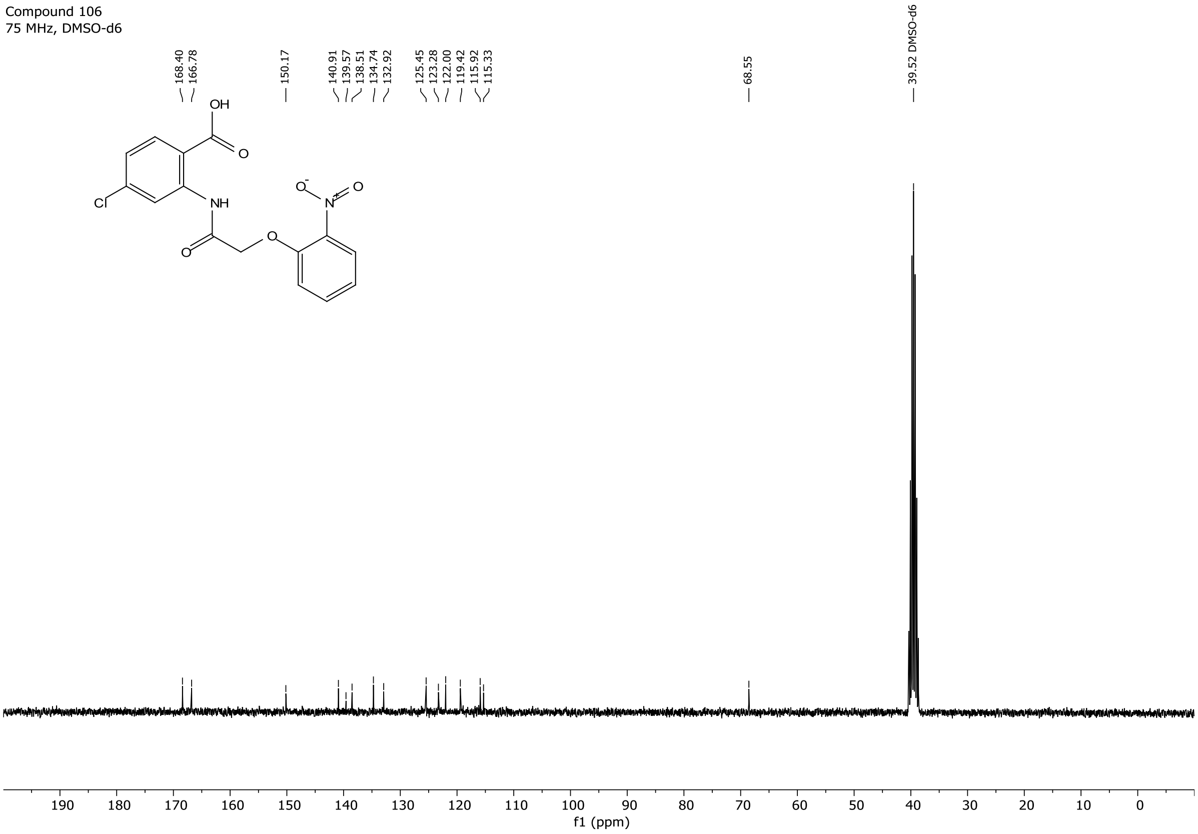

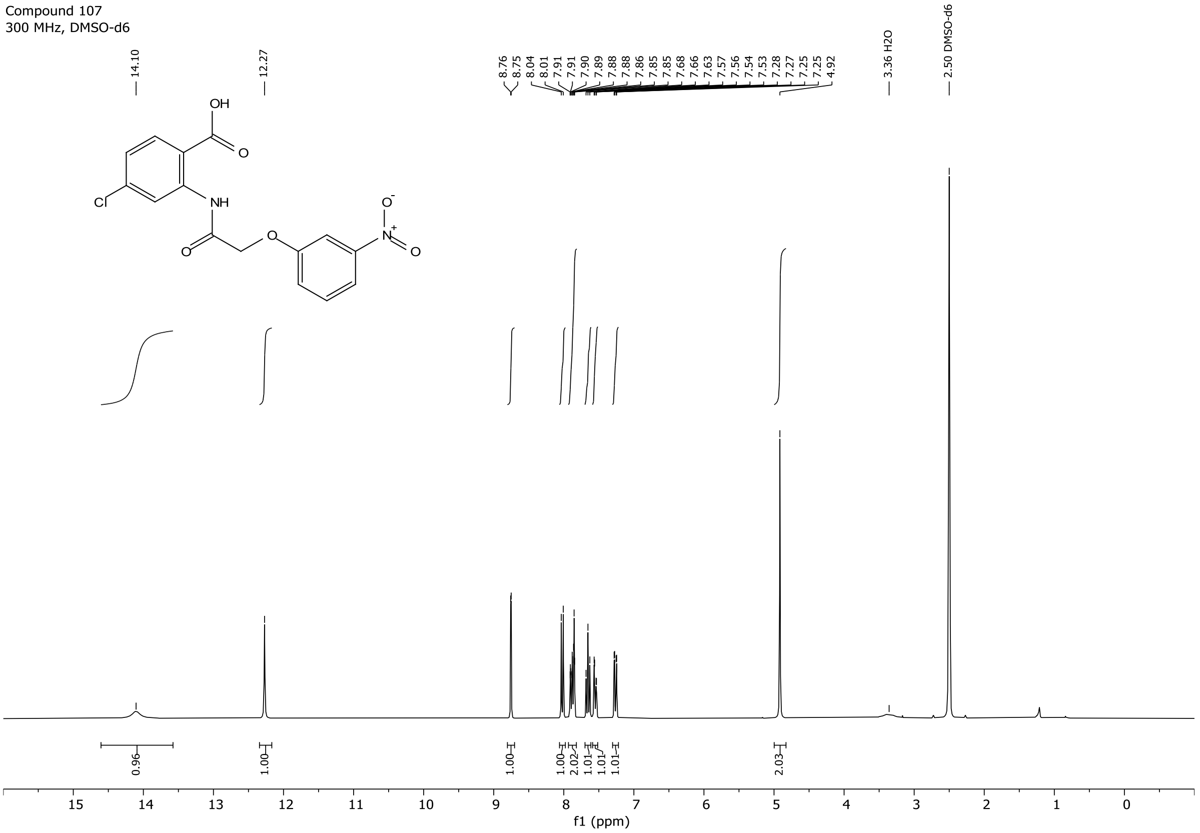

Compound 107
300 MHz, DMSO-d6
14.10
12.27
8.76
8.75
8.04
8.01
7.91
7.91
7.90
7.89
7.88
7.88
7.86
7.85
7.85
7.68
7.66
7.63
7.57
7.56
7.54
7.53
7.28
7.27
7.25
7.25
4.92
3.36 H2O
2.50 DMSO-d6
OH
O
Cl
NH
O
O
O-
N+
O
0.96
1.00
1.00
1.00
2.02
1.01
1.01
1.01
2.03
f1 (ppm)

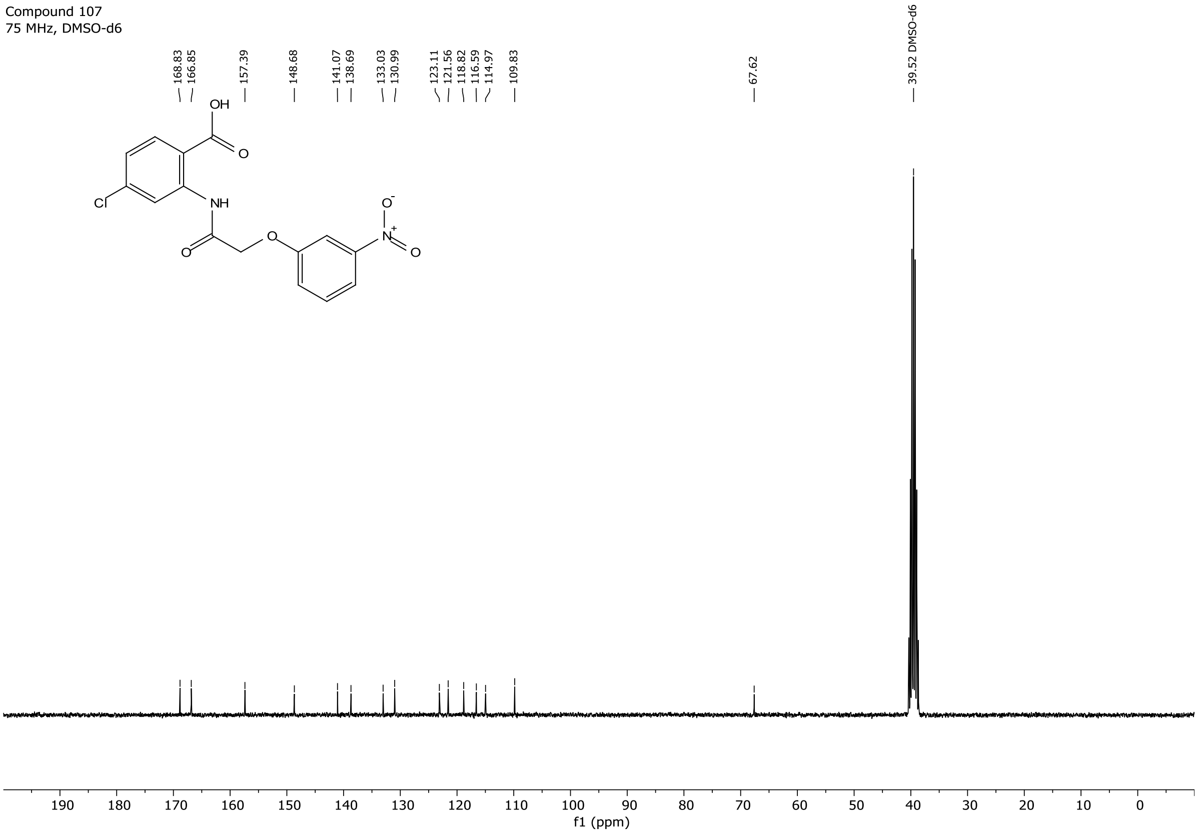

Compound 107
75 MHz, DMSO-d6
168.83
166.85
157.39
148.68
141.07
138.69
133.03
130.99
123.11
121.56
118.82
116.59
114.97
109.83
67.62
39.52 DMSO-d6
OH
O
Cl
NH
O
O
O-
N+
O
f1 (ppm)

Compound 108
300 MHz, DMSO-d6

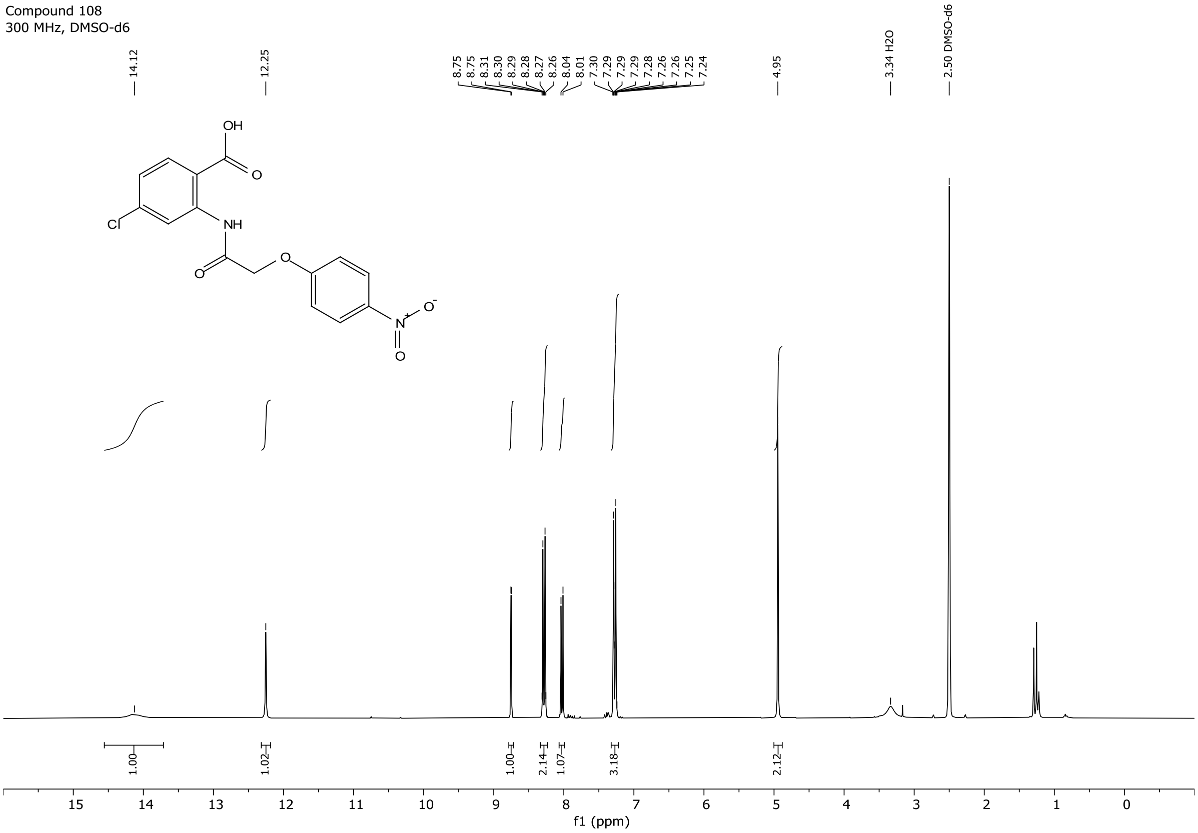

Compound 108
75 MHz, DMSO-d6

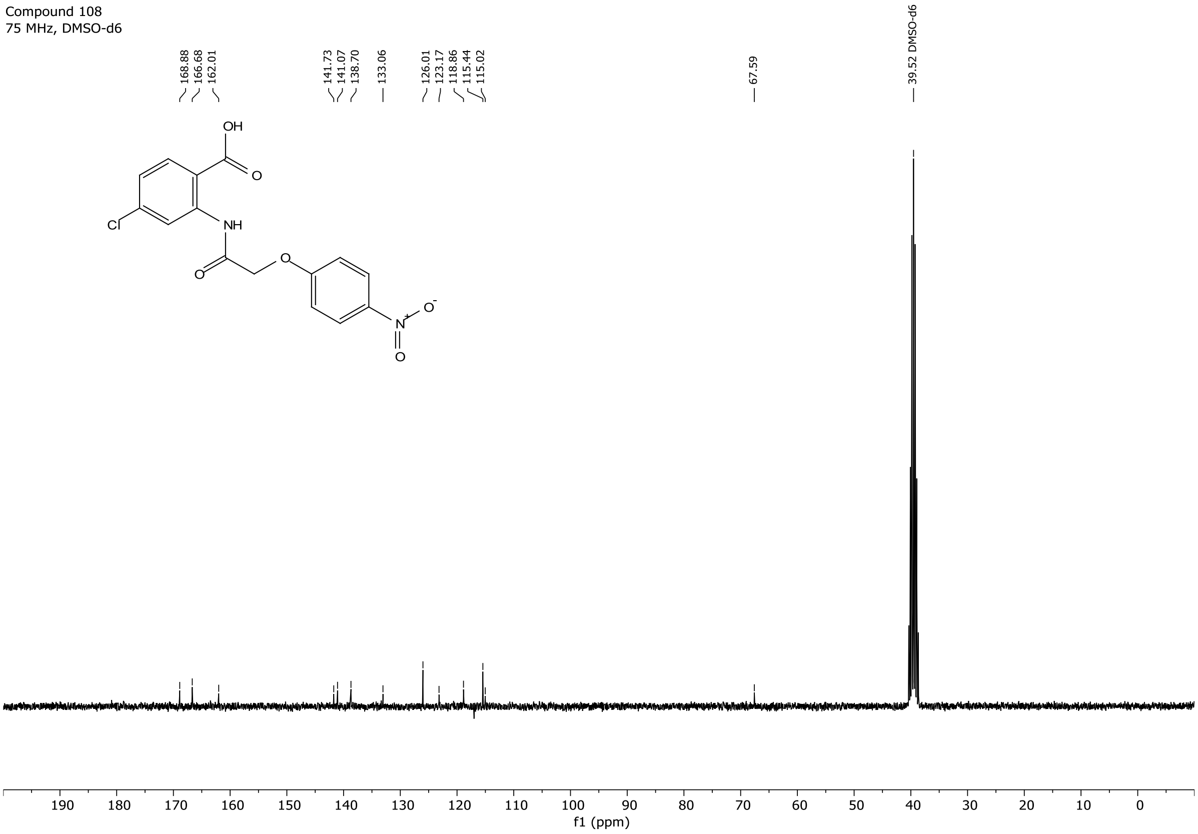

Compound 109
300 MHz, DMSO-d6

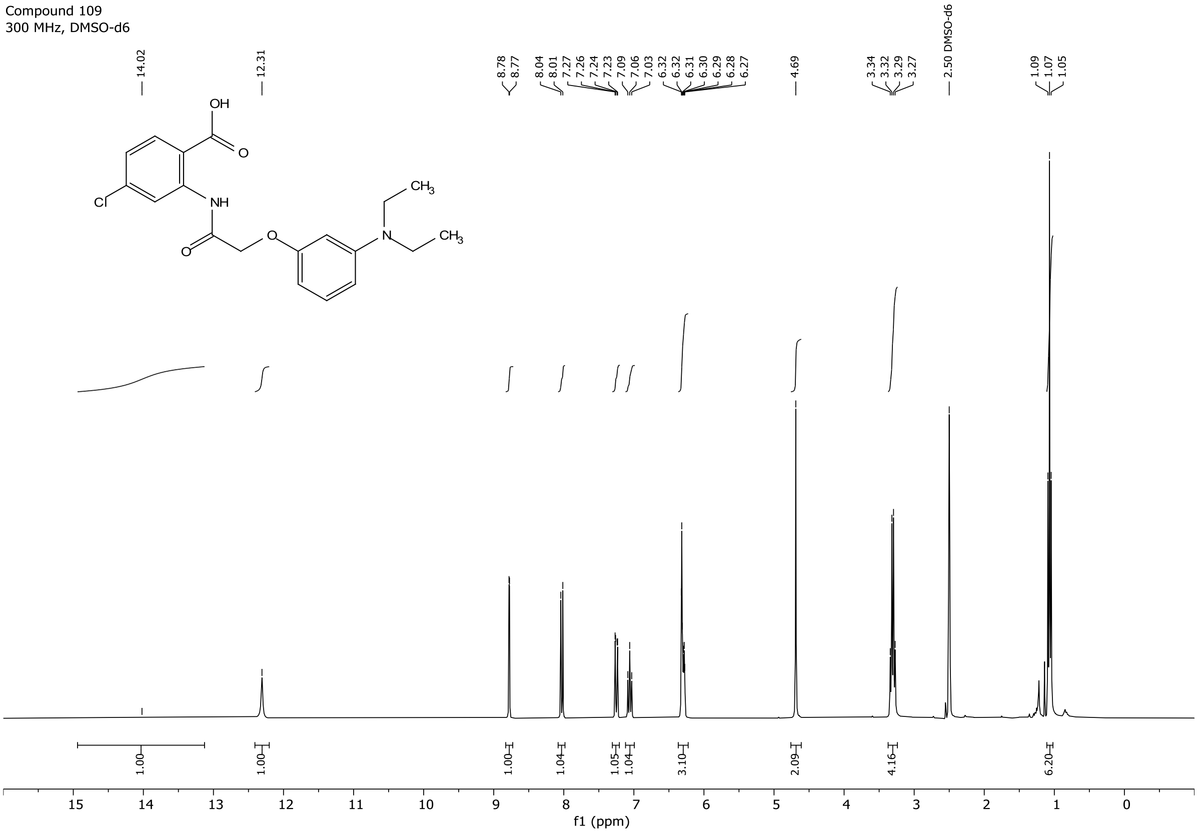

Compound 109
75 MHz, DMSO-d6

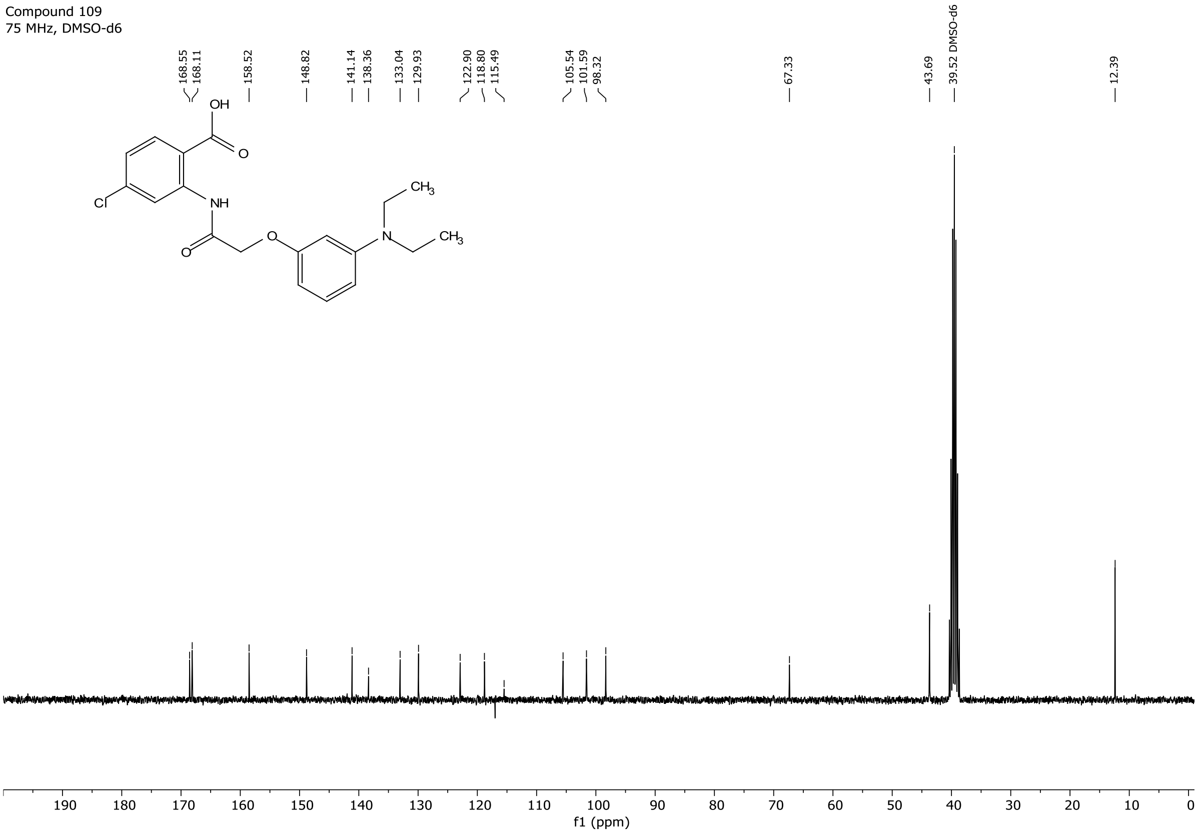

Compound 110
300 MHz, DMSO-d6

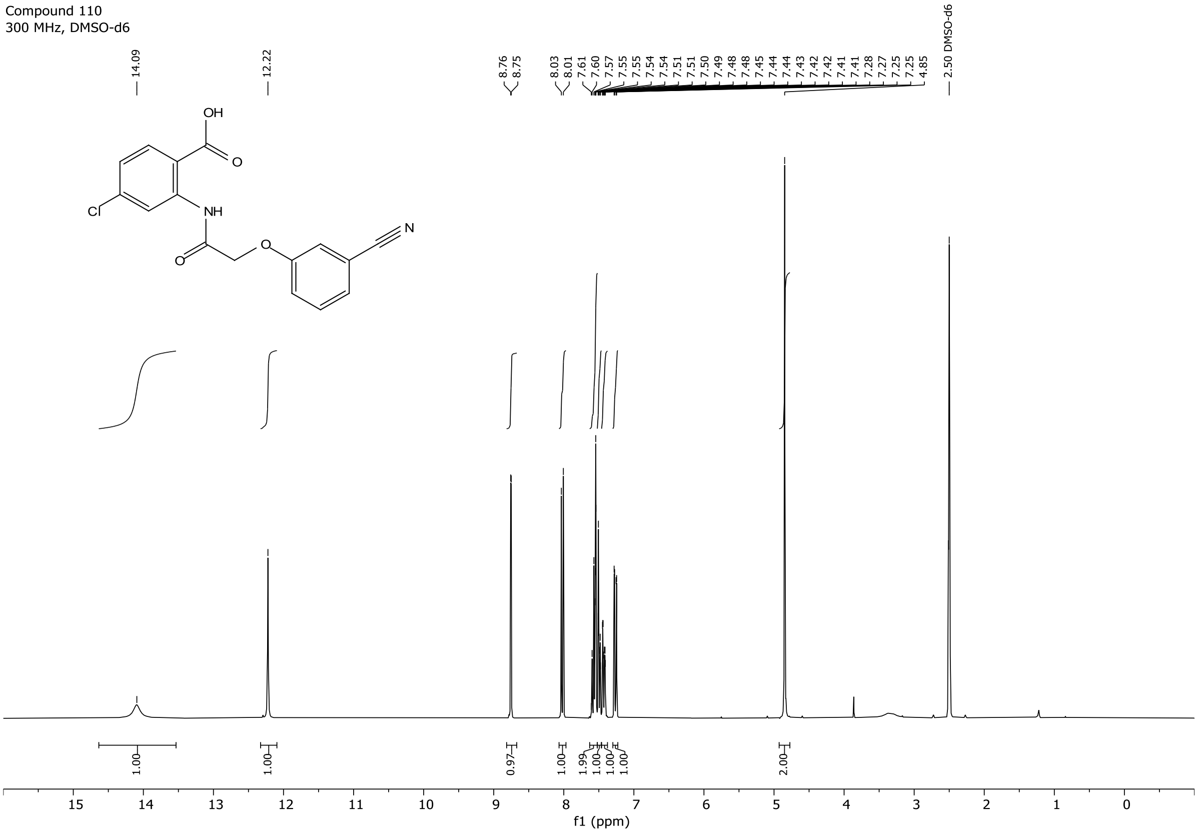

Compound 110
75 MHz, DMSO-d6

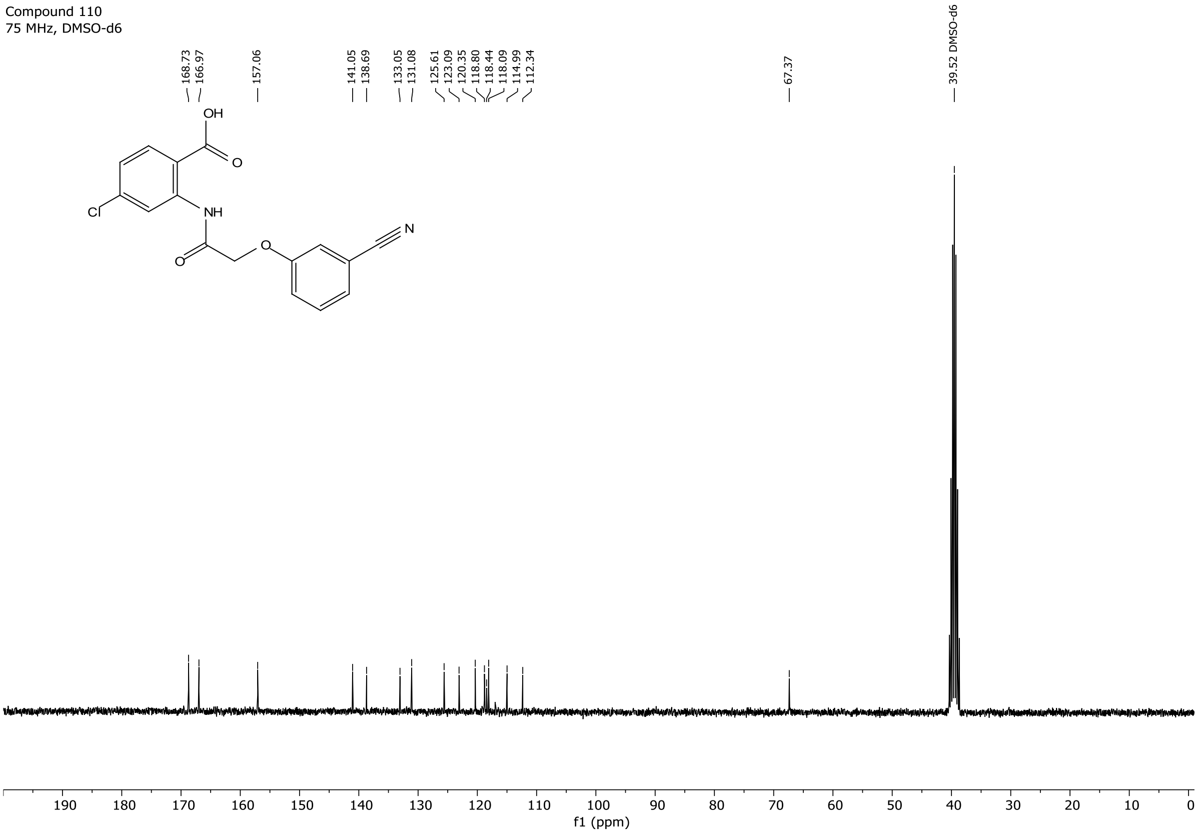

Compound 111
300 MHz, DMSO-d6

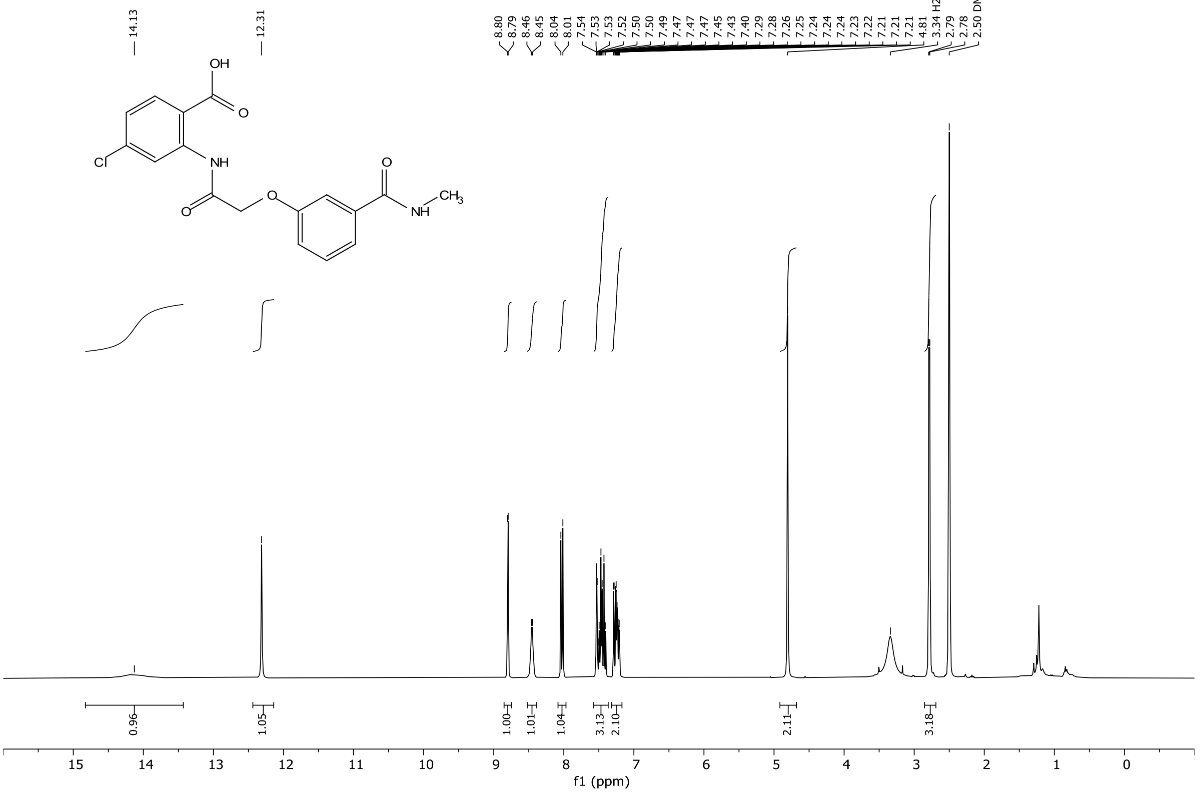

Compound 111
75 MHz, DMSO-d6

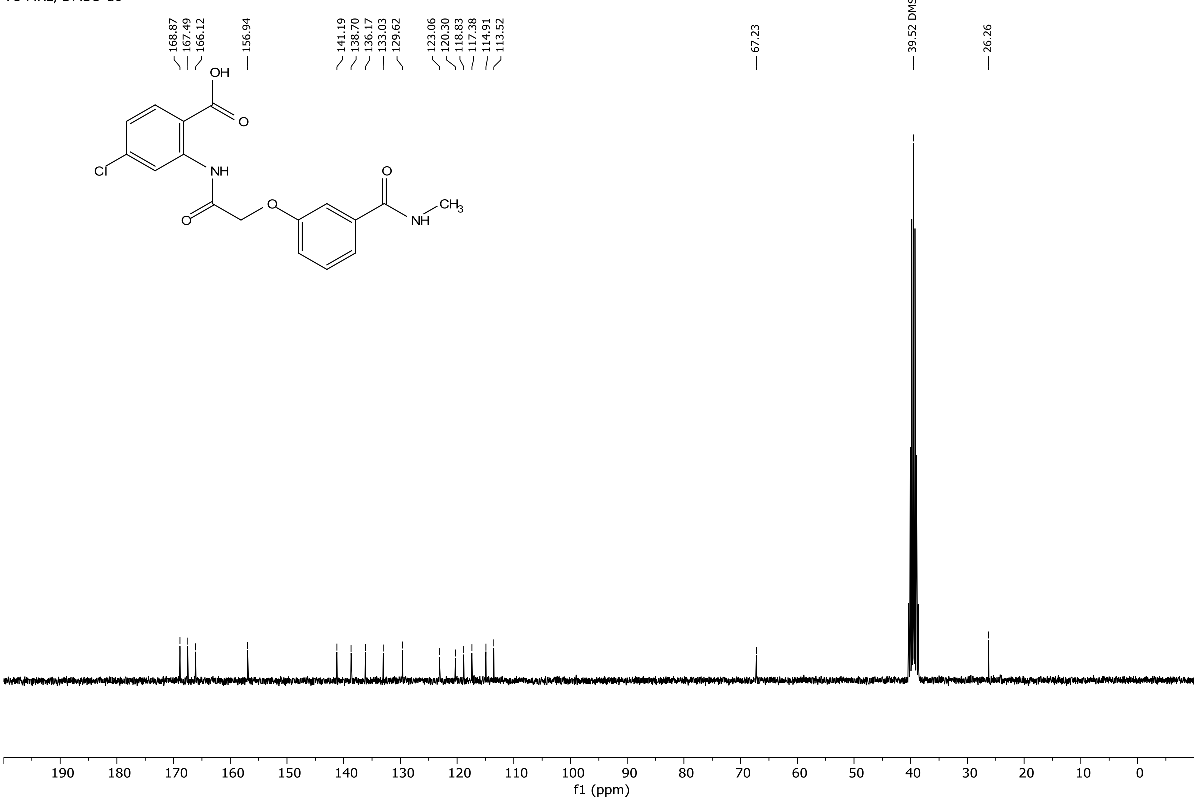

Compound 112
300 MHz, DMSO-d6

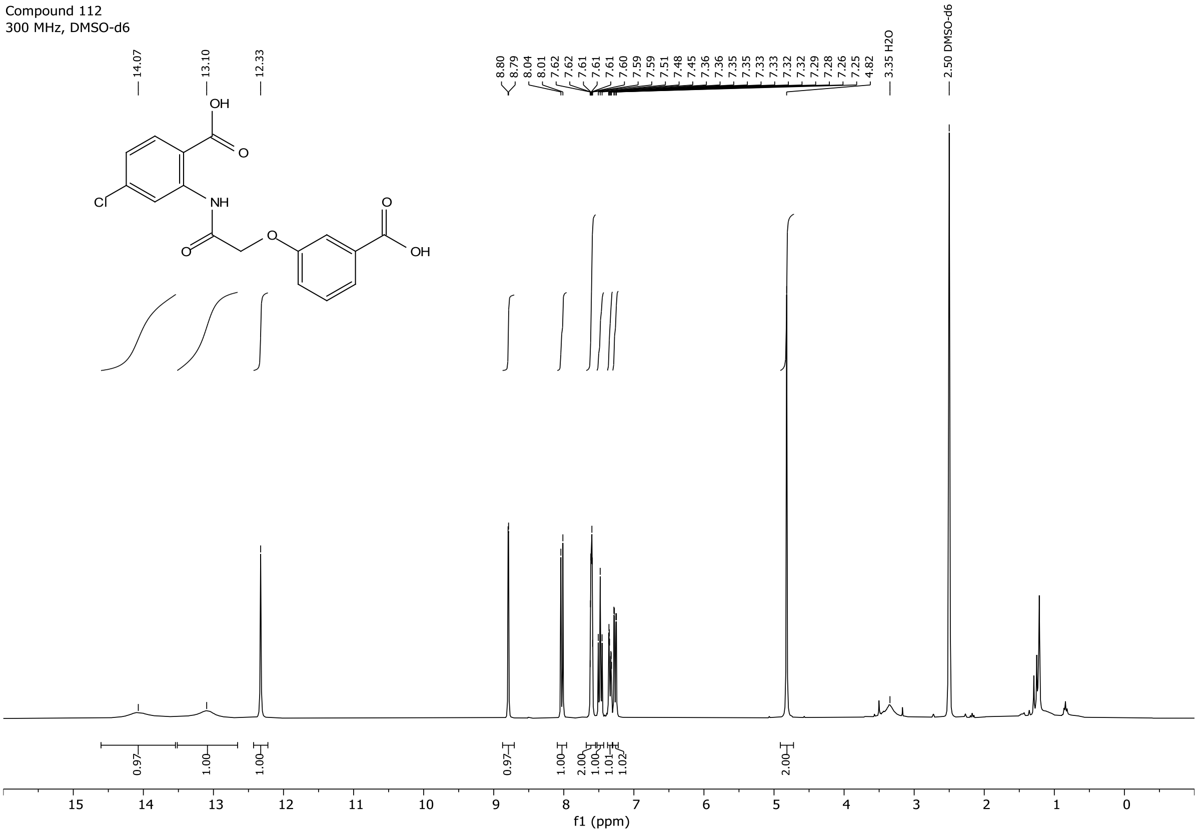

Compound 112
75 MHz, DMSO-d6

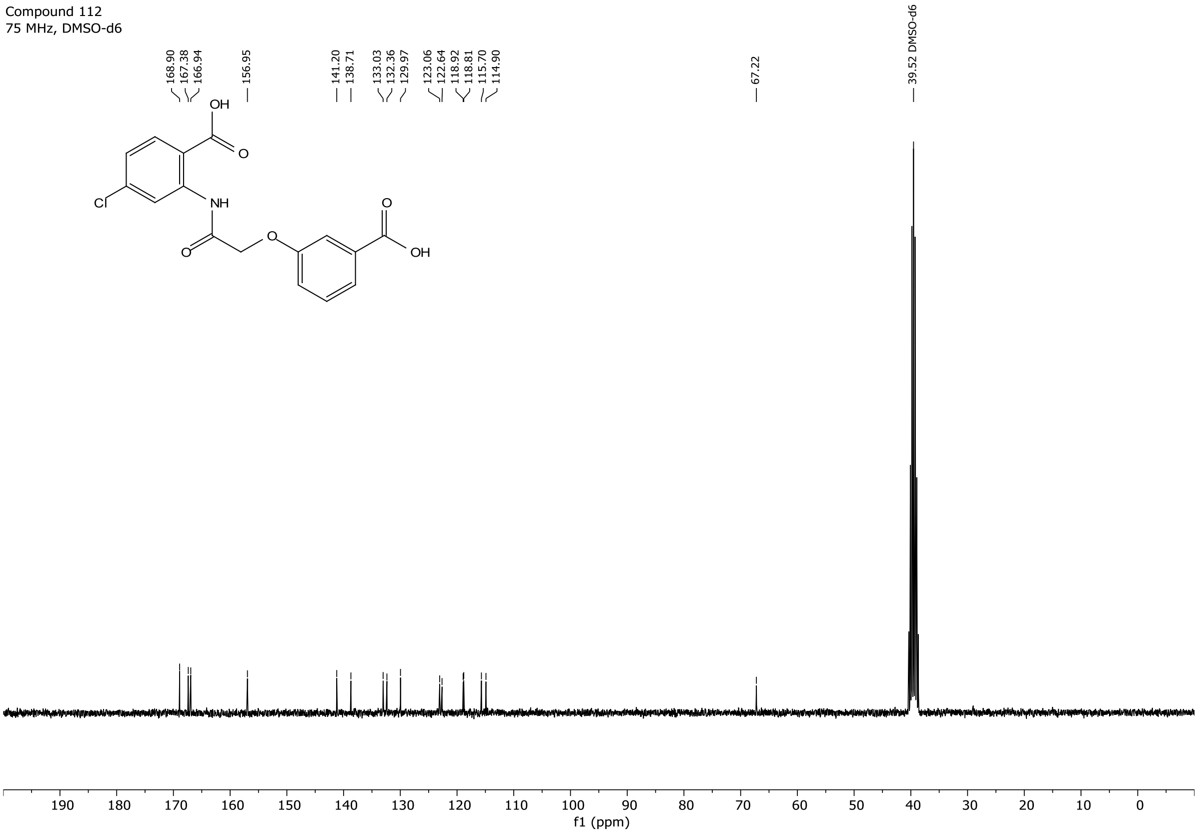

Compound 113
300 MHz, DMSO-d6

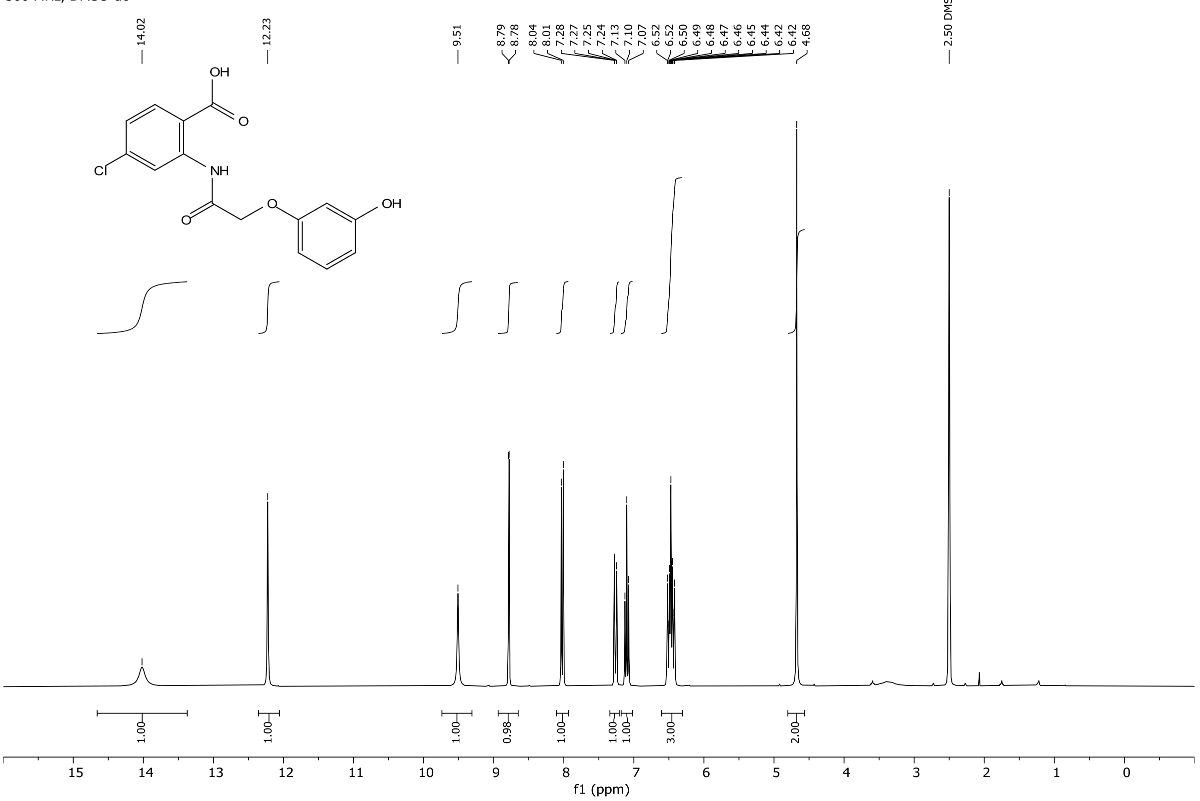

Compound 113
75 MHz, DMSO-d6

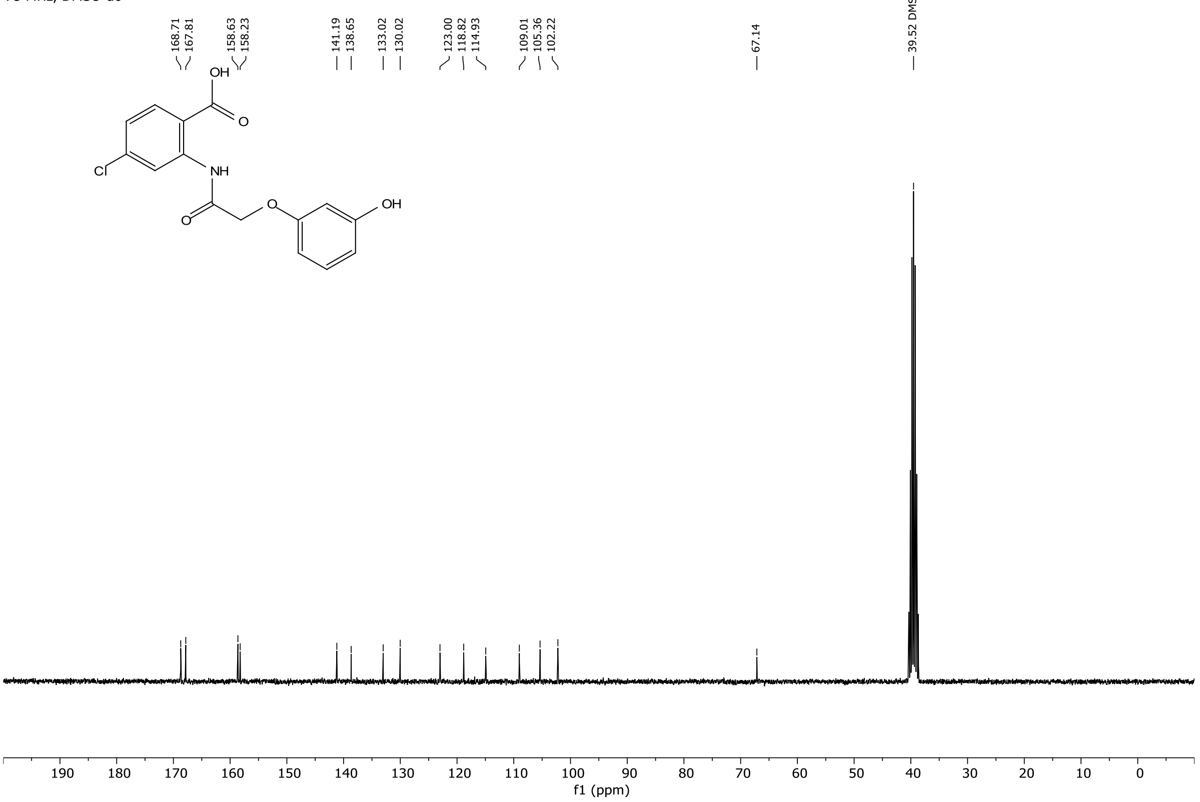

Compound 114
300 MHz, DMSO-d6

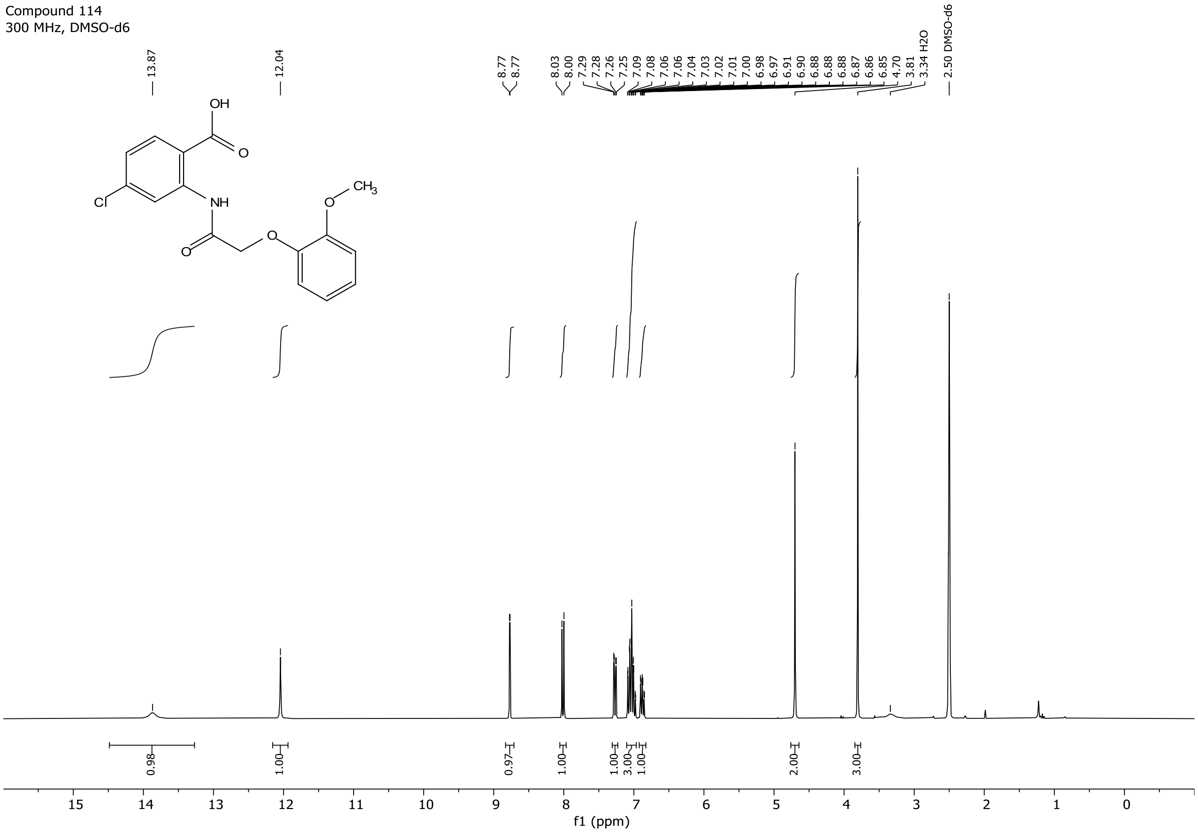

Compound 114
75 MHz, DMSO-d6

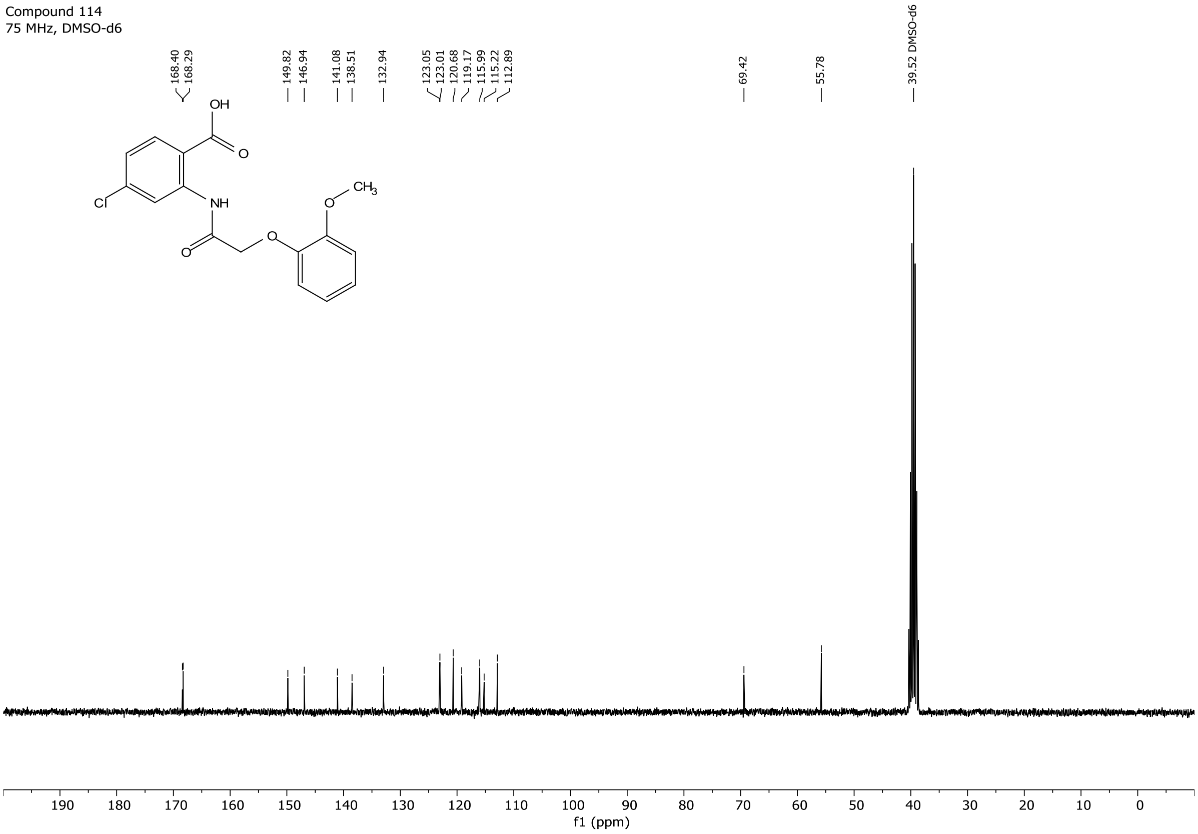

Compound 115
300 MHz, DMSO-d6

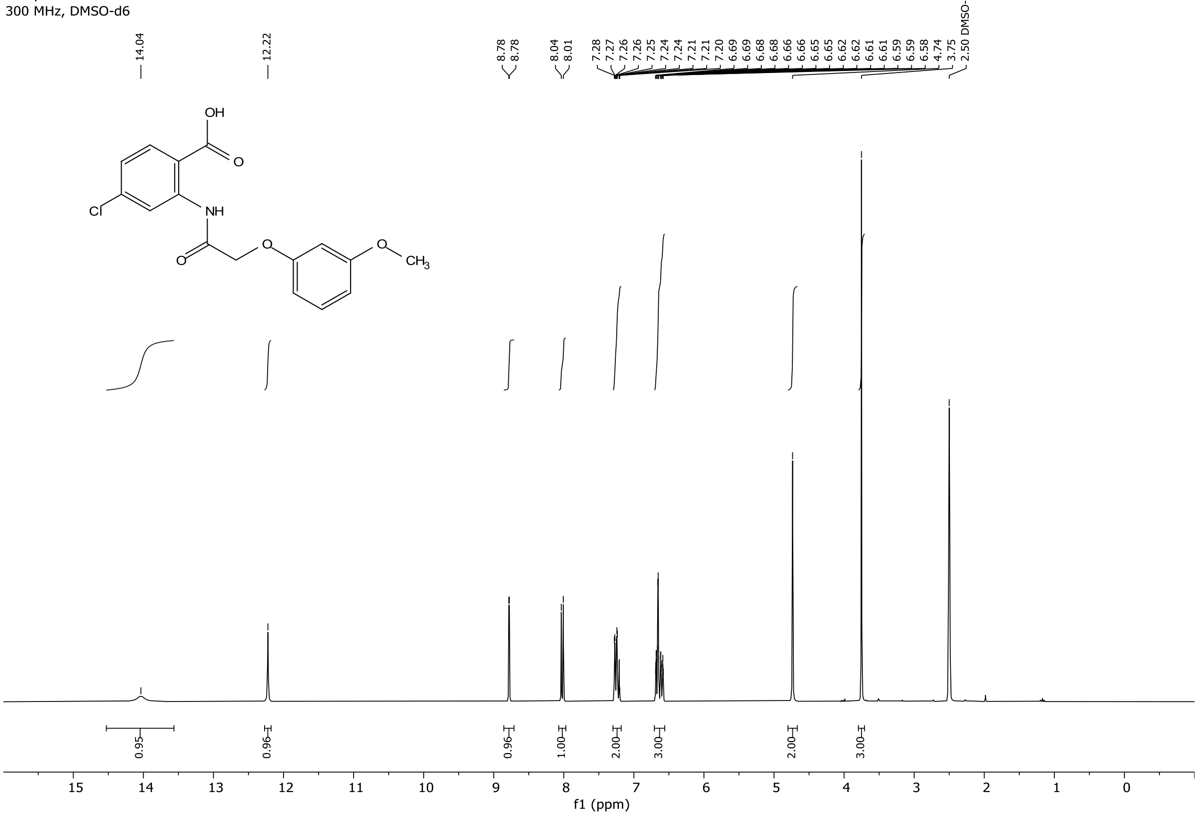

Compound 115
75 MHz, DMSO-d6

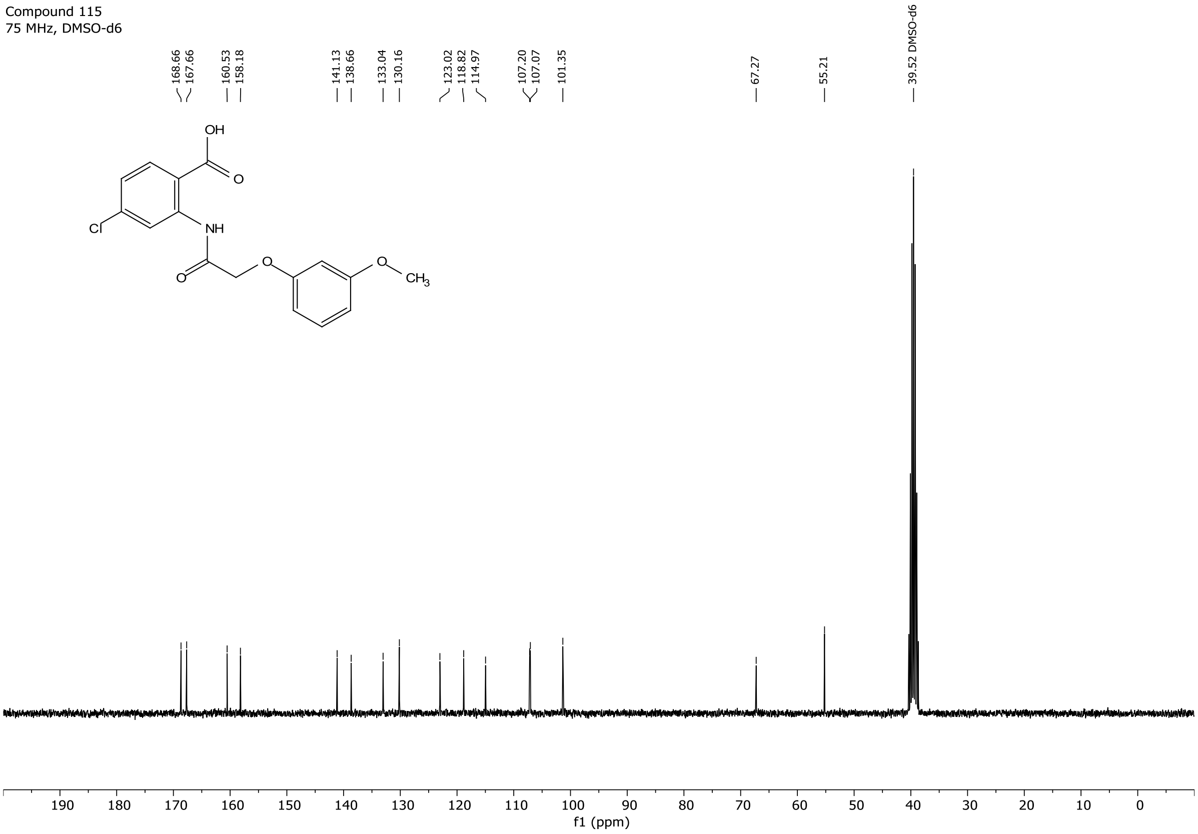

Compound 116
300 MHz, DMSO-d6

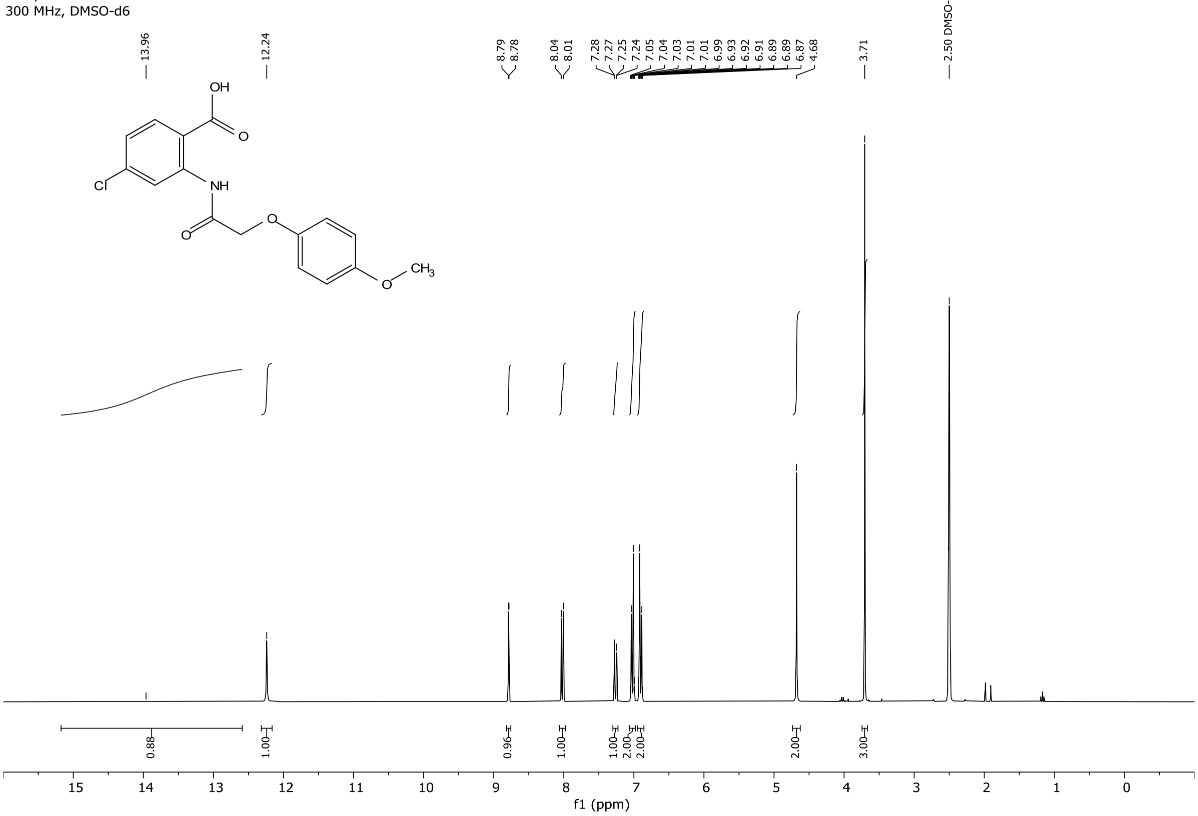

Compound 116
75 MHz, DMSO-d6

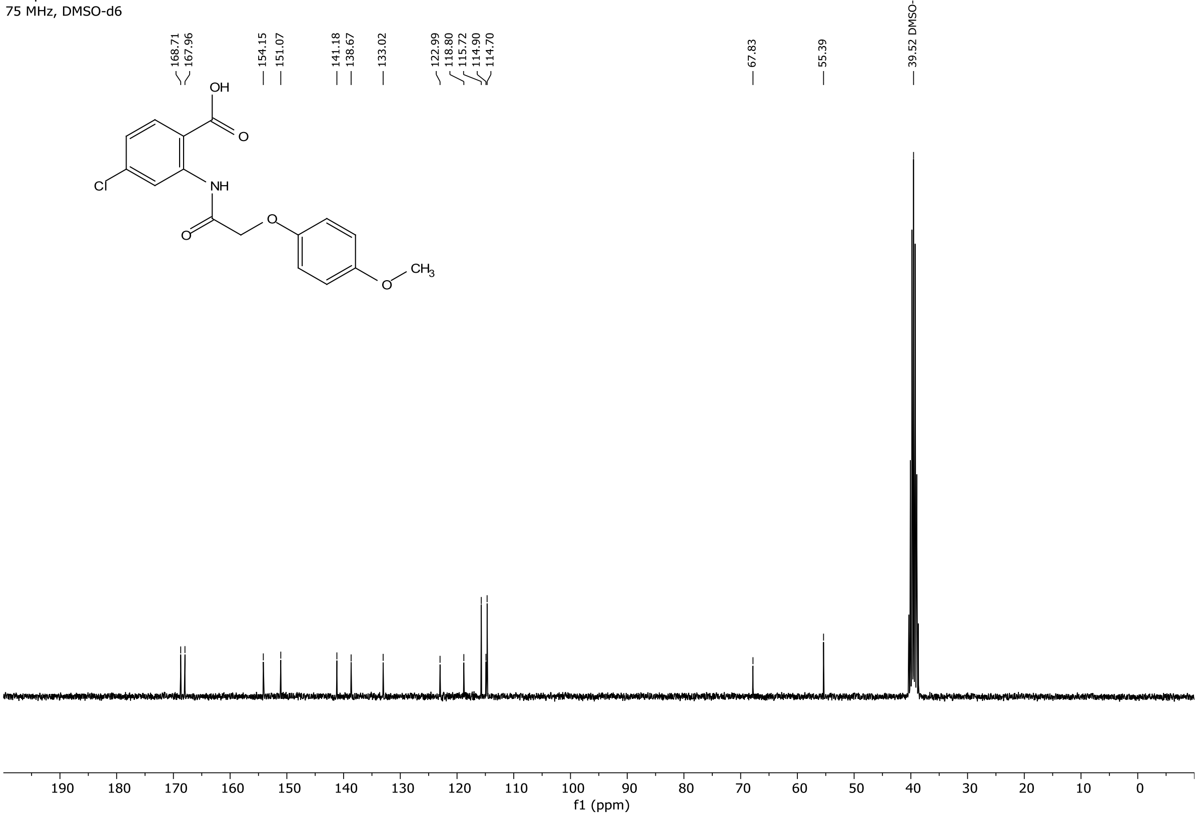

Compound 117
300 MHz, DMSO-d6

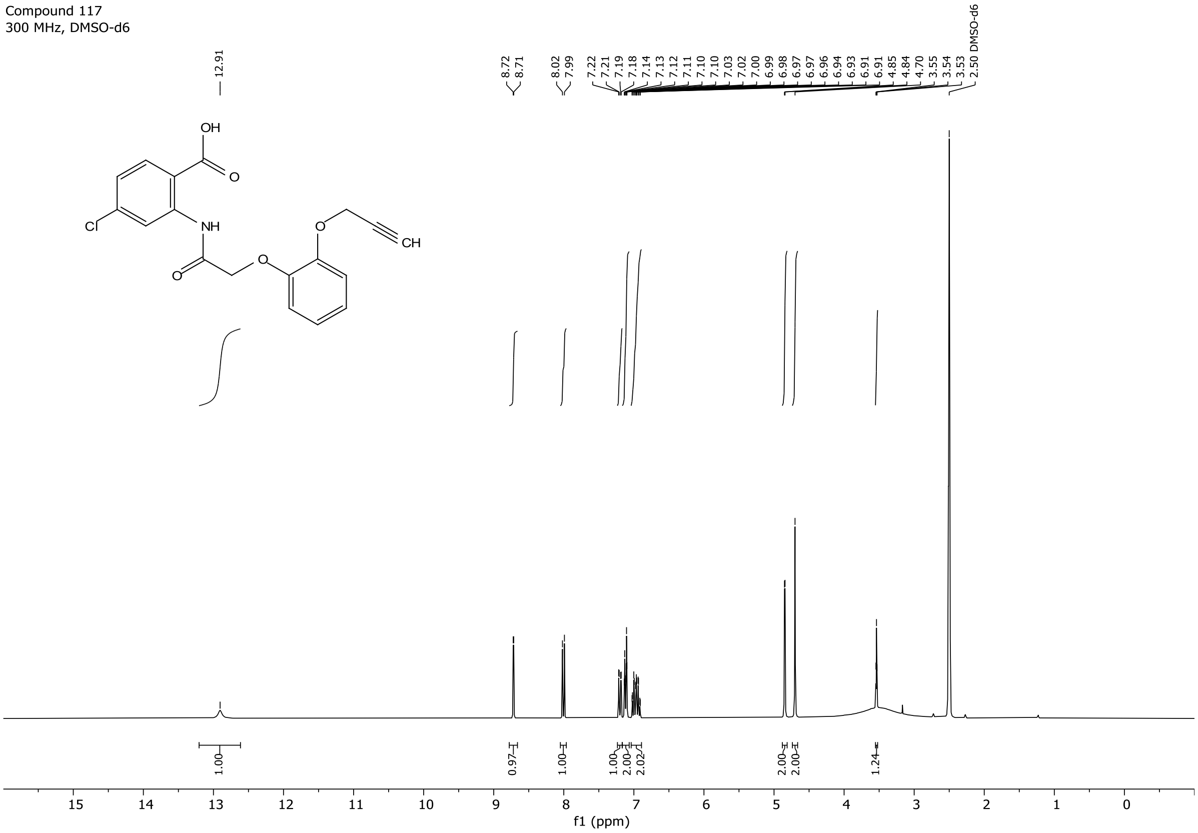

Compound 117
75 MHz, DMSO-d6

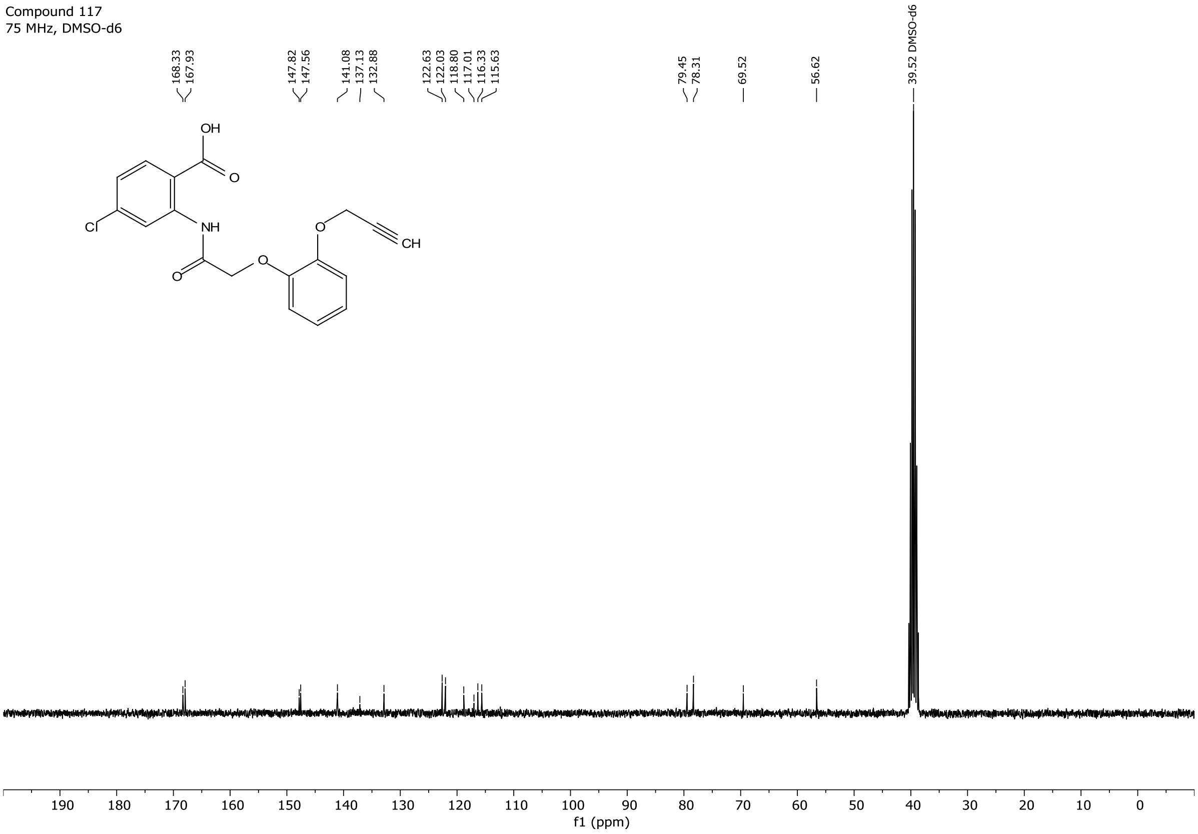

Compound 118 (PBA)
300 MHz, DMSO-d6

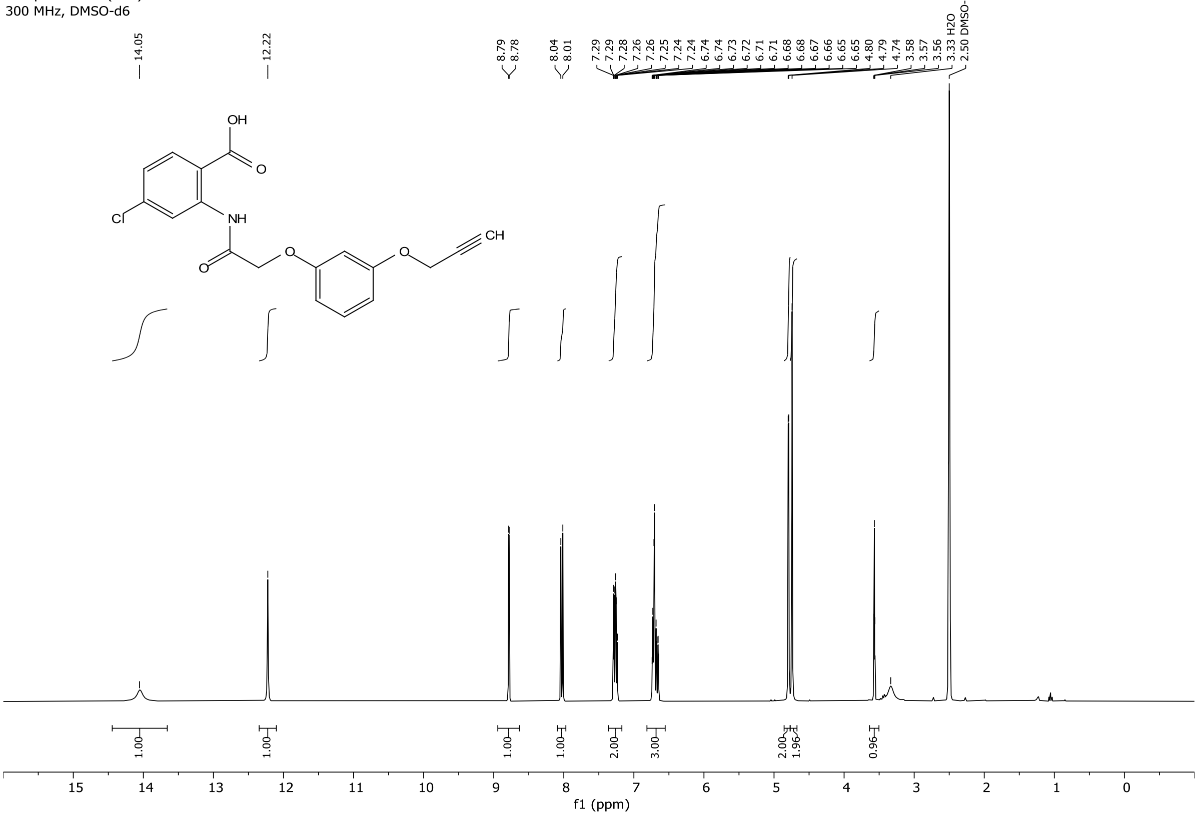

Compound 118 (PBA)
75 MHz, DMSO-d6

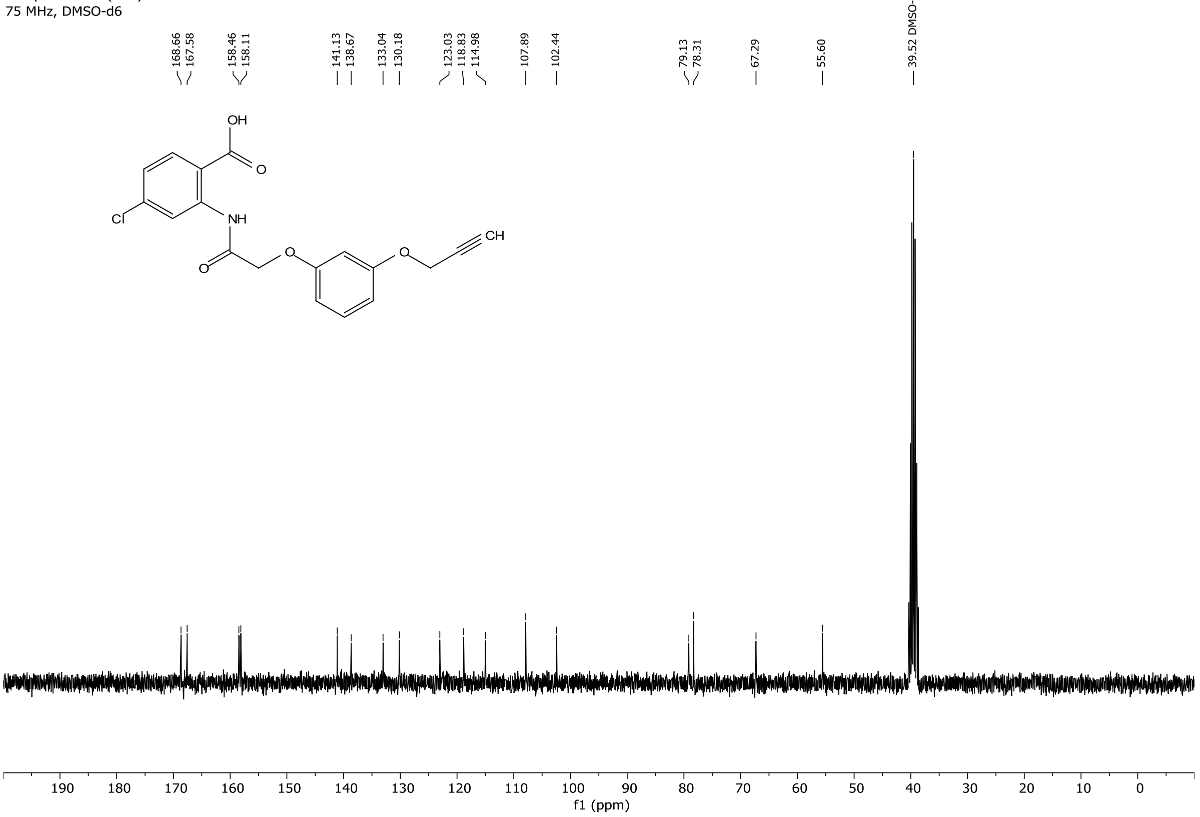

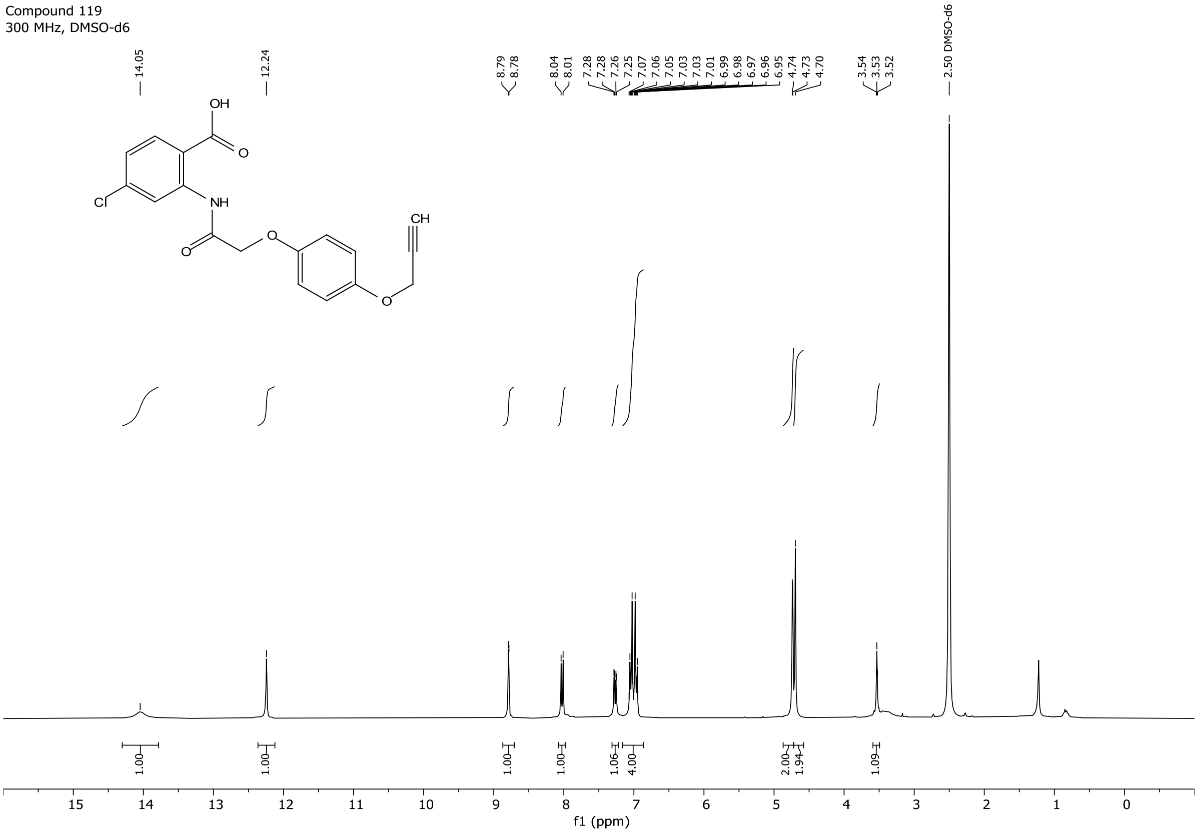
Compound 119
300 MHz, DMSO-d6
f1 (ppm)

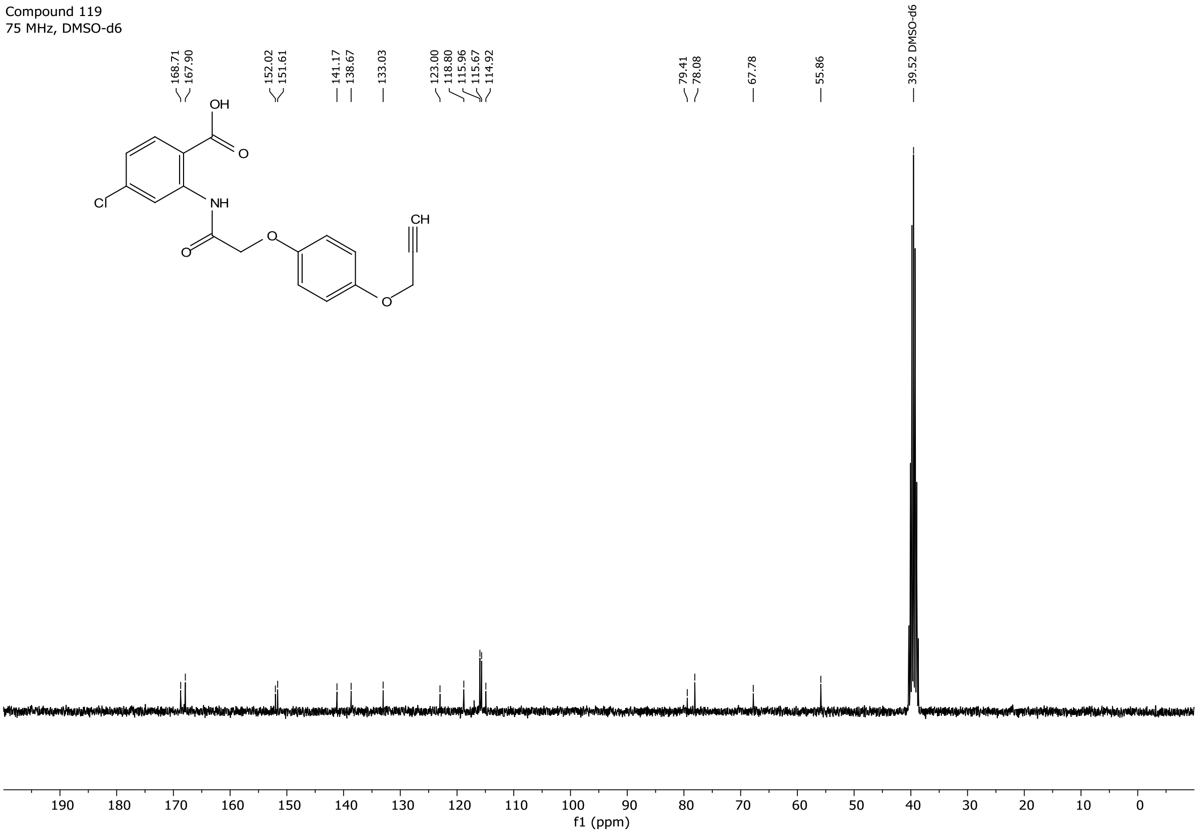
Compound 119
75 MHz, DMSO-d6
f1 (ppm)

Compound 120
300 MHz, DMSO-d6

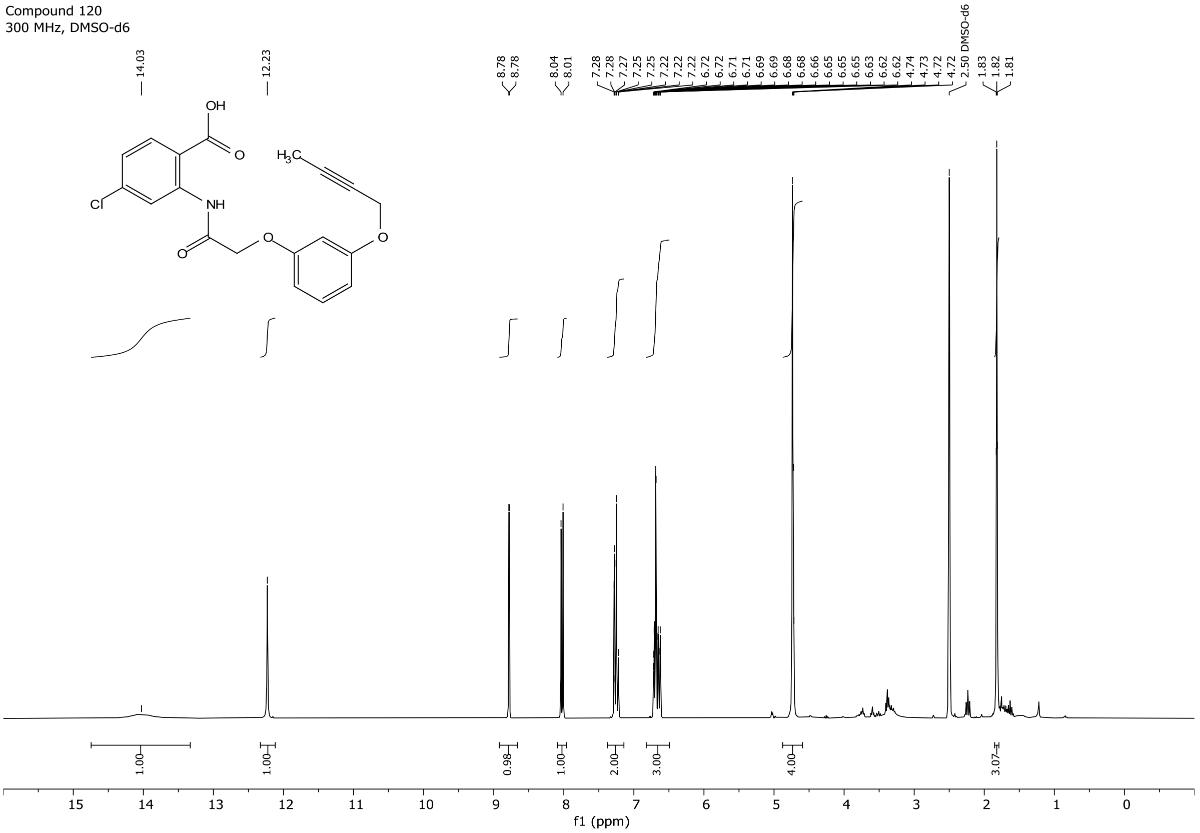

Compound 120
75 MHz, DMSO-d6

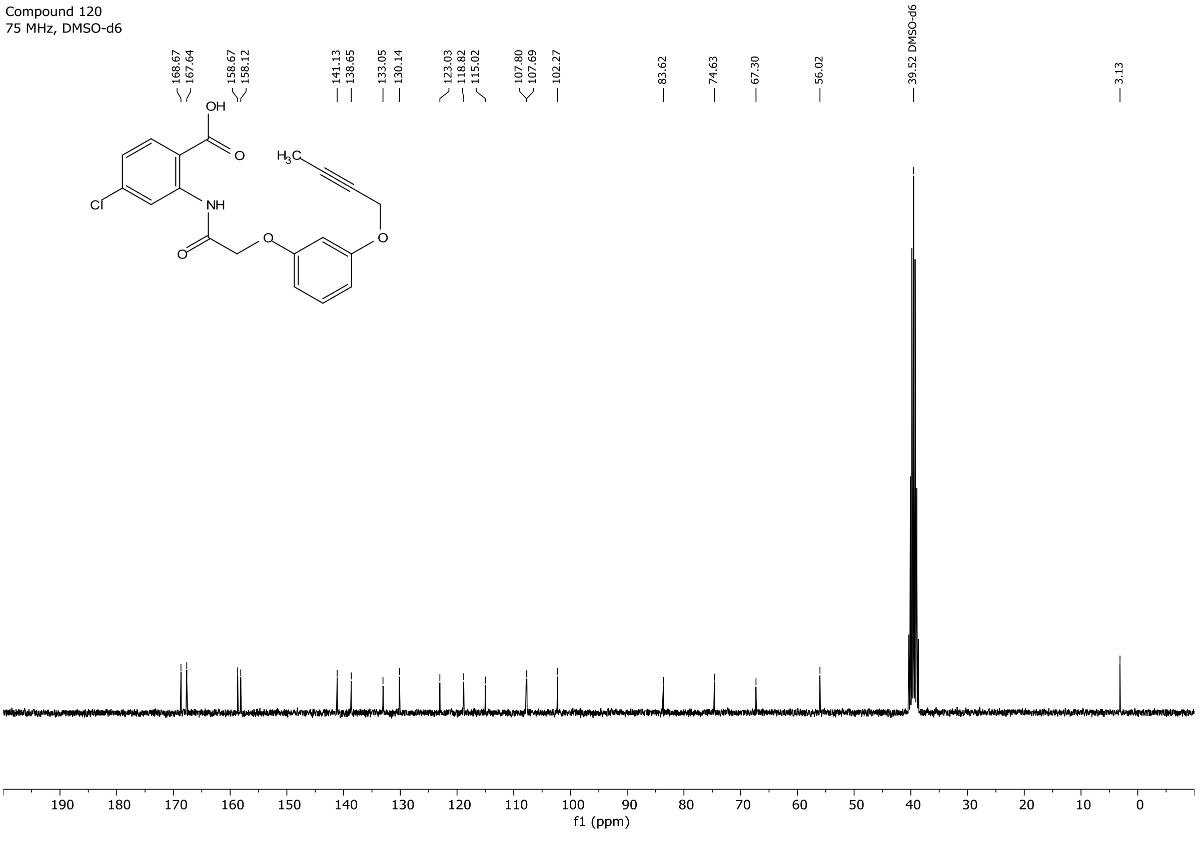

Compound 121
300 MHz, DMSO-d6

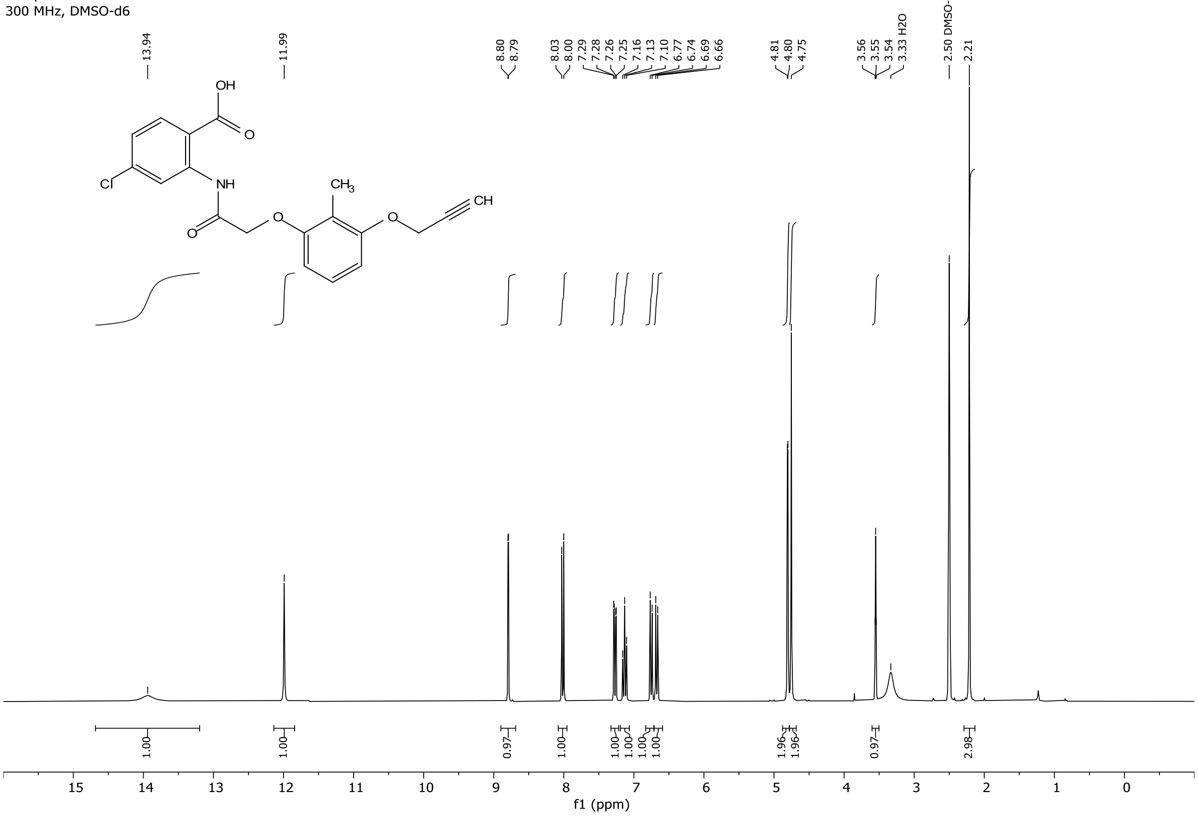

Compound 121
75 MHz, DMSO-d6

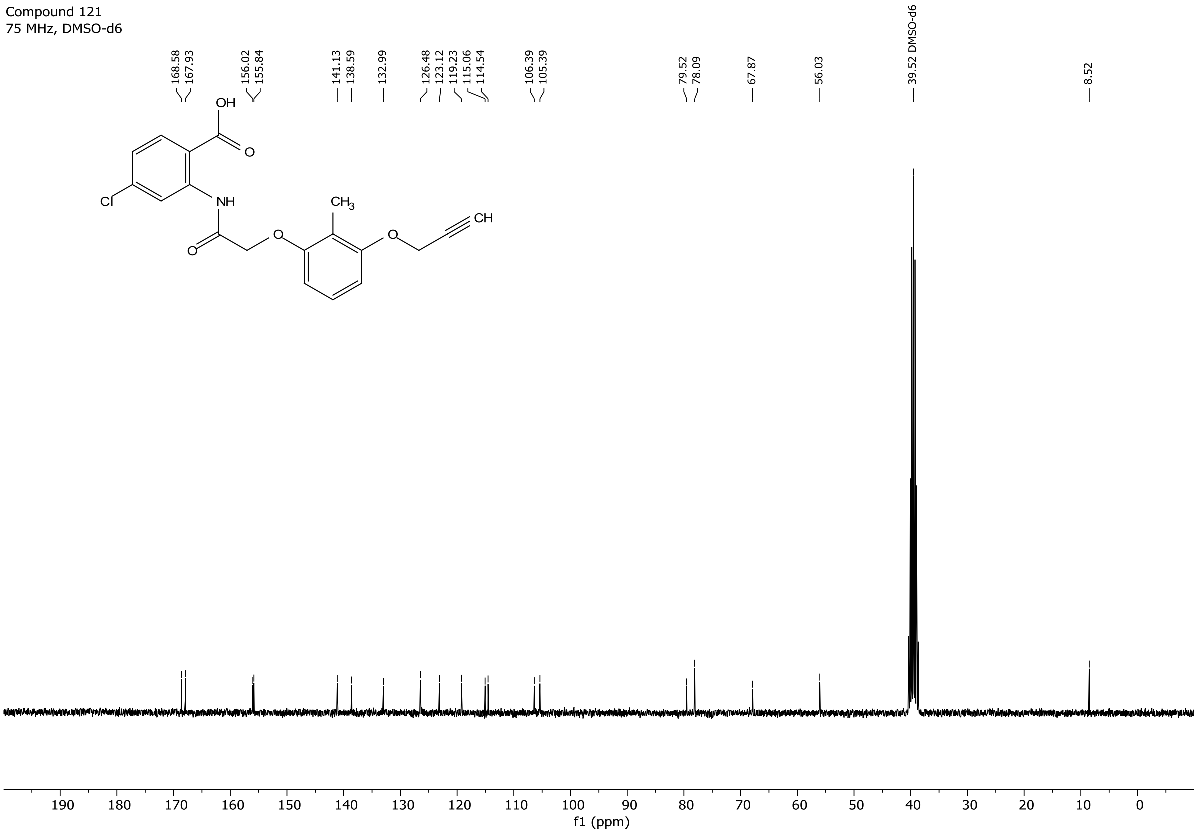

Compound 122
300 MHz, DMSO-d6

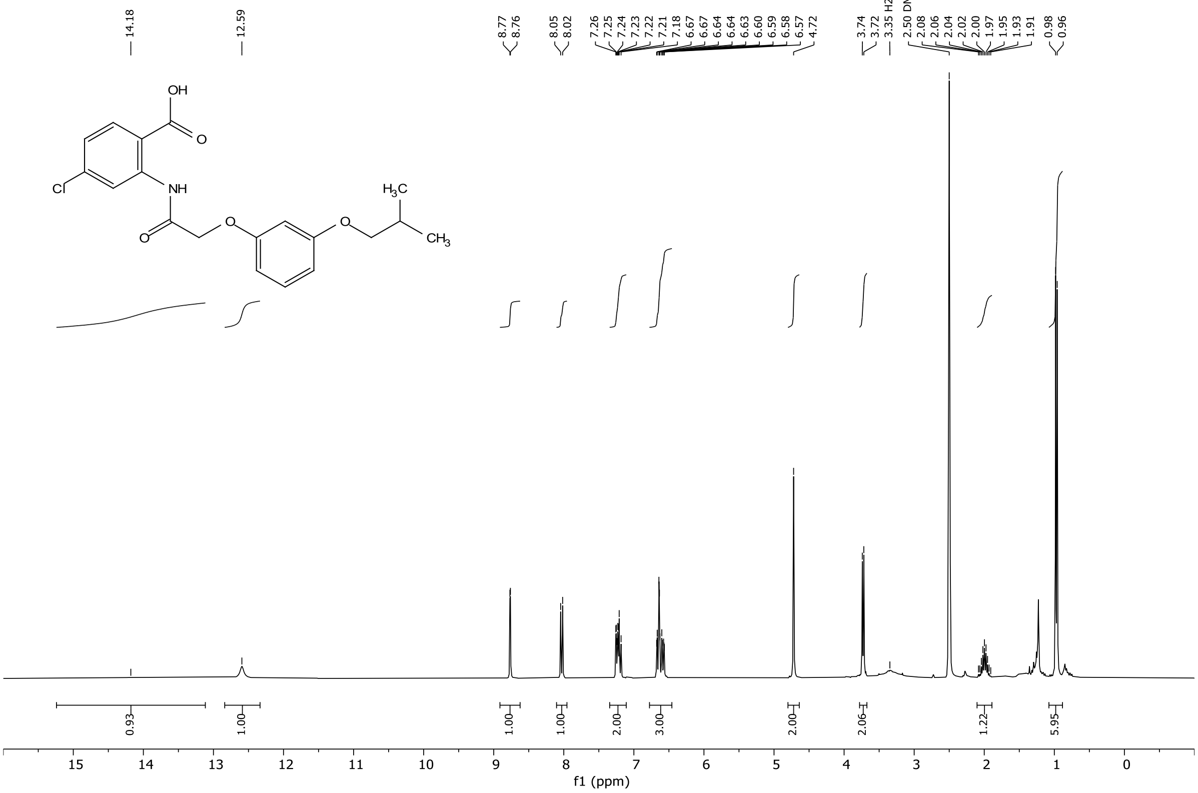

Compound 122
75 MHz, DMSO-d6

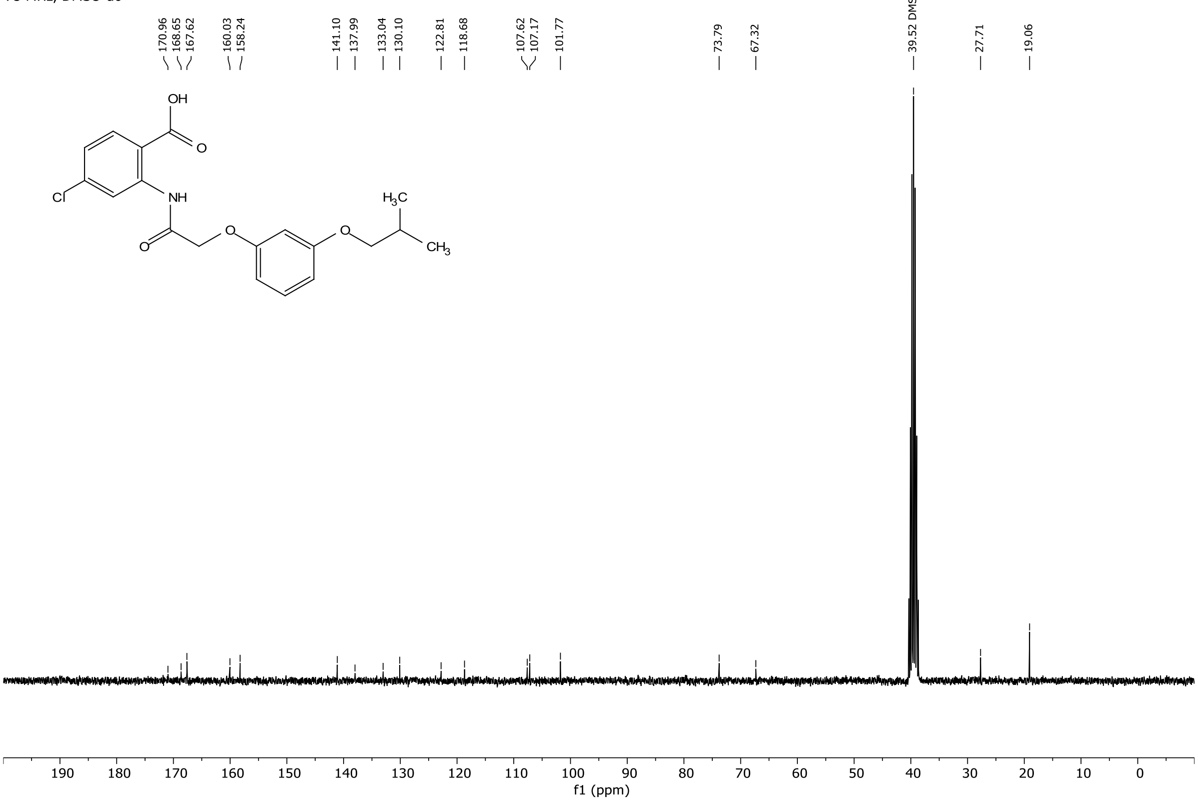

Compound 123
300 MHz, DMSO-d6

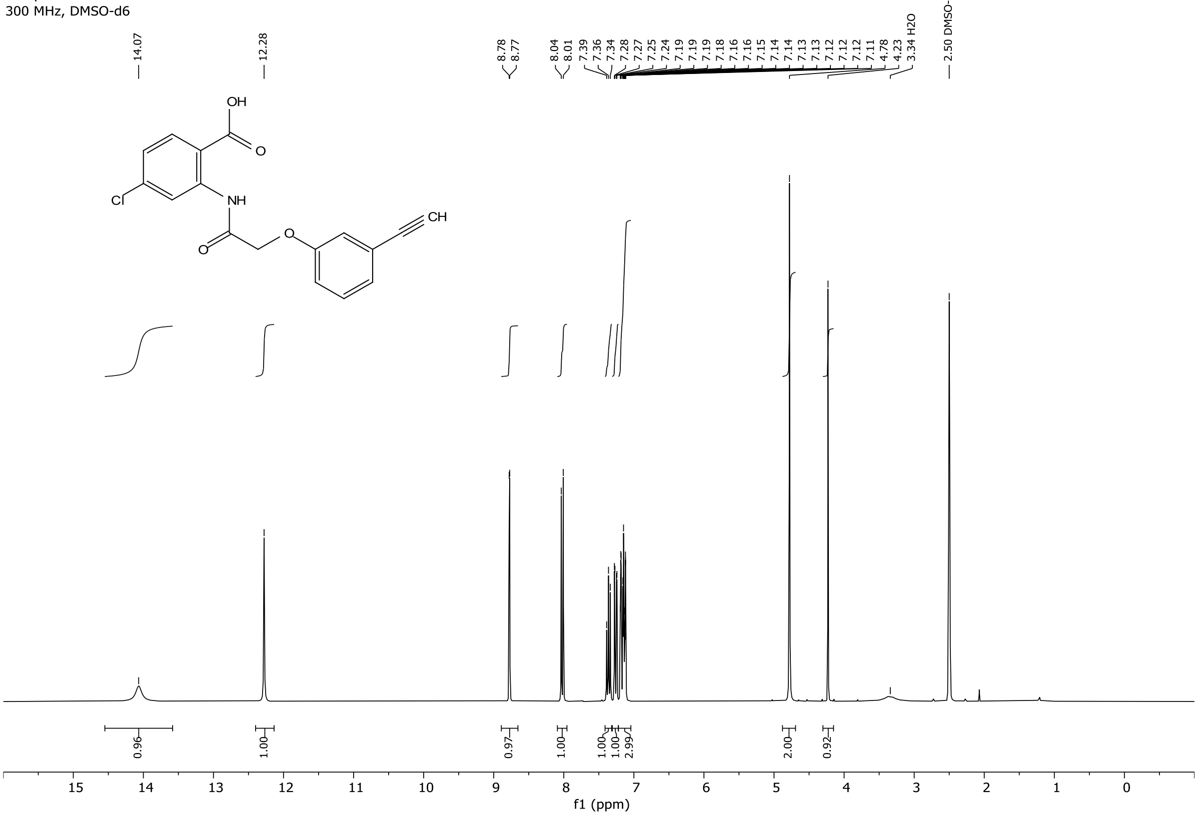

Compound 123
75 MHz, DMSO-d6

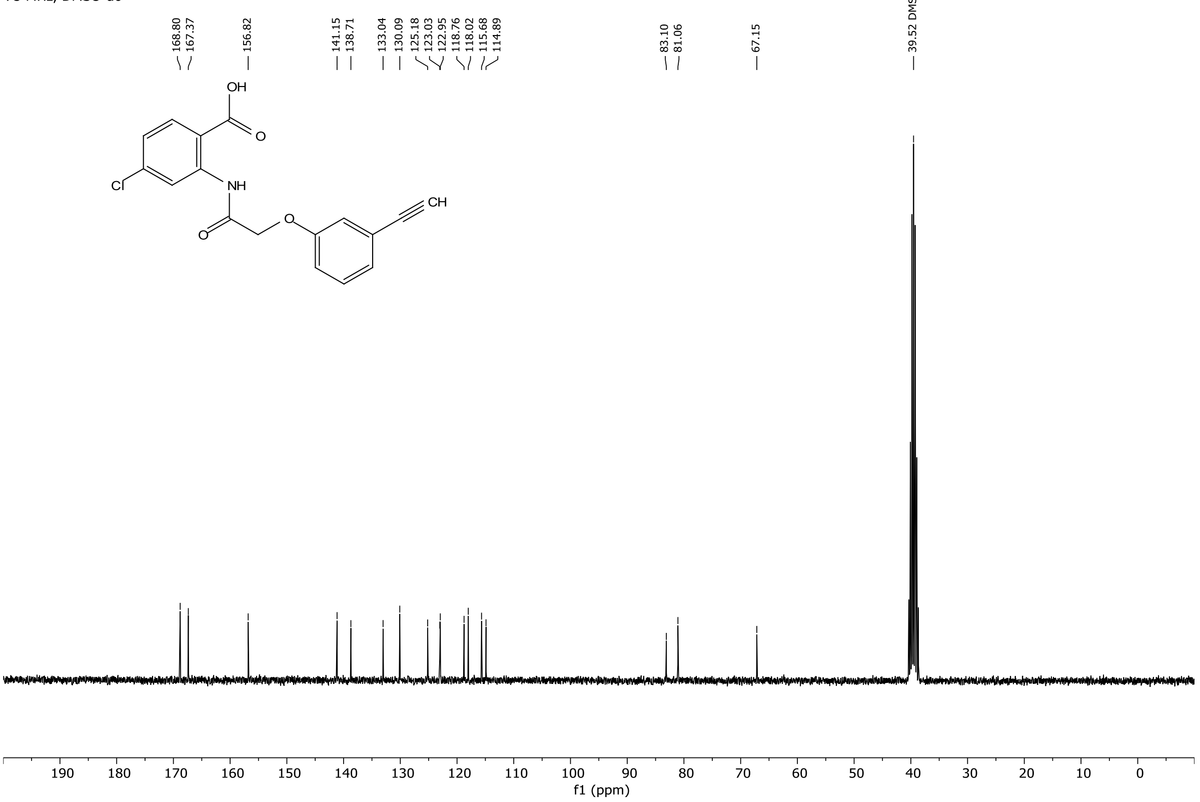

Compound 124
300 MHz, DMSO-d6

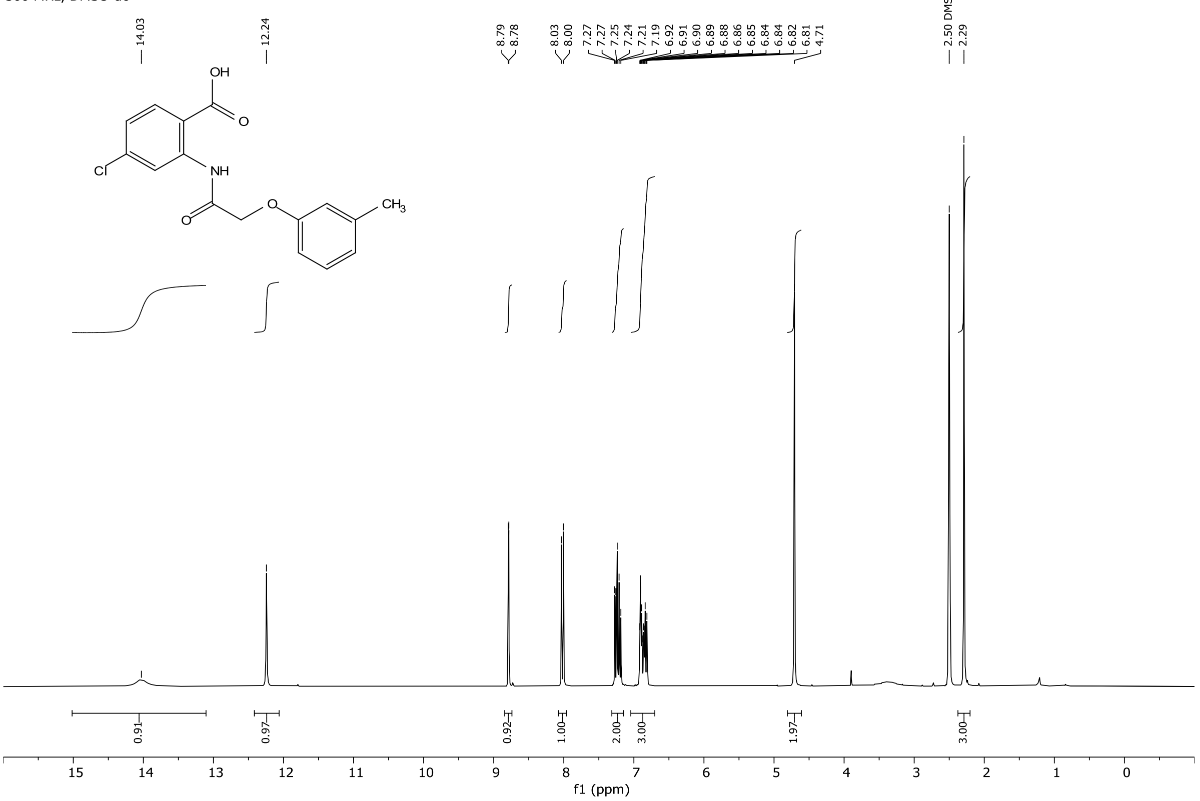

Compound 124
75 MHz, DMSO-d6

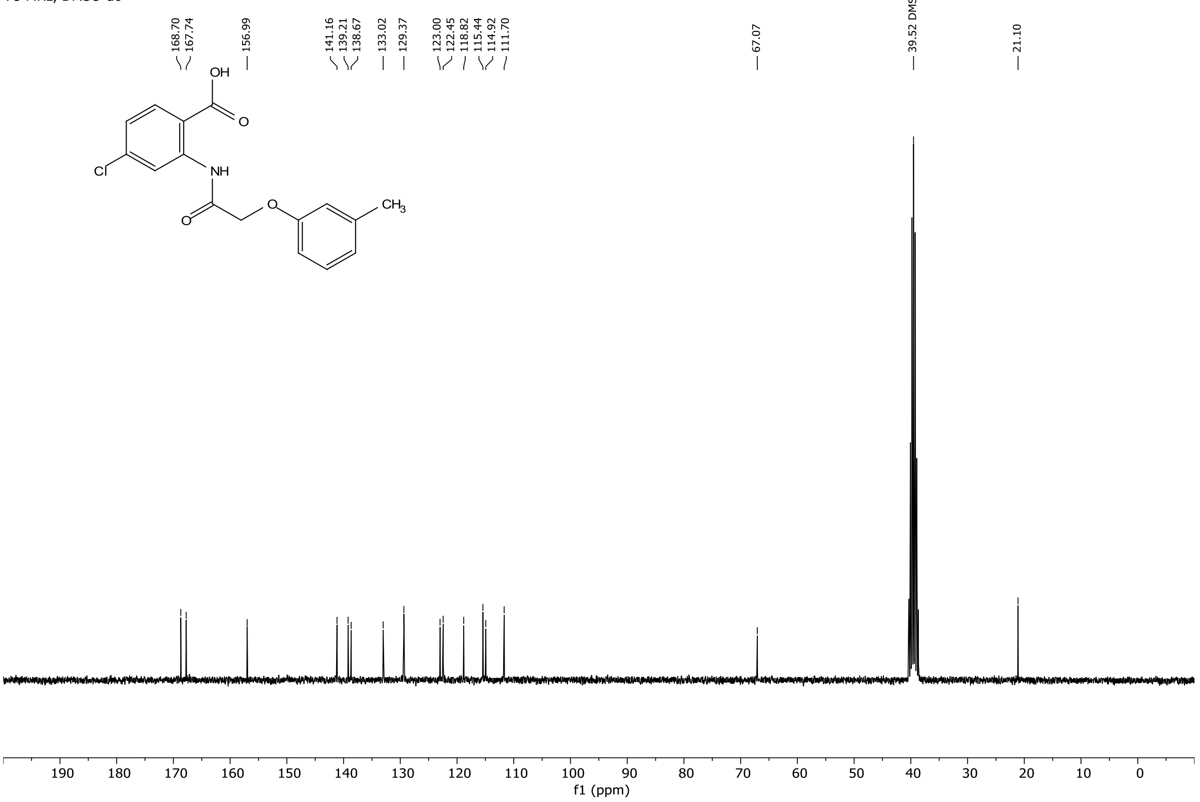

Compound 125
300 MHz, DMSO-d6

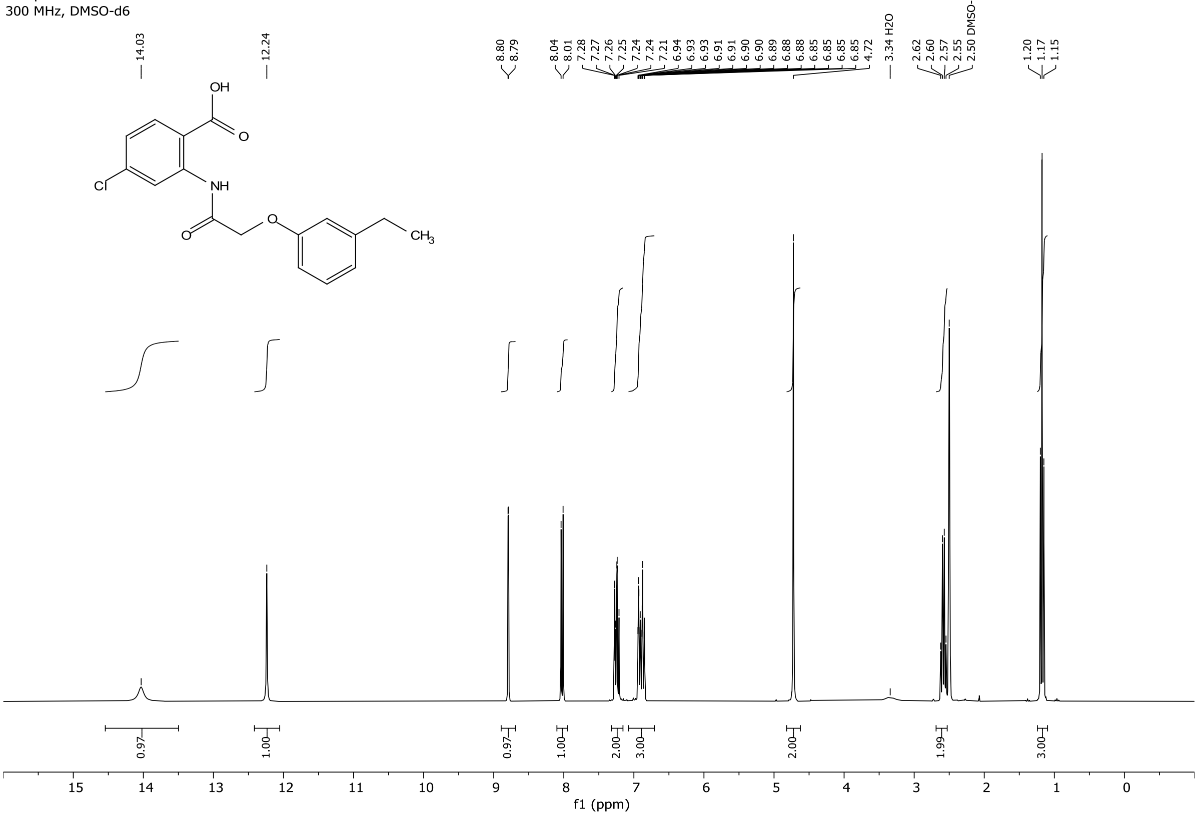

Compound 125
75 MHz, DMSO-d6

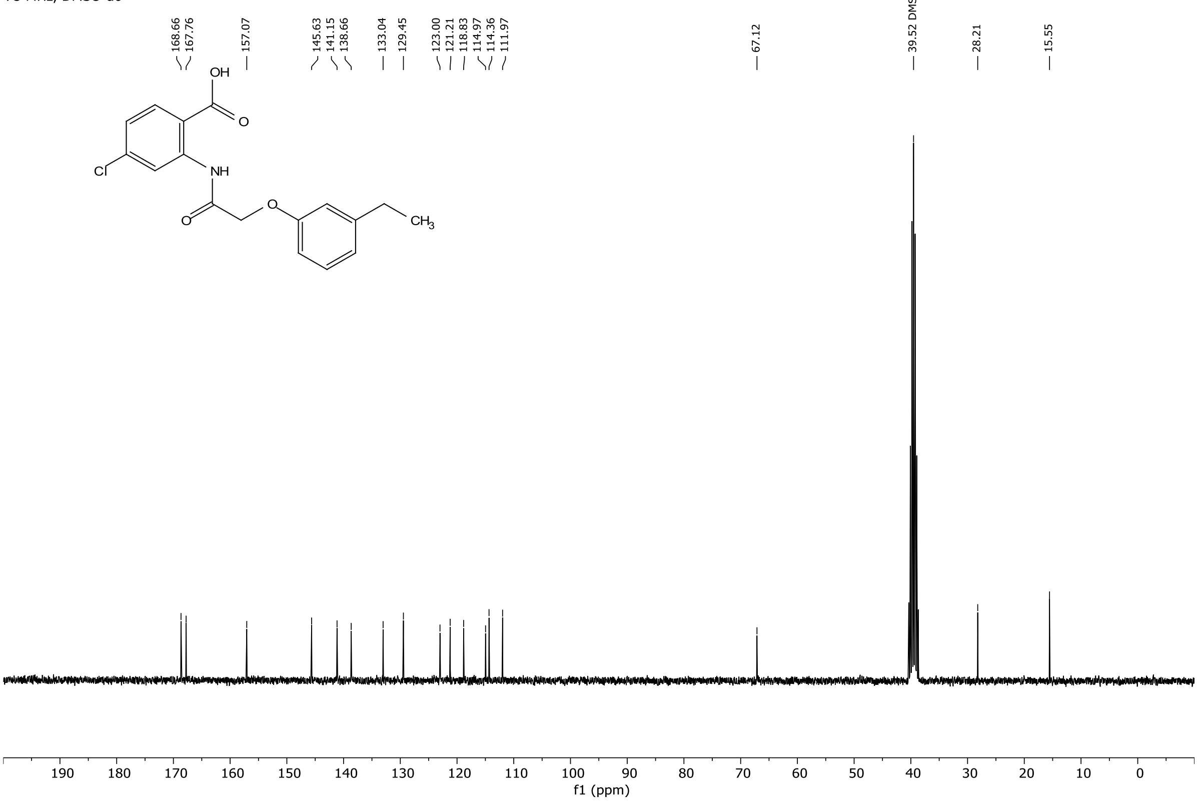

Compound 126
300 MHz, DMSO-d6

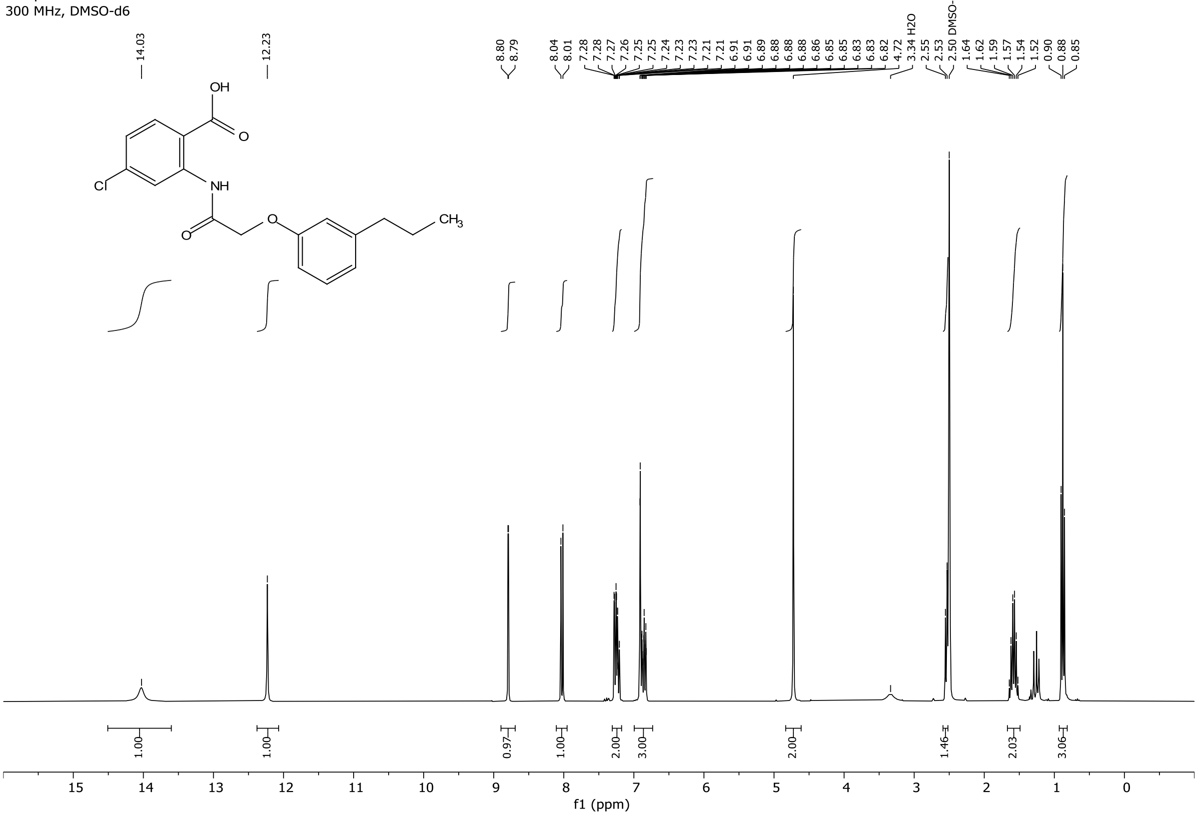

Compound 126
75 MHz, DMSO-d6

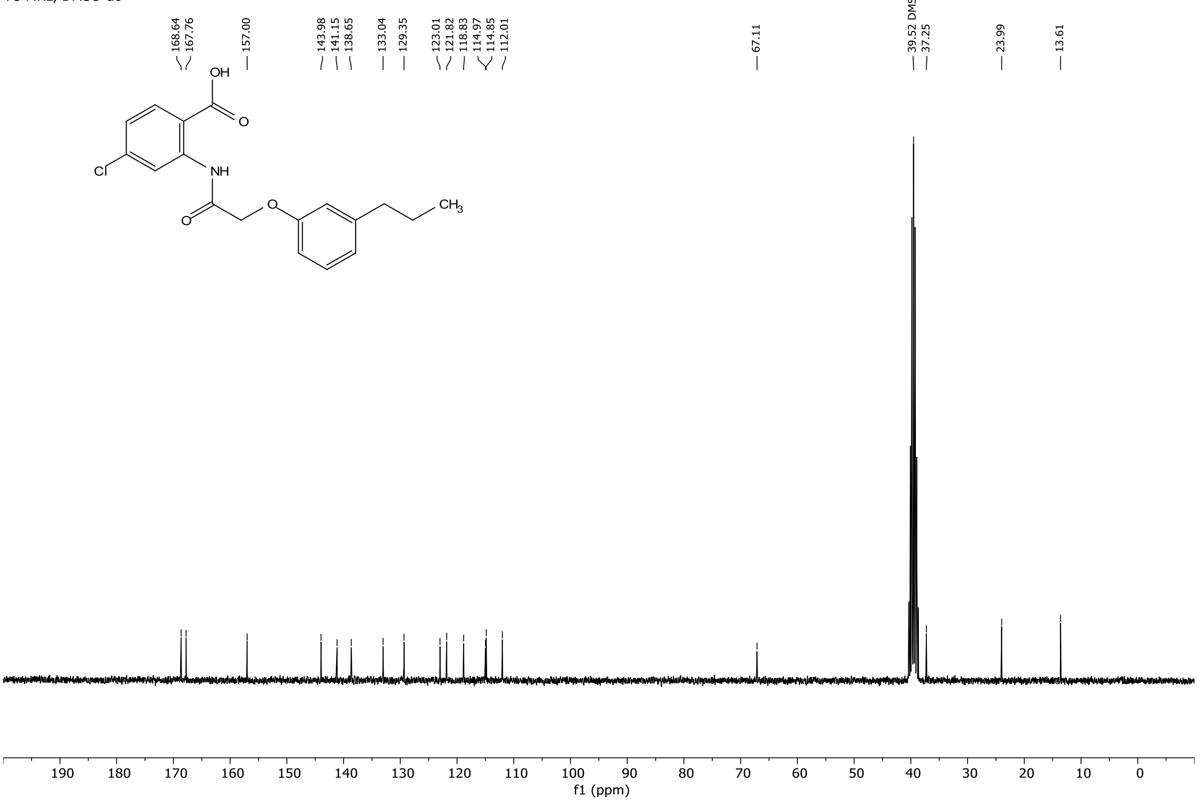

Compound 127
300 MHz, DMSO-d6

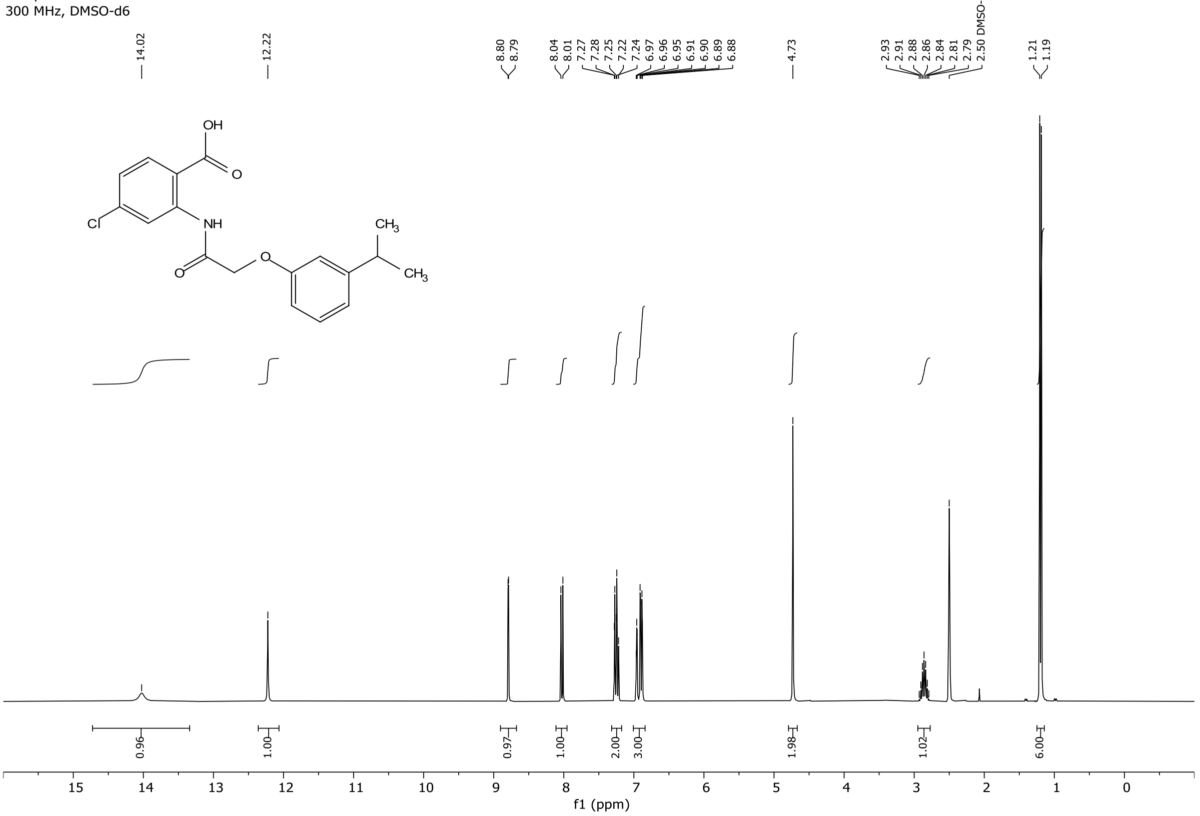

Compound 127
75 MHz, DMSO-d6

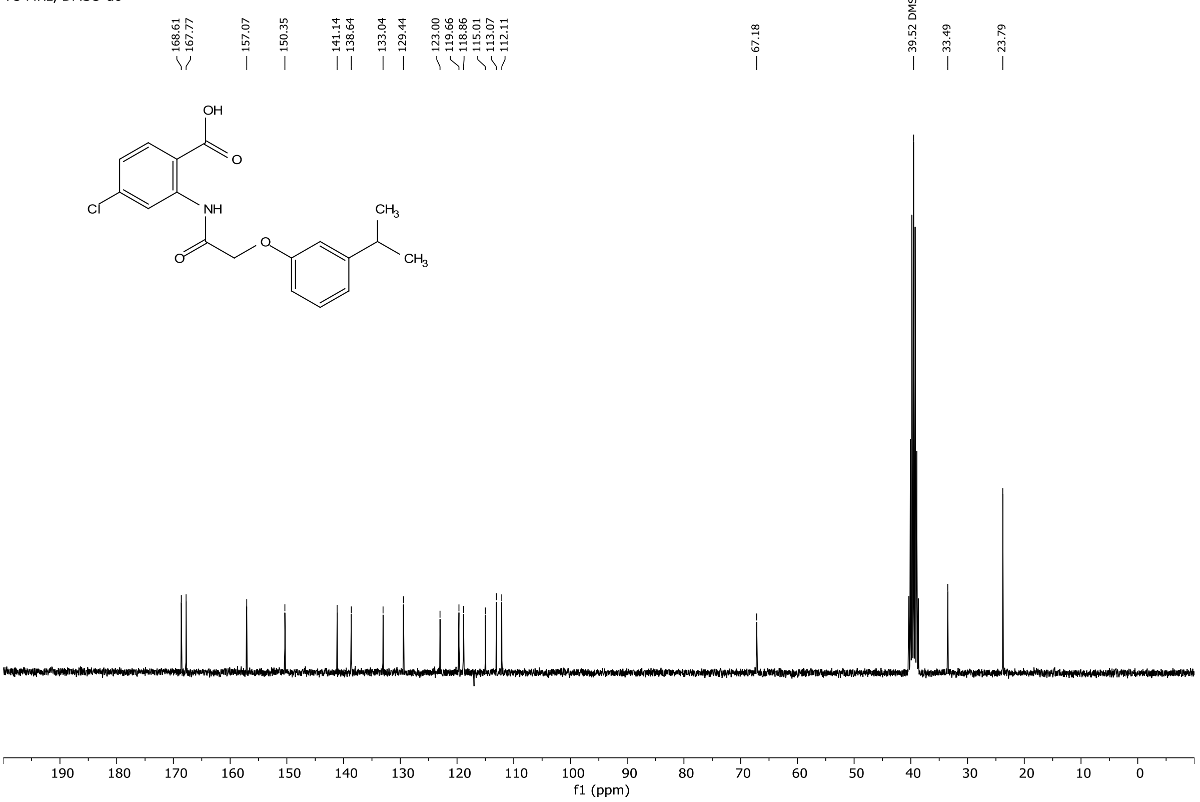

Compound 128
300 MHz, DMSO-d6

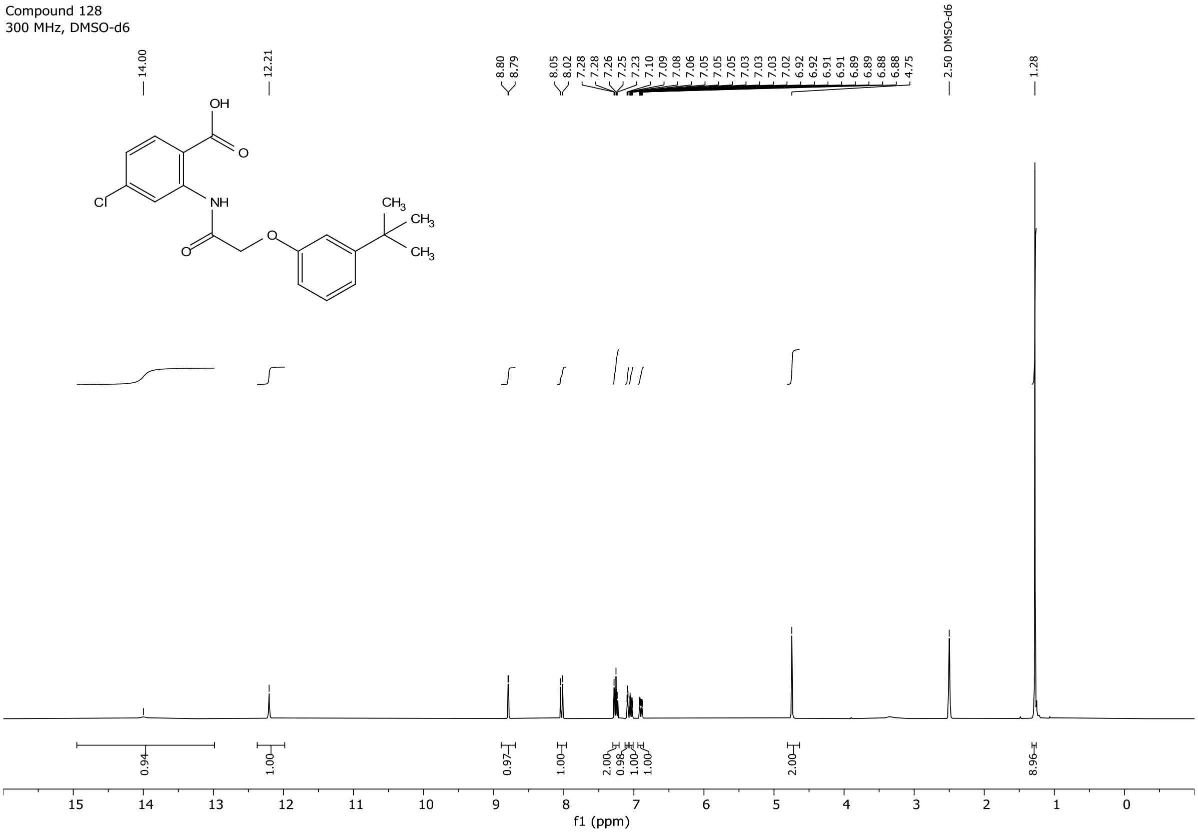

Compound 128
75 MHz, DMSO-d6

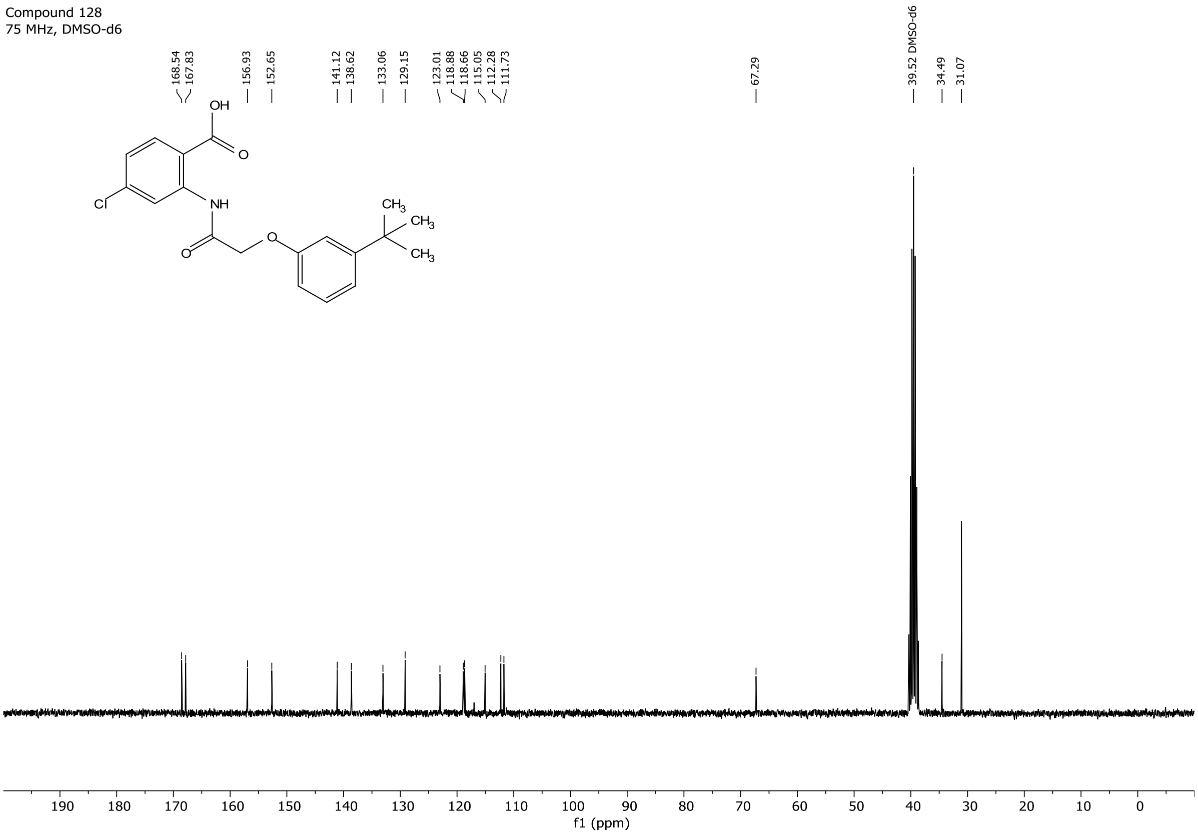

Compound 131
300 MHz, DMSO-d6

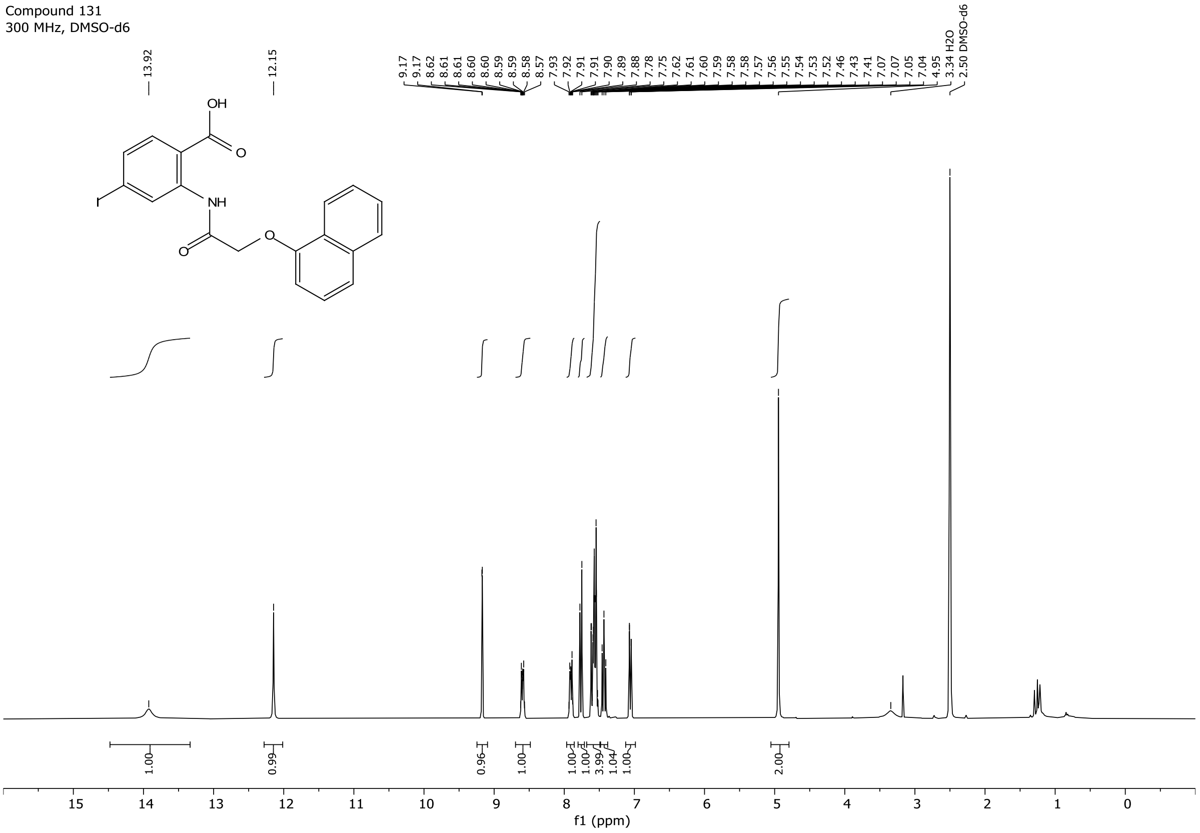

Compound 131
75 MHz, DMSO-d6

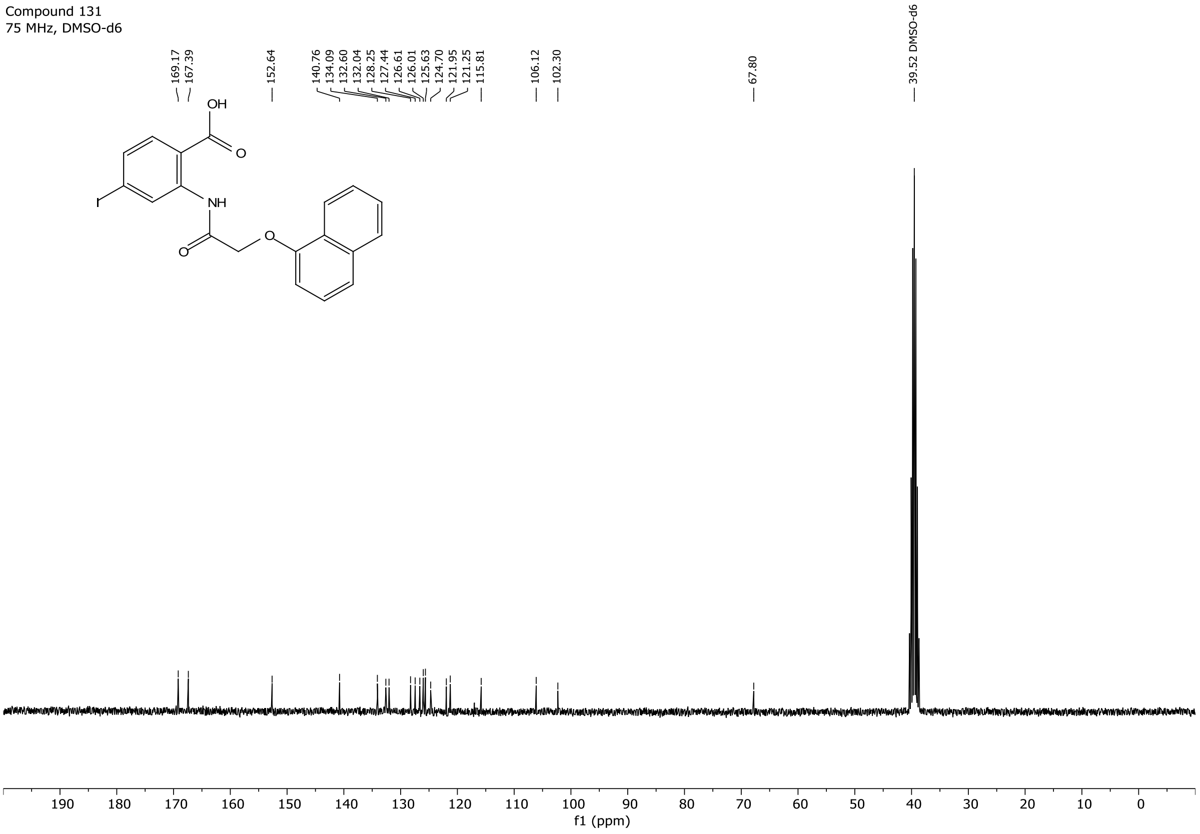

Supplement: Supplementary file 1 [file jm5c02015_si_001.pdf]
